# Increased transcriptional and metabolic capacity for lipid metabolism in the peripheral zone of the prostate may underpin its increased susceptibility to cancer

**DOI:** 10.18632/oncotarget.17926

**Published:** 2017-05-17

**Authors:** Omar Al Kadhi, Maria H. Traka, Antonietta Melchini, Perla Troncoso-Rey, Wiktor Jurkowski, Marianne Defernez, Purnima Pachori, Robert D. Mills, Richard Y. Ball, Richard F. Mithen

**Affiliations:** ^1^ Food and Health Programme, Quadram Institute Bioscience, Norwich, UK; ^2^ Department of Urology, Norfolk and Norwich University Hospitals NHS Foundation Trust, Norwich, UK; ^3^ Integrative Genomics, Earlham Institute, Norwich, UK; ^4^ Analytical Sciences Unit, Quadram Institute Bioscience, Norwich, UK; ^5^ Platforms and Pipelines Bioinformatics, Earlham Institute, Norwich, UK; ^6^ Norfolk and Waveney Cellular Pathology Service, Norfolk and Norwich University Hospital, Norwich, UK

**Keywords:** metabolism, fatty acid metabolism, steroid biosynthesis, prostate zones, prostate cancer

## Abstract

The human prostate gland comprises three distinct anatomical glandular zones, namely the peripheral, central and transitional zones. Although prostate cancer can arise throughout the prostate, it is more frequent in the peripheral zone. In contrast, hyperplasia occurs most frequently in the transitional zone. In this paper, we test the hypothesis that peripheral and transitional zones have distinct metabolic adaptations that may underlie their different inherent predispositions to cancer and hyperplasia. In order to do this, we undertook RNA sequencing and high-throughput metabolic analyses of non-cancerous tissue from the peripheral and transitional zones of patients undergoing prostatectomy. Integrated analysis of RNAseq and metabolomic data revealed that transcription of genes involved in lipid biosynthesis is higher in the peripheral zone, which was mirrored by an increase in fatty acid metabolites, such as lysolipids. The peripheral zone also exhibited increased fatty acid catabolic activity and contained higher level of neurotransmitters. Such increased capacity for *de novo* lipogenesis and fatty acid oxidation, which is characteristic of prostate cancer, can potentially provide a permissive growth environment within the peripheral zone for cancer growth and also transmit a metabolic growth advantage to newly emerging clones themselves. This lipo-rich priming may explain the observed susceptibility of the peripheral zone to oncogenesis.

## INTRODUCTION

Prostate cancer remains the second most commonly diagnosed cancer in males worldwide (15% of all male cancers diagnosed in 2012) [1]. Transcriptomic and metabolomic approaches are being used for the development of new biomarkers for both the early detection of prostate cancer, and to distinguish indolent from aggressive forms of this disease [2–5], but there have been relatively few attempts to integrate these approaches into a single analysis. The prostate gland has several important metabolic adaptations, notably an inhibition of tricarboxylic acid (TCA) cycle activity in epithelial secretory cells resulting in export of citrate into the seminal fluid [6–8], and the ability of prostate cells to accumulate high amounts of cholesterol, which has been attributed to enhanced uptake and synthesis of cholesterol [9]. These metabolic processes, amongst others, are perturbed within prostate tumours.

Among the most prominent changes observed during prostate cancer progression are those occurring in lipid-associated metabolic pathways. Several changes to key gene regulators of lipid metabolism, such as fatty acid synthase (FASN) and alpha-methylacyl-CoA racemase (α- AMACR) have been described in prostate cancer [10–11]. Recent molecular and metabolic profiling of prostate cancer also identifies lipid metabolism as a key pathway that undergoes metabolic reprogramming during prostate cancer development [12–14]. These changes include an upregulation of genes and metabolites responsible for *de novo* lipid biosynthesis and fatty acid β-oxidation, and are likely to facilitate tumour development by meeting energy demand for increased cell proliferation and also by supplying important metabolic intermediates for new cell growth.

The occurrence of cancer is not equally distributed across the three glandular zones of the prostate gland; the peripheral (PZ), central, and transitional (TZ) zones [15]. These zones are located within the same prostatic tissue but in different areas of the prostate and are not easily distinguishable by anatomical or histological means other than their location relative to the urethra. The majority (∼70%) of diagnosed prostate cancer is found in the PZ [16, 17].

In this study we comprehensively measure whole genome expression by next-generation sequencing and combine this with high-throughput metabolic profiling of the PZ and TZ from non-cancerous prostate tissue. Our aim was to identify the molecular and metabolic differences between the two zones that could underlie their differential susceptibility to prostate cancer. Using comprehensive analyses and novel omics integrative approaches we clearly demonstrate, for the first time, that the PZ is innately capable of increased lipid metabolism compared to the TZ, which may predispose the PZ to carcinogenesis and tumour growth. Our study significantly increases our understanding of prostate biology and highlights the unique metabolic nature of the prostate gland.

## RESULTS

### Patient demographics

Clinical characteristics of patients, including the demographic and tumour stage details, are shown in Table 1. Their average age was 62 ± 6.7 years, and the average PSA preoperatively was 8.7 ± 4.2 μg/L. All patients had an American Society of Anaesthesiologists (ASA) co-morbidity score of 2, which reflects the average general health status of men in this age group.

**Table 1:** Demographic and prostate histology characteristics of study patients

				Final prostatectomy histology					
ID	Age	ASA score	Pre-operative PSA (μg/L)	Gleason pattern	Pathological Stage	Tumour zone	Positive margin	PNI	RNAseq of non-cancerous samples
A	54	2	5.9	4+3	T2c	PZ	No	No	
B	64	2	8.8	3+4	T2c	PZ	No	Yes	PZ_9/TZ_9
C	67	2	6	3+4	T2c	PZ	No	Yes	
D	59	2	6.9	3+4	T2c	PZ	No	No	
E	64	2	8.5	3+4	T3a	PZ	Yes	No	
F	67	2	12.3	3+4	T2c	PZ, TZ	No	No	PZ_1
G	57	2	4.5	3+4	T2c	PZ	No	No	
H	64	2	8.5	3+4	T2c	PZ	No	No	
J	52	2	3.4	3+4	T2c	PZ, TZ	No	Yes	
K	73	2	8.5	3+4	T3a	PZ	No	No	TZ_2
L	57	2	4.3	3+4	T2c	PZ	No	No	
N	66	2	10	3+4	T3a	PZ, TZ	Yes	Yes	PZ_5/TZ_5
O	47	2	3.4	3+4	T2a	PZ, TZ	No	No	PZ_3
P	57	2	20	4+3	T2a	PZ	No	No	PZ_4/TZ_4
Q	63	2	14	3+4	T2a	PZ	No	No	
R	67	2	13.2	4+3	T2c	PZ	No	Yes	PZ_6/TZ_6
S	68	2	11.9	4+5	T3a	PZ	No	Yes	TZ_7
T	69	2	7.9	3+4	T2c	PZ	No	Yes	PZ_8/TZ_8

ASA, American society of anaesthesiologist; PSA, Prostate specific antigen; +ve margin, presence of prostate cancer at the resection margin of prostatectomy specimens; PNI, perineural invasion; PZ, peripheral zone; TZ, transitional zone.

### The two prostatic zones are transcriptionally distinct

**Figure 1:**
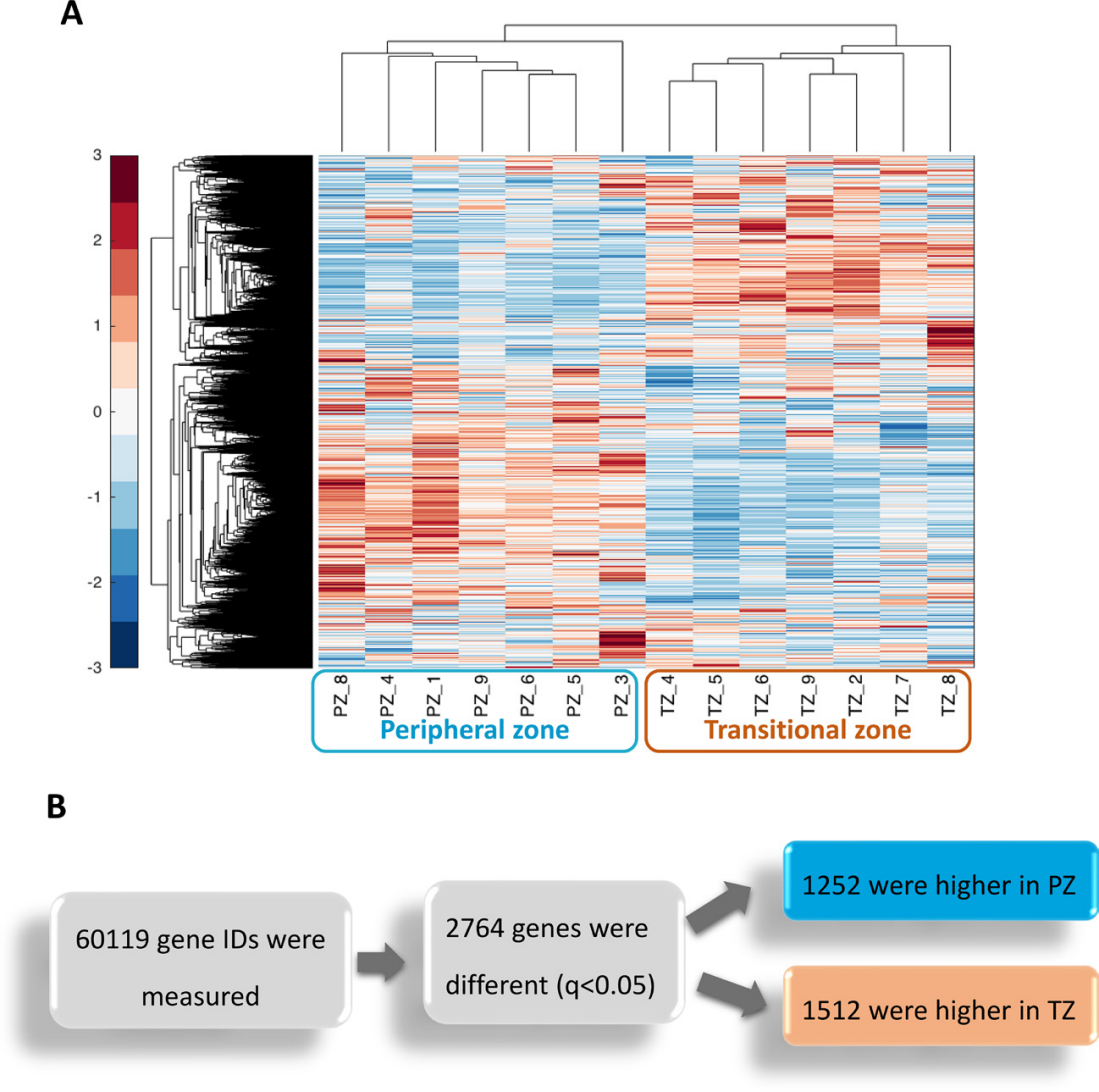
The Peripheral zone (PZ) and Transitional zone (TZ) of the prostate have distinct transcriptional signatures (**A**) Unsupervised hierarchical clustering was performed on normalised expression levels from *Cuffnorm* from prostate tissue derived from non-cancerous samples from nine patients (*n* = 14 samples). Each sample is labelled with the zone of origin (PZ or TZ) and the unique patient number (1–9). Expression values across all samples were standardised per gene, where red denotes the highest expression for that gene and blue denotes the lowest. (**B**) Differentially expressed genes between the PZ and TZ were identified with the *Cuffdiff* programme of the *Cufflinks* suite (*q* < 0.05). Genes measured include coding and non-coding RNAs.

We undertook RNA sequencing analysis of non-cancerous prostate tissue derived from the peripheral and transitional zone of nine patients (seven from PZ and seven from TZ, Table 1) in order to, first, determine whether the two prostate zones differ significantly in their transcriptional profiles, and secondly to identify the genes responsible for any such differences. Unsupervised hierarchical clustering on the normalised expression levels revealed that the two zones are transcriptionally distinct (Figure 1A). In addition, the transcriptional profiles of the respective zones from the prostate specimens were more similar to each other, than were the two zones from the same prostate gland, as evidenced by the distinct clustering of zones rather than clustering of individual patients. Further analysis identified a total of 2,764 transcripts that were differentially expressed between the two zones after multiple testing corrections (FDR *q* < 0.05). Of these 1,252 were higher in the peripheral zone compared to the transitional zone (Figure 1B).

**Figure 2:**
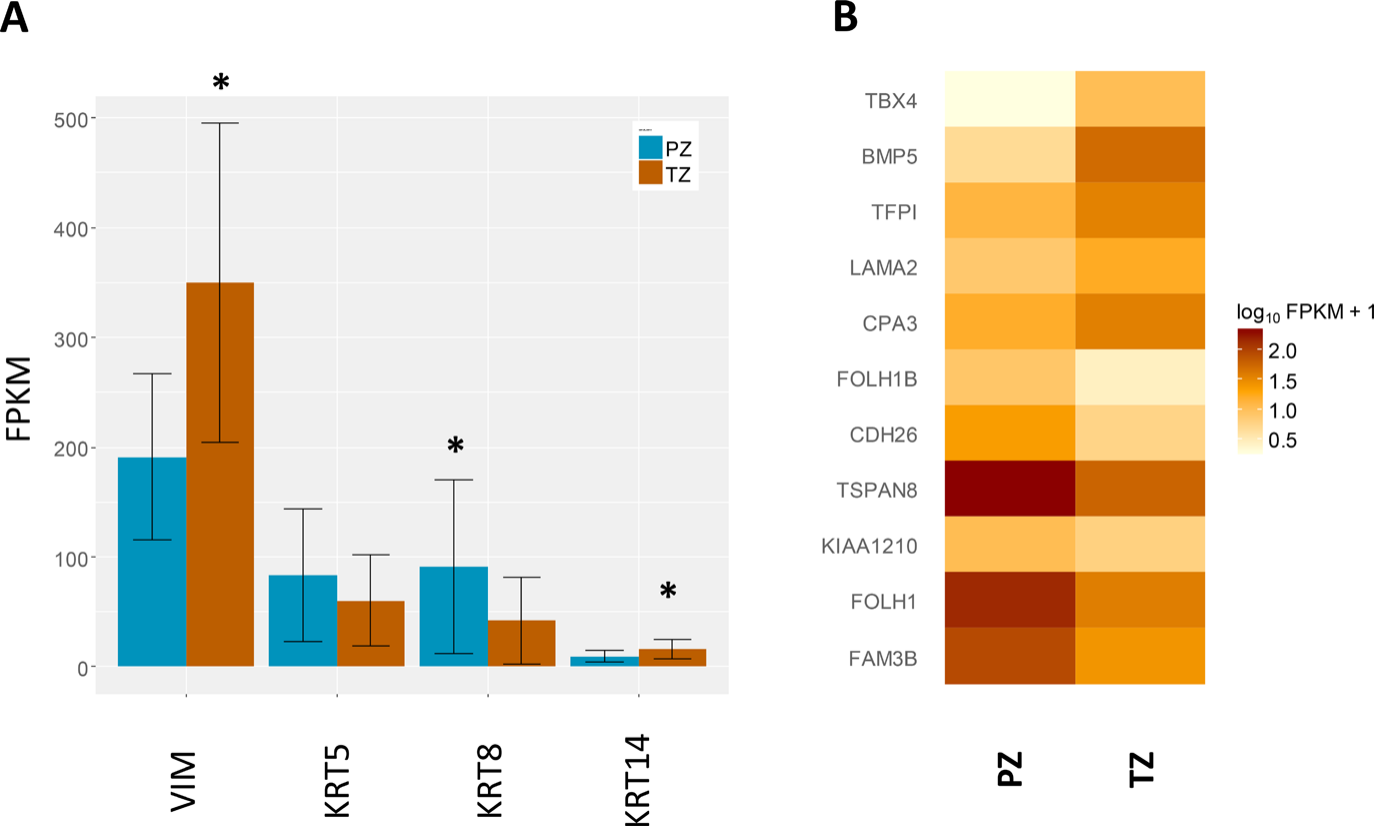
Expression of genes of interest (**A**) Expression of cell type specific markers, including the stromal marker (vimentin, VIM), basal markers (keratin (KRT)5 and KRT14), and luminal marker (KRT8). Data shown are mean transcript abundances reported in fragments per kilobase of transcript per million fragments sequenced (FPKM) units ± SD, *q-value < 0.01. (**B**) Expression of a previously identified 11-gene signature determining zone of origin. A heatmap depiction of mean FPKM values from each zone for the 11-gene signature previously identified by Sinnot and colleagues [18]. The comparative expression between the two zones mirrors that reported previously.

We then sought to identify whether there was an enrichment of a specific cell type within each zone. The expression of the stromal marker (vimentin, VIM), the basal markers (keratin (KRT)5 and KRT14), and the luminal cell marker (KRT8) were extracted from the RNAseq data (Figure 2A). VIM expression was higher in the TZ samples by 1.8-fold (q < 0.001), whereas expression of KRT8 was lower by 2-fold (q <0.005). These reflect the zonal cell distribution, characterised by increased stromal composition in the transitional zone compared to the peripheral zone.

In order to independently validate the zonal origin of our samples we used an 11-gene signature previously shown to classify zone of origin of prostate cancer samples [18]. Principal component analysis (PCA) separated accurately the two zones using the 11-gene signature (Supplementary Figure 1), confirming our sampling, and also further supporting this diagnostic gene signature. Moreover, the comparative expression of the eleven genes between the two zones from our study correlated accurately with the direction of change previously reported [18] (Figure 2B).

### Transcription of genes involved in fatty acid metabolism is higher in the peripheral zone

**Figure 3:**
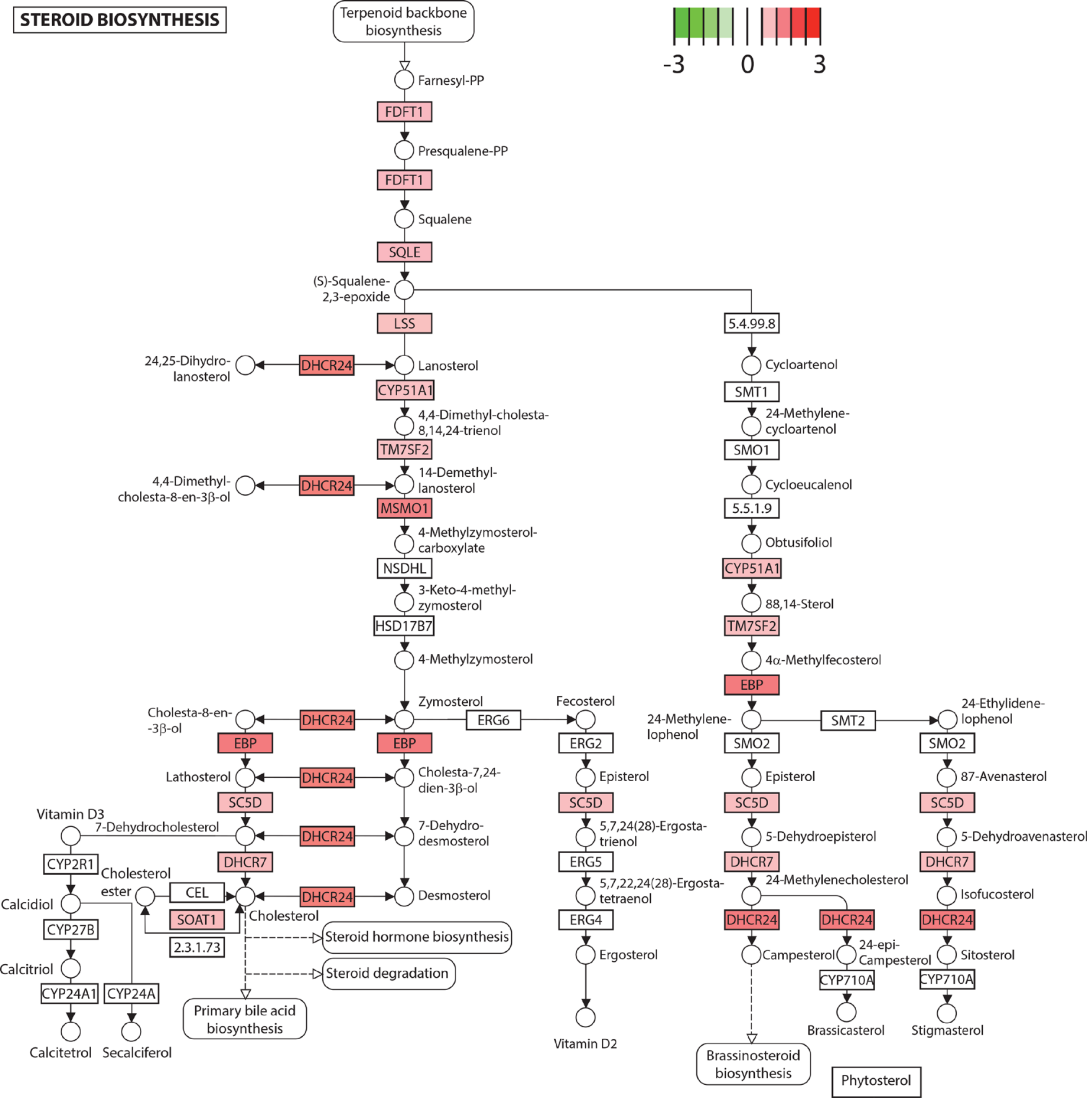
Steroid biosynthesis is upregulated in the peripheral zone Several genes in the steroid biosynthesis pathway exhibited higher expression in the peripheral compared to the transitional zone, depicted in red on the KEGG pathway (log_2_(fold change), *q* < 0.05). Genes are depicted as boxes and metabolites as circles. CY51A1: cytochrome P450 family 51 subfamily A member 1. DHCR7: 7-dehydrocholesterol reductase. DHCR24: 24-dehydrocholesterol reductase. EBP: emopamil binding protein (sterol isomerase). FDFT1: farnesyl-diphosphate farnesyltransferase 1. LSS: lanosterol synthase (2,3-oxidosqualene-lanosterol cyclase). MSMO1: methylsterol monooxygenase 1. SC5D: sterol-C5-desaturase. SOAT1: sterol O-acyltransferase 1. SQLE: squalene epoxidase. TM7SF2: transmembrane 7 superfamily member 2.

In order to identify whether the distinct gene signatures between the non-cancerous PZ and TZ are associated with particular biological processes, we performed functional analysis to identify pathways that may be overrepresented in each zone. We found that the genes that were relatively more highly expressed in the non-cancerous PZ (*q*-value < 0.05, 1252 genes) were enriched in functions related to fatty acid metabolism and steroid biosynthesis (Table 2). These pathways remained significant when over-representation analysis was undertaken on more stringent expression lists (*q*-value < 0.01, 833 genes) and on less stringent expression lists (*q*-value < 0.1, 1500 genes). These included the major metabolic lipid enzymes involved in *de novo* lipogenesis, FASN, acetyl-CoA carboxylase isoform alpha (ACACA), acyl-CoA synthetase long-chain (ACSL)1 and ACSL3, which were upregulated by 2.8-, 1.4-, 2.0-, and 1.9-fold respectively (Table 3). Several genes involved in cholesterol and steroid biosynthesis were also increased, including farnesyl-diphosphate farnesyltransferase 1 (FDFT1) and 24-dehydrocholesterol reductase (DHCR24), which were upregulated by 1.8- and 2.4-fold respectively (Figure 3, Table 3).

**Table 2:** Top fifteen pathways upregulated in the peripheral zone compared to the transitional zone

KEGG pathway	Genes^1^	Fold Enrichment	ORA score^2^
Metabolic pathways^3^	141	1.6	9.53E-08
Protein processing in endoplasmic reticulum	33	2.8	2.20E-05
Steroid biosynthesis	11	7.7	2.59E-05
Biosynthesis of antibiotics	34	2.2	9.69E-04
Valine, leucine and isoleucine degradation	13	3.9	0.004
Fatty acid metabolism	12	3.5	0.0187
Terpenoid backbone biosynthesis	8	5.1	0.0222
Glutathione metabolism	11	3.0	0.0870
Vibrio cholerae infection	11	2.9	0.1031
Peroxisome	14	2.4	0.1231
Lysosome	18	2.1	0.1258
Mucin type O-Glycan biosynthesis	7	3.2	0.3231
beta-Alanine metabolism	7	3.2	0.3231
PPAR signaling pathway	11	2.3	0.3281
Aldosterone-regulated sodium reabsorption	8	2.9	0.3422

^1^Input list was the 1252 genes expressed higher in the peripheral zone (*q*-value < 0.05, see Figure 1).

^2^Over-representation analysis (ORA) score (see Materials & Methods for details)

^3^Refers to the ‘Global and overview map’ hsa01100 as defined by KEGG [57].

**Table 3:** Expression of genes involved in fatty acid metabolism and steroid biosynthesis pathways that were upregulated in the peripheral zone

Gene name	Gene Symbol	Fold change	*q* value
fatty acid synthase	FASN	2.9	0.001
ELOVL fatty acid elongase 2	ELOVL2	2.8	0.001
stearoyl-CoA desaturase	SCD	2.6	0.001
emopamil binding protein (sterol isomerase)	EBP	2.5	0.001
24-dehydrocholesterol reductase	DHCR24	2.4	0.001
methylsterol monooxygenase 1	MSMO1	2.4	0.001
fatty acid desaturase 2	FADS2	2.3	0.001
7-dehydrocholesterol reductase	DHCR7	2.2	0.001
acyl-CoA synthetase long-chain family member 1	ACSL1	2.1	0.001
cytochrome P450 family 51 subfamily A member 1	CYP51A1	2.0	0.001
acyl-CoA synthetase long-chain family member 3	ACSL3	2.0	0.001
transmembrane 7 superfamily member 2	TM7SF2	2.0	0.001
sterol-C5-desaturase	SC5D	2.0	0.001
farnesyl-diphosphate farnesyltransferase 1	FDFT1	1.9	0.001
squalene epoxidase	SQLE	1.9	0.001
acyl-CoA dehydrogenase, long chain	ACADL	1.9	0.001
sterol O-acyltransferase 1	SOAT1	1.9	0.001
lanosterol synthase (2,3-oxidosqualene-lanosterol cyclase)	LSS	1.8	0.001
ELOVL fatty acid elongase 5	ELOVL5	1.6	0.001
acetyl-CoA carboxylase alpha	ACACA	1.4	0.040
acyl-CoA oxidase 3, pristanoyl	ACOX3	1.4	0.047
acyl-CoA dehydrogenase, short/branched chain	ACADSB	1.4	0.016
enoyl-CoA hydratase, short chain, 1, mitochondrial	ECHS1	1.3	0.038

In contrast, genes that were relatively more highly expressed in the non-cancerous TZ belonged to a variety of pathways predominantly involved in signal transduction and immune regulation (Supplementary Table 1) and were enriched in Gene Ontology functions of extracellular matrix organisation and immune response and signal transduction (data not shown).

### Metabolites involved in *de novo* lipid biosynthesis are higher in the peripheral zone

PCA analysis of metabolic profiles from the PZ and TZ of 18 men (a total of 36 samples) did not clearly differentiate the two zones (Supplementary Figure 2A). Subsequent partial least square-discriminant analysis (PLS-DA) gave clearer separation but this was not statistically significant (data not shown). This is likely due to the influence of other factors, notably inter-individual differences.

We then used N-way ANOVA to identify the metabolites that were significantly different between the two prostate zones, having factored in the structure of the data (multiple measurements per patient). This identified 50 metabolites that were different between the zones (*p* < 0.05), of which 44 were higher in the PZ and 6 higher in the TZ (Table 4).

**Table 4:** List of statistically significant differences in metabolite concentrations between the two prostate zones

Compound^1^	Pathway	Prostate zone with greater concentration	*P* value^2^
**Amino Acid**			
N-acetyl-aspartyl-glutamate (NAAG)	Glutamate metabolism	PZ	< 0.001
Serotonin	Tryptophan metabolism	PZ	< 0.001
Gamma-aminobutyrate (GABA)	Glutamate metabolism	PZ	0.003
Ophthalmate	Glutathione metabolism	PZ	0.007
Isobutyrylcarnitine	Leucine, isoleucine and valine metabolism	PZ	0.008
Hypotaurine	Methionine, cysteine, SAM and taurine metabolism	PZ	0.014
Spermidine	Polyamine metabolism	PZ	0.018
Tryptophan betaine	Tryptophan metabolism	PZ	0.021
Phenylacetylglutamine	Phenylalanine and tyrosine metabolism	PZ	0.024
Glutathione, reduced (GSH)	Glutathione metabolism	PZ	0.029
5-oxoproline	Glutathione metabolism	PZ	0.035
3-indoxyl sulfate	Tryptophan metabolism	PZ	0.045
**Carbohydrate**			
Lactate	Glycolysis, gluconeogenesis, and pyruvate metabolism	TZ	0.046
Energy			
Succinylcarnitine	TCA Cycle	PZ	< 0.001
**Lipid**			
Acetylcholine	Neurotransmitter	PZ	< 0.001
1-(1-enyl-palmitoyl)-GPE (P-16:0)	Lysolipid	PZ	< 0.001
Glycerophosphorylcholine (GPC)	Phospholipid metabolism	PZ	< 0.001
1-palmitoyl-GPE (16:0)	Lysolipid	PZ	< 0.001
1-oleoyl-GPE (18:1)	Lysolipid	PZ	0.001
1-palmitoyl-GPG (16:0)	Lysolipid	PZ	0.001
1-linoleoyl-GPE (18:2)	Lysolipid	PZ	0.001
Glycerophosphoethanolamine	Phospholipid metabolism	PZ	0.001
Docosapentaenoate (n6 DPA; 22:5n6)	Polyunsaturated fatty acid (n3 and n6)	PZ	0.002
Choline phosphate	Phospholipid metabolism	TZ	0.004
1-(1-enyl-stearoyl)-GPE (P-18:0)	Lysolipid	PZ	0.004
1-stearoyl-GPS (18:0)	Lysolipid	PZ	0.004
Octanoylcarnitine	Fatty acid metabolism (acyl carnitine)	PZ	0.008
1-oleoyl-GPI (18:1)	Lysolipid	PZ	0.008
Dihomo-linolenate (20:3n3 or n6)	Polyunsaturated fatty acid (n3 and n6)	PZ	0.01
Dihomo-linoleate (20:2n6)	Polyunsaturated fatty acid (n3 and n6)	PZ	0.01
1-palmitoleoyl-GPC (16:1)	Lysolipid	PZ	0.01
Eicosenoate (20:1)	Long chain fatty acid	PZ	0.01
1-palmitoyl-GPI (16:0)	Lysolipid	PZ	0.014
1-stearoyl-GPI (18:0)	Lysolipid	PZ	0.015
Sphingosine	Sphingolipid metabolism	PZ	0.025
1-stearoyl-GPC (18:0)	Lysolipid	PZ	0.026
Oleoylcarnitine	Fatty acid metabolism (acyl carnitine)	PZ	0.035
Deoxycarnitine	Carnitine metabolism	PZ	0.039
1-linoleoyl-GPC (18:2)	Lysolipid	PZ	0.042
1-(1-enyl-oleoyl)-GPE (P-18:1)	Lysolipid	PZ	0.046
1-arachidonoyl-GPE (20:4)	Lysolipid	PZ	0.048
Stearoylcarnitine	Fatty acid metabolism (acyl carnitine)	PZ	0.05
1-palmitoyl-GPC (16:0)	Lysolipid	PZ	0.05
**Nucleotide**			
3-aminoisobutyrate	Pyrimidine metabolism, thymine containing	PZ	< 0.001
N1-methyladenosine	Purine metabolism, adenine containing	PZ	0.001
Guanosine	Purine metabolism, guanine containing	TZ	0.002
Inosine	Purine metabolism, (hypo)xanthine/inosine containing)	TZ	0.003
Pseudouridine	Pyrimidine metabolism, uracil containing	TZ	0.01
Cytidine 5′-monophosphate (5′-CMP)	Pyrimidine metabolism, cytidine containing	PZ	0.02
Adenosine	Purine metabolism, adenine containing	TZ	0.05

GPE, glycerol-3-phosphorylethanolamine; GPG, glycero-3-phosphoglycerol; GPI, glycerol-3-phosphoinositol; PZ, peripheral zone; TZ, transitional zone.

^1^Excludes xenobiotics.

^2^*p* values from N-way ANOVA.

**Figure 4:**
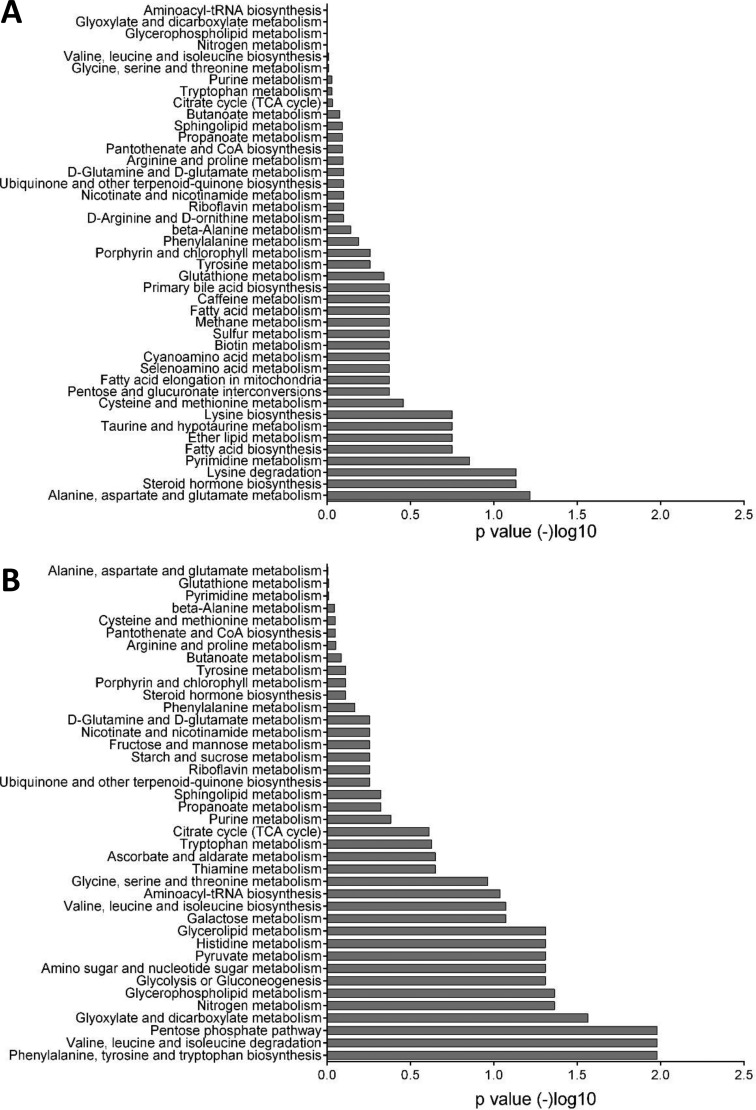
Enrichment analysis of metabolomics data (**A**) Pathways that are over-represented in the peripheral zone. Glutamate metabolism, steroid hormone biosynthesis, and fatty acid biosynthesis were among the highly significant pathways. (**B**) Pathways that are over-represented in the transitional zone. Phenylalanine, pentose phosphate pathway, and pyruvate metabolism are highly significant. Each bar represents the *p* value of each pathway expressed as –log10.

We performed enrichment analysis of the metabolites that were upregulated in each zone to give us an insight into the pathways that are involved. Similar to the transcriptional data, steroid hormone biosynthesis and fatty acid biosynthesis were among the most overrepresented pathways in the PZ, compared to phenylalanine biosynthesis and the pentose phosphate pathway, both of which were over-represented in the TZ (Figure 4, Supplementary Table 2).

Interestingly, we also observed increased levels of three neurotransmitters in the PZ, namely N-acetyl-aspartyl-glutamate (NAAG), serotonin (5-HT), and acetylcholine (Ach).

### Integration of transcriptional and metabolomics data

**Figure 5:**
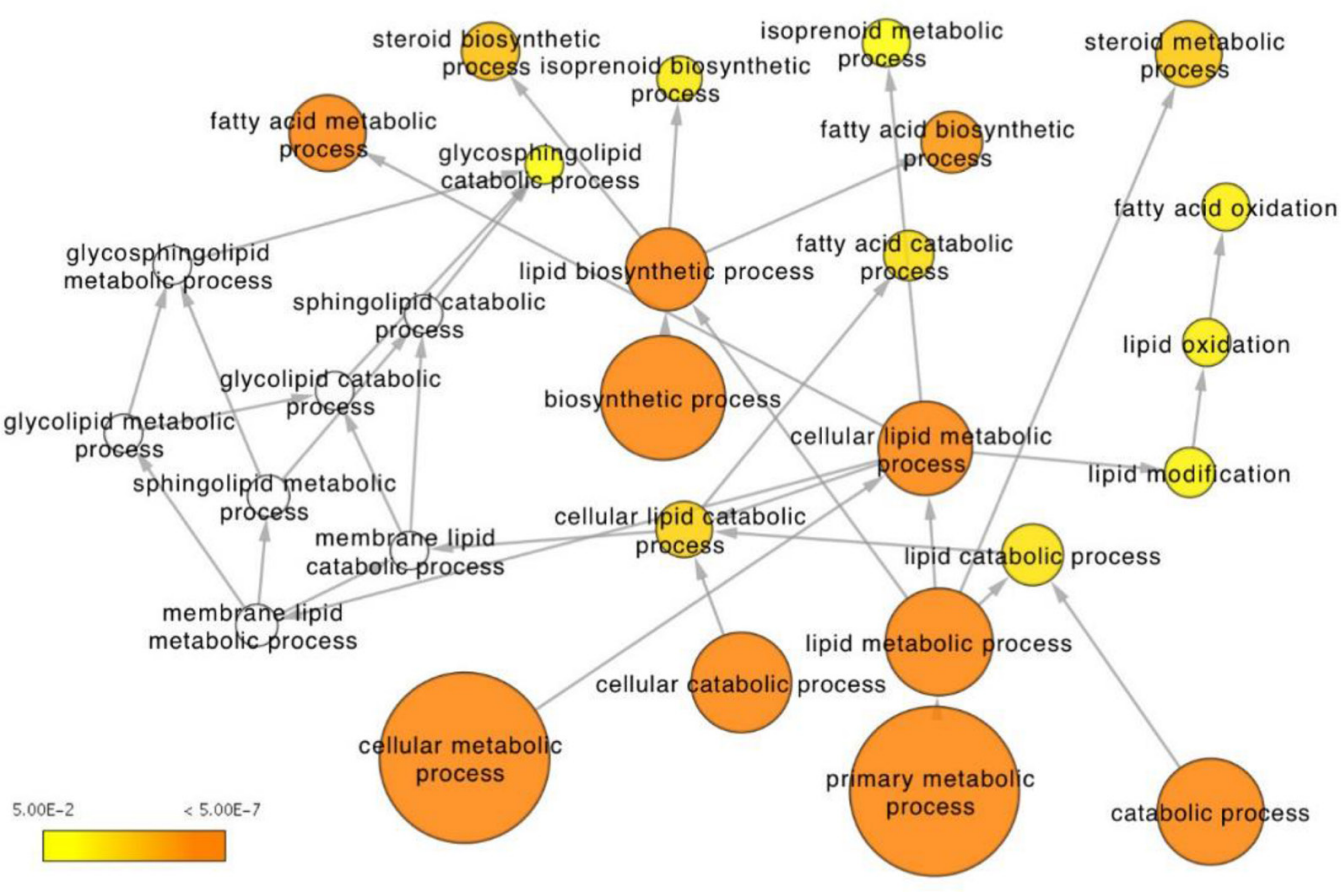
Integrative analysis of RNAseq expression and metabolomics data from the peripheral and transitional zones Depicted is the lipid metabolism Gene Ontology tree with enriched functional terms that are significantly different between the two zones (*p* < 0.05 for metabolites, *q* < 0.05 for genes). Node size corresponds to number of genes that belong to the GO term. Node color represents statistical significance: white – not significant, yet some genes present, in most cases significant before multiple testing correction; scale yellow to orange (from 5E-2 to 5E-7) – the darker the orange the more significant after multiple testing correction.

Network-based integrative analysis of the combined RNAseq and metabolomics data further supported the strong differences in fatty acid and steroid metabolism between the two zones identified by the individual analyses. In particular, over-representation analysis of biological processes finds lipid and fatty acid metabolism pathways amongst the most significant between the PZ and TZ with a high proportion of differentially active molecules (Figure 5, Supplementary Table 3). In addition, integrative analysis of the combined datasets performed better in identifying lipid metabolism as a key pathway differentiating the PZ and TZ, compared to RNAseq data alone (rank 16 compared to rank 50; Supplementary Tables 3 and 4). Genes and metabolites differentially expressed between the PZ and TZ are forming distinct clusters in the human molecular interactome network closely related with lipid metabolism (Supplementary Figure 3).

## DISCUSSION

Although cancer is more likely to arise in the peripheral than the transitional zone of the prostate, there is currently little understanding of the origins of such a difference. As there is no clear significant anatomical difference, it is likely that such susceptibility is driven by molecular differences. Earlier reports using microarray-based genome-wide expression profiling between the two zones identified some genes but with limited functional interpretation [18–20]. In this report, we have employed whole-genome RNA sequencing and parallel high-throughput metabolite profiling of non-cancerous prostate tissue derived from the peripheral and transitional zones to comprehensively study the molecular and metabolic differences that underpin the increased susceptibility of the PZ to develop cancer. In addition, we have applied novel ‘omics integration bioinformatics analysis to improve our predictions for the differential pathways that characterise each zone. With the use of genome sequencing and comprehensive metabolomics analysis of the two zones we have shown that the PZ has a unique transcriptional and metabolic signature that is characterised by increased capacity for both fatty acid and steroid biosynthesis. We have demonstrated that the PZ has an almost 3-fold increased expression of FASN, which encodes the enzyme that catalyzes the formation of long-chain fatty acids from acetyl-CoA. FASN gene expression is linked to the content of fatty acids in non-cancerous prostate tissue [21], and is consistent with the increased levels of phospholipids seen by metabolomics analysis. High FASN expression is associated with prostate cancer progression [22] and is linked to higher Gleason score both at the gene [23] and protein [24] level. Additionally, gene expression of ACACA, the rate-limiting enzyme in FA biosynthesis, was also higher in the PZ. Inhibition of ACACA by RNAi led to inhibition of LNCaP prostate cancer cell growth and subsequent *de novo* lipogenesis [25].

Increased *de novo* fatty acid and cholesterol synthesis is one of the features of prostate cancer cells [26, 27] and acts as a source of lipids for membrane production but also of signalling molecules activating oncogenic pathways, such as the phosphoinositide 3-kinase (PI3K) pathways [28]. The bulk of cell membrane lipids are phospholipids (PLs), such as phosphatidylcholine (PC) and phosphatidylethanolamine (PE), and other lipids, such as sterols, sphingolipids, and lyso-PLs. Lysolipids in particular have been suggested as biomarkers for prostate cancer [29]. It is notable that our metabolomics analysis identified several such lipids to be higher in the PZ compared to TZ, which supports a higher *de novo* lipogenesis activity in the PZ. More recently, increased lipogenesis in prostate cancer tumours was also found to be protective from endogenous and exogenous insults that lead to oxidative stress–induced cell death [30]. This could be another manner by which newly emerging cancerous clones in the PZ are evading elimination.

A significant proportion of the genes leading to cholesterol and steroid biosynthesis were higher in the PZ compared to the TZ. These included FDFT1, which is expressed at higher levels in human prostate cancer specimens and in aggressive cancers [31], and DHCR24, also known as seladin-1, an androgen regulated gene that is expressed significantly higher in prostate cancer and is positively related to T stage [32]. Circulating levels of cholesterol as well as enhanced cholesteryl ester accumulation in cellular lipid droplets are positively associated with prostate cancer and aggressiveness [33, 34]. These results are consistent with reports that pharmaceutical interventions, e.g. statins that target cholesterol metabolism, are associated with reduced prostate cancer recurrence [35, 36].

Increased FA biosynthetic activity in the PZ was coupled with increased FA catabolic capability. ACSL1 and ACSL3 were highly expressed in the PZ compared to the TZ by more than 2-fold. The protein encoded by the ACSL3 gene is an isozyme of the long-chain fatty-acid coenzyme A ligase family that converts free long-chain fatty acids into fatty acyl-CoA esters that then enter fatty acid β-oxidation for subsequent catabolism. ACSL3 is an androgen receptor (AR)-stimulated gene [37] and was recently identified in formalin-fixed prostate cancer tissue as a new 5′ translocation partner of ETS variant 1 (ETV1), an important gene in driving prostate cancer progression [38, 39]. Interestingly, pristanoyl-CoA oxidase 3 (ACOX3), encoding an enzyme involved in peroxisomal β-oxidation, was also higher in the PZ. Consistent with the gene expression, higher levels of several acyl carnitines were found in the PZ. These medium and long-chain acyl carnitines contain FAs and are mediating their transport into mitochondria for β-oxidation. Acyl carnitines are sourced from diet as well as produced endogenously, and have been linked to prostate cancer and associated with aggressive forms of the disease [29, 40–42]. Increased mitochondrial and peroxisomal FA β-oxidation is a feature of prostate cancer progression and is likely a unique metabolic adaptation that provides ATP and acetyl-CoA in prostate cancer [13, 43, 44].

Interestingly, we also found increased levels of three neurotransmitters in the PZ, namely N-acetyl-aspartyl-glutamate (NAAG), serotonin (5-HT), and acetylcholine (Ach). NAAG is a neurotransmitter that is found in abundance in the mammalian nervous system. It is catabolised to glutamate and N-acetyl-L-aspartate (NAA) by two groups of glutamate carboxypeptidases (GCP II and III). Both GCPs are membrane bound and have also been identified in the human prostate. GCPII in the prostate is called prostate specific membrane antigen (PSMA) [45] and although is expressed in both benign and cancerous prostate tissue, the degree of expression is higher in prostate cancer and increases with cancer severity [46]. Radiolabelled indium coupled with an anti-PSMA antibody has shown some promise in detecting prostate cancer recurrence after surgery (ProstaScint^®^ scan, Cytogen Corporation, US). Inhibitors of these GCPs have also shown anti-tumour properties in animal models of prostate cancer [47]. There are no studies comparing the immunohistochemical staining of PSMA in different zones of the prostate, however, the abundance of NAAG in non-cancerous PZ tissue could indicate the low activity of GCPII. McDunn and colleagues found the breakdown product NAA to be associated with organ-confined prostate cancer when compared to cancer that had extended beyond the capsule of the prostate, indicating more catabolism of NAAG with advanced disease [48].

Levels of 5-HT were also significantly higher in the PZ. 5-HT is a metabolite derived from tryptophan, and functions as a neurotransmitter but also has growth promoting properties in several cancer cell lines, including androgen resistant prostate cancer [49–51]. In hormone resistant prostate cancer, neuroendocrine (NE)-like cells emerge and are associated with poor prognosis. NE cells typically secrete 5-HT, amongst other neurotransmitters, that are thought to have an autocrine as well as a paracrine effect, promoting cell growth and migration [52]. Higher levels of 5-HT in the PZ therefore could favour the growth and migration of cancer. Finally, Ach was also found to be higher in the PZ, compared to the TZ. Ach is a neurotransmitter associated with the parasympathetic nervous system, but increased levels were recently linked to high-grade/advanced prostate cancer in both animal models and in human tissue [53]. Although increased neurotransmitter metabolites in the PZ could represent the abundance of nerve tissue in this part of the prostate compared to the TZ, their emerging role in prostate cancer development and progression could also indicate another metabolic feature of the PZ that could explain cancer predilection to this part of the gland. Further studies are required to confirm these findings.

One limitation of the study is that the non-cancerous samples were obtained from patients with confirmed prostate cancer, albeit the sampling site was histologically confirmed to be cancer-free. Such prostates could potentially have acquired an altered metabolic profile as part of the carcinogenesis process. If that was the case, we could expect such metabolic alterations to be universally acquired across the prostate, spanning both the peripheral and transitional zones. Our data show that there are major differences in the metabolic and transcriptional profiles of these two zones that have a direct relevance for prostate cancer initiation and growth.

One explanation for the observed transcriptional differences between the two zones could be the inherent differences in the cellular composition of the zones, namely the peripheral zone being epithelial cell-rich and the transitional zone being stromal cell-rich. Such compositional differences could be the origin of the molecular differences between the two zones but do not detract from the observation that increased transcriptional and metabolic capacity for lipid metabolism is a characteristic of the peripheral zone.

Recently, the genetic complexity of morphologically normal prostate tissue was uncovered using genome-wide DNA sequencing and suggested that mutational processes, consistent with field effects, are underlying prostate carcinogenesis [54]. In our study, we extended this observation to include genome-wide RNA sequencing and high-throughput metabolic profiling of morphologically normal prostate tissue distant from cancer. We have focused on the apparent differences in the two major zones of the prostate, the peripheral and the transitional zones, and have identified the potential underlying mechanisms for the increased susceptibility of the PZ to prostate cancer. We propose that the PZ is constitutionally primed for metabolic reprogramming that is vital for prostate cancer progression and is characterised by increased *de novo* FA synthesis, FA catabolism and increased levels of neurotransmitters. Such ‘priming’ can benefit prostate cancer initiation and progression in two ways. First, by providing a permissive environment, full of vital cell growth intermediates, for novel cancer clones to arise and thrive, and secondly, by transmitting a metabolic advantage to the newly emerging clones themselves. In this way, the clones that arise in the PZ have an advantage over those arising in the TZ.

## MATERIALS AND METHODS

### Patient selection and sampling

Eighteen patients undergoing radical prostatectomy for organ confined prostate cancer were consented to allow the use of prostate tissue for research via the Biorepository at the Norfolk and Norwich University Hospital (NNUH) under ethical approval granted from the Faculty of Medicine and Health Sciences Research Ethics Committee of the University of East Anglia (FMHS 20122014–37). Patients underwent endoscopic extraperitoneal radical prostatectomy. All procedures were conducted by a single surgeon. The prostate gland was removed from the abdominal cavity immediately after resection and rapidly biopsied after extraction to avoid ischaemic artefacts using a standard core biopsy instrument. The extracted whole prostatectomy specimen was placed on a surgical table or equivalent and the subsequent steps were taken to collect the biopsy samples: (1) the apex and base of the gland were identified, the prostate was then cut transversely (axial section) half way along the gland, (2) using the midline and the urethra as guides, biopsies were taken from the peripheral and transitional zones avoiding obvious tumour sites where feasible, (3) after collection of biopsies the prostate was sent for standard diagnostic histopathological examination. One tissue core from each zone was placed in RNAlater and stored at −80°C for downstream RNA sequencing. Two tissue cores from each zone were also placed in 80% HPLC-grade methanol/water for 24 hours for downstream metabolite profiling as per manufacturer’s guidelines (Metabolon Inc.). The methanol extracts were then frozen at –80 °C and tissue cores were subsequently sent for histological analysis. As the cores destined for metabolite profiling and RNA sequencing were taken from directly adjacent sites we used the histological evaluation on the metabolite profiling cores to determine whether samples were cancerous or not. The cores were confirmed during histological assessment to consist of a mixture of epithelial and stromal cells.

Clinical characteristics of patients are shown in Table 1.

### RNA sequencing and analysis

A total of 18 PZ and TZ tissues cores from nine patients were sent for RNA sequencing. Histology of the directly adjacent region later confirmed that 14 of these cores were of normal histology, and were used for analysis in the current study, and four of malignant histology. Prostate biopsy cores stored in RNAlater and weighing between 4 and 16 mg were homogenised with a QIAGEN TissueRuptor before total RNA was extracted with the QIAGEN RNeasy Mini kit. The resulting RNA was quality checked with an Agilent Bioanalyzer and samples with RIN values higher than 7 were further processed. Samples were subjected to ribodepletion with the Ribo-Zero Magnetic Gold rRNA Removal Kit (Illumina) before constructing Illumina barcoded TruSeq RNA libraries. Sequencing of 18 libraries, in pools of four, was performed over four and a half lanes of Illumina HiSeq 2500/2000 in High-Output mode using 100 bp paired-end reads. RNA-seq reads were first processed by removing Illumina adapters using Trim Galore! version 0.3.7 (Babraham Bioinformatics) and reads with Phred quality of basecalls higher than 20 and with a length of higher than 60 bp were carried forward. SortMeRNA version 1.9 [55] was used to filter any remaining ribosomal RNA from the adaptor and quality trimmed reads.

Reads were analysed using the *TopHat-Cufflinks* pipeline [56] aligned to the Ensembl GRCh37.75 reference genome. Only the 14 samples with non-cancerous histology were taken for further analysis. The *Cuffnorm* package was used to generate normalized expression levels for each sample. Genes with low expression (counts less than 100) were filtered out before performing unsupervised hierarchical clustering using Euclidean distance and an average linkage function to generate the hierarchical tree presented in Figure 1. We standardised expression values per gene across all samples and then used colour to visualise the variation in gene expression, where red denotes the highest and blue the lowest expression for that gene across all samples. The *Cuffdiff* package was also used for differential expression analysis of the PZ and TZ. Differentially expressed genes were identified as those that were different between the two zones at q<0.05 and the zone average FPKM values was higher than 1 in both zones. KEGG Pathways [57] that were over-represented were identified using Database for Annotation, Visualization and Integrated Discovery v6.8 (DAVID; http://david.abcc.ncifcrf.gov/) [58]. Significant pathways were identified according to their over-representation (ORA) scores obtained from DAVID. ORA scores are defined as the Benjamini – Hochberg adjusted modified Fisher’s exact test calculated from a 2x2 table test cross-classifying genes according to differential expression and pathway membership. RNA sequencing data have been deposited in ArrayExpress (E-MTAB-5021).

### Metabolite profiling and analysis

A total of 72 samples from eighteen men undergoing radical prostatectomy were sent to Metabolon Inc. (USA) to undergo ultrahigh performance liquid chromatography – mass spectroscopy (LC-MS) and gas chromatography – mass spectroscopy (GC-MS) with a high resolution accurate mass (HRAM) platform as previously described [59, 60]. These included two cores (one from each prostate lobe; left or right) taken from each zone (peripheral or transitional). Samples were sent in two batches. The first batch (*n* = 8 patients, 32 samples) returned 266 individual metabolites. The second batch (*n* = 10 patients, 40 samples) was processed after instrument upgrade (Metabolon^®^, USA) using the exact techniques and standardisation procedures as with the first batch and yielded 413 metabolites. There were 160 common metabolites, amongst which 150 were endogenous, between the two datasets that were taken forward for analysis. As we had two cores for each zone per patient we only further processed those cores with confirmed benign histology. In the case of both cores being benign the mean was calculated for subsequent multivariate analyses. Principal component analysis (PCA), partial least square-discriminate analysis (PLS-DA), and pathway analysis was carried out with the open-source web-based MetaboAnalyst software v3.0 (http://www.metaboanalyst.ca) [61, 62]. Data uploaded onto MetaboAnalyst were log_2_ transformed and autoscaled by dividing the metabolite values by the standard deviation. Over-representation (ORA) analysis and subsequent ORA scores were obtained from MetaboAnalyst by applying Fisher’s exact test.

Individual metabolite differences between the two zones were examined with univariate methods by analysis of variance (N-way ANOVA) in Matlab (The Mathworks, Cambridge, UK). For each metabolite the average of the zone (TZ, PZ) was used as the response variable and the log_10_ (to correct for departure from normality) of each measurement, the patient (18 factors), and the laterality of the core (left, right) as explanatory variables.

### Integration of transcriptomics and metabolomics data

Metabolomics and transcriptomics analyses were integrated with the aid of a human molecular interaction network. We used PathwayCommons version 8 (www.pathwaycommons.org; accessed 7th of July 2016) to collect interactions between genes, proteins and small molecules that correspond to biochemical pathways, gene regulation pathways and protein-protein interactions in human cells. The gene expression and metabolite concentrations data were then used to discover the extent of the connectivity of nodes (molecules) involved in differentiating the two prostatic zones. In effect, we find one connected component comprising 723 nodes (genes and metabolites) including 362 differentially expressed genes directly associated with metabolites also found as significantly differentiated. All network analyses and visualisations were performed in Cytoscape 3.1 [63]. We tested overrepresentation of Gene Ontology terms with Bingo plugin [64] in Cytoscape by applying hypergeometric test (alpha = 0.05) to genes directly associated with metabolites (Supplementary Figure 3 and Supplementary Table 2) and complete connected components of differentially expressed genes (Supplementary Table 3). Biological processes were selected as significant for *q* < 0.05 after applying correction for multiple testing with the Benjamini-Hochberg test.

## SUPPLEMENTARY MATERIALS FIGURES AND TABLES










