# Supplementary material for: Increased transcriptional and metabolic capacity for lipid metabolism in the peripheral zone of the prostate may underpin its increased susceptibility to cancer
Source: Oncotarget. 2017 May 17;8(49):84902–16. doi: 10.18632/oncotarget.17926 (PMC5689582; doi:10.18632/oncotarget.17926)
Supplement: Supplementary file 1 [file oncotarget-08-84902-s001.pdf]

Increased transcriptional and metabolic capacity for lipid metabolism in the peripheral zone of the prostate may underpin its increased susceptibility to cancer

Supplementary Materials

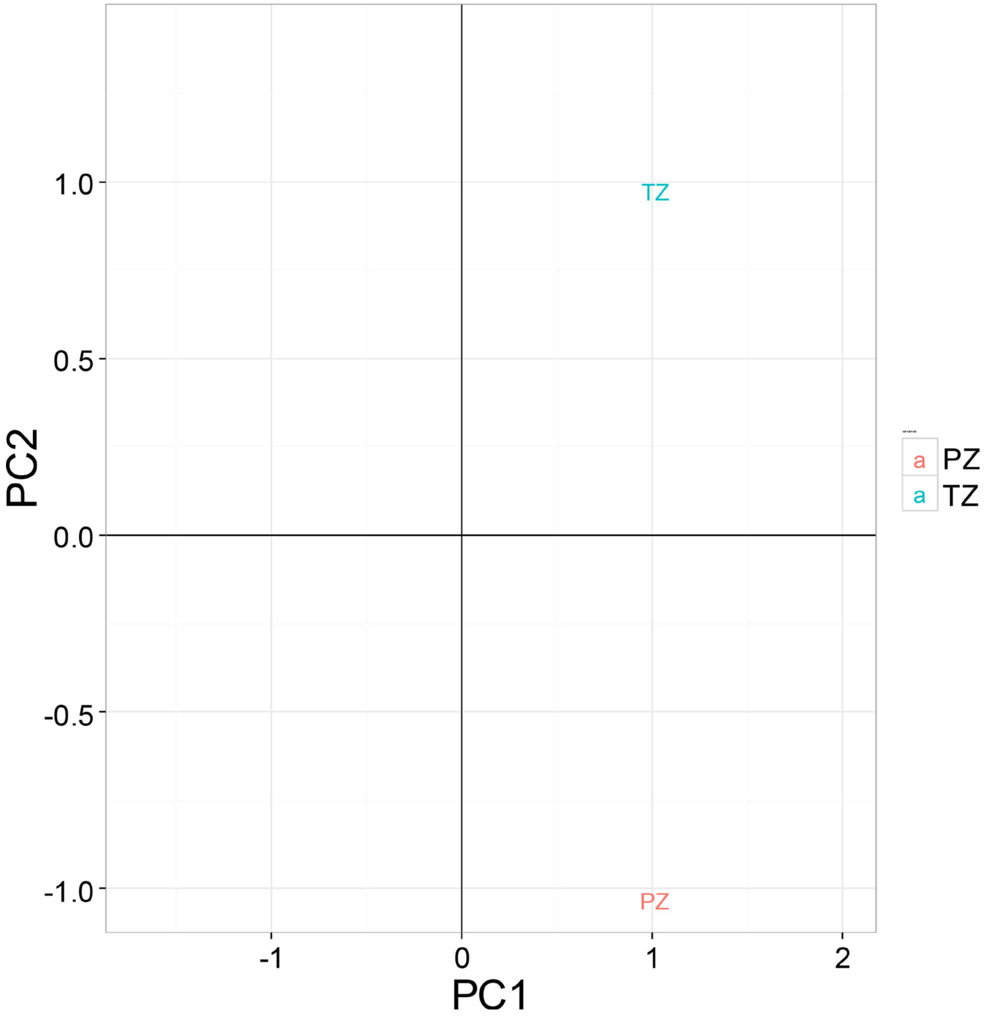

Supplementary Figure 1: Separation of the peripheral based on a previously reported zonal origin signature. PCA analysis separated accurately the two zones using the 11-gene signature previously identified by Sinnot *et al*.(2015).

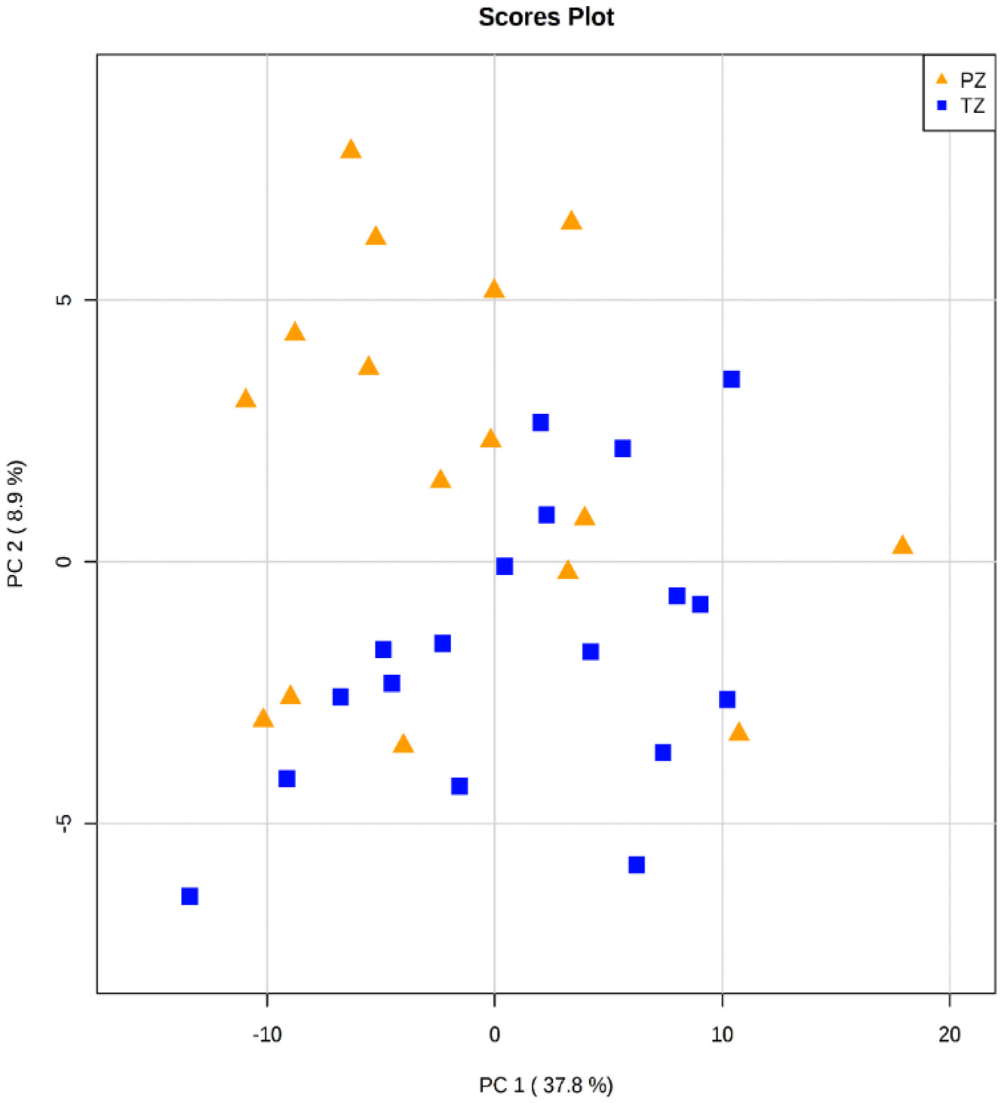

Supplementary Figure 2: Multivariate analysis of metabolomics data. (A) Principal component analysis (PCA) after calculating the average value of right and left sided samples and excluding cancer cores did not separate the prostate zones. PZ, peripheral zone (orange triangles); TZ, transitional zone (blue squares).

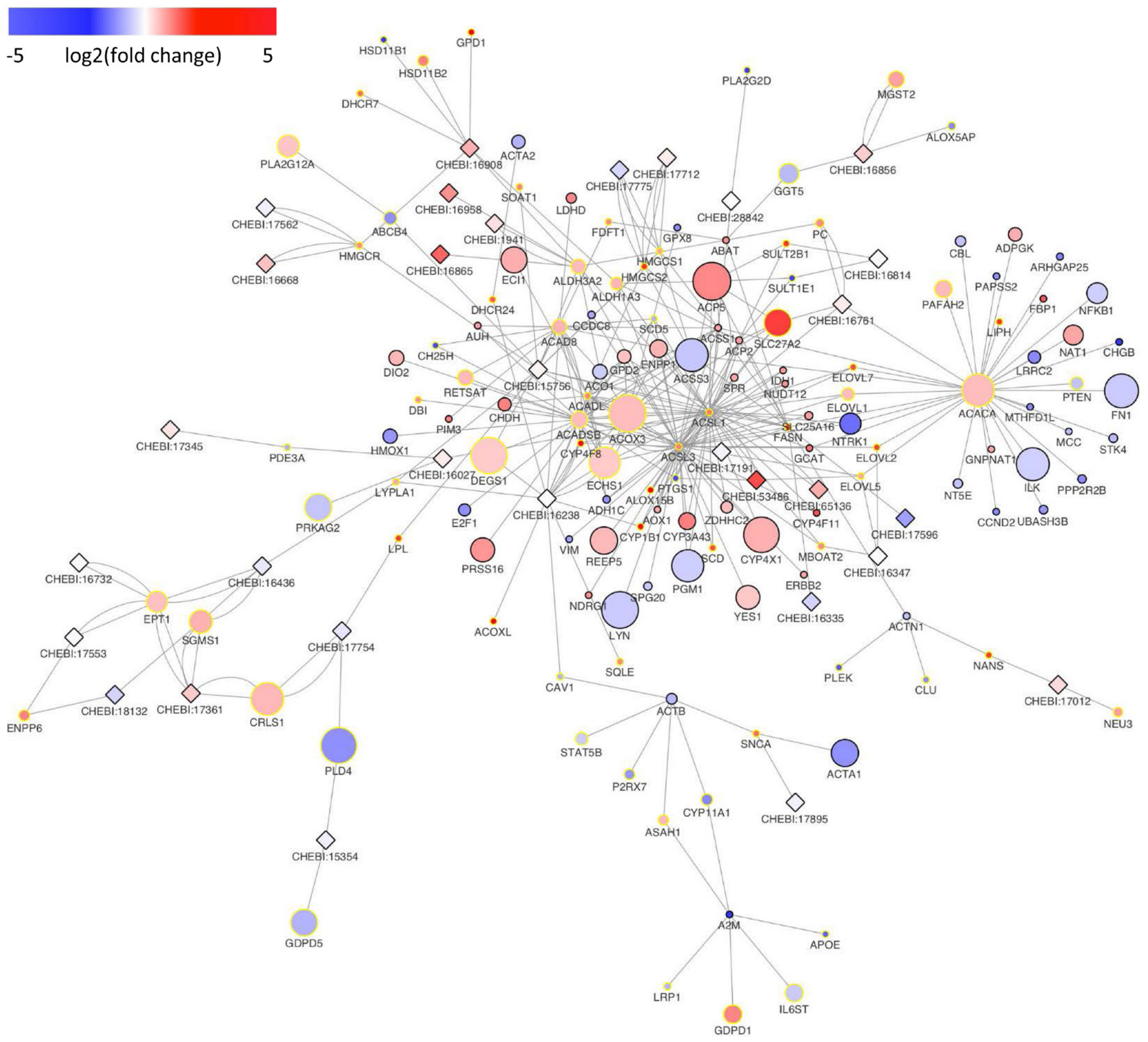

Supplementary Figure 3: Integrative analysis of RNAseqexpression and metabolomics data from the PZ and TZ.The graph depicts the network of genes and metabolites that were significantly different between the two zones. Metabolites are shown in diamond shape and are colored by $\log_2$(fold change): red –higher in PZ; blue –higher in TZ (from -5 to 5) , and size represents significance (small –high *p*-value; large –low *p*-value (more significant). The yellow border of genes (circles) denotes that the gene belongs to lipid metabolism GO term.

Supplementary Table 1: KEGG pathways that were upregulated in the transitional zone compared to the peripheral zone. See Supplementary_Table_1

Supplementary Table 2: Pathway over-representation analysis for each prostate zone against Metaboanalyst®reference metabolome. See Supplementary_Table_2

Supplementary Table 3: Overrepresentation of Gene Ontology terms within genes directly associated with metabolites. See Supplementary_Table_3

Supplementary Table 4: Overrepresentation of Gene Ontology terms within differentially expressed genes. See Supplementary_Table_4
