# Supplementary material for: Increased transcriptional and metabolic capacity for lipid metabolism in the peripheral zone of the prostate may underpin its increased susceptibility to cancer
Source: Oncotarget. 2017 May 17;8(49):84902–16. doi: 10.18632/oncotarget.17926 (PMC5689582; doi:10.18632/oncotarget.17926)
Supplement: Supplementary file 2 [file oncotarget-08-84902-s002.docx]

**Supplementary Table 1: KEGG pathways that were upregulated in the transitional zone compared to the peripheral zone.**

| **KEGG pathway** | **Genes^1^** | **Fold Enrichment** | **ORA score^2^** |
| --- | --- | --- | --- |
| Focal adhesion | 63 | 3.3 | 5.67E-16 |
| Staphylococcus aureus infection | 30 | 6.4 | 1.06E-15 |
| Cell adhesion molecules (CAMs) | 42 | 3.2 | 7.32E-10 |
| PI3K-Akt signaling pathway | 71 | 2.3 | 2.65E-09 |
| ECM-receptor interaction | 31 | 3.9 | 2.84E-09 |
| Chemokine signaling pathway | 46 | 2.7 | 1.96E-08 |
| HTLV-I infection | 56 | 2.4 | 2.48E-08 |
| Platelet activation | 36 | 3.1 | 4.90E-08 |
| Rap1 signaling pathway | 48 | 2.5 | 1.02E-07 |
| Proteoglycans in cancer | 45 | 2.5 | 3.73E-07 |
| Osteoclast differentiation | 34 | 2.9 | 7.25E-07 |
| Leishmaniasis | 24 | 3.7 | 7.70E-07 |
| Viral myocarditis | 21 | 4.0 | 1.14E-06 |
| Complement and coagulation cascades | 23 | 3.6 | 1.67E-06 |
| Pathways in cancer | 69 | 1.9 | 1.71E-06 |
| Leukocyte transendothelial migration | 31 | 2.9 | 2.22E-06 |
| Amoebiasis | 29 | 3.0 | 2.31E-06 |
| Cytokine-cytokine receptor interaction | 47 | 2.2 | 2.51E-06 |
| Phagosome | 36 | 2.6 | 2.91E-06 |
| Rheumatoid arthritis | 25 | 3.2 | 5.08E-06 |
| Regulation of actin cytoskeleton | 43 | 2.2 | 9.70E-06 |
| Dilated cardiomyopathy | 24 | 3.1 | 1.17E-05 |
| Protein digestion and absorption | 24 | 3.0 | 2.18E-05 |
| Ras signaling pathway | 43 | 2.1 | 5.08E-05 |
| Hematopoietic cell lineage | 23 | 3.0 | 5.11E-05 |
| Fc epsilon RI signaling pathway | 20 | 3.3 | 5.12E-05 |
| NF-kappa B signaling pathway | 23 | 2.9 | 6.86E-05 |
| Tuberculosis | 36 | 2.2 | 7.17E-05 |
| Intestinal immune network for IgA production | 16 | 3.7 | 9.11E-05 |
| Chagas disease (American trypanosomiasis) | 25 | 2.6 | 1.30E-04 |
| Toxoplasmosis | 27 | 2.5 | 1.35E-04 |
| Malaria | 16 | 3.6 | 1.46E-04 |
| cGMP-PKG signaling pathway | 33 | 2.2 | 1.53E-04 |
| Asthma | 12 | 4.5 | 1.93E-04 |
| Hypertrophic cardiomyopathy (HCM) | 20 | 2.8 | 3.89E-04 |
| TNF signaling pathway | 24 | 2.5 | 3.93E-04 |
| Graft-versus-host disease | 12 | 4.0 | 7.05E-04 |
| Fc gamma R-mediated phagocytosis | 20 | 2.6 | 0.001 |
| Axon guidance | 26 | 2.2 | 0.001 |
| Oxytocin signaling pathway | 30 | 2.1 | 0.001 |
| Type I diabetes mellitus | 13 | 3.4 | 0.002 |
| Allograft rejection | 12 | 3.5 | 0.002 |
| B cell receptor signaling pathway | 17 | 2.7 | 0.002 |
| Pertussis | 18 | 2.6 | 0.002 |
| Primary immunodeficiency | 11 | 3.6 | 0.003 |
| Inflammatory bowel disease (IBD) | 16 | 2.7 | 0.003 |
| Arrhythmogenic right ventricular cardiomyopathy (ARVC) | 17 | 2.6 | 0.003 |
| MAPK signaling pathway | 40 | 1.7 | 0.004 |
| T cell receptor signaling pathway | 20 | 2.1 | 0.010 |
| Systemic lupus erythematosus | 24 | 2.0 | 0.011 |
| Natural killer cell mediated cytotoxicity | 22 | 2.0 | 0.014 |
| Vascular smooth muscle contraction | 21 | 1.9 | 0.026 |
| Autoimmune thyroid disease | 12 | 2.6 | 0.026 |
| Long-term depression | 13 | 2.4 | 0.034 |
| Morphine addiction | 17 | 2.0 | 0.036 |
| Prion diseases | 9 | 3.0 | 0.036 |
| Antigen processing and presentation | 15 | 2.2 | 0.036 |
| FoxO signaling pathway | 22 | 1.8 | 0.037 |
| Insulin secretion | 16 | 2.1 | 0.040 |
| Small cell lung cancer | 16 | 2.1 | 0.040 |
| Viral carcinogenesis | 30 | 1.6 | 0.047 |
| Circadian entrainment | 17 | 2.0 | 0.048 |
| Sphingolipid signaling pathway | 20 | 1.8 | 0.049 |

^1^Input list was the 1512 genes expressed higher in the transitional zone (see Figure 1).

^2^Modified Fisher Exact P-Value, adjusted for multiple testing by the Benjamini–Hochberg method
