# Supplementary material for: Increased transcriptional and metabolic capacity for lipid metabolism in the peripheral zone of the prostate may underpin its increased susceptibility to cancer
Source: Oncotarget. 2017 May 17;8(49):84902–16. doi: 10.18632/oncotarget.17926 (PMC5689582; doi:10.18632/oncotarget.17926)
Supplement: Supplementary file 3 [file oncotarget-08-84902-s003.docx]

**Supplementary Table 2:** Pathway over-representation analysis for each prostate zone against Metaboanalyst® reference metabolome. All metabolites that were higher (on average) in each zone were uploaded to Metaboanalyst® platform.

|  | **Peripheral Zone** | | | **Transitional Zone** | | |
| --- | --- | --- | --- | --- | --- | --- |
| **Biochemical Pathway** | **ORA score^1^** | **Impact^2^** | **(-)log ORA score** | **ORA score^1^** | **Impact^2^** | **(-)log ORA score** |
| Alanine, aspartate and glutamate metabolism | 0.29621 | 0.36942 | 1.2167 | 0.99569 | 0.17664 | 0.0043223 |
| Amino sugar and nucleotide sugar metabolism | N/A | N/A | N/A | 0.26991 | 0 | 1.3097 |
| Aminoacyl-tRNA biosynthesis | 0.99786 | 0.11268 | 1.1334 | 0.35437 | 0.05634 | 1.0374 |
| Arginine and proline metabolism | 0.90859 | 0.2336 | 1.1334 | 0.94981 | 0.20012 | 0.051492 |
| Ascorbate and aldarate metabolism | N/A | N/A | N/A | 0.52222 | 0.00802 | 0.64966 |
| beta-Alanine metabolism | 0.8682 | 0.32319 | 0.85334 | 0.95734 | 0 | 0.043601 |
| Biotin metabolism | 0.68889 | 0 | 0.75044 | N/A | N/A | N/A |
| Butanoate metabolism | 0.92742 | 0.04612 | 0.75044 | 0.91754 | 0.02964 | 0.086055 |
| Caffeine metabolism | 0.68889 | 0.0305 | 0.75044 | N/A | N/A | N/A |
| Citrate cycle (TCA cycle) | 0.96888 | 0.0313 | 0.75044 | 0.54258 | 0.16514 | 0.61143 |
| Cyanoamino acid metabolism | 0.68889 | 0 | 0.45767 | N/A | N/A | N/A |
| Cysteine and methionine metabolism | 0.63275 | 0.01932 | 0.37268 | 0.9517 | 0.0478 | 0.049503 |
| D-Arginine and D-ornithine metabolism | 0.90562 | 0 | 0.37268 | N/A | N/A | N/A |
| D-Glutamine and D-glutamate metabolism | 0.90562 | 0.02674 | 0.37268 | 0.77453 | 0.1123 | 0.2555 |
| Ether lipid metabolism | 0.47216 | 0 | 0.37268 | N/A | N/A | N/A |
| Fructose and mannose metabolism | N/A | N/A | N/A | 0.77453 | 0.02948 | 0.2555 |
| Fatty acid biosynthesis | 0.47216 | 0 | 0.37268 | N/A | N/A | N/A |
| Fatty acid elongation in mitochondria | 0.68889 | 0 | 0.37268 | N/A | N/A | N/A |
| Fatty acid metabolism | 0.68889 | 0.02959 | 0.37268 | N/A | N/A | N/A |
| Galactose metabolism | N/A | N/A | N/A | 0.34268 | 0.00276 | 1.071 |
| Glutathione metabolism | 0.71114 | 0.33015 | 0.37268 | 0.98955 | 0.04551 | 0.010504 |
| Glycerolipid metabolism | N/A | N/A | N/A | 0.26991 | 0.20907 | 1.3097 |
| Glycerophospholipid metabolism | 0.99672 | 0.02626 | 0.37268 | 0.25561 | 0.25453 | 1.3641 |
| Glycine, serine and threonine metabolism | 0.98961 | 0.23265 | 0.37268 | 0.38187 | 0.00118 | 0.96266 |
| Glycolysis or Gluconeogenesis | N/A | N/A | N/A | 0.26991 | 0 | 1.3097 |
| Glyoxylate and dicarboxylate metabolism | 0.99776 | 0 | 0.34088 | 0.20941 | 0.08531 | 1.5634 |
| Lysine biosynthesis | 0.47216 | 0.16762 | 0.25796 | N/A | N/A | N/A |
| Lysine degradation | 0.32193 | 0.16793 | 0.25796 | N/A | N/A | N/A |
| Methane metabolism | 0.68889 | 0.01751 | 0.18883 | N/A | N/A | N/A |
| Histidine metabolism | N/A | N/A | N/A | 0.26991 | 0.14039 | 1.3097 |
| Nicotinate and nicotinamide metabolism | 0.90562 | 0 | 0.14134 | 0.77453 | 0.03827 | 0.2555 |
| Nitrogen metabolism | 0.99672 | 0 | 0.099138 | 0.25561 | 0 | 1.3641 |
| Pantothenate and CoA biosynthesis | 0.9125 | 0.07286 | 0.099138 | 0.9517 | 0 | 0.049503 |
| Pentose phosphate pathway | N/A | N/A | N/A | 0.13802 | 0.15737 | 1.9803 |
| Pentose and glucuronate interconversions | 0.68889 | 0 | 0.099138 | N/A | N/A | N/A |
| Phenylalanine metabolism | 0.82792 | 0 | 0.099138 | 0.84612 | 0.11906 | 0.16709 |
| Phenylalanine, tyrosine and tryptophan biosynthesis | N/A | N/A | N/A | 0.13802 | 0.008 | 1.9803 |
| Porphyrin and chlorophyll metabolism | 0.77263 | 0.03338 | 0.099138 | 0.89495 | 0 | 0.11098 |
| Primary bile acid biosynthesis | 0.68889 | 0.05524 | 0.095867 | N/A | N/A | N/A |
| Propanoate metabolism | 0.9125 | 0.08634 | 0.091571 | 0.7247 | 0 | 0.32199 |
| Purine metabolism | 0.97301 | 0.1817 | 0.091571 | 0.68184 | 0.05633 | 0.38295 |
| Pyrimidine metabolism | 0.42599 | 0.13537 | 0.091571 | 0.98955 | 0.02789 | 0.010504 |
| Riboflavin metabolism | 0.90562 | 0 | 0.075347 | 0.77453 | 0 | 0.2555 |
| Pyruvate metabolism | N/A | N/A | N/A | 0.26991 | 0.13756 | 1.3097 |
| Selenoamino acid metabolism | 0.68889 | 0 | 0.031615 | N/A | N/A | N/A |
| Sphingolipid metabolism | 0.9125 | 0.09061 | 0.028282 | 0.7247 | 0.02242 | 0.32199 |
| Starch and sucrose metabolism | N/A | N/A | N/A | 0.77453 | 0.01703 | 0.2555 |
| Steroid hormone biosynthesis | 0.32193 | 0.01589 | 0.027361 | 0.89495 | 0 | 0.11098 |
| Sulfur metabolism | 0.68889 | 0 | 0.010446 | N/A | N/A | N/A |
| Taurine and hypotaurine metabolism | 0.47216 | 0.11331 | 0.0080454 | N/A | N/A | N/A |
| Thiamine metabolism | N/A | N/A | N/A | 0.52222 | 0 | 0.64966 |
| Tryptophan metabolism | 0.97211 | 0.05734 | 0.0032902 | 0.53369 | 0.14349 | 0.62794 |
| Tyrosine metabolism | 0.77263 | 0 | 0.0032902 | 0.89495 | 0.04724 | 0.11098 |
| Ubiquinone and other terpenoid-quinone biosynthesis | 0.90562 | 0.00069 | 0.0022387 | 0.77453 | 0 | 0.2555 |
| Valine, leucine and isoleucine biosynthesis | 0.99199 | 0 | 0.0021449 | 0.34268 | 0.03975 | 1.071 |
| Valine, leucine and isoleucine degradation | N/A | N/A | N/A | 0.13802 | 0.02232 | 1.9803 |

^1^ Over-representation analysis (ORA) score (see Materials & Methods for details)

^2^ Impact calculated from pathway topology analysis
