# Supplementary material for: Increased transcriptional and metabolic capacity for lipid metabolism in the peripheral zone of the prostate may underpin its increased susceptibility to cancer
Source: Oncotarget. 2017 May 17;8(49):84902–16. doi: 10.18632/oncotarget.17926 (PMC5689582; doi:10.18632/oncotarget.17926)
Supplement: Supplementary file 4 [file oncotarget-08-84902-s004.docx]

**Supplementary Table 3: Overrepresentation of Gene Ontology terms within genes directly associated with metabolites.**

| GO-ID | p-value | corr p-value | Description |
| --- | --- | --- | --- |
| 44281 | 2.33E-49 | 5.72E-46 | small molecule metabolic process |
| 43436 | 5.46E-33 | 4.47E-30 | oxoacid metabolic process |
| 19752 | 5.46E-33 | 4.47E-30 | carboxylic acid metabolic process |
| 6082 | 1.15E-32 | 7.08E-30 | organic acid metabolic process |
| 42180 | 2.41E-32 | 1.18E-29 | cellular ketone metabolic process |
| 55114 | 2.18E-29 | 8.94E-27 | oxidation reduction |
| 8152 | 1.99E-28 | 6.97E-26 | metabolic process |
| 6519 | 9.26E-28 | 2.85E-25 | cellular amino acid and derivative metabolic process |
| 44283 | 6.04E-23 | 1.65E-20 | small molecule biosynthetic process |
| 44282 | 1.19E-22 | 2.94E-20 | small molecule catabolic process |
| 9308 | 5.19E-21 | 1.16E-18 | amine metabolic process |
| 44237 | 1.39E-20 | 2.84E-18 | cellular metabolic process |
| 44106 | 3.01E-20 | 5.69E-18 | cellular amine metabolic process |
| 6575 | 2.49E-19 | 4.38E-17 | cellular amino acid derivative metabolic process |
| 32787 | 1.13E-18 | 1.85E-16 | monocarboxylic acid metabolic process |
| 6629 | 6.87E-17 | 1.06E-14 | lipid metabolic process |
| 44238 | 9.14E-17 | 1.32E-14 | primary metabolic process |
| 6520 | 5.96E-16 | 8.14E-14 | cellular amino acid metabolic process |
| 46483 | 9.87E-15 | 1.28E-12 | heterocycle metabolic process |
| 46700 | 3.16E-14 | 3.89E-12 | heterocycle catabolic process |
| 9058 | 3.44E-14 | 4.03E-12 | biosynthetic process |
| 46394 | 6.13E-14 | 6.55E-12 | carboxylic acid biosynthetic process |
| 16053 | 6.13E-14 | 6.55E-12 | organic acid biosynthetic process |
| 46395 | 7.99E-14 | 7.86E-12 | carboxylic acid catabolic process |
| 16054 | 7.99E-14 | 7.86E-12 | organic acid catabolic process |
| 6732 | 1.70E-13 | 1.60E-11 | coenzyme metabolic process |
| 42221 | 2.13E-13 | 1.94E-11 | response to chemical stimulus |
| 44255 | 2.63E-13 | 2.31E-11 | cellular lipid metabolic process |
| 9056 | 7.94E-13 | 6.73E-11 | catabolic process |
| 55086 | 9.63E-13 | 7.90E-11 | nucleobase, nucleoside and nucleotide metabolic process |
| 51186 | 1.01E-12 | 8.01E-11 | cofactor metabolic process |
| 44248 | 1.38E-12 | 1.06E-10 | cellular catabolic process |
| 44270 | 1.59E-12 | 1.17E-10 | cellular nitrogen compound catabolic process |
| 10033 | 1.62E-12 | 1.17E-10 | response to organic substance |
| 9117 | 4.79E-12 | 3.26E-10 | nucleotide metabolic process |
| 6753 | 4.79E-12 | 3.26E-10 | nucleoside phosphate metabolic process |
| 6865 | 4.90E-12 | 3.26E-10 | amino acid transport |
| 44271 | 5.51E-12 | 3.56E-10 | cellular nitrogen compound biosynthetic process |
| 6631 | 1.24E-11 | 7.79E-10 | fatty acid metabolic process |
| 46942 | 1.78E-11 | 1.09E-09 | carboxylic acid transport |
| 34655 | 1.87E-11 | 1.09E-09 | nucleobase, nucleoside, nucleotide and nucleic acid catabolic process |
| 34656 | 1.87E-11 | 1.09E-09 | nucleobase, nucleoside and nucleotide catabolic process |
| 15849 | 2.02E-11 | 1.16E-09 | organic acid transport |
| 44249 | 2.11E-11 | 1.18E-09 | cellular biosynthetic process |
| 9310 | 2.45E-11 | 1.34E-09 | amine catabolic process |
| 6749 | 3.48E-11 | 1.86E-09 | glutathione metabolic process |
| 6066 | 5.24E-11 | 2.74E-09 | alcohol metabolic process |
| 9309 | 7.26E-11 | 3.72E-09 | amine biosynthetic process |
| 6807 | 7.73E-11 | 3.87E-09 | nitrogen compound metabolic process |
| 42398 | 7.87E-11 | 3.87E-09 | cellular amino acid derivative biosynthetic process |
| 8610 | 8.10E-11 | 3.90E-09 | lipid biosynthetic process |
| 15837 | 3.26E-10 | 1.54E-08 | amine transport |
| 9719 | 3.44E-10 | 1.60E-08 | response to endogenous stimulus |
| 6576 | 3.68E-10 | 1.67E-08 | cellular biogenic amine metabolic process |
| 9725 | 3.73E-10 | 1.67E-08 | response to hormone stimulus |
| 9166 | 5.77E-10 | 2.54E-08 | nucleotide catabolic process |
| 9063 | 6.10E-10 | 2.63E-08 | cellular amino acid catabolic process |
| 9125 | 6.28E-10 | 2.66E-08 | nucleoside monophosphate catabolic process |
| 6725 | 2.16E-09 | 8.98E-08 | cellular aromatic compound metabolic process |
| 9214 | 3.68E-09 | 1.51E-07 | cyclic nucleotide catabolic process |
| 34641 | 6.98E-09 | 2.81E-07 | cellular nitrogen compound metabolic process |
| 9123 | 1.26E-08 | 4.98E-07 | nucleoside monophosphate metabolic process |
| 6790 | 1.55E-08 | 6.06E-07 | sulfur metabolic process |
| 6195 | 1.86E-08 | 7.13E-07 | purine nucleotide catabolic process |
| 42493 | 2.11E-08 | 7.99E-07 | response to drug |
| 6163 | 3.23E-08 | 1.20E-06 | purine nucleotide metabolic process |
| 6198 | 4.99E-08 | 1.83E-06 | cAMP catabolic process |
| 6518 | 7.53E-08 | 2.72E-06 | peptide metabolic process |
| 6633 | 8.07E-08 | 2.88E-06 | fatty acid biosynthetic process |
| 16126 | 1.89E-07 | 6.64E-06 | sterol biosynthetic process |
| 9987 | 2.31E-07 | 8.00E-06 | cellular process |
| 42401 | 2.41E-07 | 8.23E-06 | cellular biogenic amine biosynthetic process |
| 5975 | 2.55E-07 | 8.58E-06 | carbohydrate metabolic process |
| 45907 | 3.14E-07 | 1.03E-05 | positive regulation of vasoconstriction |
| 46058 | 3.14E-07 | 1.03E-05 | cAMP metabolic process |
| 48545 | 3.23E-07 | 1.04E-05 | response to steroid hormone stimulus |
| 6091 | 6.03E-07 | 1.93E-05 | generation of precursor metabolites and energy |
| 31960 | 6.73E-07 | 2.12E-05 | response to corticosteroid stimulus |
| 10035 | 7.38E-07 | 2.30E-05 | response to inorganic substance |
| 9187 | 1.20E-06 | 3.69E-05 | cyclic nucleotide metabolic process |
| 6081 | 1.53E-06 | 4.63E-05 | cellular aldehyde metabolic process |
| 9074 | 1.74E-06 | 5.22E-05 | aromatic amino acid family catabolic process |
| 42219 | 1.96E-06 | 5.81E-05 | cellular amino acid derivative catabolic process |
| 9108 | 3.73E-06 | 1.09E-04 | coenzyme biosynthetic process |
| 44262 | 3.96E-06 | 1.15E-04 | cellular carbohydrate metabolic process |
| 19229 | 4.47E-06 | 1.28E-04 | regulation of vasoconstriction |
| 6694 | 4.87E-06 | 1.38E-04 | steroid biosynthetic process |
| 8652 | 5.00E-06 | 1.40E-04 | cellular amino acid biosynthetic process |
| 6695 | 6.00E-06 | 1.66E-04 | cholesterol biosynthetic process |
| 42402 | 6.30E-06 | 1.70E-04 | cellular biogenic amine catabolic process |
| 15804 | 6.30E-06 | 1.70E-04 | neutral amino acid transport |
| 46069 | 6.90E-06 | 1.84E-04 | cGMP catabolic process |
| 3013 | 9.95E-06 | 2.60E-04 | circulatory system process |
| 8015 | 9.95E-06 | 2.60E-04 | blood circulation |
| 70482 | 1.06E-05 | 2.76E-04 | response to oxygen levels |
| 8202 | 1.12E-05 | 2.87E-04 | steroid metabolic process |
| 6979 | 1.20E-05 | 3.04E-04 | response to oxidative stress |
| 8277 | 1.53E-05 | 3.83E-04 | regulation of G-protein coupled receptor protein signaling pathway |
| 51384 | 1.60E-05 | 3.97E-04 | response to glucocorticoid stimulus |
| 9072 | 2.29E-05 | 5.63E-04 | aromatic amino acid family metabolic process |
| 44242 | 2.84E-05 | 6.91E-04 | cellular lipid catabolic process |
| 44057 | 3.33E-05 | 8.04E-04 | regulation of system process |
| 9070 | 3.87E-05 | 9.09E-04 | serine family amino acid biosynthetic process |
| 6750 | 3.87E-05 | 9.09E-04 | glutathione biosynthetic process |
| 19439 | 3.88E-05 | 9.09E-04 | aromatic compound catabolic process |
| 51385 | 4.95E-05 | 1.15E-03 | response to mineralocorticoid stimulus |
| 34404 | 5.35E-05 | 1.22E-03 | nucleobase, nucleoside and nucleotide biosynthetic process |
| 34654 | 5.35E-05 | 1.22E-03 | nucleobase, nucleoside, nucleotide and nucleic acid biosynthetic process |
| 42423 | 5.71E-05 | 1.29E-03 | catecholamine biosynthetic process |
| 50896 | 6.66E-05 | 1.49E-03 | response to stimulus |
| 8217 | 9.78E-05 | 2.17E-03 | regulation of blood pressure |
| 6767 | 1.02E-04 | 2.25E-03 | water-soluble vitamin metabolic process |
| 51188 | 1.15E-04 | 2.48E-03 | cofactor biosynthetic process |
| 9069 | 1.16E-04 | 2.48E-03 | serine family amino acid metabolic process |
| 19400 | 1.16E-04 | 2.48E-03 | alditol metabolic process |
| 9062 | 1.18E-04 | 2.50E-03 | fatty acid catabolic process |
| 16125 | 1.24E-04 | 2.60E-03 | sterol metabolic process |
| 19217 | 1.26E-04 | 2.62E-03 | regulation of fatty acid metabolic process |
| 46218 | 1.32E-04 | 2.66E-03 | indolalkylamine catabolic process |
| 42436 | 1.32E-04 | 2.66E-03 | indole derivative catabolic process |
| 6534 | 1.32E-04 | 2.66E-03 | cysteine metabolic process |
| 6569 | 1.32E-04 | 2.66E-03 | tryptophan catabolic process |
| 43648 | 1.35E-04 | 2.67E-03 | dicarboxylic acid metabolic process |
| 1666 | 1.36E-04 | 2.67E-03 | response to hypoxia |
| 14070 | 1.36E-04 | 2.67E-03 | response to organic cyclic substance |
| 6144 | 1.51E-04 | 2.94E-03 | purine base metabolic process |
| 9165 | 1.53E-04 | 2.96E-03 | nucleotide biosynthetic process |
| 10038 | 1.72E-04 | 3.30E-03 | response to metal ion |
| 16042 | 1.89E-04 | 3.59E-03 | lipid catabolic process |
| 7200 | 2.27E-04 | 4.18E-03 | activation of phospholipase C activity by G-protein coupled receptor protein signaling pathway coupled to IP3 second messenger |
| 46146 | 2.28E-04 | 4.18E-03 | tetrahydrobiopterin metabolic process |
| 6002 | 2.28E-04 | 4.18E-03 | fructose 6-phosphate metabolic process |
| 6072 | 2.28E-04 | 4.18E-03 | glycerol-3-phosphate metabolic process |
| 6568 | 2.28E-04 | 4.18E-03 | tryptophan metabolic process |
| 9636 | 2.32E-04 | 4.23E-03 | response to toxin |
| 6084 | 2.36E-04 | 4.27E-03 | acetyl-CoA metabolic process |
| 55085 | 2.43E-04 | 4.36E-03 | transmembrane transport |
| 6000 | 2.55E-04 | 4.51E-03 | fructose metabolic process |
| 60324 | 2.55E-04 | 4.51E-03 | face development |
| 51240 | 3.01E-04 | 5.29E-03 | positive regulation of multicellular organismal process |
| 51412 | 3.23E-04 | 5.63E-03 | response to corticosterone stimulus |
| 8203 | 3.46E-04 | 5.88E-03 | cholesterol metabolic process |
| 6596 | 3.60E-04 | 5.88E-03 | polyamine biosynthetic process |
| 8215 | 3.64E-04 | 5.88E-03 | spermine metabolic process |
| 70813 | 3.64E-04 | 5.88E-03 | hydrogen sulfide metabolic process |
| 70814 | 3.64E-04 | 5.88E-03 | hydrogen sulfide biosynthetic process |
| 10040 | 3.64E-04 | 5.88E-03 | response to iron(II) ion |
| 6145 | 3.64E-04 | 5.88E-03 | purine base catabolic process |
| 6597 | 3.64E-04 | 5.88E-03 | spermine biosynthetic process |
| 35408 | 3.64E-04 | 5.88E-03 | histone H3-T6 phosphorylation |
| 19344 | 3.64E-04 | 5.88E-03 | cysteine biosynthetic process |
| 19459 | 3.64E-04 | 5.88E-03 | glutamate deamidation |
| 43043 | 4.03E-04 | 6.39E-03 | peptide biosynthetic process |
| 9064 | 4.03E-04 | 6.39E-03 | glutamine family amino acid metabolic process |
| 10517 | 4.03E-04 | 6.39E-03 | regulation of phospholipase activity |
| 42592 | 4.08E-04 | 6.42E-03 | homeostatic process |
| 9259 | 4.21E-04 | 6.60E-03 | ribonucleotide metabolic process |
| 9712 | 4.33E-04 | 6.66E-03 | catechol metabolic process |
| 34311 | 4.33E-04 | 6.66E-03 | diol metabolic process |
| 6584 | 4.33E-04 | 6.66E-03 | catecholamine metabolic process |
| 65008 | 4.40E-04 | 6.72E-03 | regulation of biological quality |
| 8299 | 4.96E-04 | 7.42E-03 | isoprenoid biosynthetic process |
| 33559 | 4.97E-04 | 7.42E-03 | unsaturated fatty acid metabolic process |
| 44272 | 4.97E-04 | 7.42E-03 | sulfur compound biosynthetic process |
| 18958 | 4.98E-04 | 7.42E-03 | phenol metabolic process |
| 10648 | 5.65E-04 | 8.36E-03 | negative regulation of cell communication |
| 6636 | 5.69E-04 | 8.38E-03 | unsaturated fatty acid biosynthetic process |
| 43279 | 6.08E-04 | 8.90E-03 | response to alkaloid |
| 19725 | 6.46E-04 | 9.40E-03 | cellular homeostasis |
| 10243 | 6.61E-04 | 9.56E-03 | response to organic nitrogen |
| 19637 | 6.67E-04 | 9.59E-03 | organophosphate metabolic process |
| 46496 | 7.35E-04 | 1.04E-02 | nicotinamide nucleotide metabolic process |
| 51952 | 7.35E-04 | 1.04E-02 | regulation of amine transport |
| 6595 | 7.49E-04 | 1.06E-02 | polyamine metabolic process |
| 51592 | 8.10E-04 | 1.14E-02 | response to calcium ion |
| 34440 | 8.30E-04 | 1.15E-02 | lipid oxidation |
| 19395 | 8.30E-04 | 1.15E-02 | fatty acid oxidation |
| 9112 | 8.66E-04 | 1.18E-02 | nucleobase metabolic process |
| 60322 | 8.66E-04 | 1.18E-02 | head development |
| 19318 | 8.67E-04 | 1.18E-02 | hexose metabolic process |
| 10565 | 8.96E-04 | 1.21E-02 | regulation of cellular ketone metabolic process |
| 6766 | 8.96E-04 | 1.21E-02 | vitamin metabolic process |
| 43066 | 9.27E-04 | 1.24E-02 | negative regulation of apoptosis |
| 5996 | 9.29E-04 | 1.24E-02 | monosaccharide metabolic process |
| 9150 | 9.74E-04 | 1.29E-02 | purine ribonucleotide metabolic process |
| 31294 | 1.02E-03 | 1.29E-02 | lymphocyte costimulation |
| 31295 | 1.02E-03 | 1.29E-02 | T cell costimulation |
| 60325 | 1.02E-03 | 1.29E-02 | face morphogenesis |
| 96 | 1.02E-03 | 1.29E-02 | sulfur amino acid metabolic process |
| 42558 | 1.02E-03 | 1.29E-02 | pteridine and derivative metabolic process |
| 50805 | 1.02E-03 | 1.29E-02 | negative regulation of synaptic transmission |
| 6071 | 1.02E-03 | 1.29E-02 | glycerol metabolic process |
| 32844 | 1.03E-03 | 1.29E-02 | regulation of homeostatic process |
| 6836 | 1.04E-03 | 1.29E-02 | neurotransmitter transport |
| 19362 | 1.05E-03 | 1.29E-02 | pyridine nucleotide metabolic process |
| 8643 | 1.06E-03 | 1.29E-02 | carbohydrate transport |
| 51051 | 1.06E-03 | 1.29E-02 | negative regulation of transport |
| 43069 | 1.07E-03 | 1.29E-02 | negative regulation of programmed cell death |
| 50667 | 1.08E-03 | 1.29E-02 | homocysteine metabolic process |
| 18126 | 1.08E-03 | 1.29E-02 | protein amino acid hydroxylation |
| 35405 | 1.08E-03 | 1.29E-02 | histone-threonine phosphorylation |
| 2676 | 1.08E-03 | 1.29E-02 | regulation of chronic inflammatory response |
| 2678 | 1.08E-03 | 1.29E-02 | positive regulation of chronic inflammatory response |
| 19405 | 1.08E-03 | 1.29E-02 | alditol catabolic process |
| 7207 | 1.08E-03 | 1.29E-02 | activation of phospholipase C activity by muscarinic acetylcholine receptor signaling pathway |
| 90257 | 1.11E-03 | 1.33E-02 | regulation of muscle system process |
| 50865 | 1.12E-03 | 1.34E-02 | regulation of cell activation |
| 38 | 1.20E-03 | 1.42E-02 | very long-chain fatty acid metabolic process |
| 10863 | 1.26E-03 | 1.47E-02 | positive regulation of phospholipase C activity |
| 7202 | 1.26E-03 | 1.47E-02 | activation of phospholipase C activity |
| 302 | 1.28E-03 | 1.49E-02 | response to reactive oxygen species |
| 7268 | 1.31E-03 | 1.52E-02 | synaptic transmission |
| 6164 | 1.33E-03 | 1.53E-02 | purine nucleotide biosynthetic process |
| 46068 | 1.33E-03 | 1.53E-02 | cGMP metabolic process |
| 51953 | 1.33E-03 | 1.53E-02 | negative regulation of amine transport |
| 60548 | 1.34E-03 | 1.53E-02 | negative regulation of cell death |
| 9161 | 1.40E-03 | 1.58E-02 | ribonucleoside monophosphate metabolic process |
| 19751 | 1.45E-03 | 1.63E-02 | polyol metabolic process |
| 7611 | 1.45E-03 | 1.63E-02 | learning or memory |
| 7584 | 1.46E-03 | 1.64E-02 | response to nutrient |
| 30258 | 1.48E-03 | 1.65E-02 | lipid modification |
| 45454 | 1.60E-03 | 1.77E-02 | cell redox homeostasis |
| 1570 | 1.60E-03 | 1.77E-02 | vasculogenesis |
| 50433 | 1.62E-03 | 1.77E-02 | regulation of catecholamine secretion |
| 6090 | 1.62E-03 | 1.77E-02 | pyruvate metabolic process |
| 22900 | 1.63E-03 | 1.77E-02 | electron transport chain |
| 60191 | 1.66E-03 | 1.80E-02 | regulation of lipase activity |
| 42430 | 1.71E-03 | 1.83E-02 | indole and derivative metabolic process |
| 42434 | 1.71E-03 | 1.83E-02 | indole derivative metabolic process |
| 6586 | 1.71E-03 | 1.83E-02 | indolalkylamine metabolic process |
| 9991 | 1.73E-03 | 1.83E-02 | response to extracellular stimulus |
| 10518 | 1.73E-03 | 1.83E-02 | positive regulation of phospholipase activity |
| 70887 | 1.76E-03 | 1.86E-02 | cellular response to chemical stimulus |
| 51970 | 1.86E-03 | 1.94E-02 | negative regulation of transmission of nerve impulse |
| 19319 | 1.86E-03 | 1.94E-02 | hexose biosynthetic process |
| 15749 | 1.86E-03 | 1.94E-02 | monosaccharide transport |
| 6006 | 1.97E-03 | 2.04E-02 | glucose metabolic process |
| 6635 | 2.12E-03 | 2.12E-02 | fatty acid beta-oxidation |
| 7595 | 2.12E-03 | 2.12E-02 | lactation |
| 33632 | 2.13E-03 | 2.12E-02 | regulation of cell-cell adhesion mediated by integrin |
| 46113 | 2.13E-03 | 2.12E-02 | nucleobase catabolic process |
| 46149 | 2.13E-03 | 2.12E-02 | pigment catabolic process |
| 42167 | 2.13E-03 | 2.12E-02 | heme catabolic process |
| 14805 | 2.13E-03 | 2.12E-02 | smooth muscle adaptation |
| 19695 | 2.13E-03 | 2.12E-02 | choline metabolic process |
| 19852 | 2.13E-03 | 2.12E-02 | L-ascorbic acid metabolic process |
| 9968 | 2.13E-03 | 2.12E-02 | negative regulation of signal transduction |
| 10039 | 2.15E-03 | 2.12E-02 | response to iron ion |
| 6835 | 2.15E-03 | 2.12E-02 | dicarboxylic acid transport |
| 51048 | 2.17E-03 | 2.13E-02 | negative regulation of secretion |
| 50867 | 2.24E-03 | 2.20E-02 | positive regulation of cell activation |
| 2694 | 2.34E-03 | 2.28E-02 | regulation of leukocyte activation |
| 51046 | 2.35E-03 | 2.28E-02 | regulation of secretion |
| 9119 | 2.35E-03 | 2.28E-02 | ribonucleoside metabolic process |
| 23057 | 2.36E-03 | 2.28E-02 | negative regulation of signaling process |
| 6690 | 2.58E-03 | 2.47E-02 | icosanoid metabolic process |
| 60323 | 2.65E-03 | 2.53E-02 | head morphogenesis |
| 31645 | 2.73E-03 | 2.60E-02 | negative regulation of neurological system process |
| 6733 | 2.81E-03 | 2.67E-02 | oxidoreduction coenzyme metabolic process |
| 6937 | 2.88E-03 | 2.71E-02 | regulation of muscle contraction |
| 60193 | 2.88E-03 | 2.71E-02 | positive regulation of lipase activity |
| 9260 | 3.03E-03 | 2.84E-02 | ribonucleotide biosynthetic process |
| 9755 | 3.06E-03 | 2.85E-02 | hormone-mediated signaling pathway |
| 7588 | 3.06E-03 | 2.85E-02 | excretion |
| 9311 | 3.07E-03 | 2.85E-02 | oligosaccharide metabolic process |
| 34341 | 3.21E-03 | 2.96E-02 | response to interferon-gamma |
| 6536 | 3.21E-03 | 2.96E-02 | glutamate metabolic process |
| 50804 | 3.33E-03 | 3.06E-02 | regulation of synaptic transmission |
| 45768 | 3.45E-03 | 3.08E-02 | positive regulation of anti-apoptosis |
| 46456 | 3.45E-03 | 3.08E-02 | icosanoid biosynthetic process |
| 51187 | 3.45E-03 | 3.08E-02 | cofactor catabolic process |
| 45920 | 3.50E-03 | 3.08E-02 | negative regulation of exocytosis |
| 46174 | 3.50E-03 | 3.08E-02 | polyol catabolic process |
| 6537 | 3.50E-03 | 3.08E-02 | glutamate biosynthetic process |
| 43589 | 3.50E-03 | 3.08E-02 | skin morphogenesis |
| 2664 | 3.50E-03 | 3.08E-02 | regulation of T cell tolerance induction |
| 2666 | 3.50E-03 | 3.08E-02 | positive regulation of T cell tolerance induction |
| 60291 | 3.50E-03 | 3.08E-02 | long-term synaptic potentiation |
| 19441 | 3.50E-03 | 3.08E-02 | tryptophan catabolic process to kynurenine |
| 51234 | 3.53E-03 | 3.10E-02 | establishment of localization |
| 7610 | 3.74E-03 | 3.28E-02 | behavior |
| 51260 | 3.78E-03 | 3.30E-02 | protein homooligomerization |
| 19438 | 3.84E-03 | 3.31E-02 | aromatic compound biosynthetic process |
| 19915 | 3.84E-03 | 3.31E-02 | lipid storage |
| 46364 | 3.85E-03 | 3.31E-02 | monosaccharide biosynthetic process |
| 10827 | 3.85E-03 | 3.31E-02 | regulation of glucose transport |
| 6810 | 3.97E-03 | 3.40E-02 | transport |
| 51251 | 3.99E-03 | 3.40E-02 | positive regulation of lymphocyte activation |
| 19216 | 4.02E-03 | 3.42E-02 | regulation of lipid metabolic process |
| 10646 | 4.22E-03 | 3.57E-02 | regulation of cell communication |
| 6950 | 4.75E-03 | 4.01E-02 | response to stress |
| 8016 | 4.79E-03 | 4.03E-02 | regulation of heart contraction |
| 6720 | 4.92E-03 | 4.07E-02 | isoprenoid metabolic process |
| 19932 | 4.93E-03 | 4.07E-02 | second-messenger-mediated signaling |
| 1568 | 5.04E-03 | 4.07E-02 | blood vessel development |
| 2684 | 5.04E-03 | 4.07E-02 | positive regulation of immune system process |
| 33015 | 5.18E-03 | 4.07E-02 | tetrapyrrole catabolic process |
| 9068 | 5.18E-03 | 4.07E-02 | aspartate family amino acid catabolic process |
| 9437 | 5.18E-03 | 4.07E-02 | carnitine metabolic process |
| 46325 | 5.18E-03 | 4.07E-02 | negative regulation of glucose import |
| 46479 | 5.18E-03 | 4.07E-02 | glycosphingolipid catabolic process |
| 46487 | 5.18E-03 | 4.07E-02 | glyoxylate metabolic process |
| 71453 | 5.18E-03 | 4.07E-02 | cellular response to oxygen levels |
| 71456 | 5.18E-03 | 4.07E-02 | cellular response to hypoxia |
| 6538 | 5.18E-03 | 4.07E-02 | glutamate catabolic process |
| 6558 | 5.18E-03 | 4.07E-02 | L-phenylalanine metabolic process |
| 6559 | 5.18E-03 | 4.07E-02 | L-phenylalanine catabolic process |
| 6577 | 5.18E-03 | 4.07E-02 | betaine metabolic process |
| 6729 | 5.18E-03 | 4.07E-02 | tetrahydrobiopterin biosynthetic process |
| 6787 | 5.18E-03 | 4.07E-02 | porphyrin catabolic process |
| 7210 | 5.18E-03 | 4.07E-02 | serotonin receptor signaling pathway |
| 51969 | 5.24E-03 | 4.07E-02 | regulation of transmission of nerve impulse |
| 51179 | 5.25E-03 | 4.07E-02 | localization |
| 15992 | 5.30E-03 | 4.07E-02 | proton transport |
| 9065 | 5.33E-03 | 4.07E-02 | glutamine family amino acid catabolic process |
| 9126 | 5.33E-03 | 4.07E-02 | purine nucleoside monophosphate metabolic process |
| 9167 | 5.33E-03 | 4.07E-02 | purine ribonucleoside monophosphate metabolic process |
| 42304 | 5.33E-03 | 4.07E-02 | regulation of fatty acid biosynthetic process |
| 10171 | 5.33E-03 | 4.07E-02 | body morphogenesis |
| 2026 | 5.33E-03 | 4.07E-02 | regulation of the force of heart contraction |
| 43450 | 5.33E-03 | 4.07E-02 | alkene biosynthetic process |
| 19370 | 5.33E-03 | 4.07E-02 | leukotriene biosynthetic process |
| 48015 | 5.39E-03 | 4.10E-02 | phosphoinositide-mediated signaling |
| 9612 | 5.69E-03 | 4.32E-02 | response to mechanical stimulus |
| 6940 | 5.77E-03 | 4.37E-02 | regulation of smooth muscle contraction |
| 16051 | 5.95E-03 | 4.49E-02 | carbohydrate biosynthetic process |
| 6818 | 6.10E-03 | 4.58E-02 | hydrogen transport |
| 42417 | 6.18E-03 | 4.58E-02 | dopamine metabolic process |
| 18107 | 6.18E-03 | 4.58E-02 | peptidyl-threonine phosphorylation |
| 35094 | 6.18E-03 | 4.58E-02 | response to nicotine |
| 48169 | 6.18E-03 | 4.58E-02 | regulation of long-term neuronal synaptic plasticity |
| 19226 | 6.18E-03 | 4.58E-02 | transmission of nerve impulse |
| 43627 | 6.24E-03 | 4.61E-02 | response to estrogen stimulus |
| 51241 | 6.29E-03 | 4.63E-02 | negative regulation of multicellular organismal process |
| 1944 | 6.35E-03 | 4.65E-02 | vasculature development |
| 48878 | 6.36E-03 | 4.65E-02 | chemical homeostasis |
| 15980 | 6.72E-03 | 4.90E-02 | energy derivation by oxidation of organic compounds |
| 2696 | 6.85E-03 | 4.98E-02 | positive regulation of leukocyte activation |
| 45767 | 6.94E-03 | 4.99E-02 | regulation of anti-apoptosis |
| 9124 | 6.94E-03 | 4.99E-02 | nucleoside monophosphate biosynthetic process |
| 9116 | 6.99E-03 | 4.99E-02 | nucleoside metabolic process |
| 188 | 7.11E-03 | 4.99E-02 | inactivation of MAPK activity |
| 42133 | 7.11E-03 | 4.99E-02 | neurotransmitter metabolic process |
| 2053 | 7.11E-03 | 4.99E-02 | positive regulation of mesenchymal cell proliferation |
| 2062 | 7.11E-03 | 4.99E-02 | chondrocyte differentiation |
| 31667 | 7.14E-03 | 4.99E-02 | response to nutrient levels |
| 16572 | 7.17E-03 | 4.99E-02 | histone phosphorylation |
| 45717 | 7.17E-03 | 4.99E-02 | negative regulation of fatty acid biosynthetic process |
| 42416 | 7.17E-03 | 4.99E-02 | dopamine biosynthetic process |
| 10269 | 7.17E-03 | 4.99E-02 | response to selenium ion |
| 10829 | 7.17E-03 | 4.99E-02 | negative regulation of glucose transport |
| 2643 | 7.17E-03 | 4.99E-02 | regulation of tolerance induction |
| 2645 | 7.17E-03 | 4.99E-02 | positive regulation of tolerance induction |
| 9152 | 7.50E-03 | 5.20E-02 | purine ribonucleotide biosynthetic process |
| 50727 | 7.51E-03 | 5.20E-02 | regulation of inflammatory response |
| 23051 | 7.55E-03 | 5.21E-02 | regulation of signaling process |
| 50729 | 7.57E-03 | 5.22E-02 | positive regulation of inflammatory response |
| 6954 | 7.67E-03 | 5.27E-02 | inflammatory response |
| 31644 | 7.87E-03 | 5.39E-02 | regulation of neurological system process |
| 42542 | 7.97E-03 | 5.44E-02 | response to hydrogen peroxide |
| 6094 | 8.12E-03 | 5.50E-02 | gluconeogenesis |
| 7031 | 8.12E-03 | 5.50E-02 | peroxisome organization |
| 19674 | 8.12E-03 | 5.50E-02 | NAD metabolic process |
| 1934 | 8.19E-03 | 5.54E-02 | positive regulation of protein amino acid phosphorylation |
| 15985 | 8.25E-03 | 5.54E-02 | energy coupled proton transport, down electrochemical gradient |
| 15986 | 8.25E-03 | 5.54E-02 | ATP synthesis coupled proton transport |
| 34637 | 9.04E-03 | 6.05E-02 | cellular carbohydrate biosynthetic process |
| 23046 | 9.07E-03 | 6.05E-02 | signaling process |
| 23060 | 9.07E-03 | 6.05E-02 | signal transmission |
| 9066 | 9.20E-03 | 6.05E-02 | aspartate family amino acid metabolic process |
| 45933 | 9.20E-03 | 6.05E-02 | positive regulation of muscle contraction |
| 42364 | 9.20E-03 | 6.05E-02 | water-soluble vitamin biosynthetic process |
| 18210 | 9.20E-03 | 6.05E-02 | peptidyl-threonine modification |
| 6691 | 9.20E-03 | 6.05E-02 | leukotriene metabolic process |
| 45779 | 9.44E-03 | 6.06E-02 | negative regulation of bone resorption |
| 9396 | 9.44E-03 | 6.06E-02 | folic acid and derivative biosynthetic process |
| 50665 | 9.44E-03 | 6.06E-02 | hydrogen peroxide biosynthetic process |
| 50884 | 9.44E-03 | 6.06E-02 | neuromuscular process controlling posture |
| 46851 | 9.44E-03 | 6.06E-02 | negative regulation of bone remodeling |
| 6563 | 9.44E-03 | 6.06E-02 | L-serine metabolic process |
| 19377 | 9.44E-03 | 6.06E-02 | glycolipid catabolic process |
| 3071 | 9.44E-03 | 6.06E-02 | renal system process involved in regulation of systemic arterial blood pressure |
| 3078 | 9.44E-03 | 6.06E-02 | regulation of natriuresis |
| 8361 | 9.53E-03 | 6.10E-02 | regulation of cell size |
| 6112 | 9.71E-03 | 6.19E-02 | energy reserve metabolic process |
| 7613 | 9.71E-03 | 6.19E-02 | memory |
| 51049 | 9.80E-03 | 6.23E-02 | regulation of transport |
| 43434 | 9.87E-03 | 6.26E-02 | response to peptide hormone stimulus |
| 9156 | 1.04E-02 | 6.51E-02 | ribonucleoside monophosphate biosynthetic process |
| 10464 | 1.04E-02 | 6.51E-02 | regulation of mesenchymal cell proliferation |
| 43449 | 1.04E-02 | 6.51E-02 | cellular alkene metabolic process |
| 48730 | 1.04E-02 | 6.51E-02 | epidermis morphogenesis |
| 46165 | 1.05E-02 | 6.57E-02 | alcohol biosynthetic process |
| 9141 | 1.06E-02 | 6.60E-02 | nucleoside triphosphate metabolic process |
| 51249 | 1.06E-02 | 6.61E-02 | regulation of lymphocyte activation |
| 51259 | 1.09E-02 | 6.76E-02 | protein oligomerization |
| 9628 | 1.10E-02 | 6.82E-02 | response to abiotic stimulus |
| 9266 | 1.12E-02 | 6.92E-02 | response to temperature stimulus |
| 32101 | 1.12E-02 | 6.92E-02 | regulation of response to external stimulus |
| 50801 | 1.15E-02 | 6.98E-02 | ion homeostasis |
| 45777 | 1.16E-02 | 6.98E-02 | positive regulation of blood pressure |
| 30890 | 1.16E-02 | 6.98E-02 | positive regulation of B cell proliferation |
| 19935 | 1.19E-02 | 6.98E-02 | cyclic-nucleotide-mediated signaling |
| 35295 | 1.19E-02 | 6.98E-02 | tube development |
| 9163 | 1.20E-02 | 6.98E-02 | nucleoside biosynthetic process |
| 46129 | 1.20E-02 | 6.98E-02 | purine ribonucleoside biosynthetic process |
| 42135 | 1.20E-02 | 6.98E-02 | neurotransmitter catabolic process |
| 34104 | 1.20E-02 | 6.98E-02 | negative regulation of tissue remodeling |
| 42451 | 1.20E-02 | 6.98E-02 | purine nucleoside biosynthetic process |
| 42455 | 1.20E-02 | 6.98E-02 | ribonucleoside biosynthetic process |
| 46653 | 1.20E-02 | 6.98E-02 | tetrahydrofolate metabolic process |
| 46685 | 1.20E-02 | 6.98E-02 | response to arsenic |
| 43090 | 1.20E-02 | 6.98E-02 | amino acid import |
| 35162 | 1.20E-02 | 6.98E-02 | embryonic hemopoiesis |
| 6570 | 1.20E-02 | 6.98E-02 | tyrosine metabolic process |
| 6599 | 1.20E-02 | 6.98E-02 | phosphagen metabolic process |
| 6600 | 1.20E-02 | 6.98E-02 | creatine metabolic process |
| 6734 | 1.20E-02 | 6.98E-02 | NADH metabolic process |
| 19359 | 1.20E-02 | 6.98E-02 | nicotinamide nucleotide biosynthetic process |
| 3084 | 1.20E-02 | 6.98E-02 | positive regulation of systemic arterial blood pressure |
| 44062 | 1.20E-02 | 6.98E-02 | regulation of excretion |
| 19934 | 1.20E-02 | 6.98E-02 | cGMP-mediated signaling |
| 32870 | 1.22E-02 | 7.11E-02 | cellular response to hormone stimulus |
| 6469 | 1.23E-02 | 7.13E-02 | negative regulation of protein kinase activity |
| 42327 | 1.29E-02 | 7.46E-02 | positive regulation of phosphorylation |
| 50806 | 1.30E-02 | 7.48E-02 | positive regulation of synaptic transmission |
| 9966 | 1.30E-02 | 7.48E-02 | regulation of signal transduction |
| 51336 | 1.32E-02 | 7.59E-02 | regulation of hydrolase activity |
| 6119 | 1.35E-02 | 7.72E-02 | oxidative phosphorylation |
| 6644 | 1.35E-02 | 7.74E-02 | phospholipid metabolic process |
| 51345 | 1.40E-02 | 7.96E-02 | positive regulation of hydrolase activity |
| 32846 | 1.41E-02 | 7.99E-02 | positive regulation of homeostatic process |
| 51591 | 1.41E-02 | 7.99E-02 | response to cAMP |
| 8645 | 1.44E-02 | 8.11E-02 | hexose transport |
| 3014 | 1.44E-02 | 8.11E-02 | renal system process |
| 15758 | 1.44E-02 | 8.11E-02 | glucose transport |
| 45937 | 1.45E-02 | 8.11E-02 | positive regulation of phosphate metabolic process |
| 10562 | 1.45E-02 | 8.11E-02 | positive regulation of phosphorus metabolic process |
| 60341 | 1.45E-02 | 8.11E-02 | regulation of cellular localization |
| 8210 | 1.48E-02 | 8.14E-02 | estrogen metabolic process |
| 30002 | 1.48E-02 | 8.14E-02 | cellular anion homeostasis |
| 7213 | 1.48E-02 | 8.14E-02 | muscarinic acetylcholine receptor signaling pathway |
| 32026 | 1.48E-02 | 8.14E-02 | response to magnesium ion |
| 33673 | 1.54E-02 | 8.14E-02 | negative regulation of kinase activity |
| 30323 | 1.54E-02 | 8.14E-02 | respiratory tube development |
| 7186 | 1.55E-02 | 8.14E-02 | G-protein coupled receptor protein signaling pathway |
| 51216 | 1.59E-02 | 8.14E-02 | cartilage development |
| 9110 | 1.59E-02 | 8.14E-02 | vitamin biosynthetic process |
| 70555 | 1.59E-02 | 8.14E-02 | response to interleukin-1 |
| 9409 | 1.59E-02 | 8.14E-02 | response to cold |
| 6752 | 1.59E-02 | 8.14E-02 | group transfer coenzyme metabolic process |
| 71495 | 1.60E-02 | 8.14E-02 | cellular response to endogenous stimulus |
| 3018 | 1.61E-02 | 8.14E-02 | vascular process in circulatory system |
| 34097 | 1.67E-02 | 8.14E-02 | response to cytokine stimulus |
| 71310 | 1.67E-02 | 8.14E-02 | cellular response to organic substance |
| 48729 | 1.68E-02 | 8.14E-02 | tissue morphogenesis |
| 55065 | 1.71E-02 | 8.14E-02 | metal ion homeostasis |
| 6811 | 1.75E-02 | 8.14E-02 | ion transport |
| 51971 | 1.75E-02 | 8.14E-02 | positive regulation of transmission of nerve impulse |
| 48661 | 1.75E-02 | 8.14E-02 | positive regulation of smooth muscle cell proliferation |
| 33628 | 1.78E-02 | 8.14E-02 | regulation of cell adhesion mediated by integrin |
| 43500 | 1.78E-02 | 8.14E-02 | muscle adaptation |
| 6809 | 1.78E-02 | 8.14E-02 | nitric oxide biosynthetic process |
| 32006 | 1.78E-02 | 8.14E-02 | regulation of TOR signaling cascade |
| 34220 | 1.83E-02 | 8.14E-02 | ion transmembrane transport |
| 42445 | 1.89E-02 | 8.14E-02 | hormone metabolic process |
| 90066 | 1.90E-02 | 8.14E-02 | regulation of anatomical structure size |
| 9605 | 1.90E-02 | 8.14E-02 | response to external stimulus |
| 23 | 1.91E-02 | 8.14E-02 | maltose metabolic process |
| 16560 | 1.91E-02 | 8.14E-02 | protein import into peroxisome matrix, docking |
| 33007 | 1.91E-02 | 8.14E-02 | negative regulation of mast cell activation involved in immune response |
| 45338 | 1.91E-02 | 8.14E-02 | farnesyl diphosphate metabolic process |
| 33345 | 1.91E-02 | 8.14E-02 | asparagine catabolic process via L-aspartate |
| 70278 | 1.91E-02 | 8.14E-02 | extracellular matrix constituent secretion |
| 70316 | 1.91E-02 | 8.14E-02 | regulation of G0 to G1 transition |
| 70318 | 1.91E-02 | 8.14E-02 | positive regulation of G0 to G1 transition |
| 70407 | 1.91E-02 | 8.14E-02 | oxidation-dependent protein catabolic process |
| 70495 | 1.91E-02 | 8.14E-02 | negative regulation of thrombin receptor signaling pathway |
| 70494 | 1.91E-02 | 8.14E-02 | regulation of thrombin receptor signaling pathway |
| 33634 | 1.91E-02 | 8.14E-02 | positive regulation of cell-cell adhesion mediated by integrin |
| 9073 | 1.91E-02 | 8.14E-02 | aromatic amino acid family biosynthetic process |
| 9078 | 1.91E-02 | 8.14E-02 | pyruvate family amino acid metabolic process |
| 9095 | 1.91E-02 | 8.14E-02 | aromatic amino acid family biosynthetic process, prephenate pathway |
| 9098 | 1.91E-02 | 8.14E-02 | leucine biosynthetic process |
| 9115 | 1.91E-02 | 8.14E-02 | xanthine catabolic process |
| 46083 | 1.91E-02 | 8.14E-02 | adenine metabolic process |
| 46084 | 1.91E-02 | 8.14E-02 | adenine biosynthetic process |
| 46086 | 1.91E-02 | 8.14E-02 | adenosine biosynthetic process |
| 46104 | 1.91E-02 | 8.14E-02 | thymidine metabolic process |
| 46127 | 1.91E-02 | 8.14E-02 | pyrimidine deoxyribonucleoside catabolic process |
| 70715 | 1.91E-02 | 8.14E-02 | sodium-dependent organic cation transport |
| 9372 | 1.91E-02 | 8.14E-02 | quorum sensing |
| 9438 | 1.91E-02 | 8.14E-02 | methylglyoxal metabolic process |
| 46314 | 1.91E-02 | 8.14E-02 | phosphocreatine biosynthetic process |
| 21768 | 1.91E-02 | 8.14E-02 | nucleus accumbens development |
| 42262 | 1.91E-02 | 8.14E-02 | DNA protection |
| 42264 | 1.91E-02 | 8.14E-02 | peptidyl-aspartic acid hydroxylation |
| 46370 | 1.91E-02 | 8.14E-02 | fructose biosynthetic process |
| 46417 | 1.91E-02 | 8.14E-02 | chorismate metabolic process |
| 46448 | 1.91E-02 | 8.14E-02 | tropane alkaloid metabolic process |
| 42413 | 1.91E-02 | 8.14E-02 | carnitine catabolic process |
| 34275 | 1.91E-02 | 8.14E-02 | kynurenic acid metabolic process |
| 34276 | 1.91E-02 | 8.14E-02 | kynurenic acid biosynthetic process |
| 46654 | 1.91E-02 | 8.14E-02 | tetrahydrofolate biosynthetic process |
| 50783 | 1.91E-02 | 8.14E-02 | cocaine metabolic process |
| 50812 | 1.91E-02 | 8.14E-02 | regulation of acyl-CoA biosynthetic process |
| 46724 | 1.91E-02 | 8.14E-02 | oxalic acid secretion |
| 34516 | 1.91E-02 | 8.14E-02 | response to vitamin B6 |
| 18197 | 1.91E-02 | 8.14E-02 | peptidyl-aspartic acid modification |
| 30505 | 1.91E-02 | 8.14E-02 | inorganic diphosphate transport |
| 42839 | 1.91E-02 | 8.14E-02 | D-glucuronate metabolic process |
| 42840 | 1.91E-02 | 8.14E-02 | D-glucuronate catabolic process |
| 18272 | 1.91E-02 | 8.14E-02 | protein-pyridoxal-5-phosphate linkage via peptidyl-N6-pyridoxal phosphate-L-lysine |
| 5985 | 1.91E-02 | 8.14E-02 | sucrose metabolic process |
| 42851 | 1.91E-02 | 8.14E-02 | L-alanine metabolic process |
| 6060 | 1.91E-02 | 8.14E-02 | sorbitol metabolic process |
| 6062 | 1.91E-02 | 8.14E-02 | sorbitol catabolic process |
| 6064 | 1.91E-02 | 8.14E-02 | glucuronate catabolic process |
| 1998 | 1.91E-02 | 8.14E-02 | angiotensin mediated vasoconstriction involved in regulation of systemic arterial blood pressure |
| 51160 | 1.91E-02 | 8.14E-02 | L-xylitol catabolic process |
| 51164 | 1.91E-02 | 8.14E-02 | L-xylitol metabolic process |
| 10260 | 1.91E-02 | 8.14E-02 | organ senescence |
| 6168 | 1.91E-02 | 8.14E-02 | adenine salvage |
| 6214 | 1.91E-02 | 8.14E-02 | thymidine catabolic process |
| 43096 | 1.91E-02 | 8.14E-02 | purine base salvage |
| 43181 | 1.91E-02 | 8.14E-02 | vacuolar sequestering |
| 43301 | 1.91E-02 | 8.14E-02 | negative regulation of leukocyte degranulation |
| 43305 | 1.91E-02 | 8.14E-02 | negative regulation of mast cell degranulation |
| 6522 | 1.91E-02 | 8.14E-02 | alanine metabolic process |
| 51581 | 1.91E-02 | 8.14E-02 | negative regulation of neurotransmitter uptake |
| 51585 | 1.91E-02 | 8.14E-02 | negative regulation of dopamine uptake |
| 6530 | 1.91E-02 | 8.14E-02 | asparagine catabolic process |
| 6535 | 1.91E-02 | 8.14E-02 | cysteine biosynthetic process from serine |
| 6550 | 1.91E-02 | 8.14E-02 | isoleucine catabolic process |
| 43418 | 1.91E-02 | 8.14E-02 | homocysteine catabolic process |
| 51611 | 1.91E-02 | 8.14E-02 | regulation of serotonin uptake |
| 43420 | 1.91E-02 | 8.14E-02 | anthranilate metabolic process |
| 51612 | 1.91E-02 | 8.14E-02 | negative regulation of serotonin uptake |
| 51621 | 1.91E-02 | 8.14E-02 | regulation of norepinephrine uptake |
| 51622 | 1.91E-02 | 8.14E-02 | negative regulation of norepinephrine uptake |
| 6571 | 1.91E-02 | 8.14E-02 | tyrosine biosynthetic process |
| 6579 | 1.91E-02 | 8.14E-02 | betaine catabolic process |
| 6603 | 1.91E-02 | 8.14E-02 | phosphocreatine metabolic process |
| 2534 | 1.91E-02 | 8.14E-02 | cytokine production involved in inflammatory response |
| 2575 | 1.91E-02 | 8.14E-02 | basophil chemotaxis |
| 14873 | 1.91E-02 | 8.14E-02 | response to muscle activity involved in regulation of muscle adaptation |
| 6683 | 1.91E-02 | 8.14E-02 | galactosylceramide catabolic process |
| 14895 | 1.91E-02 | 8.14E-02 | smooth muscle hypertrophy |
| 6741 | 1.91E-02 | 8.14E-02 | NADP biosynthetic process |
| 2649 | 1.91E-02 | 8.14E-02 | regulation of tolerance induction to self antigen |
| 2651 | 1.91E-02 | 8.14E-02 | positive regulation of tolerance induction to self antigen |
| 10878 | 1.91E-02 | 8.14E-02 | cholesterol storage |
| 51939 | 1.91E-02 | 8.14E-02 | gamma-aminobutyric acid import |
| 51945 | 1.91E-02 | 8.14E-02 | negative regulation of catecholamine uptake involved in synaptic transmission |
| 2830 | 1.91E-02 | 8.14E-02 | positive regulation of T-helper 2 type immune response |
| 60215 | 1.91E-02 | 8.14E-02 | primitive hemopoiesis |
| 19265 | 1.91E-02 | 8.14E-02 | glycine biosynthetic process, by transamination of glyoxylate |
| 52097 | 1.91E-02 | 8.14E-02 | interspecies quorum sensing |
| 52106 | 1.91E-02 | 8.14E-02 | quorum sensing involved in interaction with host |
| 19343 | 1.91E-02 | 8.14E-02 | cysteine biosynthetic process via cystathionine |
| 60319 | 1.91E-02 | 8.14E-02 | primitive erythrocyte differentiation |
| 19376 | 1.91E-02 | 8.14E-02 | galactolipid catabolic process |
| 60356 | 1.91E-02 | 8.14E-02 | leucine import |
| 19407 | 1.91E-02 | 8.14E-02 | hexitol catabolic process |
| 7192 | 1.91E-02 | 8.14E-02 | activation of adenylate cyclase activity by serotonin receptor signaling pathway |
| 7208 | 1.91E-02 | 8.14E-02 | activation of phospholipase C activity by serotonin receptor signaling pathway |
| 19519 | 1.91E-02 | 8.14E-02 | pentitol metabolic process |
| 19527 | 1.91E-02 | 8.14E-02 | pentitol catabolic process |
| 60730 | 1.91E-02 | 8.14E-02 | regulation of intestinal epithelial structure maintenance |
| 60731 | 1.91E-02 | 8.14E-02 | positive regulation of intestinal epithelial structure maintenance |
| 15739 | 1.91E-02 | 8.14E-02 | sialic acid transport |
| 15803 | 1.91E-02 | 8.14E-02 | branched-chain aliphatic amino acid transport |
| 15820 | 1.91E-02 | 8.14E-02 | leucine transport |
| 15827 | 1.91E-02 | 8.14E-02 | tryptophan transport |
| 48874 | 1.91E-02 | 8.14E-02 | homeostasis of number of cells in a free-living population |
| 32530 | 1.91E-02 | 8.14E-02 | regulation of microvillus organization |
| 32534 | 1.91E-02 | 8.14E-02 | regulation of microvillus assembly |
| 3008 | 1.95E-02 | 8.26E-02 | system process |
| 42326 | 1.95E-02 | 8.26E-02 | negative regulation of phosphorylation |
| 50796 | 1.95E-02 | 8.26E-02 | regulation of insulin secretion |
| 48771 | 1.95E-02 | 8.26E-02 | tissue remodeling |
| 51348 | 1.97E-02 | 8.31E-02 | negative regulation of transferase activity |
| 9205 | 2.05E-02 | 8.63E-02 | purine ribonucleoside triphosphate metabolic process |
| 7165 | 2.05E-02 | 8.64E-02 | signal transduction |
| 7612 | 2.07E-02 | 8.70E-02 | learning |
| 45428 | 2.09E-02 | 8.70E-02 | regulation of nitric oxide biosynthetic process |
| 46128 | 2.09E-02 | 8.70E-02 | purine ribonucleoside metabolic process |
| 46324 | 2.09E-02 | 8.70E-02 | regulation of glucose import |
| 42278 | 2.09E-02 | 8.70E-02 | purine nucleoside metabolic process |
| 31646 | 2.09E-02 | 8.70E-02 | positive regulation of neurological system process |
| 90181 | 2.12E-02 | 8.70E-02 | regulation of cholesterol metabolic process |
| 9083 | 2.12E-02 | 8.70E-02 | branched chain family amino acid catabolic process |
| 46112 | 2.12E-02 | 8.70E-02 | nucleobase biosynthetic process |
| 33865 | 2.12E-02 | 8.70E-02 | nucleoside bisphosphate metabolic process |
| 46209 | 2.12E-02 | 8.70E-02 | nitric oxide metabolic process |
| 14061 | 2.12E-02 | 8.70E-02 | regulation of norepinephrine secretion |
| 50930 | 2.12E-02 | 8.70E-02 | induction of positive chemotaxis |
| 7616 | 2.12E-02 | 8.70E-02 | long-term memory |
| 9199 | 2.13E-02 | 8.72E-02 | ribonucleoside triphosphate metabolic process |
| 60541 | 2.13E-02 | 8.72E-02 | respiratory system development |
| 55080 | 2.13E-02 | 8.72E-02 | cation homeostasis |
| 7568 | 2.14E-02 | 8.73E-02 | aging |
| 46890 | 2.20E-02 | 8.98E-02 | regulation of lipid biosynthetic process |
| 33555 | 2.27E-02 | 9.24E-02 | multicellular organismal response to stress |
| 30888 | 2.27E-02 | 9.24E-02 | regulation of B cell proliferation |
| 48167 | 2.33E-02 | 9.47E-02 | regulation of synaptic plasticity |
| 7187 | 2.39E-02 | 9.65E-02 | G-protein signaling, coupled to cyclic nucleotide second messenger |
| 32496 | 2.39E-02 | 9.65E-02 | response to lipopolysaccharide |
| 46320 | 2.47E-02 | 9.78E-02 | regulation of fatty acid oxidation |
| 30856 | 2.47E-02 | 9.78E-02 | regulation of epithelial cell differentiation |
| 45730 | 2.47E-02 | 9.78E-02 | respiratory burst |
| 45922 | 2.47E-02 | 9.78E-02 | negative regulation of fatty acid metabolic process |
| 9154 | 2.47E-02 | 9.78E-02 | purine ribonucleotide catabolic process |
| 42559 | 2.47E-02 | 9.78E-02 | pteridine and derivative biosynthetic process |
| 14059 | 2.47E-02 | 9.78E-02 | regulation of dopamine secretion |
| 55081 | 2.47E-02 | 9.78E-02 | anion homeostasis |
| 10744 | 2.47E-02 | 9.78E-02 | positive regulation of macrophage derived foam cell differentiation |
| 6833 | 2.47E-02 | 9.78E-02 | water transport |
| 45936 | 2.47E-02 | 9.78E-02 | negative regulation of phosphate metabolic process |
| 10563 | 2.47E-02 | 9.78E-02 | negative regulation of phosphorus metabolic process |
| 16049 | 2.47E-02 | 9.78E-02 | cell growth |
| 9144 | 2.48E-02 | 9.79E-02 | purine nucleoside triphosphate metabolic process |
| 32103 | 2.55E-02 | 1.01E-01 | positive regulation of response to external stimulus |
| 48514 | 2.57E-02 | 1.01E-01 | blood vessel morphogenesis |
| 6873 | 2.58E-02 | 1.02E-01 | cellular ion homeostasis |
| 90276 | 2.61E-02 | 1.03E-01 | regulation of peptide hormone secretion |
| 70085 | 2.66E-02 | 1.04E-01 | glycosylation |
| 6486 | 2.66E-02 | 1.04E-01 | protein amino acid glycosylation |
| 43413 | 2.66E-02 | 1.04E-01 | macromolecule glycosylation |
| 43588 | 2.67E-02 | 1.04E-01 | skin development |
| 42044 | 2.84E-02 | 1.10E-01 | fluid transport |
| 42554 | 2.84E-02 | 1.10E-01 | superoxide anion generation |
| 51602 | 2.84E-02 | 1.10E-01 | response to electrical stimulus |
| 6625 | 2.84E-02 | 1.10E-01 | protein targeting to peroxisome |
| 48265 | 2.84E-02 | 1.10E-01 | response to pain |
| 33993 | 2.88E-02 | 1.11E-01 | response to lipid |
| 48168 | 2.88E-02 | 1.11E-01 | regulation of neuronal synaptic plasticity |
| 55082 | 2.89E-02 | 1.11E-01 | cellular chemical homeostasis |
| 6754 | 2.90E-02 | 1.11E-01 | ATP biosynthetic process |
| 2791 | 2.91E-02 | 1.12E-01 | regulation of peptide secretion |
| 90087 | 2.91E-02 | 1.12E-01 | regulation of peptide transport |
| 35556 | 2.93E-02 | 1.12E-01 | intracellular signal transduction |
| 51480 | 2.96E-02 | 1.13E-01 | cytosolic calcium ion homeostasis |
| 18130 | 3.06E-02 | 1.17E-01 | heterocycle biosynthetic process |
| 55088 | 3.06E-02 | 1.17E-01 | lipid homeostasis |
| 8284 | 3.07E-02 | 1.17E-01 | positive regulation of cell proliferation |
| 32355 | 3.22E-02 | 1.21E-01 | response to estradiol stimulus |
| 33574 | 3.24E-02 | 1.21E-01 | response to testosterone stimulus |
| 9143 | 3.24E-02 | 1.21E-01 | nucleoside triphosphate catabolic process |
| 9261 | 3.24E-02 | 1.21E-01 | ribonucleotide catabolic process |
| 43574 | 3.24E-02 | 1.21E-01 | peroxisomal transport |
| 19363 | 3.24E-02 | 1.21E-01 | pyridine nucleotide biosynthetic process |
| 7620 | 3.24E-02 | 1.21E-01 | copulation |
| 50670 | 3.27E-02 | 1.21E-01 | regulation of lymphocyte proliferation |
| 9100 | 3.30E-02 | 1.21E-01 | glycoprotein metabolic process |
| 50871 | 3.32E-02 | 1.21E-01 | positive regulation of B cell activation |
| 6220 | 3.32E-02 | 1.21E-01 | pyrimidine nucleotide metabolic process |
| 60070 | 3.32E-02 | 1.21E-01 | canonical Wnt receptor signaling pathway |
| 2682 | 3.35E-02 | 1.21E-01 | regulation of immune system process |
| 42476 | 3.39E-02 | 1.21E-01 | odontogenesis |
| 50671 | 3.39E-02 | 1.21E-01 | positive regulation of lymphocyte proliferation |
| 6875 | 3.39E-02 | 1.21E-01 | cellular metal ion homeostasis |
| 32944 | 3.41E-02 | 1.21E-01 | regulation of mononuclear cell proliferation |
| 2237 | 3.50E-02 | 1.21E-01 | response to molecule of bacterial origin |
| 70663 | 3.54E-02 | 1.21E-01 | regulation of leukocyte proliferation |
| 32946 | 3.56E-02 | 1.21E-01 | positive regulation of mononuclear cell proliferation |
| 9791 | 3.56E-02 | 1.21E-01 | post-embryonic development |
| 6800 | 3.56E-02 | 1.21E-01 | oxygen and reactive oxygen species metabolic process |
| 45124 | 3.66E-02 | 1.21E-01 | regulation of bone resorption |
| 97 | 3.66E-02 | 1.21E-01 | sulfur amino acid biosynthetic process |
| 45599 | 3.66E-02 | 1.21E-01 | negative regulation of fat cell differentiation |
| 70542 | 3.66E-02 | 1.21E-01 | response to fatty acid |
| 46676 | 3.66E-02 | 1.21E-01 | negative regulation of insulin secretion |
| 14072 | 3.66E-02 | 1.21E-01 | response to isoquinoline alkaloid |
| 46850 | 3.66E-02 | 1.21E-01 | regulation of bone remodeling |
| 6103 | 3.66E-02 | 1.21E-01 | 2-oxoglutarate metabolic process |
| 43278 | 3.66E-02 | 1.21E-01 | response to morphine |
| 6760 | 3.66E-02 | 1.21E-01 | folic acid and derivative metabolic process |
| 42981 | 3.66E-02 | 1.21E-01 | regulation of apoptosis |
| 31668 | 3.68E-02 | 1.21E-01 | cellular response to extracellular stimulus |
| 43085 | 3.69E-02 | 1.21E-01 | positive regulation of catalytic activity |
| 70665 | 3.73E-02 | 1.21E-01 | positive regulation of leukocyte proliferation |
| 34754 | 3.73E-02 | 1.21E-01 | cellular hormone metabolic process |
| 6916 | 3.75E-02 | 1.21E-01 | anti-apoptosis |
| 98 | 3.78E-02 | 1.21E-01 | sulfur amino acid catabolic process |
| 45229 | 3.78E-02 | 1.21E-01 | external encapsulating structure organization |
| 45719 | 3.78E-02 | 1.21E-01 | negative regulation of glycogen biosynthetic process |
| 33484 | 3.78E-02 | 1.21E-01 | nitric oxide homeostasis |
| 45915 | 3.78E-02 | 1.21E-01 | positive regulation of catecholamine metabolic process |
| 9082 | 3.78E-02 | 1.21E-01 | branched chain family amino acid biosynthetic process |
| 9093 | 3.78E-02 | 1.21E-01 | cysteine catabolic process |
| 45964 | 3.78E-02 | 1.21E-01 | positive regulation of dopamine metabolic process |
| 9128 | 3.78E-02 | 1.21E-01 | purine nucleoside monophosphate catabolic process |
| 9158 | 3.78E-02 | 1.21E-01 | ribonucleoside monophosphate catabolic process |
| 9169 | 3.78E-02 | 1.21E-01 | purine ribonucleoside monophosphate catabolic process |
| 46108 | 3.78E-02 | 1.21E-01 | uridine metabolic process |
| 46110 | 3.78E-02 | 1.21E-01 | xanthine metabolic process |
| 46135 | 3.78E-02 | 1.21E-01 | pyrimidine nucleoside catabolic process |
| 33864 | 3.78E-02 | 1.21E-01 | positive regulation of NAD(P)H oxidase activity |
| 33875 | 3.78E-02 | 1.21E-01 | ribonucleoside bisphosphate metabolic process |
| 46168 | 3.78E-02 | 1.21E-01 | glycerol-3-phosphate catabolic process |
| 46185 | 3.78E-02 | 1.21E-01 | aldehyde catabolic process |
| 70836 | 3.78E-02 | 1.21E-01 | caveola assembly |
| 9450 | 3.78E-02 | 1.21E-01 | gamma-aminobutyric acid catabolic process |
| 34032 | 3.78E-02 | 1.21E-01 | purine nucleoside bisphosphate metabolic process |
| 34035 | 3.78E-02 | 1.21E-01 | purine ribonucleoside bisphosphate metabolic process |
| 50427 | 3.78E-02 | 1.21E-01 | 3'-phosphoadenosine 5'-phosphosulfate metabolic process |
| 34059 | 3.78E-02 | 1.21E-01 | response to anoxia |
| 46439 | 3.78E-02 | 1.21E-01 | L-cysteine metabolic process |
| 30070 | 3.78E-02 | 1.21E-01 | insulin processing |
| 42524 | 3.78E-02 | 1.21E-01 | negative regulation of tyrosine phosphorylation of Stat5 protein |
| 1692 | 3.78E-02 | 1.21E-01 | histamine metabolic process |
| 50882 | 3.78E-02 | 1.21E-01 | voluntary musculoskeletal movement |
| 1765 | 3.78E-02 | 1.21E-01 | membrane raft assembly |
| 14062 | 3.78E-02 | 1.21E-01 | regulation of serotonin secretion |
| 14063 | 3.78E-02 | 1.21E-01 | negative regulation of serotonin secretion |
| 42760 | 3.78E-02 | 1.21E-01 | very long-chain fatty acid catabolic process |
| 34619 | 3.78E-02 | 1.21E-01 | cellular chaperone-mediated protein complex assembly |
| 10044 | 3.78E-02 | 1.21E-01 | response to aluminum ion |
| 6059 | 3.78E-02 | 1.21E-01 | hexitol metabolic process |
| 18352 | 3.78E-02 | 1.21E-01 | protein-pyridoxal-5-phosphate linkage |
| 1999 | 3.78E-02 | 1.21E-01 | renal response to blood flow involved in circulatory renin-angiotensin regulation of systemic arterial blood pressure |
| 6097 | 3.78E-02 | 1.21E-01 | glyoxylate cycle |
| 2017 | 3.78E-02 | 1.21E-01 | regulation of blood volume by renal aldosterone |
| 2018 | 3.78E-02 | 1.21E-01 | renin-angiotensin regulation of aldosterone production |
| 2019 | 3.78E-02 | 1.21E-01 | regulation of renal output by angiotensin |
| 6116 | 3.78E-02 | 1.21E-01 | NADH oxidation |
| 42986 | 3.78E-02 | 1.21E-01 | positive regulation of amyloid precursor protein biosynthetic process |
| 2086 | 3.78E-02 | 1.21E-01 | diaphragm contraction |
| 6184 | 3.78E-02 | 1.21E-01 | GTP catabolic process |
| 6196 | 3.78E-02 | 1.21E-01 | AMP catabolic process |
| 6212 | 3.78E-02 | 1.21E-01 | uracil catabolic process |
| 2138 | 3.78E-02 | 1.21E-01 | retinoic acid biosynthetic process |
| 43117 | 3.78E-02 | 1.21E-01 | positive regulation of vascular permeability |
| 43163 | 3.78E-02 | 1.21E-01 | cell envelope organization |
| 35106 | 3.78E-02 | 1.21E-01 | operant conditioning |
| 51589 | 3.78E-02 | 1.21E-01 | negative regulation of neurotransmitter transport |
| 10642 | 3.78E-02 | 1.21E-01 | negative regulation of platelet-derived growth factor receptor signaling pathway |
| 18874 | 3.78E-02 | 1.21E-01 | benzoate metabolic process |
| 14806 | 3.78E-02 | 1.21E-01 | smooth muscle hyperplasia |
| 31296 | 3.78E-02 | 1.21E-01 | B cell costimulation |
| 2661 | 3.78E-02 | 1.21E-01 | regulation of B cell tolerance induction |
| 2663 | 3.78E-02 | 1.21E-01 | positive regulation of B cell tolerance induction |
| 6788 | 3.78E-02 | 1.21E-01 | heme oxidation |
| 60083 | 3.78E-02 | 1.21E-01 | smooth muscle contraction involved in micturition |
| 51932 | 3.78E-02 | 1.21E-01 | synaptic transmission, GABAergic |
| 10996 | 3.78E-02 | 1.21E-01 | response to auditory stimulus |
| 60151 | 3.78E-02 | 1.21E-01 | peroxisome localization |
| 60152 | 3.78E-02 | 1.21E-01 | microtubule-based peroxisome localization |
| 60166 | 3.78E-02 | 1.21E-01 | olfactory pit development |
| 19254 | 3.78E-02 | 1.21E-01 | carnitine metabolic process, CoA-linked |
| 60346 | 3.78E-02 | 1.21E-01 | bone trabecula formation |
| 3011 | 3.78E-02 | 1.21E-01 | involuntary skeletal muscle contraction |
| 19442 | 3.78E-02 | 1.21E-01 | tryptophan catabolic process to acetyl-CoA |
| 19448 | 3.78E-02 | 1.21E-01 | L-cysteine catabolic process |
| 60452 | 3.78E-02 | 1.21E-01 | positive regulation of cardiac muscle contraction |
| 19563 | 3.78E-02 | 1.21E-01 | glycerol catabolic process |
| 44273 | 3.78E-02 | 1.21E-01 | sulfur compound catabolic process |
| 48378 | 3.78E-02 | 1.21E-01 | regulation of lateral mesodermal cell fate specification |
| 60732 | 3.78E-02 | 1.21E-01 | positive regulation of inositol phosphate biosynthetic process |
| 15740 | 3.78E-02 | 1.21E-01 | C4-dicarboxylate transport |
| 15744 | 3.78E-02 | 1.21E-01 | succinate transport |
| 19853 | 3.78E-02 | 1.21E-01 | L-ascorbic acid biosynthetic process |
| 19860 | 3.78E-02 | 1.21E-01 | uracil metabolic process |
| 15801 | 3.78E-02 | 1.21E-01 | aromatic amino acid transport |
| 48627 | 3.78E-02 | 1.21E-01 | myoblast development |
| 48659 | 3.78E-02 | 1.21E-01 | smooth muscle cell proliferation |
| 15942 | 3.78E-02 | 1.21E-01 | formate metabolic process |
| 8065 | 3.78E-02 | 1.21E-01 | establishment of blood-nerve barrier |
| 32693 | 3.78E-02 | 1.21E-01 | negative regulation of interleukin-10 production |
| 32764 | 3.78E-02 | 1.21E-01 | negative regulation of mast cell cytokine production |
| 44236 | 3.80E-02 | 1.21E-01 | multicellular organismal metabolic process |
| 71496 | 3.82E-02 | 1.21E-01 | cellular response to external stimulus |
| 7154 | 3.82E-02 | 1.21E-01 | cell communication |
| 43067 | 4.01E-02 | 1.27E-01 | regulation of programmed cell death |
| 45087 | 4.03E-02 | 1.28E-01 | innate immune response |
| 15672 | 4.05E-02 | 1.28E-01 | monovalent inorganic cation transport |
| 1933 | 4.05E-02 | 1.28E-01 | negative regulation of protein amino acid phosphorylation |
| 42325 | 4.06E-02 | 1.28E-01 | regulation of phosphorylation |
| 90278 | 4.09E-02 | 1.28E-01 | negative regulation of peptide hormone secretion |
| 9081 | 4.09E-02 | 1.28E-01 | branched chain family amino acid metabolic process |
| 9084 | 4.09E-02 | 1.28E-01 | glutamine family amino acid biosynthetic process |
| 9127 | 4.09E-02 | 1.28E-01 | purine nucleoside monophosphate biosynthetic process |
| 9168 | 4.09E-02 | 1.28E-01 | purine ribonucleoside monophosphate biosynthetic process |
| 30149 | 4.09E-02 | 1.28E-01 | sphingolipid catabolic process |
| 46627 | 4.09E-02 | 1.28E-01 | negative regulation of insulin receptor signaling pathway |
| 10149 | 4.09E-02 | 1.28E-01 | senescence |
| 6541 | 4.09E-02 | 1.28E-01 | glutamine metabolic process |
| 6739 | 4.09E-02 | 1.28E-01 | NADP metabolic process |
| 50790 | 4.15E-02 | 1.29E-01 | regulation of catalytic activity |
| 3012 | 4.26E-02 | 1.32E-01 | muscle system process |
| 46034 | 4.27E-02 | 1.32E-01 | ATP metabolic process |
| 9206 | 4.27E-02 | 1.32E-01 | purine ribonucleoside triphosphate biosynthetic process |
| 1822 | 4.27E-02 | 1.32E-01 | kidney development |
| 50880 | 4.31E-02 | 1.33E-01 | regulation of blood vessel size |
| 35150 | 4.31E-02 | 1.33E-01 | regulation of tube size |
| 10941 | 4.34E-02 | 1.34E-01 | regulation of cell death |
| 9145 | 4.42E-02 | 1.36E-01 | purine nucleoside triphosphate biosynthetic process |
| 9201 | 4.42E-02 | 1.36E-01 | ribonucleoside triphosphate biosynthetic process |
| 45987 | 4.55E-02 | 1.38E-01 | positive regulation of smooth muscle contraction |
| 46131 | 4.55E-02 | 1.38E-01 | pyrimidine ribonucleoside metabolic process |
| 46466 | 4.55E-02 | 1.38E-01 | membrane lipid catabolic process |
| 51055 | 4.55E-02 | 1.38E-01 | negative regulation of lipid biosynthetic process |
| 6516 | 4.55E-02 | 1.38E-01 | glycoprotein catabolic process |
| 2792 | 4.55E-02 | 1.38E-01 | negative regulation of peptide secretion |
| 31623 | 4.55E-02 | 1.38E-01 | receptor internalization |
| 48008 | 4.55E-02 | 1.38E-01 | platelet-derived growth factor receptor signaling pathway |
| 7212 | 4.55E-02 | 1.38E-01 | dopamine receptor signaling pathway |
| 32535 | 4.57E-02 | 1.39E-01 | regulation of cellular component size |
| 33273 | 4.58E-02 | 1.39E-01 | response to vitamin |
| 30324 | 4.58E-02 | 1.39E-01 | lung development |
| 43407 | 4.58E-02 | 1.39E-01 | negative regulation of MAP kinase activity |
| 9142 | 4.74E-02 | 1.43E-01 | nucleoside triphosphate biosynthetic process |
| 30308 | 4.74E-02 | 1.43E-01 | negative regulation of cell growth |
| 72001 | 4.74E-02 | 1.43E-01 | renal system development |
| 48523 | 4.82E-02 | 1.45E-01 | negative regulation of cellular process |
| 10675 | 4.86E-02 | 1.46E-01 | regulation of cellular carbohydrate metabolic process |
| 60021 | 4.86E-02 | 1.46E-01 | palate development |
| 34103 | 5.02E-02 | 1.50E-01 | regulation of tissue remodeling |
| 1569 | 5.02E-02 | 1.50E-01 | patterning of blood vessels |
| 6471 | 5.02E-02 | 1.50E-01 | protein amino acid ADP-ribosylation |
| 2698 | 5.02E-02 | 1.50E-01 | negative regulation of immune effector process |
| 32228 | 5.02E-02 | 1.50E-01 | regulation of synaptic transmission, GABAergic |
| 6952 | 5.14E-02 | 1.53E-01 | defense response |
| 17157 | 5.14E-02 | 1.53E-01 | regulation of exocytosis |
| 6109 | 5.14E-02 | 1.53E-01 | regulation of carbohydrate metabolic process |
| 51341 | 5.14E-02 | 1.53E-01 | regulation of oxidoreductase activity |
| 19218 | 5.14E-02 | 1.53E-01 | regulation of steroid metabolic process |
| 48660 | 5.14E-02 | 1.53E-01 | regulation of smooth muscle cell proliferation |
| 30003 | 5.22E-02 | 1.53E-01 | cellular cation homeostasis |
| 16052 | 5.42E-02 | 1.53E-01 | carbohydrate catabolic process |
| 45744 | 5.51E-02 | 1.53E-01 | negative regulation of G-protein coupled receptor protein signaling pathway |
| 1659 | 5.51E-02 | 1.53E-01 | temperature homeostasis |
| 44092 | 5.59E-02 | 1.53E-01 | negative regulation of molecular function |
| 103 | 5.62E-02 | 1.53E-01 | sulfate assimilation |
| 8295 | 5.62E-02 | 1.53E-01 | spermidine biosynthetic process |
| 16559 | 5.62E-02 | 1.53E-01 | peroxisome fission |
| 33004 | 5.62E-02 | 1.53E-01 | negative regulation of mast cell activation |
| 45329 | 5.62E-02 | 1.53E-01 | carnitine biosynthetic process |
| 17004 | 5.62E-02 | 1.53E-01 | cytochrome complex assembly |
| 45760 | 5.62E-02 | 1.53E-01 | positive regulation of action potential |
| 45916 | 5.62E-02 | 1.53E-01 | negative regulation of complement activation |
| 33630 | 5.62E-02 | 1.53E-01 | positive regulation of cell adhesion mediated by integrin |
| 46007 | 5.62E-02 | 1.53E-01 | negative regulation of activated T cell proliferation |
| 9173 | 5.62E-02 | 1.53E-01 | pyrimidine ribonucleoside monophosphate metabolic process |
| 9174 | 5.62E-02 | 1.53E-01 | pyrimidine ribonucleoside monophosphate biosynthetic process |
| 46049 | 5.62E-02 | 1.53E-01 | UMP metabolic process |
| 46085 | 5.62E-02 | 1.53E-01 | adenosine metabolic process |
| 46121 | 5.62E-02 | 1.53E-01 | deoxyribonucleoside catabolic process |
| 9448 | 5.62E-02 | 1.53E-01 | gamma-aminobutyric acid metabolic process |
| 42396 | 5.62E-02 | 1.53E-01 | phosphagen biosynthetic process |
| 1543 | 5.62E-02 | 1.53E-01 | ovarian follicle rupture |
| 34371 | 5.62E-02 | 1.53E-01 | chylomicron remodeling |
| 30320 | 5.62E-02 | 1.53E-01 | cellular monovalent inorganic anion homeostasis |
| 34433 | 5.62E-02 | 1.53E-01 | steroid esterification |
| 34434 | 5.62E-02 | 1.53E-01 | sterol esterification |
| 34435 | 5.62E-02 | 1.53E-01 | cholesterol esterification |
| 34505 | 5.62E-02 | 1.53E-01 | tooth mineralization |
| 55064 | 5.62E-02 | 1.53E-01 | chloride ion homeostasis |
| 55083 | 5.62E-02 | 1.53E-01 | monovalent inorganic anion homeostasis |
| 42866 | 5.62E-02 | 1.53E-01 | pyruvate biosynthetic process |
| 10107 | 5.62E-02 | 1.53E-01 | potassium ion import |
| 30644 | 5.62E-02 | 1.53E-01 | cellular chloride ion homeostasis |
| 51136 | 5.62E-02 | 1.53E-01 | regulation of NK T cell differentiation |
| 51138 | 5.62E-02 | 1.53E-01 | positive regulation of NK T cell differentiation |
| 6085 | 5.62E-02 | 1.53E-01 | acetyl-CoA biosynthetic process |
| 10193 | 5.62E-02 | 1.53E-01 | response to ozone |
| 18401 | 5.62E-02 | 1.53E-01 | peptidyl-proline hydroxylation to 4-hydroxy-L-proline |
| 2034 | 5.62E-02 | 1.53E-01 | regulation of blood vessel size by renin-angiotensin |
| 2035 | 5.62E-02 | 1.53E-01 | brain renin-angiotensin system |
| 30730 | 5.62E-02 | 1.53E-01 | sequestering of triglyceride |
| 2072 | 5.62E-02 | 1.53E-01 | optic cup morphogenesis involved in camera-type eye development |
| 6208 | 5.62E-02 | 1.53E-01 | pyrimidine base catabolic process |
| 6210 | 5.62E-02 | 1.53E-01 | thymine catabolic process |
| 6222 | 5.62E-02 | 1.53E-01 | UMP biosynthetic process |
| 35067 | 5.62E-02 | 1.53E-01 | negative regulation of histone acetylation |
| 31077 | 5.62E-02 | 1.53E-01 | post-embryonic camera-type eye development |
| 6528 | 5.62E-02 | 1.53E-01 | asparagine metabolic process |
| 6564 | 5.62E-02 | 1.53E-01 | L-serine biosynthetic process |
| 6565 | 5.62E-02 | 1.53E-01 | L-serine catabolic process |
| 10715 | 5.62E-02 | 1.53E-01 | regulation of extracellular matrix disassembly |
| 10716 | 5.62E-02 | 1.53E-01 | negative regulation of extracellular matrix disassembly |
| 14832 | 5.62E-02 | 1.53E-01 | urinary bladder smooth muscle contraction |
| 10742 | 5.62E-02 | 1.53E-01 | macrophage derived foam cell differentiation |
| 14848 | 5.62E-02 | 1.53E-01 | urinary tract smooth muscle contraction |
| 14874 | 5.62E-02 | 1.53E-01 | response to stimulus involved in regulation of muscle adaptation |
| 6689 | 5.62E-02 | 1.53E-01 | ganglioside catabolic process |
| 10800 | 5.62E-02 | 1.53E-01 | positive regulation of peptidyl-threonine phosphorylation |
| 2636 | 5.62E-02 | 1.53E-01 | positive regulation of germinal center formation |
| 10919 | 5.62E-02 | 1.53E-01 | regulation of inositol phosphate biosynthetic process |
| 19371 | 5.62E-02 | 1.53E-01 | cyclooxygenase pathway |
| 3072 | 5.62E-02 | 1.53E-01 | renal control of peripheral vascular resistance involved in regulation of systemic arterial blood pressure |
| 19471 | 5.62E-02 | 1.53E-01 | 4-hydroxyproline metabolic process |
| 48170 | 5.62E-02 | 1.53E-01 | positive regulation of long-term neuronal synaptic plasticity |
| 19511 | 5.62E-02 | 1.53E-01 | peptidyl-proline hydroxylation |
| 48305 | 5.62E-02 | 1.53E-01 | immunoglobulin secretion |
| 19805 | 5.62E-02 | 1.53E-01 | quinolinate biosynthetic process |
| 19859 | 5.62E-02 | 1.53E-01 | thymine metabolic process |
| 48552 | 5.62E-02 | 1.53E-01 | regulation of metalloenzyme activity |
| 48554 | 5.62E-02 | 1.53E-01 | positive regulation of metalloenzyme activity |
| 7614 | 5.62E-02 | 1.53E-01 | short-term memory |
| 32224 | 5.62E-02 | 1.53E-01 | positive regulation of synaptic transmission, cholinergic |
| 32497 | 5.62E-02 | 1.53E-01 | detection of lipopolysaccharide |
| 90077 | 5.62E-02 | 1.53E-01 | foam cell differentiation |
| 32763 | 5.62E-02 | 1.53E-01 | regulation of mast cell cytokine production |
| 51174 | 5.67E-02 | 1.54E-01 | regulation of phosphorus metabolic process |
| 19220 | 5.67E-02 | 1.54E-01 | regulation of phosphate metabolic process |
| 1932 | 5.70E-02 | 1.54E-01 | regulation of protein amino acid phosphorylation |
| 42127 | 5.70E-02 | 1.54E-01 | regulation of cell proliferation |
| 6672 | 5.74E-02 | 1.55E-01 | ceramide metabolic process |
| 45471 | 5.75E-02 | 1.55E-01 | response to ethanol |
| 8654 | 5.79E-02 | 1.56E-01 | phospholipid biosynthetic process |
| 7267 | 5.83E-02 | 1.57E-01 | cell-cell signaling |
| 23052 | 5.89E-02 | 1.58E-01 | signaling |
| 45792 | 5.97E-02 | 1.60E-01 | negative regulation of cell size |
| 50730 | 5.97E-02 | 1.60E-01 | regulation of peptidyl-tyrosine phosphorylation |
| 50870 | 5.97E-02 | 1.60E-01 | positive regulation of T cell activation |
| 32845 | 6.02E-02 | 1.60E-01 | negative regulation of homeostatic process |
| 33344 | 6.02E-02 | 1.60E-01 | cholesterol efflux |
| 46626 | 6.02E-02 | 1.60E-01 | regulation of insulin receptor signaling pathway |
| 50879 | 6.02E-02 | 1.60E-01 | multicellular organismal movement |
| 50881 | 6.02E-02 | 1.60E-01 | musculoskeletal movement |
| 50927 | 6.02E-02 | 1.60E-01 | positive regulation of positive chemotaxis |
| 43154 | 6.02E-02 | 1.60E-01 | negative regulation of caspase activity |
| 6692 | 6.02E-02 | 1.60E-01 | prostanoid metabolic process |
| 6693 | 6.02E-02 | 1.60E-01 | prostaglandin metabolic process |
| 60401 | 6.02E-02 | 1.60E-01 | cytosolic calcium ion transport |
| 48519 | 6.12E-02 | 1.62E-01 | negative regulation of biological process |
| 46164 | 6.21E-02 | 1.64E-01 | alcohol catabolic process |
| 43086 | 6.27E-02 | 1.66E-01 | negative regulation of catalytic activity |
| 6874 | 6.33E-02 | 1.67E-01 | cellular calcium ion homeostasis |
| 7204 | 6.35E-02 | 1.68E-01 | elevation of cytosolic calcium ion concentration |
| 32879 | 6.42E-02 | 1.69E-01 | regulation of localization |
| 42220 | 6.54E-02 | 1.70E-01 | response to cocaine |
| 30004 | 6.54E-02 | 1.70E-01 | cellular monovalent inorganic cation homeostasis |
| 34367 | 6.54E-02 | 1.70E-01 | macromolecular complex remodeling |
| 34368 | 6.54E-02 | 1.70E-01 | protein-lipid complex remodeling |
| 34369 | 6.54E-02 | 1.70E-01 | plasma lipoprotein particle remodeling |
| 50926 | 6.54E-02 | 1.70E-01 | regulation of positive chemotaxis |
| 14073 | 6.54E-02 | 1.70E-01 | response to tropane |
| 1974 | 6.54E-02 | 1.70E-01 | blood vessel remodeling |
| 1990 | 6.54E-02 | 1.70E-01 | regulation of systemic arterial blood pressure by hormone |
| 6099 | 6.54E-02 | 1.70E-01 | tricarboxylic acid cycle |
| 10524 | 6.54E-02 | 1.70E-01 | positive regulation of calcium ion transport into cytosol |
| 8544 | 6.63E-02 | 1.73E-01 | epidermis development |
| 6638 | 6.68E-02 | 1.74E-01 | neutral lipid metabolic process |
| 6812 | 6.79E-02 | 1.76E-01 | cation transport |
| 9101 | 6.80E-02 | 1.77E-01 | glycoprotein biosynthetic process |
| 35466 | 6.93E-02 | 1.79E-01 | regulation of signaling pathway |
| 44275 | 6.93E-02 | 1.79E-01 | cellular carbohydrate catabolic process |
| 2822 | 7.01E-02 | 1.79E-01 | regulation of adaptive immune response based on somatic recombination of immune receptors built from immunoglobulin superfamily domains |
| 46356 | 7.07E-02 | 1.79E-01 | acetyl-CoA catabolic process |
| 6687 | 7.07E-02 | 1.79E-01 | glycosphingolipid metabolic process |
| 48701 | 7.07E-02 | 1.79E-01 | embryonic cranial skeleton morphogenesis |
| 10817 | 7.33E-02 | 1.79E-01 | regulation of hormone levels |
| 50864 | 7.35E-02 | 1.79E-01 | regulation of B cell activation |
| 2819 | 7.35E-02 | 1.79E-01 | regulation of adaptive immune response |
| 55074 | 7.41E-02 | 1.79E-01 | calcium ion homeostasis |
| 8216 | 7.43E-02 | 1.79E-01 | spermidine metabolic process |
| 16557 | 7.43E-02 | 1.79E-01 | peroxisome membrane biogenesis |
| 288 | 7.43E-02 | 1.79E-01 | nuclear-transcribed mRNA catabolic process, deadenylation-dependent decay |
| 70096 | 7.43E-02 | 1.79E-01 | mitochondrial outer membrane translocase complex assembly |
| 33240 | 7.43E-02 | 1.79E-01 | positive regulation of cellular amine metabolic process |
| 33539 | 7.43E-02 | 1.79E-01 | fatty acid beta-oxidation using acyl-CoA dehydrogenase |
| 9129 | 7.43E-02 | 1.79E-01 | pyrimidine nucleoside monophosphate metabolic process |
| 9130 | 7.43E-02 | 1.79E-01 | pyrimidine nucleoside monophosphate biosynthetic process |
| 46125 | 7.43E-02 | 1.79E-01 | pyrimidine deoxyribonucleoside metabolic process |
| 33860 | 7.43E-02 | 1.79E-01 | regulation of NAD(P)H oxidase activity |
| 70874 | 7.43E-02 | 1.79E-01 | negative regulation of glycogen metabolic process |
| 46322 | 7.43E-02 | 1.79E-01 | negative regulation of fatty acid oxidation |
| 46477 | 7.43E-02 | 1.79E-01 | glycosylceramide catabolic process |
| 42420 | 7.43E-02 | 1.79E-01 | dopamine catabolic process |
| 1561 | 7.43E-02 | 1.79E-01 | fatty acid alpha-oxidation |
| 71277 | 7.43E-02 | 1.79E-01 | cellular response to calcium ion |
| 42661 | 7.43E-02 | 1.79E-01 | regulation of mesodermal cell fate specification |
| 18208 | 7.43E-02 | 1.79E-01 | peptidyl-proline modification |
| 1956 | 7.43E-02 | 1.79E-01 | positive regulation of neurotransmitter secretion |
| 6166 | 7.43E-02 | 1.79E-01 | purine ribonucleoside salvage |
| 6207 | 7.43E-02 | 1.79E-01 | 'de novo' pyrimidine base biosynthetic process |
| 2118 | 7.43E-02 | 1.79E-01 | aggressive behavior |
| 43174 | 7.43E-02 | 1.79E-01 | nucleoside salvage |
| 51409 | 7.43E-02 | 1.79E-01 | response to nitrosative stress |
| 10613 | 7.43E-02 | 1.79E-01 | positive regulation of cardiac muscle hypertrophy |
| 6531 | 7.43E-02 | 1.79E-01 | aspartate metabolic process |
| 6533 | 7.43E-02 | 1.79E-01 | aspartate catabolic process |
| 10640 | 7.43E-02 | 1.79E-01 | regulation of platelet-derived growth factor receptor signaling pathway |
| 6545 | 7.43E-02 | 1.79E-01 | glycine biosynthetic process |
| 6549 | 7.43E-02 | 1.79E-01 | isoleucine metabolic process |
| 14742 | 7.43E-02 | 1.79E-01 | positive regulation of muscle hypertrophy |
| 6681 | 7.43E-02 | 1.79E-01 | galactosylceramide metabolic process |
| 6686 | 7.43E-02 | 1.79E-01 | sphingomyelin biosynthetic process |
| 10799 | 7.43E-02 | 1.79E-01 | regulation of peptidyl-threonine phosphorylation |
| 6704 | 7.43E-02 | 1.79E-01 | glucocorticoid biosynthetic process |
| 43619 | 7.43E-02 | 1.79E-01 | regulation of transcription from RNA polymerase II promoter in response to oxidative stress |
| 60009 | 7.43E-02 | 1.79E-01 | Sertoli cell development |
| 2701 | 7.43E-02 | 1.79E-01 | negative regulation of production of molecular mediator of immune response |
| 2719 | 7.43E-02 | 1.79E-01 | negative regulation of cytokine production involved in immune response |
| 6883 | 7.43E-02 | 1.79E-01 | cellular sodium ion homeostasis |
| 7008 | 7.43E-02 | 1.79E-01 | outer mitochondrial membrane organization |
| 2921 | 7.43E-02 | 1.79E-01 | negative regulation of humoral immune response |
| 7035 | 7.43E-02 | 1.79E-01 | vacuolar acidification |
| 60292 | 7.43E-02 | 1.79E-01 | long term synaptic depression |
| 31639 | 7.43E-02 | 1.79E-01 | plasminogen activation |
| 19372 | 7.43E-02 | 1.79E-01 | lipoxygenase pathway |
| 19374 | 7.43E-02 | 1.79E-01 | galactolipid metabolic process |
| 3079 | 7.43E-02 | 1.79E-01 | positive regulation of natriuresis |
| 48143 | 7.43E-02 | 1.79E-01 | astrocyte activation |
| 60456 | 7.43E-02 | 1.79E-01 | positive regulation of digestive system process |
| 44268 | 7.43E-02 | 1.79E-01 | multicellular organismal protein metabolic process |
| 60753 | 7.43E-02 | 1.79E-01 | regulation of mast cell chemotaxis |
| 60754 | 7.43E-02 | 1.79E-01 | positive regulation of mast cell chemotaxis |
| 19856 | 7.43E-02 | 1.79E-01 | pyrimidine base biosynthetic process |
| 15793 | 7.43E-02 | 1.79E-01 | glycerol transport |
| 15812 | 7.43E-02 | 1.79E-01 | gamma-aminobutyric acid transport |
| 15824 | 7.43E-02 | 1.79E-01 | proline transport |
| 7638 | 7.43E-02 | 1.79E-01 | mechanosensory behavior |
| 32222 | 7.43E-02 | 1.79E-01 | regulation of synaptic transmission, cholinergic |
| 16137 | 7.43E-02 | 1.79E-01 | glycoside metabolic process |
| 31349 | 7.44E-02 | 1.80E-01 | positive regulation of defense response |
| 48705 | 7.58E-02 | 1.83E-01 | skeletal system morphogenesis |
| 31401 | 7.60E-02 | 1.83E-01 | positive regulation of protein modification process |
| 17144 | 7.62E-02 | 1.83E-01 | drug metabolic process |
| 6213 | 7.62E-02 | 1.83E-01 | pyrimidine nucleoside metabolic process |
| 32024 | 7.62E-02 | 1.83E-01 | positive regulation of insulin secretion |
| 32570 | 7.62E-02 | 1.83E-01 | response to progesterone stimulus |
| 8629 | 7.70E-02 | 1.84E-01 | induction of apoptosis by intracellular signals |
| 46519 | 7.70E-02 | 1.84E-01 | sphingoid metabolic process |
| 46883 | 7.70E-02 | 1.84E-01 | regulation of hormone secretion |
| 7188 | 7.97E-02 | 1.90E-01 | G-protein signaling, coupled to cAMP nucleotide second messenger |
| 48871 | 7.97E-02 | 1.90E-01 | multicellular organismal homeostasis |
| 43524 | 8.05E-02 | 1.92E-01 | negative regulation of neuron apoptosis |
| 45429 | 8.18E-02 | 1.94E-01 | positive regulation of nitric oxide biosynthetic process |
| 50873 | 8.18E-02 | 1.94E-01 | brown fat cell differentiation |
| 43255 | 8.18E-02 | 1.94E-01 | regulation of carbohydrate biosynthetic process |
| 10595 | 8.18E-02 | 1.94E-01 | positive regulation of endothelial cell migration |
| 43488 | 8.18E-02 | 1.94E-01 | regulation of mRNA stability |
| 2824 | 8.18E-02 | 1.94E-01 | positive regulation of adaptive immune response based on somatic recombination of immune receptors built from immunoglobulin superfamily domains |
| 7040 | 8.18E-02 | 1.94E-01 | lysosome organization |
| 7618 | 8.18E-02 | 1.94E-01 | mating |
| 50863 | 8.23E-02 | 1.95E-01 | regulation of T cell activation |
| 18193 | 8.27E-02 | 1.95E-01 | peptidyl-amino acid modification |
| 34599 | 8.41E-02 | 1.98E-01 | cellular response to oxidative stress |
| 44264 | 8.41E-02 | 1.98E-01 | cellular polysaccharide metabolic process |
| 30879 | 8.51E-02 | 2.00E-01 | mammary gland development |
| 50886 | 8.76E-02 | 2.04E-01 | endocrine process |
| 42743 | 8.76E-02 | 2.04E-01 | hydrogen peroxide metabolic process |
| 43271 | 8.76E-02 | 2.04E-01 | negative regulation of ion transport |
| 43487 | 8.76E-02 | 2.04E-01 | regulation of RNA stability |
| 10743 | 8.76E-02 | 2.04E-01 | regulation of macrophage derived foam cell differentiation |
| 6801 | 8.76E-02 | 2.04E-01 | superoxide metabolic process |
| 2821 | 8.76E-02 | 2.04E-01 | positive regulation of adaptive immune response |
| 31347 | 8.86E-02 | 2.04E-01 | regulation of defense response |
| 55066 | 9.00E-02 | 2.04E-01 | di-, tri-valent inorganic cation homeostasis |
| 10959 | 9.07E-02 | 2.04E-01 | regulation of metal ion transport |
| 42440 | 9.14E-02 | 2.04E-01 | pigment metabolic process |
| 32526 | 9.14E-02 | 2.04E-01 | response to retinoic acid |
| 40012 | 9.16E-02 | 2.04E-01 | regulation of locomotion |
| 32811 | 9.19E-02 | 2.04E-01 | negative regulation of epinephrine secretion |
| 90189 | 9.19E-02 | 2.04E-01 | regulation of branching involved in ureteric bud morphogenesis |
| 90190 | 9.19E-02 | 2.04E-01 | positive regulation of branching involved in ureteric bud morphogenesis |
| 32964 | 9.19E-02 | 2.04E-01 | collagen biosynthetic process |
| 70233 | 9.19E-02 | 2.04E-01 | negative regulation of T cell apoptosis |
| 45743 | 9.19E-02 | 2.04E-01 | positive regulation of fibroblast growth factor receptor signaling pathway |
| 70472 | 9.19E-02 | 2.04E-01 | regulation of uterine smooth muscle contraction |
| 70474 | 9.19E-02 | 2.04E-01 | positive regulation of uterine smooth muscle contraction |
| 45989 | 9.19E-02 | 2.04E-01 | positive regulation of striated muscle contraction |
| 42424 | 9.19E-02 | 2.04E-01 | catecholamine catabolic process |
| 34313 | 9.19E-02 | 2.04E-01 | diol catabolic process |
| 34339 | 9.19E-02 | 2.04E-01 | regulation of transcription from RNA polymerase II promoter by nuclear hormone receptor |
| 42532 | 9.19E-02 | 2.04E-01 | negative regulation of tyrosine phosphorylation of STAT protein |
| 42713 | 9.19E-02 | 2.04E-01 | sperm ejaculation |
| 71398 | 9.19E-02 | 2.04E-01 | cellular response to fatty acid |
| 30449 | 9.19E-02 | 2.04E-01 | regulation of complement activation |
| 46874 | 9.19E-02 | 2.04E-01 | quinolinate metabolic process |
| 5984 | 9.19E-02 | 2.04E-01 | disaccharide metabolic process |
| 1921 | 9.19E-02 | 2.04E-01 | positive regulation of receptor recycling |
| 1957 | 9.19E-02 | 2.04E-01 | intramembranous ossification |
| 6063 | 9.19E-02 | 2.04E-01 | uronic acid metabolic process |
| 1977 | 9.19E-02 | 2.04E-01 | renal system process involved in regulation of blood volume |
| 6102 | 9.19E-02 | 2.04E-01 | isocitrate metabolic process |
| 42984 | 9.19E-02 | 2.04E-01 | regulation of amyloid precursor protein biosynthetic process |
| 43101 | 9.19E-02 | 2.04E-01 | purine salvage |
| 51387 | 9.19E-02 | 2.04E-01 | negative regulation of nerve growth factor receptor signaling pathway |
| 2246 | 9.19E-02 | 2.04E-01 | wound healing involved in inflammatory response |
| 43267 | 9.19E-02 | 2.04E-01 | negative regulation of potassium ion transport |
| 6551 | 9.19E-02 | 2.04E-01 | leucine metabolic process |
| 6572 | 9.19E-02 | 2.04E-01 | tyrosine catabolic process |
| 10700 | 9.19E-02 | 2.04E-01 | negative regulation of norepinephrine secretion |
| 14850 | 9.19E-02 | 2.04E-01 | response to muscle activity |
| 14896 | 9.19E-02 | 2.04E-01 | muscle hypertrophy |
| 43587 | 9.19E-02 | 2.04E-01 | tongue morphogenesis |
| 2634 | 9.19E-02 | 2.04E-01 | regulation of germinal center formation |
| 43618 | 9.19E-02 | 2.04E-01 | regulation of transcription from RNA polymerase II promoter in response to stress |
| 10890 | 9.19E-02 | 2.04E-01 | positive regulation of sequestering of triglyceride |
| 60056 | 9.19E-02 | 2.04E-01 | mammary gland involution |
| 60073 | 9.19E-02 | 2.04E-01 | micturition |
| 31657 | 9.19E-02 | 2.04E-01 | regulation of cyclin-dependent protein kinase activity involved by G1/S |
| 31659 | 9.19E-02 | 2.04E-01 | positive regulation of cyclin-dependent protein kinase activity involved in G1/S |
| 7199 | 9.19E-02 | 2.04E-01 | G-protein signaling, coupled to cGMP nucleotide second messenger |
| 44091 | 9.19E-02 | 2.04E-01 | membrane biogenesis |
| 19585 | 9.19E-02 | 2.04E-01 | glucuronate metabolic process |
| 48266 | 9.19E-02 | 2.04E-01 | behavioral response to pain |
| 19614 | 9.19E-02 | 2.04E-01 | catechol catabolic process |
| 31953 | 9.19E-02 | 2.04E-01 | negative regulation of protein amino acid autophosphorylation |
| 15840 | 9.19E-02 | 2.04E-01 | urea transport |
| 60900 | 9.19E-02 | 2.04E-01 | embryonic camera-type eye formation |
| 45008 | 9.19E-02 | 2.04E-01 | depyrimidination |
| 45019 | 9.19E-02 | 2.04E-01 | negative regulation of nitric oxide biosynthetic process |
| 90080 | 9.19E-02 | 2.04E-01 | positive regulation of MAPKKK cascade by fibroblast growth factor receptor signaling pathway |
| 7398 | 9.30E-02 | 2.06E-01 | ectoderm development |
| 31399 | 9.31E-02 | 2.06E-01 | regulation of protein modification process |
| 22414 | 9.32E-02 | 2.06E-01 | reproductive process |
| 9109 | 9.34E-02 | 2.06E-01 | coenzyme catabolic process |
| 1101 | 9.34E-02 | 2.06E-01 | response to acid |
| 30199 | 9.34E-02 | 2.06E-01 | collagen fibril organization |
| 3044 | 9.34E-02 | 2.06E-01 | regulation of systemic arterial blood pressure mediated by a chemical signal |
| 45926 | 9.39E-02 | 2.07E-01 | negative regulation of growth |
| 7179 | 9.52E-02 | 2.09E-01 | transforming growth factor beta receptor signaling pathway |
| 9611 | 9.58E-02 | 2.10E-01 | response to wounding |
| 3 | 9.58E-02 | 2.10E-01 | reproduction |
| 43523 | 9.65E-02 | 2.12E-01 | regulation of neuron apoptosis |
| 45859 | 9.72E-02 | 2.13E-01 | regulation of protein kinase activity |
| 45598 | 9.94E-02 | 2.16E-01 | regulation of fat cell differentiation |
| 42168 | 9.94E-02 | 2.16E-01 | heme metabolic process |
| 22407 | 9.94E-02 | 2.16E-01 | regulation of cell-cell adhesion |
| 51353 | 9.94E-02 | 2.16E-01 | positive regulation of oxidoreductase activity |
| 10466 | 9.94E-02 | 2.16E-01 | negative regulation of peptidase activity |
| 6939 | 9.94E-02 | 2.16E-01 | smooth muscle contraction |
| 60688 | 9.94E-02 | 2.16E-01 | regulation of morphogenesis of a branching structure |
| 9887 | 1.01E-01 | 2.21E-01 | organ morphogenesis |
| 19933 | 1.02E-01 | 2.23E-01 | cAMP-mediated signaling |
| 40007 | 1.04E-01 | 2.26E-01 | growth |
| 44093 | 1.05E-01 | 2.26E-01 | positive regulation of molecular function |
| 32270 | 1.05E-01 | 2.26E-01 | positive regulation of cellular protein metabolic process |
| 32768 | 1.05E-01 | 2.26E-01 | regulation of monooxygenase activity |
| 90277 | 1.05E-01 | 2.26E-01 | positive regulation of peptide hormone secretion |
| 43547 | 1.05E-01 | 2.26E-01 | positive regulation of GTPase activity |
| 7006 | 1.05E-01 | 2.26E-01 | mitochondrial membrane organization |
| 31670 | 1.05E-01 | 2.26E-01 | cellular response to nutrient |
| 7270 | 1.05E-01 | 2.26E-01 | nerve-nerve synaptic transmission |
| 1657 | 1.07E-01 | 2.26E-01 | ureteric bud development |
| 70271 | 1.08E-01 | 2.26E-01 | protein complex biogenesis |
| 6461 | 1.08E-01 | 2.26E-01 | protein complex assembly |
| 48872 | 1.09E-01 | 2.26E-01 | homeostasis of number of cells |
| 70371 | 1.09E-01 | 2.26E-01 | ERK1 and ERK2 cascade |
| 9120 | 1.09E-01 | 2.26E-01 | deoxyribonucleoside metabolic process |
| 46033 | 1.09E-01 | 2.26E-01 | AMP metabolic process |
| 46426 | 1.09E-01 | 2.26E-01 | negative regulation of JAK-STAT cascade |
| 46514 | 1.09E-01 | 2.26E-01 | ceramide catabolic process |
| 34308 | 1.09E-01 | 2.26E-01 | monohydric alcohol metabolic process |
| 42537 | 1.09E-01 | 2.26E-01 | benzene and derivative metabolic process |
| 34379 | 1.09E-01 | 2.26E-01 | very-low-density lipoprotein particle assembly |
| 30432 | 1.09E-01 | 2.26E-01 | peristalsis |
| 14060 | 1.09E-01 | 2.26E-01 | regulation of epinephrine secretion |
| 55078 | 1.09E-01 | 2.26E-01 | sodium ion homeostasis |
| 1867 | 1.09E-01 | 2.26E-01 | complement activation, lectin pathway |
| 5980 | 1.09E-01 | 2.26E-01 | glycogen catabolic process |
| 42886 | 1.09E-01 | 2.26E-01 | amide transport |
| 6067 | 1.09E-01 | 2.26E-01 | ethanol metabolic process |
| 30643 | 1.09E-01 | 2.26E-01 | cellular phosphate ion homeostasis |
| 6069 | 1.09E-01 | 2.26E-01 | ethanol oxidation |
| 2063 | 1.09E-01 | 2.26E-01 | chondrocyte development |
| 6171 | 1.09E-01 | 2.26E-01 | cAMP biosynthetic process |
| 51386 | 1.09E-01 | 2.26E-01 | regulation of nerve growth factor receptor signaling pathway |
| 10560 | 1.09E-01 | 2.26E-01 | positive regulation of glycoprotein biosynthetic process |
| 6467 | 1.09E-01 | 2.26E-01 | protein thiol-disulfide exchange |
| 51580 | 1.09E-01 | 2.26E-01 | regulation of neurotransmitter uptake |
| 51584 | 1.09E-01 | 2.26E-01 | regulation of dopamine uptake |
| 51590 | 1.09E-01 | 2.26E-01 | positive regulation of neurotransmitter transport |
| 51593 | 1.09E-01 | 2.26E-01 | response to folic acid |
| 2532 | 1.09E-01 | 2.26E-01 | production of molecular mediator involved in inflammatory response |
| 43620 | 1.09E-01 | 2.26E-01 | regulation of transcription in response to stress |
| 60008 | 1.09E-01 | 2.26E-01 | Sertoli cell differentiation |
| 10886 | 1.09E-01 | 2.26E-01 | positive regulation of cholesterol storage |
| 10955 | 1.09E-01 | 2.26E-01 | negative regulation of protein maturation by peptide bond cleavage |
| 51940 | 1.09E-01 | 2.26E-01 | regulation of catecholamine uptake involved in synaptic transmission |
| 51962 | 1.09E-01 | 2.26E-01 | positive regulation of nervous system development |
| 51965 | 1.09E-01 | 2.26E-01 | positive regulation of synaptogenesis |
| 51967 | 1.09E-01 | 2.26E-01 | negative regulation of synaptic transmission, glutamatergic |
| 60351 | 1.09E-01 | 2.26E-01 | cartilage development involved in endochondral bone morphogenesis |
| 44089 | 1.09E-01 | 2.26E-01 | positive regulation of cellular component biogenesis |
| 60665 | 1.09E-01 | 2.26E-01 | regulation of branching involved in salivary gland morphogenesis by mesenchymal-epithelial signaling |
| 32230 | 1.09E-01 | 2.26E-01 | positive regulation of synaptic transmission, GABAergic |
| 48739 | 1.09E-01 | 2.26E-01 | cardiac muscle fiber development |
| 32469 | 1.09E-01 | 2.26E-01 | endoplasmic reticulum calcium ion homeostasis |
| 16090 | 1.09E-01 | 2.26E-01 | prenol metabolic process |
| 16093 | 1.09E-01 | 2.26E-01 | polyprenol metabolic process |
| 19748 | 1.11E-01 | 2.30E-01 | secondary metabolic process |
| 6816 | 1.11E-01 | 2.30E-01 | calcium ion transport |
| 18105 | 1.12E-01 | 2.30E-01 | peptidyl-serine phosphorylation |
| 10522 | 1.12E-01 | 2.30E-01 | regulation of calcium ion transport into cytosol |
| 6664 | 1.12E-01 | 2.30E-01 | glycolipid metabolic process |
| 2793 | 1.12E-01 | 2.30E-01 | positive regulation of peptide secretion |
| 45333 | 1.12E-01 | 2.30E-01 | cellular respiration |
| 1558 | 1.12E-01 | 2.31E-01 | regulation of cell growth |
| 48609 | 1.13E-01 | 2.31E-01 | reproductive process in a multicellular organism |
| 32504 | 1.13E-01 | 2.31E-01 | multicellular organism reproduction |
| 46903 | 1.13E-01 | 2.32E-01 | secretion |
| 51047 | 1.14E-01 | 2.34E-01 | positive regulation of secretion |
| 65009 | 1.15E-01 | 2.35E-01 | regulation of molecular function |
| 50900 | 1.15E-01 | 2.36E-01 | leukocyte migration |
| 43549 | 1.17E-01 | 2.40E-01 | regulation of kinase activity |
| 46620 | 1.18E-01 | 2.41E-01 | regulation of organ growth |
| 46888 | 1.18E-01 | 2.41E-01 | negative regulation of hormone secretion |
| 51239 | 1.19E-01 | 2.43E-01 | regulation of multicellular organismal process |
| 31400 | 1.22E-01 | 2.49E-01 | negative regulation of protein modification process |
| 40017 | 1.22E-01 | 2.49E-01 | positive regulation of locomotion |
| 10553 | 1.23E-01 | 2.50E-01 | negative regulation of gene-specific transcription from RNA polymerase II promoter |
| 48146 | 1.24E-01 | 2.50E-01 | positive regulation of fibroblast proliferation |
| 61138 | 1.25E-01 | 2.50E-01 | morphogenesis of a branching epithelium |
| 33137 | 1.26E-01 | 2.50E-01 | negative regulation of peptidyl-serine phosphorylation |
| 33194 | 1.26E-01 | 2.50E-01 | response to hydroperoxide |
| 70232 | 1.26E-01 | 2.50E-01 | regulation of T cell apoptosis |
| 33604 | 1.26E-01 | 2.50E-01 | negative regulation of catecholamine secretion |
| 9223 | 1.26E-01 | 2.50E-01 | pyrimidine deoxyribonucleotide catabolic process |
| 9251 | 1.26E-01 | 2.50E-01 | glucan catabolic process |
| 70723 | 1.26E-01 | 2.50E-01 | response to cholesterol |
| 9404 | 1.26E-01 | 2.50E-01 | toxin metabolic process |
| 46521 | 1.26E-01 | 2.50E-01 | sphingoid catabolic process |
| 46622 | 1.26E-01 | 2.50E-01 | positive regulation of organ growth |
| 1573 | 1.26E-01 | 2.50E-01 | ganglioside metabolic process |
| 34372 | 1.26E-01 | 2.50E-01 | very-low-density lipoprotein particle remodeling |
| 34383 | 1.26E-01 | 2.50E-01 | low-density lipoprotein particle clearance |
| 9820 | 1.26E-01 | 2.50E-01 | alkaloid metabolic process |
| 30319 | 1.26E-01 | 2.50E-01 | cellular di-, tri-valent inorganic anion homeostasis |
| 71396 | 1.26E-01 | 2.50E-01 | cellular response to lipid |
| 55062 | 1.26E-01 | 2.50E-01 | phosphate ion homeostasis |
| 1967 | 1.26E-01 | 2.50E-01 | suckling behavior |
| 51131 | 1.26E-01 | 2.50E-01 | chaperone-mediated protein complex assembly |
| 51145 | 1.26E-01 | 2.50E-01 | smooth muscle cell differentiation |
| 6101 | 1.26E-01 | 2.50E-01 | citrate metabolic process |
| 31116 | 1.26E-01 | 2.50E-01 | positive regulation of microtubule polymerization |
| 51775 | 1.26E-01 | 2.50E-01 | response to redox state |
| 10873 | 1.26E-01 | 2.50E-01 | positive regulation of cholesterol esterification |
| 2686 | 1.26E-01 | 2.50E-01 | negative regulation of leukocyte migration |
| 6977 | 1.26E-01 | 2.50E-01 | DNA damage response, signal transduction by p53 class mediator resulting in cell cycle arrest |
| 31579 | 1.26E-01 | 2.50E-01 | membrane raft organization |
| 3016 | 1.26E-01 | 2.50E-01 | respiratory system process |
| 44247 | 1.26E-01 | 2.50E-01 | cellular polysaccharide catabolic process |
| 32042 | 1.26E-01 | 2.50E-01 | mitochondrial DNA metabolic process |
| 15791 | 1.26E-01 | 2.50E-01 | polyol transport |
| 15838 | 1.26E-01 | 2.50E-01 | betaine transport |
| 32229 | 1.26E-01 | 2.50E-01 | negative regulation of synaptic transmission, GABAergic |
| 15879 | 1.26E-01 | 2.50E-01 | carnitine transport |
| 9888 | 1.27E-01 | 2.51E-01 | tissue development |
| 7420 | 1.27E-01 | 2.51E-01 | brain development |
| 7631 | 1.28E-01 | 2.52E-01 | feeding behavior |
| 48583 | 1.30E-01 | 2.56E-01 | regulation of response to stimulus |
| 70838 | 1.30E-01 | 2.56E-01 | divalent metal ion transport |
| 8306 | 1.31E-01 | 2.56E-01 | associative learning |
| 8543 | 1.31E-01 | 2.56E-01 | fibroblast growth factor receptor signaling pathway |
| 43467 | 1.31E-01 | 2.56E-01 | regulation of generation of precursor metabolites and energy |
| 51705 | 1.31E-01 | 2.56E-01 | behavioral interaction between organisms |
| 44259 | 1.31E-01 | 2.56E-01 | multicellular organismal macromolecule metabolic process |
| 30198 | 1.35E-01 | 2.64E-01 | extracellular matrix organization |
| 52547 | 1.35E-01 | 2.64E-01 | regulation of peptidase activity |
| 51247 | 1.35E-01 | 2.64E-01 | positive regulation of protein metabolic process |
| 1655 | 1.36E-01 | 2.66E-01 | urogenital system development |
| 6955 | 1.36E-01 | 2.66E-01 | immune response |
| 9060 | 1.37E-01 | 2.67E-01 | aerobic respiration |
| 42439 | 1.37E-01 | 2.67E-01 | ethanolamine and derivative metabolic process |
| 51899 | 1.37E-01 | 2.67E-01 | membrane depolarization |
| 19233 | 1.37E-01 | 2.67E-01 | sensory perception of pain |
| 30005 | 1.40E-01 | 2.68E-01 | cellular di-, tri-valent inorganic cation homeostasis |
| 33189 | 1.41E-01 | 2.68E-01 | response to vitamin A |
| 1505 | 1.41E-01 | 2.68E-01 | regulation of neurotransmitter levels |
| 7166 | 1.41E-01 | 2.68E-01 | cell surface receptor linked signaling pathway |
| 2376 | 1.41E-01 | 2.68E-01 | immune system process |
| 51338 | 1.42E-01 | 2.68E-01 | regulation of transferase activity |
| 48584 | 1.42E-01 | 2.68E-01 | positive regulation of response to stimulus |
| 7417 | 1.43E-01 | 2.68E-01 | central nervous system development |
| 8211 | 1.43E-01 | 2.68E-01 | glucocorticoid metabolic process |
| 45080 | 1.43E-01 | 2.68E-01 | positive regulation of chemokine biosynthetic process |
| 90183 | 1.43E-01 | 2.68E-01 | regulation of kidney development |
| 33002 | 1.43E-01 | 2.68E-01 | muscle cell proliferation |
| 8653 | 1.43E-01 | 2.68E-01 | lipopolysaccharide metabolic process |
| 33483 | 1.43E-01 | 2.68E-01 | gas homeostasis |
| 9103 | 1.43E-01 | 2.68E-01 | lipopolysaccharide biosynthetic process |
| 9113 | 1.43E-01 | 2.68E-01 | purine base biosynthetic process |
| 9164 | 1.43E-01 | 2.68E-01 | nucleoside catabolic process |
| 42136 | 1.43E-01 | 2.68E-01 | neurotransmitter biosynthetic process |
| 9435 | 1.43E-01 | 2.68E-01 | NAD biosynthetic process |
| 34285 | 1.43E-01 | 2.68E-01 | response to disaccharide stimulus |
| 71158 | 1.43E-01 | 2.68E-01 | positive regulation of cell cycle arrest |
| 9744 | 1.43E-01 | 2.68E-01 | response to sucrose stimulus |
| 50732 | 1.43E-01 | 2.68E-01 | negative regulation of peptidyl-tyrosine phosphorylation |
| 42574 | 1.43E-01 | 2.68E-01 | retinal metabolic process |
| 14002 | 1.43E-01 | 2.68E-01 | astrocyte development |
| 42756 | 1.43E-01 | 2.68E-01 | drinking behavior |
| 55061 | 1.43E-01 | 2.68E-01 | di-, tri-valent inorganic anion homeostasis |
| 1991 | 1.43E-01 | 2.68E-01 | regulation of systemic arterial blood pressure by circulatory renin-angiotensin |
| 6108 | 1.43E-01 | 2.68E-01 | malate metabolic process |
| 2016 | 1.43E-01 | 2.68E-01 | regulation of blood volume by renin-angiotensin |
| 43092 | 1.43E-01 | 2.68E-01 | L-amino acid import |
| 43094 | 1.43E-01 | 2.68E-01 | cellular metabolic compound salvage |
| 6244 | 1.43E-01 | 2.68E-01 | pyrimidine nucleotide catabolic process |
| 6285 | 1.43E-01 | 2.68E-01 | base-excision repair, AP site formation |
| 10470 | 1.43E-01 | 2.68E-01 | regulation of gastrulation |
| 35065 | 1.43E-01 | 2.68E-01 | regulation of histone acetylation |
| 43288 | 1.43E-01 | 2.68E-01 | apocarotenoid metabolic process |
| 6491 | 1.43E-01 | 2.68E-01 | N-glycan processing |
| 6677 | 1.43E-01 | 2.68E-01 | glycosylceramide metabolic process |
| 10872 | 1.43E-01 | 2.68E-01 | regulation of cholesterol esterification |
| 60045 | 1.43E-01 | 2.68E-01 | positive regulation of cardiac muscle cell proliferation |
| 51900 | 1.43E-01 | 2.68E-01 | regulation of mitochondrial depolarization |
| 19433 | 1.43E-01 | 2.68E-01 | triglyceride catabolic process |
| 48148 | 1.43E-01 | 2.68E-01 | behavioral response to cocaine |
| 7217 | 1.43E-01 | 2.68E-01 | tachykinin receptor signaling pathway |
| 60638 | 1.43E-01 | 2.68E-01 | mesenchymal-epithelial cell signaling |
| 48569 | 1.43E-01 | 2.68E-01 | post-embryonic organ development |
| 48703 | 1.43E-01 | 2.68E-01 | embryonic viscerocranium morphogenesis |
| 48857 | 1.43E-01 | 2.68E-01 | neural nucleus development |
| 32490 | 1.43E-01 | 2.68E-01 | detection of molecule of bacterial origin |
| 6487 | 1.44E-01 | 2.69E-01 | protein amino acid N-linked glycosylation |
| 43269 | 1.45E-01 | 2.71E-01 | regulation of ion transport |
| 45137 | 1.48E-01 | 2.76E-01 | development of primary sexual characteristics |
| 5977 | 1.50E-01 | 2.80E-01 | glycogen metabolic process |
| 31214 | 1.50E-01 | 2.80E-01 | biomineral formation |
| 6970 | 1.50E-01 | 2.80E-01 | response to osmotic stress |
| 7626 | 1.53E-01 | 2.85E-01 | locomotory behavior |
| 33013 | 1.57E-01 | 2.86E-01 | tetrapyrrole metabolic process |
| 9410 | 1.57E-01 | 2.86E-01 | response to xenobiotic stimulus |
| 6073 | 1.57E-01 | 2.86E-01 | cellular glucan metabolic process |
| 43112 | 1.57E-01 | 2.86E-01 | receptor metabolic process |
| 6778 | 1.57E-01 | 2.86E-01 | porphyrin metabolic process |
| 10906 | 1.57E-01 | 2.86E-01 | regulation of glucose metabolic process |
| 51928 | 1.57E-01 | 2.86E-01 | positive regulation of calcium ion transport |
| 44042 | 1.57E-01 | 2.86E-01 | glucan metabolic process |
| 30100 | 1.59E-01 | 2.86E-01 | regulation of endocytosis |
| 90263 | 1.59E-01 | 2.86E-01 | positive regulation of canonical Wnt receptor signaling pathway |
| 8354 | 1.59E-01 | 2.86E-01 | germ cell migration |
| 33006 | 1.59E-01 | 2.86E-01 | regulation of mast cell activation involved in immune response |
| 45540 | 1.59E-01 | 2.86E-01 | regulation of cholesterol biosynthetic process |
| 70207 | 1.59E-01 | 2.86E-01 | protein homotrimerization |
| 70229 | 1.59E-01 | 2.86E-01 | negative regulation of lymphocyte apoptosis |
| 9203 | 1.59E-01 | 2.86E-01 | ribonucleoside triphosphate catabolic process |
| 9207 | 1.59E-01 | 2.86E-01 | purine ribonucleoside triphosphate catabolic process |
| 42026 | 1.59E-01 | 2.86E-01 | protein refolding |
| 42053 | 1.59E-01 | 2.86E-01 | regulation of dopamine metabolic process |
| 21756 | 1.59E-01 | 2.86E-01 | striatum development |
| 46461 | 1.59E-01 | 2.86E-01 | neutral lipid catabolic process |
| 46464 | 1.59E-01 | 2.86E-01 | acylglycerol catabolic process |
| 34370 | 1.59E-01 | 2.86E-01 | triglyceride-rich lipoprotein particle remodeling |
| 34374 | 1.59E-01 | 2.86E-01 | low-density lipoprotein particle remodeling |
| 18065 | 1.59E-01 | 2.86E-01 | protein-cofactor linkage |
| 30431 | 1.59E-01 | 2.86E-01 | sleep |
| 1782 | 1.59E-01 | 2.86E-01 | B cell homeostasis |
| 1919 | 1.59E-01 | 2.86E-01 | regulation of receptor recycling |
| 1963 | 1.59E-01 | 2.86E-01 | synaptic transmission, dopaminergic |
| 1976 | 1.59E-01 | 2.86E-01 | neurological system process involved in regulation of systemic arterial blood pressure |
| 42953 | 1.59E-01 | 2.86E-01 | lipoprotein transport |
| 42987 | 1.59E-01 | 2.86E-01 | amyloid precursor protein catabolic process |
| 43011 | 1.59E-01 | 2.86E-01 | myeloid dendritic cell differentiation |
| 10288 | 1.59E-01 | 2.86E-01 | response to lead ion |
| 6206 | 1.59E-01 | 2.86E-01 | pyrimidine base metabolic process |
| 43129 | 1.59E-01 | 2.86E-01 | surfactant homeostasis |
| 30857 | 1.59E-01 | 2.86E-01 | negative regulation of epithelial cell differentiation |
| 43304 | 1.59E-01 | 2.86E-01 | regulation of mast cell degranulation |
| 10611 | 1.59E-01 | 2.86E-01 | regulation of cardiac muscle hypertrophy |
| 31112 | 1.59E-01 | 2.86E-01 | positive regulation of microtubule polymerization or depolymerization |
| 10889 | 1.59E-01 | 2.86E-01 | regulation of sequestering of triglyceride |
| 31638 | 1.59E-01 | 2.86E-01 | zymogen activation |
| 40020 | 1.59E-01 | 2.86E-01 | regulation of meiosis |
| 3254 | 1.59E-01 | 2.86E-01 | regulation of membrane depolarization |
| 44269 | 1.59E-01 | 2.86E-01 | glycerol ether catabolic process |
| 7625 | 1.59E-01 | 2.86E-01 | grooming behavior |
| 15988 | 1.59E-01 | 2.86E-01 | energy coupled proton transport, against electrochemical gradient |
| 15991 | 1.59E-01 | 2.86E-01 | ATP hydrolysis coupled proton transport |
| 48875 | 1.59E-01 | 2.86E-01 | chemical homeostasis within a tissue |
| 45807 | 1.64E-01 | 2.94E-01 | positive regulation of endocytosis |
| 3073 | 1.64E-01 | 2.94E-01 | regulation of systemic arterial blood pressure |
| 50877 | 1.64E-01 | 2.94E-01 | neurological system process |
| 48732 | 1.65E-01 | 2.95E-01 | gland development |
| 1525 | 1.66E-01 | 2.97E-01 | angiogenesis |
| 43405 | 1.66E-01 | 2.97E-01 | regulation of MAP kinase activity |
| 30193 | 1.71E-01 | 3.03E-01 | regulation of blood coagulation |
| 30301 | 1.71E-01 | 3.03E-01 | cholesterol transport |
| 15918 | 1.71E-01 | 3.03E-01 | sterol transport |
| 23056 | 1.71E-01 | 3.03E-01 | positive regulation of signaling process |
| 6936 | 1.72E-01 | 3.03E-01 | muscle contraction |
| 32869 | 1.73E-01 | 3.03E-01 | cellular response to insulin stimulus |
| 7498 | 1.73E-01 | 3.03E-01 | mesoderm development |
| 2 | 1.75E-01 | 3.03E-01 | mitochondrial genome maintenance |
| 16558 | 1.75E-01 | 3.03E-01 | protein import into peroxisome matrix |
| 9077 | 1.75E-01 | 3.03E-01 | histidine family amino acid catabolic process |
| 70613 | 1.75E-01 | 3.03E-01 | regulation of protein processing |
| 42069 | 1.75E-01 | 3.03E-01 | regulation of catecholamine metabolic process |
| 42088 | 1.75E-01 | 3.03E-01 | T-helper 1 type immune response |
| 71241 | 1.75E-01 | 3.03E-01 | cellular response to inorganic substance |
| 71248 | 1.75E-01 | 3.03E-01 | cellular response to metal ion |
| 42659 | 1.75E-01 | 3.03E-01 | regulation of cell fate specification |
| 50853 | 1.75E-01 | 3.03E-01 | B cell receptor signaling pathway |
| 50908 | 1.75E-01 | 3.03E-01 | detection of light stimulus involved in visual perception |
| 1773 | 1.75E-01 | 3.03E-01 | myeloid dendritic cell activation |
| 1774 | 1.75E-01 | 3.03E-01 | microglial cell activation |
| 14065 | 1.75E-01 | 3.03E-01 | phosphoinositide 3-kinase cascade |
| 14074 | 1.75E-01 | 3.03E-01 | response to purine |
| 50962 | 1.75E-01 | 3.03E-01 | detection of light stimulus involved in sensory perception |
| 55093 | 1.75E-01 | 3.03E-01 | response to hyperoxia |
| 51000 | 1.75E-01 | 3.03E-01 | positive regulation of nitric-oxide synthase activity |
| 55117 | 1.75E-01 | 3.03E-01 | regulation of cardiac muscle contraction |
| 1935 | 1.75E-01 | 3.03E-01 | endothelial cell proliferation |
| 10453 | 1.75E-01 | 3.03E-01 | regulation of cell fate commitment |
| 43300 | 1.75E-01 | 3.03E-01 | regulation of leukocyte degranulation |
| 31057 | 1.75E-01 | 3.03E-01 | negative regulation of histone modification |
| 31113 | 1.75E-01 | 3.03E-01 | regulation of microtubule polymerization |
| 10634 | 1.75E-01 | 3.03E-01 | positive regulation of epithelial cell migration |
| 6548 | 1.75E-01 | 3.03E-01 | histidine catabolic process |
| 14743 | 1.75E-01 | 3.03E-01 | regulation of muscle hypertrophy |
| 43584 | 1.75E-01 | 3.03E-01 | nose development |
| 6740 | 1.75E-01 | 3.03E-01 | NADPH regeneration |
| 10953 | 1.75E-01 | 3.03E-01 | regulation of protein maturation by peptide bond cleavage |
| 2828 | 1.75E-01 | 3.03E-01 | regulation of T-helper 2 type immune response |
| 60343 | 1.75E-01 | 3.03E-01 | trabecula formation |
| 19432 | 1.75E-01 | 3.03E-01 | triglyceride biosynthetic process |
| 48200 | 1.75E-01 | 3.03E-01 | Golgi transport vesicle coating |
| 48205 | 1.75E-01 | 3.03E-01 | COPI coating of Golgi vesicle |
| 31952 | 1.75E-01 | 3.03E-01 | regulation of protein amino acid autophosphorylation |
| 60693 | 1.75E-01 | 3.03E-01 | regulation of branching involved in salivary gland morphogenesis |
| 32094 | 1.75E-01 | 3.03E-01 | response to food |
| 15936 | 1.75E-01 | 3.03E-01 | coenzyme A metabolic process |
| 48745 | 1.75E-01 | 3.03E-01 | smooth muscle tissue development |
| 32735 | 1.75E-01 | 3.03E-01 | positive regulation of interleukin-12 production |
| 45833 | 1.77E-01 | 3.05E-01 | negative regulation of lipid metabolic process |
| 18209 | 1.77E-01 | 3.05E-01 | peptidyl-serine modification |
| 2685 | 1.77E-01 | 3.05E-01 | regulation of leukocyte migration |
| 7193 | 1.77E-01 | 3.05E-01 | inhibition of adenylate cyclase activity by G-protein signaling pathway |
| 7422 | 1.77E-01 | 3.05E-01 | peripheral nervous system development |
| 31669 | 1.77E-01 | 3.05E-01 | cellular response to nutrient levels |
| 48469 | 1.77E-01 | 3.05E-01 | cell maturation |
| 51924 | 1.82E-01 | 3.13E-01 | regulation of calcium ion transport |
| 50921 | 1.84E-01 | 3.16E-01 | positive regulation of chemotaxis |
| 10578 | 1.84E-01 | 3.16E-01 | regulation of adenylate cyclase activity involved in G-protein signaling pathway |
| 10579 | 1.84E-01 | 3.16E-01 | positive regulation of adenylate cyclase activity by G-protein signaling pathway |
| 43393 | 1.84E-01 | 3.16E-01 | regulation of protein binding |
| 7189 | 1.84E-01 | 3.16E-01 | activation of adenylate cyclase activity by G-protein signaling pathway |
| 1501 | 1.86E-01 | 3.18E-01 | skeletal system development |
| 9743 | 1.87E-01 | 3.20E-01 | response to carbohydrate stimulus |
| 51100 | 1.87E-01 | 3.20E-01 | negative regulation of binding |
| 35239 | 1.87E-01 | 3.20E-01 | tube morphogenesis |
| 8285 | 1.90E-01 | 3.20E-01 | negative regulation of cell proliferation |
| 55067 | 1.91E-01 | 3.20E-01 | monovalent inorganic cation homeostasis |
| 30595 | 1.91E-01 | 3.20E-01 | leukocyte chemotaxis |
| 10594 | 1.91E-01 | 3.20E-01 | regulation of endothelial cell migration |
| 6641 | 1.91E-01 | 3.20E-01 | triglyceride metabolic process |
| 32769 | 1.91E-01 | 3.20E-01 | negative regulation of monooxygenase activity |
| 45073 | 1.91E-01 | 3.20E-01 | regulation of chemokine biosynthetic process |
| 305 | 1.91E-01 | 3.20E-01 | response to oxygen radical |
| 45723 | 1.91E-01 | 3.20E-01 | positive regulation of fatty acid biosynthetic process |
| 45912 | 1.91E-01 | 3.20E-01 | negative regulation of carbohydrate metabolic process |
| 9146 | 1.91E-01 | 3.20E-01 | purine nucleoside triphosphate catabolic process |
| 21544 | 1.91E-01 | 3.20E-01 | subpallium development |
| 71363 | 1.91E-01 | 3.20E-01 | cellular response to growth factor stimulus |
| 1885 | 1.91E-01 | 3.20E-01 | endothelial cell development |
| 1960 | 1.91E-01 | 3.20E-01 | negative regulation of cytokine-mediated signaling pathway |
| 6107 | 1.91E-01 | 3.20E-01 | oxaloacetate metabolic process |
| 42982 | 1.91E-01 | 3.20E-01 | amyloid precursor protein metabolic process |
| 43030 | 1.91E-01 | 3.20E-01 | regulation of macrophage activation |
| 51281 | 1.91E-01 | 3.20E-01 | positive regulation of release of sequestered calcium ion into cytosol |
| 51445 | 1.91E-01 | 3.20E-01 | regulation of meiotic cell cycle |
| 10677 | 1.91E-01 | 3.20E-01 | negative regulation of cellular carbohydrate metabolic process |
| 2886 | 1.91E-01 | 3.20E-01 | regulation of myeloid leukocyte mediated immunity |
| 19369 | 1.91E-01 | 3.20E-01 | arachidonic acid metabolic process |
| 48194 | 1.91E-01 | 3.20E-01 | Golgi vesicle budding |
| 7320 | 1.91E-01 | 3.20E-01 | insemination |
| 15697 | 1.91E-01 | 3.20E-01 | quaternary ammonium group transport |
| 15893 | 1.91E-01 | 3.20E-01 | drug transport |
| 48708 | 1.91E-01 | 3.20E-01 | astrocyte differentiation |
| 43062 | 1.92E-01 | 3.21E-01 | extracellular structure organization |
| 6665 | 1.92E-01 | 3.21E-01 | sphingolipid metabolic process |
| 50818 | 1.98E-01 | 3.31E-01 | regulation of coagulation |
| 48145 | 1.98E-01 | 3.31E-01 | regulation of fibroblast proliferation |
| 61041 | 1.98E-01 | 3.31E-01 | regulation of wound healing |
| 90068 | 1.98E-01 | 3.31E-01 | positive regulation of cell cycle process |
| 1763 | 2.01E-01 | 3.35E-01 | morphogenesis of a branching structure |
| 60627 | 2.01E-01 | 3.35E-01 | regulation of vesicle-mediated transport |
| 51605 | 2.02E-01 | 3.35E-01 | protein maturation by peptide bond cleavage |
| 2009 | 2.02E-01 | 3.35E-01 | morphogenesis of an epithelium |
| 7565 | 2.05E-01 | 3.35E-01 | female pregnancy |
| 50679 | 2.05E-01 | 3.35E-01 | positive regulation of epithelial cell proliferation |
| 42632 | 2.05E-01 | 3.35E-01 | cholesterol homeostasis |
| 1818 | 2.05E-01 | 3.35E-01 | negative regulation of cytokine production |
| 55092 | 2.05E-01 | 3.35E-01 | sterol homeostasis |
| 7033 | 2.05E-01 | 3.35E-01 | vacuole organization |
| 48706 | 2.07E-01 | 3.35E-01 | embryonic skeletal system development |
| 32967 | 2.07E-01 | 3.35E-01 | positive regulation of collagen biosynthetic process |
| 45616 | 2.07E-01 | 3.35E-01 | regulation of keratinocyte differentiation |
| 45736 | 2.07E-01 | 3.35E-01 | negative regulation of cyclin-dependent protein kinase activity |
| 45737 | 2.07E-01 | 3.35E-01 | positive regulation of cyclin-dependent protein kinase activity |
| 45742 | 2.07E-01 | 3.35E-01 | positive regulation of epidermal growth factor receptor signaling pathway |
| 45822 | 2.07E-01 | 3.35E-01 | negative regulation of heart contraction |
| 45885 | 2.07E-01 | 3.35E-01 | positive regulation of survival gene product expression |
| 9071 | 2.07E-01 | 3.35E-01 | serine family amino acid catabolic process |
| 9075 | 2.07E-01 | 3.35E-01 | histidine family amino acid metabolic process |
| 46039 | 2.07E-01 | 3.35E-01 | GTP metabolic process |
| 9219 | 2.07E-01 | 3.35E-01 | pyrimidine deoxyribonucleotide metabolic process |
| 42310 | 2.07E-01 | 3.35E-01 | vasoconstriction |
| 46460 | 2.07E-01 | 3.35E-01 | neutral lipid biosynthetic process |
| 46463 | 2.07E-01 | 3.35E-01 | acylglycerol biosynthetic process |
| 46503 | 2.07E-01 | 3.35E-01 | glycerolipid catabolic process |
| 1556 | 2.07E-01 | 3.35E-01 | oocyte maturation |
| 42522 | 2.07E-01 | 3.35E-01 | regulation of tyrosine phosphorylation of Stat5 protein |
| 34377 | 2.07E-01 | 3.35E-01 | plasma lipoprotein particle assembly |
| 1958 | 2.07E-01 | 3.35E-01 | endochondral ossification |
| 6110 | 2.07E-01 | 3.35E-01 | regulation of glycolysis |
| 30949 | 2.07E-01 | 3.35E-01 | positive regulation of vascular endothelial growth factor receptor signaling pathway |
| 6547 | 2.07E-01 | 3.35E-01 | histidine metabolic process |
| 10714 | 2.07E-01 | 3.35E-01 | positive regulation of collagen metabolic process |
| 14821 | 2.07E-01 | 3.35E-01 | phasic smooth muscle contraction |
| 6684 | 2.07E-01 | 3.35E-01 | sphingomyelin metabolic process |
| 43586 | 2.07E-01 | 3.35E-01 | tongue development |
| 10885 | 2.07E-01 | 3.35E-01 | regulation of cholesterol storage |
| 2920 | 2.07E-01 | 3.35E-01 | regulation of humoral immune response |
| 3081 | 2.07E-01 | 3.35E-01 | regulation of systemic arterial blood pressure by renin-angiotensin |
| 48199 | 2.07E-01 | 3.35E-01 | vesicle targeting, to, from or within Golgi |
| 48488 | 2.07E-01 | 3.35E-01 | synaptic vesicle endocytosis |
| 32225 | 2.07E-01 | 3.35E-01 | regulation of synaptic transmission, dopaminergic |
| 65005 | 2.07E-01 | 3.35E-01 | protein-lipid complex assembly |
| 32868 | 2.09E-01 | 3.38E-01 | response to insulin stimulus |
| 71375 | 2.12E-01 | 3.42E-01 | cellular response to peptide hormone stimulus |
| 51262 | 2.12E-01 | 3.43E-01 | protein tetramerization |
| 46887 | 2.19E-01 | 3.50E-01 | positive regulation of hormone secretion |
| 51495 | 2.19E-01 | 3.50E-01 | positive regulation of cytoskeleton organization |
| 51248 | 2.20E-01 | 3.50E-01 | negative regulation of protein metabolic process |
| 48585 | 2.21E-01 | 3.50E-01 | negative regulation of response to stimulus |
| 32582 | 2.22E-01 | 3.50E-01 | negative regulation of gene-specific transcription |
| 70228 | 2.22E-01 | 3.50E-01 | regulation of lymphocyte apoptosis |
| 46006 | 2.22E-01 | 3.50E-01 | regulation of activated T cell proliferation |
| 46457 | 2.22E-01 | 3.50E-01 | prostanoid biosynthetic process |
| 1516 | 2.22E-01 | 3.50E-01 | prostaglandin biosynthetic process |
| 1542 | 2.22E-01 | 3.50E-01 | ovulation from ovarian follicle |
| 30318 | 2.22E-01 | 3.50E-01 | melanocyte differentiation |
| 46716 | 2.22E-01 | 3.50E-01 | muscle cell homeostasis |
| 55021 | 2.22E-01 | 3.50E-01 | regulation of cardiac muscle tissue growth |
| 55024 | 2.22E-01 | 3.50E-01 | regulation of cardiac muscle tissue development |
| 51209 | 2.22E-01 | 3.50E-01 | release of sequestered calcium ion into cytosol |
| 51282 | 2.22E-01 | 3.50E-01 | regulation of sequestering of calcium ion |
| 51283 | 2.22E-01 | 3.50E-01 | negative regulation of sequestering of calcium ion |
| 43114 | 2.22E-01 | 3.50E-01 | regulation of vascular permeability |
| 51452 | 2.22E-01 | 3.50E-01 | intracellular pH reduction |
| 43266 | 2.22E-01 | 3.50E-01 | regulation of potassium ion transport |
| 10559 | 2.22E-01 | 3.50E-01 | regulation of glycoprotein biosynthetic process |
| 10632 | 2.22E-01 | 3.50E-01 | regulation of epithelial cell migration |
| 6555 | 2.22E-01 | 3.50E-01 | methionine metabolic process |
| 43502 | 2.22E-01 | 3.50E-01 | regulation of muscle adaptation |
| 43536 | 2.22E-01 | 3.50E-01 | positive regulation of blood vessel endothelial cell migration |
| 10884 | 2.22E-01 | 3.50E-01 | positive regulation of lipid storage |
| 60043 | 2.22E-01 | 3.50E-01 | regulation of cardiac muscle cell proliferation |
| 31571 | 2.22E-01 | 3.50E-01 | G1/S DNA damage checkpoint |
| 3009 | 2.22E-01 | 3.50E-01 | skeletal muscle contraction |
| 44058 | 2.22E-01 | 3.50E-01 | regulation of digestive system process |
| 40036 | 2.22E-01 | 3.50E-01 | regulation of fibroblast growth factor receptor signaling pathway |
| 60749 | 2.22E-01 | 3.50E-01 | mammary gland alveolus development |
| 32091 | 2.22E-01 | 3.50E-01 | negative regulation of protein binding |
| 48596 | 2.22E-01 | 3.50E-01 | embryonic camera-type eye morphogenesis |
| 48662 | 2.22E-01 | 3.50E-01 | negative regulation of smooth muscle cell proliferation |
| 32653 | 2.22E-01 | 3.50E-01 | regulation of interleukin-10 production |
| 43933 | 2.23E-01 | 3.52E-01 | macromolecular complex subunit organization |
| 1936 | 2.26E-01 | 3.56E-01 | regulation of endothelial cell proliferation |
| 60326 | 2.26E-01 | 3.56E-01 | cell chemotaxis |
| 48520 | 2.26E-01 | 3.56E-01 | positive regulation of behavior |
| 51235 | 2.27E-01 | 3.56E-01 | maintenance of location |
| 6643 | 2.27E-01 | 3.56E-01 | membrane lipid metabolic process |
| 8406 | 2.29E-01 | 3.60E-01 | gonad development |
| 30335 | 2.29E-01 | 3.60E-01 | positive regulation of cell migration |
| 55002 | 2.33E-01 | 3.66E-01 | striated muscle cell development |
| 6639 | 2.33E-01 | 3.66E-01 | acylglycerol metabolic process |
| 7162 | 2.33E-01 | 3.66E-01 | negative regulation of cell adhesion |
| 33003 | 2.37E-01 | 3.66E-01 | regulation of mast cell activation |
| 33138 | 2.37E-01 | 3.66E-01 | positive regulation of peptidyl-serine phosphorylation |
| 33238 | 2.37E-01 | 3.66E-01 | regulation of cellular amine metabolic process |
| 70328 | 2.37E-01 | 3.66E-01 | triglyceride homeostasis |
| 45851 | 2.37E-01 | 3.66E-01 | pH reduction |
| 45909 | 2.37E-01 | 3.66E-01 | positive regulation of vasodilation |
| 9190 | 2.37E-01 | 3.66E-01 | cyclic nucleotide biosynthetic process |
| 9264 | 2.37E-01 | 3.66E-01 | deoxyribonucleotide catabolic process |
| 46504 | 2.37E-01 | 3.66E-01 | glycerol ether biosynthetic process |
| 50931 | 2.37E-01 | 3.66E-01 | pigment cell differentiation |
| 22409 | 2.37E-01 | 3.66E-01 | positive regulation of cell-cell adhesion |
| 51193 | 2.37E-01 | 3.66E-01 | regulation of cofactor metabolic process |
| 51196 | 2.37E-01 | 3.66E-01 | regulation of coenzyme metabolic process |
| 2286 | 2.37E-01 | 3.66E-01 | T cell activation involved in immune response |
| 51496 | 2.37E-01 | 3.66E-01 | positive regulation of stress fiber assembly |
| 6544 | 2.37E-01 | 3.66E-01 | glycine metabolic process |
| 51923 | 2.37E-01 | 3.66E-01 | sulfation |
| 60389 | 2.37E-01 | 3.66E-01 | pathway-restricted SMAD protein phosphorylation |
| 60420 | 2.37E-01 | 3.66E-01 | regulation of heart growth |
| 7263 | 2.37E-01 | 3.66E-01 | nitric oxide mediated signal transduction |
| 48339 | 2.37E-01 | 3.66E-01 | paraxial mesoderm development |
| 15800 | 2.37E-01 | 3.66E-01 | acidic amino acid transport |
| 34101 | 2.40E-01 | 3.71E-01 | erythrocyte homeostasis |
| 2274 | 2.40E-01 | 3.71E-01 | myeloid leukocyte activation |
| 43270 | 2.40E-01 | 3.71E-01 | positive regulation of ion transport |
| 10942 | 2.41E-01 | 3.72E-01 | positive regulation of cell death |
| 60537 | 2.41E-01 | 3.72E-01 | muscle tissue development |
| 7548 | 2.43E-01 | 3.75E-01 | sex differentiation |
| 42391 | 2.46E-01 | 3.78E-01 | regulation of membrane potential |
| 48754 | 2.47E-01 | 3.80E-01 | branching morphogenesis of a tube |
| 15674 | 2.47E-01 | 3.80E-01 | di-, tri-valent inorganic cation transport |
| 48593 | 2.48E-01 | 3.80E-01 | camera-type eye morphogenesis |
| 32770 | 2.51E-01 | 3.82E-01 | positive regulation of monooxygenase activity |
| 45604 | 2.51E-01 | 3.82E-01 | regulation of epidermal cell differentiation |
| 45921 | 2.51E-01 | 3.82E-01 | positive regulation of exocytosis |
| 9651 | 2.51E-01 | 3.82E-01 | response to salt stress |
| 21955 | 2.51E-01 | 3.82E-01 | central nervous system neuron axonogenesis |
| 71156 | 2.51E-01 | 3.82E-01 | regulation of cell cycle arrest |
| 14048 | 2.51E-01 | 3.82E-01 | regulation of glutamate secretion |
| 14068 | 2.51E-01 | 3.82E-01 | positive regulation of phosphoinositide 3-kinase cascade |
| 1937 | 2.51E-01 | 3.82E-01 | negative regulation of endothelial cell proliferation |
| 51354 | 2.51E-01 | 3.82E-01 | negative regulation of oxidoreductase activity |
| 51453 | 2.51E-01 | 3.82E-01 | regulation of intracellular pH |
| 43526 | 2.51E-01 | 3.82E-01 | neuroprotection |
| 10976 | 2.51E-01 | 3.82E-01 | positive regulation of neuron projection development |
| 51963 | 2.51E-01 | 3.82E-01 | regulation of synaptogenesis |
| 7274 | 2.51E-01 | 3.82E-01 | neuromuscular synaptic transmission |
| 44253 | 2.51E-01 | 3.82E-01 | positive regulation of multicellular organismal metabolic process |
| 32092 | 2.51E-01 | 3.82E-01 | positive regulation of protein binding |
| 2683 | 2.52E-01 | 3.83E-01 | negative regulation of immune system process |
| 51674 | 2.53E-01 | 3.84E-01 | localization of cell |
| 48870 | 2.53E-01 | 3.84E-01 | cell motility |
| 50920 | 2.55E-01 | 3.86E-01 | regulation of chemotaxis |
| 6662 | 2.55E-01 | 3.86E-01 | glycerol ether metabolic process |
| 51716 | 2.55E-01 | 3.87E-01 | cellular response to stimulus |
| 43281 | 2.57E-01 | 3.89E-01 | regulation of caspase activity |
| 6820 | 2.57E-01 | 3.89E-01 | anion transport |
| 82 | 2.62E-01 | 3.96E-01 | G1/S transition of mitotic cell cycle |
| 16486 | 2.66E-01 | 3.98E-01 | peptide hormone processing |
| 32885 | 2.66E-01 | 3.98E-01 | regulation of polysaccharide biosynthetic process |
| 32965 | 2.66E-01 | 3.98E-01 | regulation of collagen biosynthetic process |
| 70206 | 2.66E-01 | 3.98E-01 | protein trimerization |
| 45940 | 2.66E-01 | 3.98E-01 | positive regulation of steroid metabolic process |
| 9220 | 2.66E-01 | 3.98E-01 | pyrimidine ribonucleotide biosynthetic process |
| 9584 | 2.66E-01 | 3.98E-01 | detection of visible light |
| 1502 | 2.66E-01 | 3.98E-01 | cartilage condensation |
| 14066 | 2.66E-01 | 3.98E-01 | regulation of phosphoinositide 3-kinase cascade |
| 5979 | 2.66E-01 | 3.98E-01 | regulation of glycogen biosynthetic process |
| 51279 | 2.66E-01 | 3.98E-01 | regulation of release of sequestered calcium ion into cytosol |
| 51402 | 2.66E-01 | 3.98E-01 | neuron apoptosis |
| 30947 | 2.66E-01 | 3.98E-01 | regulation of vascular endothelial growth factor receptor signaling pathway |
| 10812 | 2.66E-01 | 3.98E-01 | negative regulation of cell-substrate adhesion |
| 10962 | 2.66E-01 | 3.98E-01 | regulation of glucan biosynthetic process |
| 15807 | 2.66E-01 | 3.98E-01 | L-amino acid transport |
| 48844 | 2.66E-01 | 3.98E-01 | artery morphogenesis |
| 80134 | 2.66E-01 | 3.98E-01 | regulation of response to stress |
| 10647 | 2.66E-01 | 3.98E-01 | positive regulation of cell communication |
| 51272 | 2.66E-01 | 3.98E-01 | positive regulation of cellular component movement |
| 18904 | 2.69E-01 | 4.01E-01 | organic ether metabolic process |
| 33124 | 2.75E-01 | 4.09E-01 | regulation of GTP catabolic process |
| 43087 | 2.75E-01 | 4.09E-01 | regulation of GTPase activity |
| 23033 | 2.75E-01 | 4.09E-01 | signaling pathway |
| 50905 | 2.76E-01 | 4.09E-01 | neuromuscular process |
| 51350 | 2.76E-01 | 4.09E-01 | negative regulation of lyase activity |
| 31280 | 2.76E-01 | 4.09E-01 | negative regulation of cyclase activity |
| 7194 | 2.76E-01 | 4.09E-01 | negative regulation of adenylate cyclase activity |
| 9967 | 2.76E-01 | 4.09E-01 | positive regulation of signal transduction |
| 48562 | 2.79E-01 | 4.09E-01 | embryonic organ morphogenesis |
| 80 | 2.80E-01 | 4.09E-01 | G1 phase of mitotic cell cycle |
| 32881 | 2.80E-01 | 4.09E-01 | regulation of polysaccharide metabolic process |
| 33198 | 2.80E-01 | 4.09E-01 | response to ATP |
| 33280 | 2.80E-01 | 4.09E-01 | response to vitamin D |
| 9218 | 2.80E-01 | 4.09E-01 | pyrimidine ribonucleotide metabolic process |
| 70873 | 2.80E-01 | 4.09E-01 | regulation of glycogen metabolic process |
| 21795 | 2.80E-01 | 4.09E-01 | cerebral cortex cell migration |
| 42311 | 2.80E-01 | 4.09E-01 | vasodilation |
| 34199 | 2.80E-01 | 4.09E-01 | activation of protein kinase A activity |
| 46513 | 2.80E-01 | 4.09E-01 | ceramide biosynthetic process |
| 30279 | 2.80E-01 | 4.09E-01 | negative regulation of ossification |
| 42573 | 2.80E-01 | 4.09E-01 | retinoic acid metabolic process |
| 30641 | 2.80E-01 | 4.09E-01 | regulation of cellular pH |
| 31076 | 2.80E-01 | 4.09E-01 | embryonic camera-type eye development |
| 43470 | 2.80E-01 | 4.09E-01 | regulation of carbohydrate catabolic process |
| 43471 | 2.80E-01 | 4.09E-01 | regulation of cellular carbohydrate catabolic process |
| 10712 | 2.80E-01 | 4.09E-01 | regulation of collagen metabolic process |
| 6637 | 2.80E-01 | 4.09E-01 | acyl-CoA metabolic process |
| 35383 | 2.80E-01 | 4.09E-01 | thioester metabolic process |
| 60048 | 2.80E-01 | 4.09E-01 | cardiac muscle contraction |
| 2709 | 2.80E-01 | 4.09E-01 | regulation of T cell mediated immunity |
| 51926 | 2.80E-01 | 4.09E-01 | negative regulation of calcium ion transport |
| 6901 | 2.80E-01 | 4.09E-01 | vesicle coating |
| 48048 | 2.80E-01 | 4.09E-01 | embryonic eye morphogenesis |
| 60350 | 2.80E-01 | 4.09E-01 | endochondral bone morphogenesis |
| 60445 | 2.80E-01 | 4.09E-01 | branching involved in salivary gland morphogenesis |
| 32233 | 2.80E-01 | 4.09E-01 | positive regulation of actin filament bundle assembly |
| 9617 | 2.80E-01 | 4.09E-01 | response to bacterium |
| 45761 | 2.83E-01 | 4.12E-01 | regulation of adenylate cyclase activity |
| 55001 | 2.83E-01 | 4.12E-01 | muscle cell development |
| 6898 | 2.83E-01 | 4.12E-01 | receptor-mediated endocytosis |
| 16050 | 2.83E-01 | 4.12E-01 | vesicle organization |
| 165 | 2.83E-01 | 4.12E-01 | MAPKKK cascade |
| 21700 | 2.88E-01 | 4.19E-01 | developmental maturation |
| 52548 | 2.88E-01 | 4.19E-01 | regulation of endopeptidase activity |
| 6140 | 2.88E-01 | 4.19E-01 | regulation of nucleotide metabolic process |
| 23034 | 2.90E-01 | 4.20E-01 | intracellular signaling pathway |
| 48646 | 2.90E-01 | 4.20E-01 | anatomical structure formation involved in morphogenesis |
| 45444 | 2.90E-01 | 4.20E-01 | fat cell differentiation |
| 2695 | 2.90E-01 | 4.20E-01 | negative regulation of leukocyte activation |
| 7190 | 2.90E-01 | 4.20E-01 | activation of adenylate cyclase activity |
| 30001 | 2.91E-01 | 4.20E-01 | metal ion transport |
| 16477 | 2.91E-01 | 4.20E-01 | cell migration |
| 45445 | 2.93E-01 | 4.20E-01 | myoblast differentiation |
| 42116 | 2.93E-01 | 4.20E-01 | macrophage activation |
| 70997 | 2.93E-01 | 4.20E-01 | neuron death |
| 42462 | 2.93E-01 | 4.20E-01 | eye photoreceptor cell development |
| 46638 | 2.93E-01 | 4.20E-01 | positive regulation of alpha-beta T cell differentiation |
| 34381 | 2.93E-01 | 4.20E-01 | lipoprotein particle clearance |
| 46717 | 2.93E-01 | 4.20E-01 | acid secretion |
| 18205 | 2.93E-01 | 4.20E-01 | peptidyl-lysine modification |
| 30593 | 2.93E-01 | 4.20E-01 | neutrophil chemotaxis |
| 51181 | 2.93E-01 | 4.20E-01 | cofactor transport |
| 30728 | 2.93E-01 | 4.20E-01 | ovulation |
| 30901 | 2.93E-01 | 4.20E-01 | midbrain development |
| 43489 | 2.93E-01 | 4.20E-01 | RNA stabilization |
| 2718 | 2.93E-01 | 4.20E-01 | regulation of cytokine production involved in immune response |
| 60079 | 2.93E-01 | 4.20E-01 | regulation of excitatory postsynaptic membrane potential |
| 48255 | 2.93E-01 | 4.20E-01 | mRNA stabilization |
| 60840 | 2.93E-01 | 4.20E-01 | artery development |
| 32410 | 2.93E-01 | 4.20E-01 | negative regulation of transporter activity |
| 31279 | 2.93E-01 | 4.20E-01 | regulation of cyclase activity |
| 50776 | 2.94E-01 | 4.20E-01 | regulation of immune response |
| 45860 | 2.97E-01 | 4.24E-01 | positive regulation of protein kinase activity |
| 45762 | 2.97E-01 | 4.24E-01 | positive regulation of adenylate cyclase activity |
| 42773 | 2.97E-01 | 4.24E-01 | ATP synthesis coupled electron transport |
| 42775 | 2.97E-01 | 4.24E-01 | mitochondrial ATP synthesis coupled electron transport |
| 43433 | 2.97E-01 | 4.24E-01 | negative regulation of transcription factor activity |
| 90048 | 2.97E-01 | 4.24E-01 | negative regulation of transcription regulator activity |
| 32501 | 2.98E-01 | 4.24E-01 | multicellular organismal process |
| 51339 | 3.04E-01 | 4.32E-01 | regulation of lyase activity |
| 50731 | 3.04E-01 | 4.32E-01 | positive regulation of peptidyl-tyrosine phosphorylation |
| 51346 | 3.04E-01 | 4.32E-01 | negative regulation of hydrolase activity |
| 31281 | 3.04E-01 | 4.32E-01 | positive regulation of cyclase activity |
| 48738 | 3.04E-01 | 4.32E-01 | cardiac muscle tissue development |
| 50778 | 3.05E-01 | 4.33E-01 | positive regulation of immune response |
| 30177 | 3.07E-01 | 4.33E-01 | positive regulation of Wnt receptor signaling pathway |
| 50807 | 3.07E-01 | 4.33E-01 | regulation of synapse organization |
| 71300 | 3.07E-01 | 4.33E-01 | cellular response to retinoic acid |
| 1759 | 3.07E-01 | 4.33E-01 | induction of an organ |
| 50999 | 3.07E-01 | 4.33E-01 | regulation of nitric-oxide synthase activity |
| 30539 | 3.07E-01 | 4.33E-01 | male genitalia development |
| 2285 | 3.07E-01 | 4.33E-01 | lymphocyte activation involved in immune response |
| 31069 | 3.07E-01 | 4.33E-01 | hair follicle morphogenesis |
| 6900 | 3.07E-01 | 4.33E-01 | membrane budding |
| 51966 | 3.07E-01 | 4.33E-01 | regulation of synaptic transmission, glutamatergic |
| 60402 | 3.07E-01 | 4.33E-01 | calcium ion transport into cytosol |
| 51329 | 3.09E-01 | 4.36E-01 | interphase of mitotic cell cycle |
| 2703 | 3.11E-01 | 4.39E-01 | regulation of leukocyte mediated immunity |
| 65003 | 3.12E-01 | 4.40E-01 | macromolecular complex assembly |
| 79 | 3.18E-01 | 4.45E-01 | regulation of cyclin-dependent protein kinase activity |
| 51349 | 3.18E-01 | 4.45E-01 | positive regulation of lyase activity |
| 48704 | 3.18E-01 | 4.45E-01 | embryonic skeletal system morphogenesis |
| 8206 | 3.20E-01 | 4.45E-01 | bile acid metabolic process |
| 33135 | 3.20E-01 | 4.45E-01 | regulation of peptidyl-serine phosphorylation |
| 45446 | 3.20E-01 | 4.45E-01 | endothelial cell differentiation |
| 45884 | 3.20E-01 | 4.45E-01 | regulation of survival gene product expression |
| 9595 | 3.20E-01 | 4.45E-01 | detection of biotic stimulus |
| 22029 | 3.20E-01 | 4.45E-01 | telencephalon cell migration |
| 30317 | 3.20E-01 | 4.45E-01 | sperm motility |
| 71299 | 3.20E-01 | 4.45E-01 | cellular response to vitamin A |
| 1754 | 3.20E-01 | 4.45E-01 | eye photoreceptor cell differentiation |
| 46849 | 3.20E-01 | 4.45E-01 | bone remodeling |
| 34612 | 3.20E-01 | 4.45E-01 | response to tumor necrosis factor |
| 46928 | 3.20E-01 | 4.45E-01 | regulation of neurotransmitter secretion |
| 51318 | 3.20E-01 | 4.45E-01 | G1 phase |
| 43200 | 3.20E-01 | 4.45E-01 | response to amino acid stimulus |
| 51492 | 3.20E-01 | 4.45E-01 | regulation of stress fiber assembly |
| 7214 | 3.20E-01 | 4.45E-01 | gamma-aminobutyric acid signaling pathway |
| 44246 | 3.20E-01 | 4.45E-01 | regulation of multicellular organismal metabolic process |
| 48599 | 3.20E-01 | 4.45E-01 | oocyte development |
| 32436 | 3.20E-01 | 4.45E-01 | positive regulation of proteasomal ubiquitin-dependent protein catabolic process |
| 31329 | 3.20E-01 | 4.45E-01 | regulation of cellular catabolic process |
| 6139 | 3.23E-01 | 4.49E-01 | nucleobase, nucleoside, nucleotide and nucleic acid metabolic process |
| 7264 | 3.25E-01 | 4.51E-01 | small GTPase mediated signal transduction |
| 16485 | 3.25E-01 | 4.51E-01 | protein processing |
| 2697 | 3.25E-01 | 4.51E-01 | regulation of immune effector process |
| 33674 | 3.27E-01 | 4.53E-01 | positive regulation of kinase activity |
| 48513 | 3.30E-01 | 4.56E-01 | organ development |
| 51325 | 3.30E-01 | 4.56E-01 | interphase |
| 32269 | 3.32E-01 | 4.56E-01 | negative regulation of cellular protein metabolic process |
| 9582 | 3.32E-01 | 4.56E-01 | detection of abiotic stimulus |
| 50866 | 3.32E-01 | 4.56E-01 | negative regulation of cell activation |
| 86 | 3.33E-01 | 4.56E-01 | G2/M transition of mitotic cell cycle |
| 45776 | 3.33E-01 | 4.56E-01 | negative regulation of blood pressure |
| 45913 | 3.33E-01 | 4.56E-01 | positive regulation of carbohydrate metabolic process |
| 9394 | 3.33E-01 | 4.56E-01 | 2'-deoxyribonucleotide metabolic process |
| 21885 | 3.33E-01 | 4.56E-01 | forebrain cell migration |
| 46520 | 3.33E-01 | 4.56E-01 | sphingoid biosynthetic process |
| 42461 | 3.33E-01 | 4.56E-01 | photoreceptor cell development |
| 46635 | 3.33E-01 | 4.56E-01 | positive regulation of alpha-beta T cell activation |
| 9994 | 3.33E-01 | 4.56E-01 | oocyte differentiation |
| 30514 | 3.33E-01 | 4.56E-01 | negative regulation of BMP signaling pathway |
| 51017 | 3.33E-01 | 4.56E-01 | actin filament bundle assembly |
| 10676 | 3.33E-01 | 4.56E-01 | positive regulation of cellular carbohydrate metabolic process |
| 60047 | 3.33E-01 | 4.56E-01 | heart contraction |
| 6890 | 3.33E-01 | 4.56E-01 | retrograde vesicle-mediated transport, Golgi to ER |
| 3015 | 3.33E-01 | 4.56E-01 | heart process |
| 48259 | 3.33E-01 | 4.56E-01 | regulation of receptor-mediated endocytosis |
| 15850 | 3.33E-01 | 4.56E-01 | organic alcohol transport |
| 32273 | 3.33E-01 | 4.56E-01 | positive regulation of protein polymerization |
| 32655 | 3.33E-01 | 4.56E-01 | regulation of interleukin-12 production |
| 60249 | 3.35E-01 | 4.58E-01 | anatomical structure homeostasis |
| 51098 | 3.36E-01 | 4.58E-01 | regulation of binding |
| 43065 | 3.38E-01 | 4.61E-01 | positive regulation of apoptosis |
| 1894 | 3.39E-01 | 4.63E-01 | tissue homeostasis |
| 10551 | 3.40E-01 | 4.63E-01 | regulation of gene-specific transcription from RNA polymerase II promoter |
| 8624 | 3.41E-01 | 4.64E-01 | induction of apoptosis by extracellular signals |
| 7178 | 3.41E-01 | 4.64E-01 | transmembrane receptor protein serine/threonine kinase signaling pathway |
| 33121 | 3.44E-01 | 4.67E-01 | regulation of purine nucleotide catabolic process |
| 30811 | 3.44E-01 | 4.67E-01 | regulation of nucleotide catabolic process |
| 43068 | 3.45E-01 | 4.67E-01 | positive regulation of programmed cell death |
| 45682 | 3.46E-01 | 4.67E-01 | regulation of epidermis development |
| 70848 | 3.46E-01 | 4.67E-01 | response to growth factor stimulus |
| 42312 | 3.46E-01 | 4.67E-01 | regulation of vasodilation |
| 50803 | 3.46E-01 | 4.67E-01 | regulation of synapse structure and activity |
| 55010 | 3.46E-01 | 4.67E-01 | ventricular cardiac muscle tissue morphogenesis |
| 1959 | 3.46E-01 | 4.67E-01 | regulation of cytokine-mediated signaling pathway |
| 31056 | 3.46E-01 | 4.67E-01 | regulation of histone modification |
| 43535 | 3.46E-01 | 4.67E-01 | regulation of blood vessel endothelial cell migration |
| 60078 | 3.46E-01 | 4.67E-01 | regulation of postsynaptic membrane potential |
| 6903 | 3.46E-01 | 4.67E-01 | vesicle targeting |
| 60491 | 3.46E-01 | 4.67E-01 | regulation of cell projection assembly |
| 3158 | 3.46E-01 | 4.67E-01 | endothelium development |
| 3229 | 3.46E-01 | 4.67E-01 | ventricular cardiac muscle tissue development |
| 15695 | 3.46E-01 | 4.67E-01 | organic cation transport |
| 9408 | 3.46E-01 | 4.67E-01 | response to heat |
| 9607 | 3.50E-01 | 4.73E-01 | response to biotic stimulus |
| 22904 | 3.53E-01 | 4.76E-01 | respiratory electron transport chain |
| 43392 | 3.53E-01 | 4.76E-01 | negative regulation of DNA binding |
| 44087 | 3.57E-01 | 4.79E-01 | regulation of cellular component biogenesis |
| 51347 | 3.58E-01 | 4.79E-01 | positive regulation of transferase activity |
| 45923 | 3.59E-01 | 4.79E-01 | positive regulation of fatty acid metabolic process |
| 46530 | 3.59E-01 | 4.79E-01 | photoreceptor cell differentiation |
| 46637 | 3.59E-01 | 4.79E-01 | regulation of alpha-beta T cell differentiation |
| 71295 | 3.59E-01 | 4.79E-01 | cellular response to vitamin |
| 46889 | 3.59E-01 | 4.79E-01 | positive regulation of lipid biosynthetic process |
| 51180 | 3.59E-01 | 4.79E-01 | vitamin transport |
| 6221 | 3.59E-01 | 4.79E-01 | pyrimidine nucleotide biosynthetic process |
| 35264 | 3.59E-01 | 4.79E-01 | multicellular organism growth |
| 2690 | 3.59E-01 | 4.79E-01 | positive regulation of leukocyte chemotaxis |
| 48066 | 3.59E-01 | 4.79E-01 | developmental pigmentation |
| 3156 | 3.59E-01 | 4.79E-01 | regulation of organ formation |
| 32642 | 3.59E-01 | 4.79E-01 | regulation of chemokine production |
| 42129 | 3.60E-01 | 4.81E-01 | regulation of T cell proliferation |
| 51651 | 3.60E-01 | 4.81E-01 | maintenance of location in cell |
| 6986 | 3.60E-01 | 4.81E-01 | response to unfolded protein |
| 7265 | 3.62E-01 | 4.82E-01 | Ras protein signal transduction |
| 30817 | 3.67E-01 | 4.89E-01 | regulation of cAMP biosynthetic process |
| 30900 | 3.70E-01 | 4.92E-01 | forebrain development |
| 8542 | 3.71E-01 | 4.92E-01 | visual learning |
| 42058 | 3.71E-01 | 4.92E-01 | regulation of epidermal growth factor receptor signaling pathway |
| 30195 | 3.71E-01 | 4.92E-01 | negative regulation of blood coagulation |
| 14823 | 3.71E-01 | 4.92E-01 | response to activity |
| 6942 | 3.71E-01 | 4.92E-01 | regulation of striated muscle contraction |
| 31575 | 3.71E-01 | 4.92E-01 | G1/S transition checkpoint |
| 40018 | 3.71E-01 | 4.92E-01 | positive regulation of multicellular organism growth |
| 32231 | 3.71E-01 | 4.92E-01 | regulation of actin filament bundle assembly |
| 16339 | 3.71E-01 | 4.92E-01 | calcium-dependent cell-cell adhesion |
| 5976 | 3.72E-01 | 4.93E-01 | polysaccharide metabolic process |
| 7276 | 3.73E-01 | 4.93E-01 | gamete generation |
| 51707 | 3.74E-01 | 4.95E-01 | response to other organism |
| 7292 | 3.74E-01 | 4.95E-01 | female gamete generation |
| 30814 | 3.77E-01 | 4.98E-01 | regulation of cAMP metabolic process |
| 43123 | 3.77E-01 | 4.98E-01 | positive regulation of I-kappaB kinase/NF-kappaB cascade |
| 51604 | 3.77E-01 | 4.98E-01 | protein maturation |
| 48608 | 3.79E-01 | 5.00E-01 | reproductive structure development |
| 40008 | 3.80E-01 | 5.01E-01 | regulation of growth |
| 50795 | 3.81E-01 | 5.02E-01 | regulation of behavior |
| 32268 | 3.81E-01 | 5.02E-01 | regulation of cellular protein metabolic process |
| 42692 | 3.82E-01 | 5.02E-01 | muscle cell differentiation |
| 45168 | 3.83E-01 | 5.02E-01 | cell-cell signaling involved in cell fate commitment |
| 42755 | 3.83E-01 | 5.02E-01 | eating behavior |
| 2260 | 3.83E-01 | 5.02E-01 | lymphocyte homeostasis |
| 51588 | 3.83E-01 | 5.02E-01 | regulation of neurotransmitter transport |
| 31128 | 3.83E-01 | 5.02E-01 | developmental induction |
| 43542 | 3.83E-01 | 5.02E-01 | endothelial cell migration |
| 3208 | 3.83E-01 | 5.02E-01 | cardiac ventricle morphogenesis |
| 35467 | 3.85E-01 | 5.05E-01 | negative regulation of signaling pathway |
| 51606 | 3.88E-01 | 5.08E-01 | detection of stimulus |
| 45787 | 3.88E-01 | 5.08E-01 | positive regulation of cell cycle |
| 46486 | 3.92E-01 | 5.13E-01 | glycerolipid metabolic process |
| 48568 | 3.92E-01 | 5.13E-01 | embryonic organ development |
| 23014 | 3.94E-01 | 5.13E-01 | signal transmission via phosphorylation event |
| 7243 | 3.94E-01 | 5.13E-01 | intracellular protein kinase cascade |
| 33692 | 3.95E-01 | 5.13E-01 | cellular polysaccharide biosynthetic process |
| 42130 | 3.95E-01 | 5.13E-01 | negative regulation of T cell proliferation |
| 50819 | 3.95E-01 | 5.13E-01 | negative regulation of coagulation |
| 18149 | 3.95E-01 | 5.13E-01 | peptide cross-linking |
| 6040 | 3.95E-01 | 5.13E-01 | amino sugar metabolic process |
| 10332 | 3.95E-01 | 5.13E-01 | response to gamma radiation |
| 6284 | 3.95E-01 | 5.13E-01 | base-excision repair |
| 2377 | 3.95E-01 | 5.13E-01 | immunoglobulin production |
| 31110 | 3.95E-01 | 5.13E-01 | regulation of microtubule polymerization or depolymerization |
| 60349 | 3.95E-01 | 5.13E-01 | bone morphogenesis |
| 7435 | 3.95E-01 | 5.13E-01 | salivary gland morphogenesis |
| 90090 | 3.95E-01 | 5.13E-01 | negative regulation of canonical Wnt receptor signaling pathway |
| 61061 | 3.95E-01 | 5.13E-01 | muscle structure development |
| 30334 | 3.96E-01 | 5.14E-01 | regulation of cell migration |
| 8585 | 4.01E-01 | 5.20E-01 | female gonad development |
| 48518 | 4.05E-01 | 5.24E-01 | positive regulation of biological process |
| 9262 | 4.06E-01 | 5.24E-01 | deoxyribonucleotide metabolic process |
| 21782 | 4.06E-01 | 5.24E-01 | glial cell development |
| 55008 | 4.06E-01 | 5.24E-01 | cardiac muscle tissue morphogenesis |
| 2440 | 4.06E-01 | 5.24E-01 | production of molecular mediator of immune response |
| 2673 | 4.06E-01 | 5.24E-01 | regulation of acute inflammatory response |
| 10883 | 4.06E-01 | 5.24E-01 | regulation of lipid storage |
| 60415 | 4.06E-01 | 5.24E-01 | muscle tissue morphogenesis |
| 7632 | 4.06E-01 | 5.24E-01 | visual behavior |
| 32434 | 4.06E-01 | 5.24E-01 | regulation of proteasomal ubiquitin-dependent protein catabolic process |
| 61136 | 4.06E-01 | 5.24E-01 | regulation of proteasomal protein catabolic process |
| 22602 | 4.08E-01 | 5.25E-01 | ovulation cycle process |
| 30802 | 4.08E-01 | 5.25E-01 | regulation of cyclic nucleotide biosynthetic process |
| 30808 | 4.08E-01 | 5.25E-01 | regulation of nucleotide biosynthetic process |
| 51050 | 4.12E-01 | 5.30E-01 | positive regulation of transport |
| 14706 | 4.13E-01 | 5.31E-01 | striated muscle tissue development |
| 32147 | 4.13E-01 | 5.31E-01 | activation of protein kinase activity |
| 48592 | 4.15E-01 | 5.33E-01 | eye morphogenesis |
| 272 | 4.18E-01 | 5.34E-01 | polysaccharide catabolic process |
| 45582 | 4.18E-01 | 5.34E-01 | positive regulation of T cell differentiation |
| 46677 | 4.18E-01 | 5.34E-01 | response to antibiotic |
| 50777 | 4.18E-01 | 5.34E-01 | negative regulation of immune response |
| 30330 | 4.18E-01 | 5.34E-01 | DNA damage response, signal transduction by p53 class mediator |
| 1658 | 4.18E-01 | 5.34E-01 | branching involved in ureteric bud morphogenesis |
| 50810 | 4.18E-01 | 5.34E-01 | regulation of steroid biosynthetic process |
| 2688 | 4.18E-01 | 5.34E-01 | regulation of leukocyte chemotaxis |
| 48286 | 4.18E-01 | 5.34E-01 | lung alveolus development |
| 7431 | 4.18E-01 | 5.34E-01 | salivary gland development |
| 51130 | 4.19E-01 | 5.35E-01 | positive regulation of cellular component organization |
| 10740 | 4.19E-01 | 5.35E-01 | positive regulation of intracellular protein kinase cascade |
| 30799 | 4.23E-01 | 5.40E-01 | regulation of cyclic nucleotide metabolic process |
| 46546 | 4.28E-01 | 5.45E-01 | development of primary male sexual characteristics |
| 30534 | 4.28E-01 | 5.45E-01 | adult behavior |
| 6813 | 4.29E-01 | 5.45E-01 | potassium ion transport |
| 21983 | 4.29E-01 | 5.45E-01 | pituitary gland development |
| 1523 | 4.29E-01 | 5.45E-01 | retinoid metabolic process |
| 51289 | 4.29E-01 | 5.45E-01 | protein homotetramerization |
| 6776 | 4.29E-01 | 5.45E-01 | vitamin A metabolic process |
| 48806 | 4.29E-01 | 5.45E-01 | genitalia development |
| 16101 | 4.29E-01 | 5.45E-01 | diterpenoid metabolic process |
| 1775 | 4.29E-01 | 5.45E-01 | cell activation |
| 51704 | 4.31E-01 | 5.47E-01 | multi-organism process |
| 21537 | 4.34E-01 | 5.51E-01 | telencephalon development |
| 43122 | 4.39E-01 | 5.55E-01 | regulation of I-kappaB kinase/NF-kappaB cascade |
| 51246 | 4.39E-01 | 5.55E-01 | regulation of protein metabolic process |
| 32963 | 4.40E-01 | 5.55E-01 | collagen metabolic process |
| 45995 | 4.40E-01 | 5.55E-01 | regulation of embryonic development |
| 21954 | 4.40E-01 | 5.55E-01 | central nervous system neuron development |
| 42509 | 4.40E-01 | 5.55E-01 | regulation of tyrosine phosphorylation of STAT protein |
| 30510 | 4.40E-01 | 5.55E-01 | regulation of BMP signaling pathway |
| 60443 | 4.40E-01 | 5.55E-01 | mammary gland morphogenesis |
| 60675 | 4.40E-01 | 5.55E-01 | ureteric bud morphogenesis |
| 35468 | 4.41E-01 | 5.56E-01 | positive regulation of signaling pathway |
| 46545 | 4.41E-01 | 5.56E-01 | development of primary female sexual characteristics |
| 22612 | 4.47E-01 | 5.64E-01 | gland morphogenesis |
| 9894 | 4.50E-01 | 5.65E-01 | regulation of catabolic process |
| 32945 | 4.50E-01 | 5.65E-01 | negative regulation of mononuclear cell proliferation |
| 45621 | 4.50E-01 | 5.65E-01 | positive regulation of lymphocyte differentiation |
| 70664 | 4.50E-01 | 5.65E-01 | negative regulation of leukocyte proliferation |
| 50672 | 4.50E-01 | 5.65E-01 | negative regulation of lymphocyte proliferation |
| 46634 | 4.50E-01 | 5.65E-01 | regulation of alpha-beta T cell activation |
| 50885 | 4.50E-01 | 5.65E-01 | neuromuscular process controlling balance |
| 2687 | 4.50E-01 | 5.65E-01 | positive regulation of leukocyte migration |
| 3231 | 4.50E-01 | 5.65E-01 | cardiac ventricle development |
| 16055 | 4.54E-01 | 5.69E-01 | Wnt receptor signaling pathway |
| 42698 | 4.54E-01 | 5.69E-01 | ovulation cycle |
| 7005 | 4.59E-01 | 5.75E-01 | mitochondrion organization |
| 45321 | 4.60E-01 | 5.75E-01 | leukocyte activation |
| 9306 | 4.61E-01 | 5.75E-01 | protein secretion |
| 42446 | 4.61E-01 | 5.75E-01 | hormone biosynthetic process |
| 50728 | 4.61E-01 | 5.75E-01 | negative regulation of inflammatory response |
| 1776 | 4.61E-01 | 5.75E-01 | leukocyte homeostasis |
| 1938 | 4.61E-01 | 5.75E-01 | positive regulation of endothelial cell proliferation |
| 31334 | 4.61E-01 | 5.75E-01 | positive regulation of protein complex assembly |
| 48489 | 4.61E-01 | 5.75E-01 | synaptic vesicle transport |
| 8219 | 4.65E-01 | 5.80E-01 | cell death |
| 60429 | 4.65E-01 | 5.80E-01 | epithelium development |
| 9653 | 4.68E-01 | 5.83E-01 | anatomical structure morphogenesis |
| 8630 | 4.71E-01 | 5.85E-01 | DNA damage response, signal transduction resulting in induction of apoptosis |
| 50906 | 4.71E-01 | 5.85E-01 | detection of stimulus involved in sensory perception |
| 30500 | 4.71E-01 | 5.85E-01 | regulation of bone mineralization |
| 71466 | 4.71E-01 | 5.85E-01 | cellular response to xenobiotic stimulus |
| 43409 | 4.71E-01 | 5.85E-01 | negative regulation of MAPKKK cascade |
| 51650 | 4.71E-01 | 5.85E-01 | establishment of vesicle localization |
| 6805 | 4.71E-01 | 5.85E-01 | xenobiotic metabolic process |
| 46660 | 4.73E-01 | 5.86E-01 | female sex differentiation |
| 46661 | 4.73E-01 | 5.86E-01 | male sex differentiation |
| 43410 | 4.73E-01 | 5.86E-01 | positive regulation of MAPKKK cascade |
| 45017 | 4.73E-01 | 5.86E-01 | glycerolipid biosynthetic process |
| 16265 | 4.76E-01 | 5.89E-01 | death |
| 48522 | 4.77E-01 | 5.90E-01 | positive regulation of cellular process |
| 48731 | 4.77E-01 | 5.90E-01 | system development |
| 9593 | 4.81E-01 | 5.94E-01 | detection of chemical stimulus |
| 30148 | 4.81E-01 | 5.94E-01 | sphingolipid biosynthetic process |
| 21987 | 4.81E-01 | 5.94E-01 | cerebral cortex development |
| 6885 | 4.81E-01 | 5.94E-01 | regulation of pH |
| 3206 | 4.81E-01 | 5.94E-01 | cardiac chamber morphogenesis |
| 7416 | 4.81E-01 | 5.94E-01 | synapse assembly |
| 51270 | 4.82E-01 | 5.94E-01 | regulation of cellular component movement |
| 51090 | 4.83E-01 | 5.95E-01 | regulation of transcription factor activity |
| 90046 | 4.83E-01 | 5.95E-01 | regulation of transcription regulator activity |
| 45927 | 4.85E-01 | 5.97E-01 | positive regulation of growth |
| 50678 | 4.85E-01 | 5.97E-01 | regulation of epithelial cell proliferation |
| 7160 | 4.85E-01 | 5.97E-01 | cell-matrix adhesion |
| 21761 | 4.91E-01 | 6.03E-01 | limbic system development |
| 50851 | 4.91E-01 | 6.03E-01 | antigen receptor-mediated signaling pathway |
| 7205 | 4.91E-01 | 6.03E-01 | activation of protein kinase C activity by G-protein coupled receptor protein signaling pathway |
| 48747 | 4.91E-01 | 6.03E-01 | muscle fiber development |
| 51146 | 4.91E-01 | 6.03E-01 | striated muscle cell differentiation |
| 7167 | 4.93E-01 | 6.04E-01 | enzyme linked receptor protein signaling pathway |
| 10564 | 4.93E-01 | 6.04E-01 | regulation of cell cycle process |
| 51094 | 4.96E-01 | 6.07E-01 | positive regulation of developmental process |
| 3006 | 4.96E-01 | 6.07E-01 | reproductive developmental process |
| 45765 | 4.97E-01 | 6.08E-01 | regulation of angiogenesis |
| 3007 | 4.97E-01 | 6.08E-01 | heart morphogenesis |
| 43161 | 4.98E-01 | 6.08E-01 | proteasomal ubiquitin-dependent protein catabolic process |
| 10498 | 4.98E-01 | 6.08E-01 | proteasomal protein catabolic process |
| 46425 | 5.01E-01 | 6.10E-01 | regulation of JAK-STAT cascade |
| 1649 | 5.01E-01 | 6.10E-01 | osteoblast differentiation |
| 55072 | 5.01E-01 | 6.10E-01 | iron ion homeostasis |
| 31100 | 5.01E-01 | 6.10E-01 | organ regeneration |
| 35272 | 5.01E-01 | 6.10E-01 | exocrine system development |
| 7585 | 5.01E-01 | 6.10E-01 | respiratory gaseous exchange |
| 2526 | 5.10E-01 | 6.20E-01 | acute inflammatory response |
| 32502 | 5.10E-01 | 6.20E-01 | developmental process |
| 70167 | 5.11E-01 | 6.20E-01 | regulation of biomineral formation |
| 17148 | 5.11E-01 | 6.20E-01 | negative regulation of translation |
| 45862 | 5.11E-01 | 6.20E-01 | positive regulation of proteolysis |
| 46467 | 5.11E-01 | 6.20E-01 | membrane lipid biosynthetic process |
| 6721 | 5.11E-01 | 6.20E-01 | terpenoid metabolic process |
| 21536 | 5.20E-01 | 6.29E-01 | diencephalon development |
| 9583 | 5.20E-01 | 6.29E-01 | detection of light stimulus |
| 2064 | 5.20E-01 | 6.29E-01 | epithelial cell development |
| 51648 | 5.20E-01 | 6.29E-01 | vesicle localization |
| 2573 | 5.20E-01 | 6.29E-01 | myeloid leukocyte differentiation |
| 6941 | 5.20E-01 | 6.29E-01 | striated muscle contraction |
| 9416 | 5.21E-01 | 6.30E-01 | response to light stimulus |
| 10638 | 5.21E-01 | 6.30E-01 | positive regulation of organelle organization |
| 8213 | 5.29E-01 | 6.37E-01 | protein amino acid alkylation |
| 1707 | 5.29E-01 | 6.37E-01 | mesoderm formation |
| 2263 | 5.29E-01 | 6.37E-01 | cell activation involved in immune response |
| 2366 | 5.29E-01 | 6.37E-01 | leukocyte activation involved in immune response |
| 6479 | 5.29E-01 | 6.37E-01 | protein amino acid methylation |
| 51701 | 5.29E-01 | 6.37E-01 | interaction with host |
| 6775 | 5.29E-01 | 6.37E-01 | fat-soluble vitamin metabolic process |
| 3205 | 5.29E-01 | 6.37E-01 | cardiac chamber development |
| 48477 | 5.29E-01 | 6.37E-01 | oogenesis |
| 60828 | 5.29E-01 | 6.37E-01 | regulation of canonical Wnt receptor signaling pathway |
| 42770 | 5.33E-01 | 6.41E-01 | DNA damage response, signal transduction |
| 19953 | 5.34E-01 | 6.42E-01 | sexual reproduction |
| 51781 | 5.38E-01 | 6.46E-01 | positive regulation of cell division |
| 2700 | 5.38E-01 | 6.46E-01 | regulation of production of molecular mediator of immune response |
| 6956 | 5.38E-01 | 6.46E-01 | complement activation |
| 15711 | 5.38E-01 | 6.46E-01 | organic anion transport |
| 7399 | 5.38E-01 | 6.46E-01 | nervous system development |
| 30155 | 5.40E-01 | 6.47E-01 | regulation of cell adhesion |
| 30099 | 5.45E-01 | 6.52E-01 | myeloid cell differentiation |
| 7605 | 5.45E-01 | 6.52E-01 | sensory perception of sound |
| 956 | 5.47E-01 | 6.53E-01 | nuclear-transcribed mRNA catabolic process |
| 6304 | 5.47E-01 | 6.53E-01 | DNA modification |
| 2429 | 5.47E-01 | 6.53E-01 | immune response-activating cell surface receptor signaling pathway |
| 2541 | 5.47E-01 | 6.53E-01 | activation of plasma proteins involved in acute inflammatory response |
| 31424 | 5.47E-01 | 6.53E-01 | keratinization |
| 7266 | 5.47E-01 | 6.53E-01 | Rho protein signal transduction |
| 48332 | 5.47E-01 | 6.53E-01 | mesoderm morphogenesis |
| 2253 | 5.50E-01 | 6.56E-01 | activation of immune response |
| 51640 | 5.50E-01 | 6.56E-01 | organelle localization |
| 43623 | 5.54E-01 | 6.59E-01 | cellular protein complex assembly |
| 10876 | 5.54E-01 | 6.59E-01 | lipid localization |
| 33044 | 5.56E-01 | 6.59E-01 | regulation of chromosome organization |
| 70507 | 5.56E-01 | 6.59E-01 | regulation of microtubule cytoskeleton organization |
| 42102 | 5.56E-01 | 6.59E-01 | positive regulation of T cell proliferation |
| 50868 | 5.56E-01 | 6.59E-01 | negative regulation of T cell activation |
| 22404 | 5.56E-01 | 6.59E-01 | molting cycle process |
| 22405 | 5.56E-01 | 6.59E-01 | hair cycle process |
| 1942 | 5.56E-01 | 6.59E-01 | hair follicle development |
| 6096 | 5.56E-01 | 6.59E-01 | glycolysis |
| 31348 | 5.56E-01 | 6.59E-01 | negative regulation of defense response |
| 32409 | 5.56E-01 | 6.59E-01 | regulation of transporter activity |
| 32940 | 5.57E-01 | 6.60E-01 | secretion by cell |
| 48511 | 5.58E-01 | 6.61E-01 | rhythmic process |
| 6917 | 5.60E-01 | 6.63E-01 | induction of apoptosis |
| 12502 | 5.64E-01 | 6.66E-01 | induction of programmed cell death |
| 45766 | 5.64E-01 | 6.66E-01 | positive regulation of angiogenesis |
| 9267 | 5.64E-01 | 6.66E-01 | cellular response to starvation |
| 46148 | 5.64E-01 | 6.66E-01 | pigment biosynthetic process |
| 30218 | 5.64E-01 | 6.66E-01 | erythrocyte differentiation |
| 6120 | 5.64E-01 | 6.66E-01 | mitochondrial electron transport, NADH to ubiquinone |
| 31589 | 5.67E-01 | 6.69E-01 | cell-substrate adhesion |
| 48856 | 5.70E-01 | 6.72E-01 | anatomical structure development |
| 35107 | 5.73E-01 | 6.72E-01 | appendage morphogenesis |
| 35108 | 5.73E-01 | 6.72E-01 | limb morphogenesis |
| 45732 | 5.73E-01 | 6.72E-01 | positive regulation of protein catabolic process |
| 42303 | 5.73E-01 | 6.72E-01 | molting cycle |
| 46489 | 5.73E-01 | 6.72E-01 | phosphoinositide biosynthetic process |
| 42633 | 5.73E-01 | 6.72E-01 | hair cycle |
| 1816 | 5.73E-01 | 6.72E-01 | cytokine production |
| 48232 | 5.73E-01 | 6.72E-01 | male gamete generation |
| 7283 | 5.73E-01 | 6.72E-01 | spermatogenesis |
| 9893 | 5.77E-01 | 6.77E-01 | positive regulation of metabolic process |
| 50954 | 5.78E-01 | 6.78E-01 | sensory perception of mechanical stimulus |
| 31325 | 5.79E-01 | 6.79E-01 | positive regulation of cellular metabolic process |
| 32583 | 5.80E-01 | 6.79E-01 | regulation of gene-specific transcription |
| 22607 | 5.80E-01 | 6.79E-01 | cellular component assembly |
| 51101 | 5.80E-01 | 6.79E-01 | regulation of DNA binding |
| 2768 | 5.81E-01 | 6.79E-01 | immune response-regulating cell surface receptor signaling pathway |
| 10627 | 5.85E-01 | 6.84E-01 | regulation of intracellular protein kinase cascade |
| 1819 | 5.89E-01 | 6.87E-01 | positive regulation of cytokine production |
| 1541 | 5.89E-01 | 6.87E-01 | ovarian follicle development |
| 1704 | 5.89E-01 | 6.87E-01 | formation of primary germ layer |
| 14075 | 5.89E-01 | 6.87E-01 | response to amine stimulus |
| 90101 | 5.89E-01 | 6.87E-01 | negative regulation of transmembrane receptor protein serine/threonine kinase signaling pathway |
| 8283 | 5.91E-01 | 6.89E-01 | cell proliferation |
| 60173 | 5.94E-01 | 6.91E-01 | limb development |
| 48736 | 5.94E-01 | 6.91E-01 | appendage development |
| 9314 | 5.94E-01 | 6.91E-01 | response to radiation |
| 50953 | 5.94E-01 | 6.91E-01 | sensory perception of light stimulus |
| 7601 | 5.94E-01 | 6.91E-01 | visual perception |
| 271 | 5.97E-01 | 6.93E-01 | polysaccharide biosynthetic process |
| 72175 | 5.97E-01 | 6.93E-01 | epithelial tube formation |
| 15718 | 5.97E-01 | 6.93E-01 | monocarboxylic acid transport |
| 43408 | 5.97E-01 | 6.93E-01 | regulation of MAPKKK cascade |
| 45580 | 6.04E-01 | 7.01E-01 | regulation of T cell differentiation |
| 7169 | 6.05E-01 | 7.02E-01 | transmembrane receptor protein tyrosine kinase signaling pathway |
| 7517 | 6.05E-01 | 7.02E-01 | muscle organ development |
| 40011 | 6.07E-01 | 7.03E-01 | locomotion |
| 51302 | 6.12E-01 | 7.09E-01 | regulation of cell division |
| 6402 | 6.12E-01 | 7.09E-01 | mRNA catabolic process |
| 7050 | 6.15E-01 | 7.11E-01 | cell cycle arrest |
| 45595 | 6.16E-01 | 7.12E-01 | regulation of cell differentiation |
| 32886 | 6.19E-01 | 7.13E-01 | regulation of microtubule-based process |
| 8344 | 6.19E-01 | 7.13E-01 | adult locomotory behavior |
| 21543 | 6.19E-01 | 7.13E-01 | pallium development |
| 30307 | 6.19E-01 | 7.13E-01 | positive regulation of cell growth |
| 35148 | 6.19E-01 | 7.13E-01 | tube formation |
| 2706 | 6.19E-01 | 7.13E-01 | regulation of lymphocyte mediated immunity |
| 15698 | 6.19E-01 | 7.13E-01 | inorganic anion transport |
| 48638 | 6.19E-01 | 7.13E-01 | regulation of developmental growth |
| 16202 | 6.19E-01 | 7.13E-01 | regulation of striated muscle tissue development |
| 51099 | 6.20E-01 | 7.13E-01 | positive regulation of binding |
| 30855 | 6.26E-01 | 7.19E-01 | epithelial cell differentiation |
| 45834 | 6.27E-01 | 7.19E-01 | positive regulation of lipid metabolic process |
| 42108 | 6.27E-01 | 7.19E-01 | positive regulation of cytokine biosynthetic process |
| 51250 | 6.27E-01 | 7.19E-01 | negative regulation of lymphocyte activation |
| 48634 | 6.27E-01 | 7.19E-01 | regulation of muscle organ development |
| 32313 | 6.27E-01 | 7.19E-01 | regulation of Rab GTPase activity |
| 32483 | 6.27E-01 | 7.19E-01 | regulation of Rab protein signal transduction |
| 6887 | 6.29E-01 | 7.22E-01 | exocytosis |
| 7093 | 6.34E-01 | 7.26E-01 | mitotic cell cycle checkpoint |
| 48610 | 6.34E-01 | 7.26E-01 | reproductive cellular process |
| 42472 | 6.41E-01 | 7.33E-01 | inner ear morphogenesis |
| 6007 | 6.41E-01 | 7.33E-01 | glucose catabolic process |
| 10741 | 6.41E-01 | 7.33E-01 | negative regulation of intracellular protein kinase cascade |
| 6508 | 6.43E-01 | 7.35E-01 | proteolysis |
| 6793 | 6.44E-01 | 7.36E-01 | phosphorus metabolic process |
| 6796 | 6.44E-01 | 7.36E-01 | phosphate metabolic process |
| 30178 | 6.48E-01 | 7.38E-01 | negative regulation of Wnt receptor signaling pathway |
| 43473 | 6.48E-01 | 7.38E-01 | pigmentation |
| 10810 | 6.48E-01 | 7.38E-01 | regulation of cell-substrate adhesion |
| 31331 | 6.48E-01 | 7.38E-01 | positive regulation of cellular catabolic process |
| 7623 | 6.48E-01 | 7.38E-01 | circadian rhythm |
| 1503 | 6.53E-01 | 7.44E-01 | ossification |
| 45793 | 6.55E-01 | 7.45E-01 | positive regulation of cell size |
| 51092 | 6.55E-01 | 7.45E-01 | positive regulation of NF-kappaB transcription factor activity |
| 2757 | 6.55E-01 | 7.45E-01 | immune response-activating signal transduction |
| 51789 | 6.58E-01 | 7.48E-01 | response to protein stimulus |
| 45619 | 6.61E-01 | 7.51E-01 | regulation of lymphocyte differentiation |
| 32102 | 6.61E-01 | 7.51E-01 | negative regulation of response to external stimulus |
| 44403 | 6.61E-01 | 7.51E-01 | symbiosis, encompassing mutualism through parasitism |
| 48762 | 6.61E-01 | 7.51E-01 | mesenchymal cell differentiation |
| 42110 | 6.67E-01 | 7.55E-01 | T cell activation |
| 43010 | 6.67E-01 | 7.55E-01 | camera-type eye development |
| 6814 | 6.67E-01 | 7.55E-01 | sodium ion transport |
| 42594 | 6.68E-01 | 7.55E-01 | response to starvation |
| 51291 | 6.68E-01 | 7.55E-01 | protein heterooligomerization |
| 6308 | 6.68E-01 | 7.55E-01 | DNA catabolic process |
| 6892 | 6.68E-01 | 7.55E-01 | post-Golgi vesicle-mediated transport |
| 7229 | 6.68E-01 | 7.55E-01 | integrin-mediated signaling pathway |
| 8360 | 6.74E-01 | 7.62E-01 | regulation of cell shape |
| 1764 | 6.74E-01 | 7.62E-01 | neuron migration |
| 31396 | 6.76E-01 | 7.63E-01 | regulation of protein ubiquitination |
| 32318 | 6.76E-01 | 7.63E-01 | regulation of Ras GTPase activity |
| 77 | 6.80E-01 | 7.66E-01 | DNA damage checkpoint |
| 50808 | 6.80E-01 | 7.66E-01 | synapse organization |
| 10001 | 6.80E-01 | 7.66E-01 | glial cell differentiation |
| 40014 | 6.80E-01 | 7.66E-01 | regulation of multicellular organism growth |
| 32507 | 6.80E-01 | 7.66E-01 | maintenance of protein location in cell |
| 7275 | 6.83E-01 | 7.69E-01 | multicellular organismal development |
| 2764 | 6.86E-01 | 7.72E-01 | immune response-regulating signaling pathway |
| 6928 | 6.89E-01 | 7.75E-01 | cellular component movement |
| 8584 | 6.92E-01 | 7.78E-01 | male gonad development |
| 6968 | 6.92E-01 | 7.78E-01 | cellular defense response |
| 46649 | 6.93E-01 | 7.79E-01 | lymphocyte activation |
| 7423 | 6.95E-01 | 7.80E-01 | sensory organ development |
| 44085 | 6.96E-01 | 7.81E-01 | cellular component biogenesis |
| 6730 | 6.97E-01 | 7.82E-01 | one-carbon metabolic process |
| 60562 | 6.97E-01 | 7.82E-01 | epithelial tube morphogenesis |
| 9411 | 6.98E-01 | 7.82E-01 | response to UV |
| 9749 | 6.98E-01 | 7.82E-01 | response to glucose stimulus |
| 7249 | 6.98E-01 | 7.82E-01 | I-kappaB kinase/NF-kappaB cascade |
| 30216 | 7.04E-01 | 7.87E-01 | keratinocyte differentiation |
| 31570 | 7.04E-01 | 7.87E-01 | DNA integrity checkpoint |
| 19320 | 7.04E-01 | 7.87E-01 | hexose catabolic process |
| 31344 | 7.06E-01 | 7.89E-01 | regulation of cell projection organization |
| 2252 | 7.10E-01 | 7.93E-01 | immune effector process |
| 60485 | 7.10E-01 | 7.93E-01 | mesenchyme development |
| 7519 | 7.15E-01 | 7.98E-01 | skeletal muscle tissue development |
| 60348 | 7.18E-01 | 8.01E-01 | bone development |
| 46365 | 7.21E-01 | 8.02E-01 | monosaccharide catabolic process |
| 30162 | 7.21E-01 | 8.02E-01 | regulation of proteolysis |
| 42471 | 7.21E-01 | 8.02E-01 | ear morphogenesis |
| 34284 | 7.21E-01 | 8.02E-01 | response to monosaccharide stimulus |
| 9746 | 7.21E-01 | 8.02E-01 | response to hexose stimulus |
| 10212 | 7.21E-01 | 8.02E-01 | response to ionizing radiation |
| 32271 | 7.21E-01 | 8.02E-01 | regulation of protein polymerization |
| 45597 | 7.22E-01 | 8.02E-01 | positive regulation of cell differentiation |
| 10604 | 7.22E-01 | 8.02E-01 | positive regulation of macromolecule metabolic process |
| 30336 | 7.26E-01 | 8.06E-01 | negative regulation of cell migration |
| 31346 | 7.26E-01 | 8.06E-01 | positive regulation of cell projection organization |
| 60538 | 7.26E-01 | 8.06E-01 | skeletal muscle organ development |
| 9913 | 7.31E-01 | 8.11E-01 | epidermal cell differentiation |
| 43280 | 7.31E-01 | 8.11E-01 | positive regulation of caspase activity |
| 10952 | 7.31E-01 | 8.11E-01 | positive regulation of peptidase activity |
| 45185 | 7.37E-01 | 8.15E-01 | maintenance of protein location |
| 45785 | 7.37E-01 | 8.15E-01 | positive regulation of cell adhesion |
| 42063 | 7.37E-01 | 8.15E-01 | gliogenesis |
| 16331 | 7.37E-01 | 8.15E-01 | morphogenesis of embryonic epithelium |
| 46474 | 7.42E-01 | 8.19E-01 | glycerophospholipid biosynthetic process |
| 50769 | 7.42E-01 | 8.19E-01 | positive regulation of neurogenesis |
| 9896 | 7.42E-01 | 8.19E-01 | positive regulation of catabolic process |
| 51443 | 7.42E-01 | 8.19E-01 | positive regulation of ubiquitin-protein ligase activity |
| 2460 | 7.42E-01 | 8.19E-01 | adaptive immune response based on somatic recombination of immune receptors built from immunoglobulin superfamily domains |
| 16310 | 7.43E-01 | 8.20E-01 | phosphorylation |
| 1817 | 7.45E-01 | 8.22E-01 | regulation of cytokine production |
| 2250 | 7.47E-01 | 8.23E-01 | adaptive immune response |
| 51656 | 7.47E-01 | 8.23E-01 | establishment of organelle localization |
| 6869 | 7.48E-01 | 8.23E-01 | lipid transport |
| 7156 | 7.48E-01 | 8.23E-01 | homophilic cell adhesion |
| 9057 | 7.50E-01 | 8.25E-01 | macromolecule catabolic process |
| 50793 | 7.51E-01 | 8.25E-01 | regulation of developmental process |
| 51271 | 7.51E-01 | 8.25E-01 | negative regulation of cellular component movement |
| 6401 | 7.51E-01 | 8.25E-01 | RNA catabolic process |
| 43414 | 7.51E-01 | 8.25E-01 | macromolecule methylation |
| 40013 | 7.51E-01 | 8.25E-01 | negative regulation of locomotion |
| 32259 | 7.51E-01 | 8.25E-01 | methylation |
| 51351 | 7.56E-01 | 8.29E-01 | positive regulation of ligase activity |
| 9615 | 7.58E-01 | 8.31E-01 | response to virus |
| 42113 | 7.61E-01 | 8.33E-01 | B cell activation |
| 42176 | 7.61E-01 | 8.33E-01 | regulation of protein catabolic process |
| 9581 | 7.61E-01 | 8.33E-01 | detection of external stimulus |
| 16570 | 7.62E-01 | 8.33E-01 | histone modification |
| 51493 | 7.62E-01 | 8.33E-01 | regulation of cytoskeleton organization |
| 7389 | 7.64E-01 | 8.36E-01 | pattern specification process |
| 21953 | 7.66E-01 | 8.36E-01 | central nervous system neuron differentiation |
| 1508 | 7.66E-01 | 8.36E-01 | regulation of action potential |
| 6839 | 7.66E-01 | 8.36E-01 | mitochondrial transport |
| 51056 | 7.67E-01 | 8.37E-01 | regulation of small GTPase mediated signal transduction |
| 16569 | 7.72E-01 | 8.42E-01 | covalent chromatin modification |
| 48598 | 7.73E-01 | 8.43E-01 | embryonic morphogenesis |
| 42035 | 7.74E-01 | 8.43E-01 | regulation of cytokine biosynthetic process |
| 35270 | 7.74E-01 | 8.43E-01 | endocrine system development |
| 6959 | 7.74E-01 | 8.43E-01 | humoral immune response |
| 7015 | 7.74E-01 | 8.43E-01 | actin filament organization |
| 1654 | 7.75E-01 | 8.43E-01 | eye development |
| 30384 | 7.79E-01 | 8.47E-01 | phosphoinositide metabolic process |
| 31397 | 7.79E-01 | 8.47E-01 | negative regulation of protein ubiquitination |
| 33157 | 7.83E-01 | 8.50E-01 | regulation of intracellular protein transport |
| 46777 | 7.83E-01 | 8.50E-01 | protein amino acid autophosphorylation |
| 31099 | 7.83E-01 | 8.50E-01 | regeneration |
| 6417 | 7.84E-01 | 8.51E-01 | regulation of translation |
| 7369 | 7.87E-01 | 8.54E-01 | gastrulation |
| 43632 | 7.91E-01 | 8.57E-01 | modification-dependent macromolecule catabolic process |
| 19941 | 7.91E-01 | 8.57E-01 | modification-dependent protein catabolic process |
| 51438 | 7.91E-01 | 8.57E-01 | regulation of ubiquitin-protein ligase activity |
| 51091 | 7.95E-01 | 8.60E-01 | positive regulation of transcription factor activity |
| 48839 | 7.95E-01 | 8.60E-01 | inner ear development |
| 90047 | 7.95E-01 | 8.60E-01 | positive regulation of transcription regulator activity |
| 7507 | 7.98E-01 | 8.63E-01 | heart development |
| 122 | 8.00E-01 | 8.64E-01 | negative regulation of transcription from RNA polymerase II promoter |
| 51340 | 8.03E-01 | 8.67E-01 | regulation of ligase activity |
| 10720 | 8.03E-01 | 8.67E-01 | positive regulation of cell development |
| 7218 | 8.07E-01 | 8.71E-01 | neuropeptide signaling pathway |
| 30278 | 8.11E-01 | 8.75E-01 | regulation of ossification |
| 51128 | 8.12E-01 | 8.75E-01 | regulation of cellular component organization |
| 30111 | 8.14E-01 | 8.77E-01 | regulation of Wnt receptor signaling pathway |
| 30326 | 8.14E-01 | 8.77E-01 | embryonic limb morphogenesis |
| 35113 | 8.14E-01 | 8.77E-01 | embryonic appendage morphogenesis |
| 43009 | 8.22E-01 | 8.85E-01 | chordate embryonic development |
| 7281 | 8.25E-01 | 8.88E-01 | germ cell development |
| 30097 | 8.26E-01 | 8.88E-01 | hemopoiesis |
| 45596 | 8.28E-01 | 8.90E-01 | negative regulation of cell differentiation |
| 46578 | 8.28E-01 | 8.90E-01 | regulation of Ras protein signal transduction |
| 7586 | 8.28E-01 | 8.90E-01 | digestion |
| 9792 | 8.29E-01 | 8.90E-01 | embryonic development ending in birth or egg hatching |
| 31327 | 8.31E-01 | 8.92E-01 | negative regulation of cellular biosynthetic process |
| 90092 | 8.31E-01 | 8.92E-01 | regulation of transmembrane receptor protein serine/threonine kinase signaling pathway |
| 9891 | 8.31E-01 | 8.92E-01 | positive regulation of biosynthetic process |
| 43388 | 8.35E-01 | 8.95E-01 | positive regulation of DNA binding |
| 42330 | 8.37E-01 | 8.96E-01 | taxis |
| 6935 | 8.37E-01 | 8.96E-01 | chemotaxis |
| 65007 | 8.38E-01 | 8.96E-01 | biological regulation |
| 32956 | 8.38E-01 | 8.96E-01 | regulation of actin cytoskeleton organization |
| 43254 | 8.38E-01 | 8.96E-01 | regulation of protein complex assembly |
| 6457 | 8.39E-01 | 8.97E-01 | protein folding |
| 31398 | 8.41E-01 | 8.99E-01 | positive regulation of protein ubiquitination |
| 10608 | 8.43E-01 | 9.00E-01 | posttranscriptional regulation of gene expression |
| 9890 | 8.44E-01 | 9.01E-01 | negative regulation of biosynthetic process |
| 33043 | 8.45E-01 | 9.02E-01 | regulation of organelle organization |
| 32386 | 8.47E-01 | 9.04E-01 | regulation of intracellular transport |
| 31175 | 8.49E-01 | 9.05E-01 | neuron projection development |
| 16568 | 8.49E-01 | 9.05E-01 | chromatin modification |
| 51603 | 8.49E-01 | 9.05E-01 | proteolysis involved in cellular protein catabolic process |
| 32970 | 8.50E-01 | 9.05E-01 | regulation of actin filament-based process |
| 44265 | 8.51E-01 | 9.06E-01 | cellular macromolecule catabolic process |
| 10639 | 8.53E-01 | 9.07E-01 | negative regulation of organelle organization |
| 45786 | 8.53E-01 | 9.07E-01 | negative regulation of cell cycle |
| 6915 | 8.53E-01 | 9.07E-01 | apoptosis |
| 44257 | 8.54E-01 | 9.08E-01 | cellular protein catabolic process |
| 51129 | 8.55E-01 | 9.09E-01 | negative regulation of cellular component organization |
| 43583 | 8.58E-01 | 9.11E-01 | ear development |
| 16044 | 8.59E-01 | 9.11E-01 | cellular membrane organization |
| 51726 | 8.59E-01 | 9.11E-01 | regulation of cell cycle |
| 61024 | 8.60E-01 | 9.12E-01 | membrane organization |
| 12501 | 8.66E-01 | 9.18E-01 | programmed cell death |
| 35023 | 8.69E-01 | 9.21E-01 | regulation of Rho protein signal transduction |
| 6464 | 8.70E-01 | 9.21E-01 | protein modification process |
| 10975 | 8.72E-01 | 9.23E-01 | regulation of neuron projection development |
| 48666 | 8.73E-01 | 9.24E-01 | neuron development |
| 75 | 8.74E-01 | 9.24E-01 | cell cycle checkpoint |
| 48534 | 8.76E-01 | 9.26E-01 | hemopoietic or lymphoid organ development |
| 43406 | 8.76E-01 | 9.26E-01 | positive regulation of MAP kinase activity |
| 31328 | 8.87E-01 | 9.37E-01 | positive regulation of cellular biosynthetic process |
| 43412 | 8.87E-01 | 9.37E-01 | macromolecule modification |
| 31324 | 8.91E-01 | 9.41E-01 | negative regulation of cellular metabolic process |
| 30163 | 8.92E-01 | 9.41E-01 | protein catabolic process |
| 17038 | 8.96E-01 | 9.45E-01 | protein import |
| 42060 | 8.97E-01 | 9.45E-01 | wound healing |
| 50890 | 8.99E-01 | 9.47E-01 | cognition |
| 2520 | 9.00E-01 | 9.47E-01 | immune system development |
| 6605 | 9.00E-01 | 9.47E-01 | protein targeting |
| 6511 | 9.02E-01 | 9.49E-01 | ubiquitin-dependent protein catabolic process |
| 6650 | 9.04E-01 | 9.51E-01 | glycerophospholipid metabolic process |
| 9892 | 9.05E-01 | 9.52E-01 | negative regulation of metabolic process |
| 33554 | 9.10E-01 | 9.56E-01 | cellular response to stress |
| 2521 | 9.15E-01 | 9.61E-01 | leukocyte differentiation |
| 51093 | 9.17E-01 | 9.63E-01 | negative regulation of developmental process |
| 16337 | 9.19E-01 | 9.65E-01 | cell-cell adhesion |
| 48468 | 9.20E-01 | 9.66E-01 | cell development |
| 1701 | 9.21E-01 | 9.66E-01 | in utero embryonic development |
| 48193 | 9.23E-01 | 9.67E-01 | Golgi vesicle transport |
| 22008 | 9.24E-01 | 9.68E-01 | neurogenesis |
| 16071 | 9.25E-01 | 9.69E-01 | mRNA metabolic process |
| 10324 | 9.28E-01 | 9.71E-01 | membrane invagination |
| 6897 | 9.28E-01 | 9.71E-01 | endocytosis |
| 30182 | 9.28E-01 | 9.71E-01 | neuron differentiation |
| 45165 | 9.28E-01 | 9.71E-01 | cell fate commitment |
| 22603 | 9.30E-01 | 9.72E-01 | regulation of anatomical structure morphogenesis |
| 51960 | 9.33E-01 | 9.75E-01 | regulation of nervous system development |
| 6259 | 9.33E-01 | 9.75E-01 | DNA metabolic process |
| 50794 | 9.36E-01 | 9.77E-01 | regulation of cellular process |
| 10558 | 9.40E-01 | 9.80E-01 | negative regulation of macromolecule biosynthetic process |
| 51223 | 9.40E-01 | 9.80E-01 | regulation of protein transport |
| 48699 | 9.40E-01 | 9.80E-01 | generation of neurons |
| 50878 | 9.41E-01 | 9.80E-01 | regulation of body fluid levels |
| 45944 | 9.41E-01 | 9.81E-01 | positive regulation of transcription from RNA polymerase II promoter |
| 9952 | 9.42E-01 | 9.81E-01 | anterior/posterior pattern formation |
| 34622 | 9.43E-01 | 9.82E-01 | cellular macromolecular complex assembly |
| 22604 | 9.44E-01 | 9.82E-01 | regulation of cell morphogenesis |
| 9790 | 9.45E-01 | 9.82E-01 | embryonic development |
| 6974 | 9.47E-01 | 9.85E-01 | response to DNA damage stimulus |
| 45892 | 9.48E-01 | 9.85E-01 | negative regulation of transcription, DNA-dependent |
| 70201 | 9.49E-01 | 9.85E-01 | regulation of establishment of protein localization |
| 51253 | 9.53E-01 | 9.89E-01 | negative regulation of RNA metabolic process |
| 6325 | 9.58E-01 | 9.94E-01 | chromatin organization |
| 16481 | 9.62E-01 | 9.98E-01 | negative regulation of transcription |
| 45664 | 9.63E-01 | 9.98E-01 | regulation of neuron differentiation |
| 50789 | 9.64E-01 | 9.99E-01 | regulation of biological process |
| 32880 | 9.64E-01 | 9.99E-01 | regulation of protein localization |
| 7346 | 9.66E-01 | 1 | regulation of mitotic cell cycle |
| 30154 | 9.67E-01 | 1 | cell differentiation |
| 7409 | 9.68E-01 | 1 | axonogenesis |
| 34621 | 9.68E-01 | 1 | cellular macromolecular complex subunit organization |
| 6468 | 9.70E-01 | 1 | protein amino acid phosphorylation |
| 30030 | 9.71E-01 | 1 | cell projection organization |
| 10605 | 9.73E-01 | 1 | negative regulation of macromolecule metabolic process |
| 6260 | 9.73E-01 | 1 | DNA replication |
| 8380 | 9.75E-01 | 1 | RNA splicing |
| 48667 | 9.76E-01 | 1 | cell morphogenesis involved in neuron differentiation |
| 10628 | 9.76E-01 | 1 | positive regulation of gene expression |
| 10557 | 9.76E-01 | 1 | positive regulation of macromolecule biosynthetic process |
| 6357 | 9.77E-01 | 1 | regulation of transcription from RNA polymerase II promoter |
| 51173 | 9.77E-01 | 1 | positive regulation of nitrogen compound metabolic process |
| 48869 | 9.77E-01 | 1 | cellular developmental process |
| 19538 | 9.78E-01 | 1 | protein metabolic process |
| 278 | 9.78E-01 | 1 | mitotic cell cycle |
| 48812 | 9.79E-01 | 1 | neuron projection morphogenesis |
| 6281 | 9.80E-01 | 1 | DNA repair |
| 10629 | 9.82E-01 | 1 | negative regulation of gene expression |
| 6397 | 9.83E-01 | 1 | mRNA processing |
| 50767 | 9.83E-01 | 1 | regulation of neurogenesis |
| 45934 | 9.83E-01 | 1 | negative regulation of nucleobase, nucleoside, nucleotide and nucleic acid metabolic process |
| 3002 | 9.83E-01 | 1 | regionalization |
| 51172 | 9.84E-01 | 1 | negative regulation of nitrogen compound metabolic process |
| 43687 | 9.86E-01 | 1 | post-translational protein modification |
| 44419 | 9.87E-01 | 1 | interspecies interaction between organisms |
| 45893 | 9.87E-01 | 1 | positive regulation of transcription, DNA-dependent |
| 51254 | 9.88E-01 | 1 | positive regulation of RNA metabolic process |
| 48858 | 9.89E-01 | 1 | cell projection morphogenesis |
| 16192 | 9.89E-01 | 1 | vesicle-mediated transport |
| 30036 | 9.90E-01 | 1 | actin cytoskeleton organization |
| 22403 | 9.91E-01 | 1 | cell cycle phase |
| 904 | 9.91E-01 | 1 | cell morphogenesis involved in differentiation |
| 48285 | 9.91E-01 | 1 | organelle fission |
| 51276 | 9.91E-01 | 1 | chromosome organization |
| 32990 | 9.91E-01 | 1 | cell part morphogenesis |
| 51641 | 9.92E-01 | 1 | cellular localization |
| 7017 | 9.92E-01 | 1 | microtubule-based process |
| 30029 | 9.92E-01 | 1 | actin filament-based process |
| 60284 | 9.92E-01 | 1 | regulation of cell development |
| 44267 | 9.92E-01 | 1 | cellular protein metabolic process |
| 6886 | 9.93E-01 | 1 | intracellular protein transport |
| 7155 | 9.94E-01 | 1 | cell adhesion |
| 22610 | 9.94E-01 | 1 | biological adhesion |
| 51649 | 9.94E-01 | 1 | establishment of localization in cell |
| 6996 | 9.96E-01 | 1 | organelle organization |
| 7600 | 9.96E-01 | 1 | sensory perception |
| 45941 | 9.96E-01 | 1 | positive regulation of transcription |
| 22402 | 9.96E-01 | 1 | cell cycle process |
| 15031 | 9.97E-01 | 1 | protein transport |
| 16043 | 9.97E-01 | 1 | cellular component organization |
| 8104 | 9.97E-01 | 1 | protein localization |
| 45184 | 9.97E-01 | 1 | establishment of protein localization |
| 902 | 9.98E-01 | 1 | cell morphogenesis |
| 34613 | 9.98E-01 | 1 | cellular protein localization |
| 70727 | 9.98E-01 | 1 | cellular macromolecule localization |
| 33036 | 9.98E-01 | 1 | macromolecule localization |
| 9059 | 9.99E-01 | 1 | macromolecule biosynthetic process |
| 45935 | 9.99E-01 | 1 | positive regulation of nucleobase, nucleoside, nucleotide and nucleic acid metabolic process |
| 32989 | 9.99E-01 | 1 | cellular component morphogenesis |
| 46907 | 9.99E-01 | 1 | intracellular transport |
| 34645 | 9.99E-01 | 1 | cellular macromolecule biosynthetic process |
| 7049 | 9.99E-01 | 1 | cell cycle |
| 6396 | 1.00E+00 | 1 | RNA processing |
| 7010 | 1.00E+00 | 1 | cytoskeleton organization |
| 43170 | 1.00E+00 | 1 | macromolecule metabolic process |
| 19222 | 1.00E+00 | 1 | regulation of metabolic process |
| 44260 | 1.00E+00 | 1 | cellular macromolecule metabolic process |
| 31323 | 1.00E+00 | 1 | regulation of cellular metabolic process |
| 90304 | 1.00E+00 | 1 | nucleic acid metabolic process |
| 9889 | 1.00E+00 | 1 | regulation of biosynthetic process |
| 16070 | 1.00E+00 | 1 | RNA metabolic process |
| 31326 | 1 | 1 | regulation of cellular biosynthetic process |
| 80090 | 1 | 1 | regulation of primary metabolic process |
| 51171 | 1 | 1 | regulation of nitrogen compound metabolic process |
| 19219 | 1 | 1 | regulation of nucleobase, nucleoside, nucleotide and nucleic acid metabolic process |
| 10467 | 1 | 1 | gene expression |
| 10468 | 1 | 1 | regulation of gene expression |
| 10556 | 1 | 1 | regulation of macromolecule biosynthetic process |
| 51252 | 1 | 1 | regulation of RNA metabolic process |
| 60255 | 1 | 1 | regulation of macromolecule metabolic process |
| 45449 | 1 | 1 | regulation of transcription |
| 6355 | 1 | 1 | regulation of transcription, DNA-dependent |
| 8150 | 1 | 1 | biological_process |
