# Supplementary material for: Increased transcriptional and metabolic capacity for lipid metabolism in the peripheral zone of the prostate may underpin its increased susceptibility to cancer
Source: Oncotarget. 2017 May 17;8(49):84902–16. doi: 10.18632/oncotarget.17926 (PMC5689582; doi:10.18632/oncotarget.17926)
Supplement: Supplementary file 5 [file oncotarget-08-84902-s005.docx]

**Supplementary Table 4: Overrepresentation of Gene Ontology terms within differentially expressed genes.**

| **GO-ID** | **p-value** | **corr p-value** | **Description** |
| --- | --- | --- | --- |
| 42221 | 2.57E-29 | 1.25E-25 | response to chemical stimulus |
| 10033 | 1.04E-25 | 2.53E-22 | response to organic substance |
| 65008 | 3.59E-22 | 5.83E-19 | regulation of biological quality |
| 23052 | 7.98E-22 | 9.72E-19 | signaling |
| 9987 | 3.32E-21 | 2.57E-18 | cellular process |
| 32879 | 3.60E-21 | 2.57E-18 | regulation of localization |
| 48731 | 3.69E-21 | 2.57E-18 | system development |
| 7155 | 2.32E-20 | 1.42E-17 | cell adhesion |
| 22610 | 2.65E-20 | 1.43E-17 | biological adhesion |
| 9725 | 4.30E-20 | 2.09E-17 | response to hormone stimulus |
| 9719 | 1.95E-19 | 8.61E-17 | response to endogenous stimulus |
| 48856 | 2.82E-19 | 1.15E-16 | anatomical structure development |
| 51270 | 1.73E-18 | 6.50E-16 | regulation of cellular component movement |
| 48513 | 2.93E-18 | 1.02E-15 | organ development |
| 9611 | 1.05E-17 | 3.41E-15 | response to wounding |
| 51239 | 3.35E-17 | 1.02E-14 | regulation of multicellular organismal process |
| 7275 | 3.85E-17 | 1.10E-14 | multicellular organismal development |
| 32502 | 4.51E-17 | 1.22E-14 | developmental process |
| 23046 | 7.30E-17 | 1.78E-14 | signaling process |
| 23060 | 7.30E-17 | 1.78E-14 | signal transmission |
| 7165 | 9.13E-17 | 2.12E-14 | signal transduction |
| 44281 | 1.51E-16 | 3.35E-14 | small molecule metabolic process |
| 48545 | 3.08E-16 | 6.53E-14 | response to steroid hormone stimulus |
| 40012 | 7.97E-16 | 1.62E-13 | regulation of locomotion |
| 48518 | 3.26E-15 | 6.35E-13 | positive regulation of biological process |
| 30334 | 1.42E-14 | 2.66E-12 | regulation of cell migration |
| 42592 | 2.46E-14 | 4.44E-12 | homeostatic process |
| 9653 | 5.86E-14 | 1.02E-11 | anatomical structure morphogenesis |
| 32101 | 6.15E-14 | 1.03E-11 | regulation of response to external stimulus |
| 1568 | 9.07E-14 | 1.47E-11 | blood vessel development |
| 1944 | 1.12E-13 | 1.75E-11 | vasculature development |
| 10646 | 1.15E-13 | 1.76E-11 | regulation of cell communication |
| 7166 | 1.83E-13 | 2.69E-11 | cell surface receptor linked signaling pathway |
| 23033 | 1.92E-13 | 2.75E-11 | signaling pathway |
| 48522 | 2.71E-13 | 3.77E-11 | positive regulation of cellular process |
| 51272 | 3.02E-13 | 4.09E-11 | positive regulation of cellular component movement |
| 7610 | 3.65E-13 | 4.80E-11 | behavior |
| 50896 | 4.60E-13 | 5.90E-11 | response to stimulus |
| 44057 | 8.06E-13 | 1.01E-10 | regulation of system process |
| 51128 | 1.03E-12 | 1.26E-10 | regulation of cellular component organization |
| 32501 | 1.09E-12 | 1.29E-10 | multicellular organismal process |
| 48514 | 1.73E-12 | 2.00E-10 | blood vessel morphogenesis |
| 2682 | 2.25E-12 | 2.55E-10 | regulation of immune system process |
| 43067 | 2.66E-12 | 2.94E-10 | regulation of programmed cell death |
| 50793 | 2.85E-12 | 3.08E-10 | regulation of developmental process |
| 48583 | 2.91E-12 | 3.08E-10 | regulation of response to stimulus |
| 9605 | 4.37E-12 | 4.53E-10 | response to external stimulus |
| 10941 | 4.82E-12 | 4.89E-10 | regulation of cell death |
| 2376 | 5.25E-12 | 5.19E-10 | immune system process |
| 6629 | 5.32E-12 | 5.19E-10 | lipid metabolic process |
| 42981 | 6.01E-12 | 5.74E-10 | regulation of apoptosis |
| 6950 | 6.68E-12 | 6.26E-10 | response to stress |
| 19725 | 7.33E-12 | 6.74E-10 | cellular homeostasis |
| 16337 | 2.20E-11 | 1.99E-09 | cell-cell adhesion |
| 40017 | 2.87E-11 | 2.54E-09 | positive regulation of locomotion |
| 2684 | 2.95E-11 | 2.57E-09 | positive regulation of immune system process |
| 6928 | 4.11E-11 | 3.51E-09 | cellular component movement |
| 30335 | 6.16E-11 | 5.16E-09 | positive regulation of cell migration |
| 50865 | 6.25E-11 | 5.16E-09 | regulation of cell activation |
| 50801 | 7.10E-11 | 5.77E-09 | ion homeostasis |
| 65007 | 7.31E-11 | 5.83E-09 | biological regulation |
| 40011 | 8.24E-11 | 6.48E-09 | locomotion |
| 6468 | 8.38E-11 | 6.48E-09 | protein amino acid phosphorylation |
| 48878 | 8.71E-11 | 6.63E-09 | chemical homeostasis |
| 43062 | 9.11E-11 | 6.83E-09 | extracellular structure organization |
| 6082 | 1.69E-10 | 1.25E-08 | organic acid metabolic process |
| 43436 | 1.94E-10 | 1.37E-08 | oxoacid metabolic process |
| 19752 | 1.94E-10 | 1.37E-08 | carboxylic acid metabolic process |
| 55065 | 1.94E-10 | 1.37E-08 | metal ion homeostasis |
| 30198 | 2.02E-10 | 1.41E-08 | extracellular matrix organization |
| 6954 | 2.06E-10 | 1.41E-08 | inflammatory response |
| 7167 | 2.19E-10 | 1.48E-08 | enzyme linked receptor protein signaling pathway |
| 55080 | 2.35E-10 | 1.56E-08 | cation homeostasis |
| 51179 | 2.63E-10 | 1.73E-08 | localization |
| 22603 | 2.73E-10 | 1.77E-08 | regulation of anatomical structure morphogenesis |
| 48519 | 3.43E-10 | 2.17E-08 | negative regulation of biological process |
| 30155 | 3.43E-10 | 2.17E-08 | regulation of cell adhesion |
| 10035 | 4.34E-10 | 2.71E-08 | response to inorganic substance |
| 70482 | 7.04E-10 | 4.34E-08 | response to oxygen levels |
| 42180 | 7.33E-10 | 4.46E-08 | cellular ketone metabolic process |
| 8284 | 7.42E-10 | 4.46E-08 | positive regulation of cell proliferation |
| 1525 | 7.86E-10 | 4.67E-08 | angiogenesis |
| 6875 | 1.26E-09 | 7.39E-08 | cellular metal ion homeostasis |
| 6066 | 1.36E-09 | 7.88E-08 | alcohol metabolic process |
| 55082 | 1.42E-09 | 8.15E-08 | cellular chemical homeostasis |
| 6952 | 1.46E-09 | 8.24E-08 | defense response |
| 1666 | 1.61E-09 | 8.99E-08 | response to hypoxia |
| 35556 | 1.71E-09 | 9.46E-08 | intracellular signal transduction |
| 6873 | 1.78E-09 | 9.76E-08 | cellular ion homeostasis |
| 51094 | 1.94E-09 | 1.05E-07 | positive regulation of developmental process |
| 44283 | 2.03E-09 | 1.09E-07 | small molecule biosynthetic process |
| 16043 | 2.10E-09 | 1.11E-07 | cellular component organization |
| 7626 | 2.50E-09 | 1.30E-07 | locomotory behavior |
| 9887 | 2.50E-09 | 1.30E-07 | organ morphogenesis |
| 7611 | 2.55E-09 | 1.31E-07 | learning or memory |
| 51049 | 2.81E-09 | 1.43E-07 | regulation of transport |
| 7229 | 2.88E-09 | 1.45E-07 | integrin-mediated signaling pathway |
| 50727 | 3.21E-09 | 1.60E-07 | regulation of inflammatory response |
| 51241 | 4.01E-09 | 1.97E-07 | negative regulation of multicellular organismal process |
| 42127 | 4.09E-09 | 1.99E-07 | regulation of cell proliferation |
| 32496 | 5.10E-09 | 2.46E-07 | response to lipopolysaccharide |
| 30036 | 5.55E-09 | 2.60E-07 | actin cytoskeleton organization |
| 6519 | 5.57E-09 | 2.60E-07 | cellular amino acid and derivative metabolic process |
| 48523 | 5.57E-09 | 2.60E-07 | negative regulation of cellular process |
| 51240 | 5.60E-09 | 2.60E-07 | positive regulation of multicellular organismal process |
| 30029 | 5.71E-09 | 2.62E-07 | actin filament-based process |
| 42493 | 6.14E-09 | 2.80E-07 | response to drug |
| 55074 | 6.75E-09 | 3.04E-07 | calcium ion homeostasis |
| 32103 | 6.85E-09 | 3.06E-07 | positive regulation of response to external stimulus |
| 6793 | 7.94E-09 | 3.48E-07 | phosphorus metabolic process |
| 6796 | 7.94E-09 | 3.48E-07 | phosphate metabolic process |
| 9612 | 8.42E-09 | 3.66E-07 | response to mechanical stimulus |
| 6575 | 1.12E-08 | 4.83E-07 | cellular amino acid derivative metabolic process |
| 35466 | 1.26E-08 | 5.31E-07 | regulation of signaling pathway |
| 51174 | 1.27E-08 | 5.31E-07 | regulation of phosphorus metabolic process |
| 19220 | 1.27E-08 | 5.31E-07 | regulation of phosphate metabolic process |
| 43627 | 1.33E-08 | 5.52E-07 | response to estrogen stimulus |
| 42325 | 1.62E-08 | 6.70E-07 | regulation of phosphorylation |
| 14070 | 2.07E-08 | 8.43E-07 | response to organic cyclic substance |
| 50804 | 2.08E-08 | 8.43E-07 | regulation of synaptic transmission |
| 6874 | 2.16E-08 | 8.71E-07 | cellular calcium ion homeostasis |
| 30003 | 2.41E-08 | 9.63E-07 | cellular cation homeostasis |
| 48584 | 2.65E-08 | 1.05E-06 | positive regulation of response to stimulus |
| 1775 | 3.11E-08 | 1.21E-06 | cell activation |
| 10647 | 3.12E-08 | 1.21E-06 | positive regulation of cell communication |
| 43069 | 3.48E-08 | 1.35E-06 | negative regulation of programmed cell death |
| 16310 | 3.51E-08 | 1.35E-06 | phosphorylation |
| 10942 | 3.58E-08 | 1.36E-06 | positive regulation of cell death |
| 6909 | 3.91E-08 | 1.48E-06 | phagocytosis |
| 51969 | 3.97E-08 | 1.49E-06 | regulation of transmission of nerve impulse |
| 23034 | 4.24E-08 | 1.58E-06 | intracellular signaling pathway |
| 48646 | 4.38E-08 | 1.62E-06 | anatomical structure formation involved in morphogenesis |
| 44093 | 4.70E-08 | 1.72E-06 | positive regulation of molecular function |
| 43066 | 4.86E-08 | 1.76E-06 | negative regulation of apoptosis |
| 2237 | 5.14E-08 | 1.84E-06 | response to molecule of bacterial origin |
| 31344 | 5.14E-08 | 1.84E-06 | regulation of cell projection organization |
| 43068 | 5.44E-08 | 1.93E-06 | positive regulation of programmed cell death |
| 44255 | 6.21E-08 | 2.19E-06 | cellular lipid metabolic process |
| 7010 | 6.54E-08 | 2.29E-06 | cytoskeleton organization |
| 22604 | 7.05E-08 | 2.44E-06 | regulation of cell morphogenesis |
| 31644 | 7.05E-08 | 2.44E-06 | regulation of neurological system process |
| 30199 | 7.12E-08 | 2.44E-06 | collagen fibril organization |
| 60548 | 7.81E-08 | 2.66E-06 | negative regulation of cell death |
| 51130 | 7.87E-08 | 2.66E-06 | positive regulation of cellular component organization |
| 45595 | 7.94E-08 | 2.67E-06 | regulation of cell differentiation |
| 30154 | 8.58E-08 | 2.86E-06 | cell differentiation |
| 50729 | 8.67E-08 | 2.87E-06 | positive regulation of inflammatory response |
| 43065 | 9.02E-08 | 2.97E-06 | positive regulation of apoptosis |
| 50789 | 1.06E-07 | 3.45E-06 | regulation of biological process |
| 2694 | 1.10E-07 | 3.55E-06 | regulation of leukocyte activation |
| 48869 | 1.10E-07 | 3.55E-06 | cellular developmental process |
| 55066 | 1.14E-07 | 3.66E-06 | di-, tri-valent inorganic cation homeostasis |
| 32787 | 1.18E-07 | 3.74E-06 | monocarboxylic acid metabolic process |
| 31589 | 1.27E-07 | 3.99E-06 | cell-substrate adhesion |
| 3013 | 1.28E-07 | 3.99E-06 | circulatory system process |
| 8015 | 1.28E-07 | 3.99E-06 | blood circulation |
| 7154 | 1.32E-07 | 4.10E-06 | cell communication |
| 44282 | 1.42E-07 | 4.37E-06 | small molecule catabolic process |
| 16477 | 1.46E-07 | 4.47E-06 | cell migration |
| 42330 | 1.59E-07 | 4.82E-06 | taxis |
| 6935 | 1.59E-07 | 4.82E-06 | chemotaxis |
| 50790 | 1.75E-07 | 5.27E-06 | regulation of catalytic activity |
| 8152 | 1.79E-07 | 5.34E-06 | metabolic process |
| 1501 | 1.96E-07 | 5.81E-06 | skeletal system development |
| 50867 | 2.10E-07 | 6.21E-06 | positive regulation of cell activation |
| 9888 | 2.43E-07 | 7.12E-06 | tissue development |
| 10648 | 2.46E-07 | 7.17E-06 | negative regulation of cell communication |
| 8202 | 2.53E-07 | 7.35E-06 | steroid metabolic process |
| 51704 | 2.56E-07 | 7.38E-06 | multi-organism process |
| 43085 | 2.76E-07 | 7.91E-06 | positive regulation of catalytic activity |
| 45597 | 2.81E-07 | 8.00E-06 | positive regulation of cell differentiation |
| 31960 | 2.99E-07 | 8.46E-06 | response to corticosteroid stimulus |
| 51271 | 3.03E-07 | 8.53E-06 | negative regulation of cellular component movement |
| 44087 | 3.15E-07 | 8.81E-06 | regulation of cellular component biogenesis |
| 30030 | 3.30E-07 | 9.18E-06 | cell projection organization |
| 35468 | 3.71E-07 | 1.03E-05 | positive regulation of signaling pathway |
| 51674 | 3.88E-07 | 1.06E-05 | localization of cell |
| 48870 | 3.88E-07 | 1.06E-05 | cell motility |
| 23051 | 4.18E-07 | 1.14E-05 | regulation of signaling process |
| 10038 | 4.43E-07 | 1.20E-05 | response to metal ion |
| 55114 | 4.57E-07 | 1.23E-05 | oxidation reduction |
| 9308 | 4.68E-07 | 1.25E-05 | amine metabolic process |
| 90066 | 4.77E-07 | 1.27E-05 | regulation of anatomical structure size |
| 7584 | 4.82E-07 | 1.28E-05 | response to nutrient |
| 51336 | 5.12E-07 | 1.35E-05 | regulation of hydrolase activity |
| 65009 | 5.39E-07 | 1.41E-05 | regulation of molecular function |
| 80134 | 5.86E-07 | 1.53E-05 | regulation of response to stress |
| 32355 | 6.23E-07 | 1.61E-05 | response to estradiol stimulus |
| 50794 | 6.77E-07 | 1.73E-05 | regulation of cellular process |
| 46394 | 6.80E-07 | 1.73E-05 | carboxylic acid biosynthetic process |
| 16053 | 6.80E-07 | 1.73E-05 | organic acid biosynthetic process |
| 51050 | 7.11E-07 | 1.80E-05 | positive regulation of transport |
| 1932 | 7.22E-07 | 1.82E-05 | regulation of protein amino acid phosphorylation |
| 9991 | 7.96E-07 | 2.00E-05 | response to extracellular stimulus |
| 30005 | 8.44E-07 | 2.11E-05 | cellular di-, tri-valent inorganic cation homeostasis |
| 45321 | 9.73E-07 | 2.41E-05 | leukocyte activation |
| 45860 | 9.73E-07 | 2.41E-05 | positive regulation of protein kinase activity |
| 9966 | 9.87E-07 | 2.43E-05 | regulation of signal transduction |
| 51384 | 1.02E-06 | 2.50E-05 | response to glucocorticoid stimulus |
| 7162 | 1.07E-06 | 2.61E-05 | negative regulation of cell adhesion |
| 33674 | 1.11E-06 | 2.69E-05 | positive regulation of kinase activity |
| 60191 | 1.13E-06 | 2.72E-05 | regulation of lipase activity |
| 44106 | 1.13E-06 | 2.72E-05 | cellular amine metabolic process |
| 45859 | 1.17E-06 | 2.79E-05 | regulation of protein kinase activity |
| 7568 | 1.20E-06 | 2.84E-05 | aging |
| 61041 | 1.20E-06 | 2.84E-05 | regulation of wound healing |
| 50776 | 1.23E-06 | 2.90E-05 | regulation of immune response |
| 7399 | 1.33E-06 | 3.12E-05 | nervous system development |
| 8203 | 1.39E-06 | 3.25E-05 | cholesterol metabolic process |
| 6790 | 1.47E-06 | 3.41E-05 | sulfur metabolic process |
| 31347 | 1.49E-06 | 3.45E-05 | regulation of defense response |
| 31099 | 1.64E-06 | 3.77E-05 | regeneration |
| 70887 | 1.71E-06 | 3.91E-05 | cellular response to chemical stimulus |
| 16126 | 1.87E-06 | 4.27E-05 | sterol biosynthetic process |
| 43549 | 1.90E-06 | 4.31E-05 | regulation of kinase activity |
| 16125 | 2.25E-06 | 5.07E-05 | sterol metabolic process |
| 1655 | 2.30E-06 | 5.17E-05 | urogenital system development |
| 34097 | 2.35E-06 | 5.26E-05 | response to cytokine stimulus |
| 8361 | 2.40E-06 | 5.33E-05 | regulation of cell size |
| 46649 | 2.41E-06 | 5.33E-05 | lymphocyte activation |
| 51707 | 2.42E-06 | 5.33E-05 | response to other organism |
| 18193 | 2.43E-06 | 5.34E-05 | peptidyl-amino acid modification |
| 7169 | 2.70E-06 | 5.90E-05 | transmembrane receptor protein tyrosine kinase signaling pathway |
| 51347 | 3.03E-06 | 6.59E-05 | positive regulation of transferase activity |
| 42060 | 4.12E-06 | 8.92E-05 | wound healing |
| 40007 | 4.20E-06 | 9.06E-05 | growth |
| 7267 | 4.24E-06 | 9.09E-05 | cell-cell signaling |
| 61061 | 4.55E-06 | 9.72E-05 | muscle structure development |
| 10517 | 4.58E-06 | 9.73E-05 | regulation of phospholipase activity |
| 51043 | 4.93E-06 | 1.04E-04 | regulation of membrane protein ectodomain proteolysis |
| 60389 | 4.93E-06 | 1.04E-04 | pathway-restricted SMAD protein phosphorylation |
| 61024 | 5.14E-06 | 1.08E-04 | membrane organization |
| 35239 | 5.25E-06 | 1.10E-04 | tube morphogenesis |
| 32535 | 5.28E-06 | 1.10E-04 | regulation of cellular component size |
| 32844 | 5.32E-06 | 1.10E-04 | regulation of homeostatic process |
| 43086 | 5.43E-06 | 1.12E-04 | negative regulation of catalytic activity |
| 48585 | 5.50E-06 | 1.13E-04 | negative regulation of response to stimulus |
| 10975 | 5.61E-06 | 1.15E-04 | regulation of neuron projection development |
| 10562 | 6.19E-06 | 1.26E-04 | positive regulation of phosphorus metabolic process |
| 45937 | 6.19E-06 | 1.26E-04 | positive regulation of phosphate metabolic process |
| 42398 | 6.23E-06 | 1.26E-04 | cellular amino acid derivative biosynthetic process |
| 10769 | 6.27E-06 | 1.26E-04 | regulation of cell morphogenesis involved in differentiation |
| 51338 | 6.70E-06 | 1.34E-04 | regulation of transferase activity |
| 44238 | 6.72E-06 | 1.34E-04 | primary metabolic process |
| 30336 | 7.64E-06 | 1.52E-04 | negative regulation of cell migration |
| 1763 | 7.74E-06 | 1.53E-04 | morphogenesis of a branching structure |
| 9310 | 8.01E-06 | 1.58E-04 | amine catabolic process |
| 2544 | 8.85E-06 | 1.74E-04 | chronic inflammatory response |
| 7613 | 9.34E-06 | 1.83E-04 | memory |
| 16044 | 9.67E-06 | 1.88E-04 | cellular membrane organization |
| 35295 | 9.91E-06 | 1.92E-04 | tube development |
| 2696 | 1.09E-05 | 2.11E-04 | positive regulation of leukocyte activation |
| 30193 | 1.10E-05 | 2.11E-04 | regulation of blood coagulation |
| 8610 | 1.10E-05 | 2.12E-04 | lipid biosynthetic process |
| 42327 | 1.18E-05 | 2.25E-04 | positive regulation of phosphorylation |
| 45785 | 1.19E-05 | 2.27E-04 | positive regulation of cell adhesion |
| 32570 | 1.20E-05 | 2.27E-04 | response to progesterone stimulus |
| 50778 | 1.20E-05 | 2.27E-04 | positive regulation of immune response |
| 51129 | 1.27E-05 | 2.38E-04 | negative regulation of cellular component organization |
| 46942 | 1.28E-05 | 2.41E-04 | carboxylic acid transport |
| 48167 | 1.33E-05 | 2.47E-04 | regulation of synaptic plasticity |
| 51234 | 1.37E-05 | 2.55E-04 | establishment of localization |
| 22008 | 1.41E-05 | 2.61E-04 | neurogenesis |
| 31349 | 1.45E-05 | 2.67E-04 | positive regulation of defense response |
| 15849 | 1.48E-05 | 2.72E-04 | organic acid transport |
| 46395 | 1.49E-05 | 2.72E-04 | carboxylic acid catabolic process |
| 16054 | 1.49E-05 | 2.72E-04 | organic acid catabolic process |
| 2685 | 1.50E-05 | 2.73E-04 | regulation of leukocyte migration |
| 31175 | 1.53E-05 | 2.78E-04 | neuron projection development |
| 44237 | 1.60E-05 | 2.88E-04 | cellular metabolic process |
| 9125 | 1.67E-05 | 3.01E-04 | nucleoside monophosphate catabolic process |
| 42110 | 1.74E-05 | 3.11E-04 | T cell activation |
| 35467 | 1.76E-05 | 3.14E-04 | negative regulation of signaling pathway |
| 48699 | 1.79E-05 | 3.18E-04 | generation of neurons |
| 10812 | 1.81E-05 | 3.19E-04 | negative regulation of cell-substrate adhesion |
| 6810 | 1.81E-05 | 3.19E-04 | transport |
| 6695 | 1.85E-05 | 3.25E-04 | cholesterol biosynthetic process |
| 71310 | 1.93E-05 | 3.38E-04 | cellular response to organic substance |
| 18212 | 2.03E-05 | 3.54E-04 | peptidyl-tyrosine modification |
| 6865 | 2.11E-05 | 3.66E-04 | amino acid transport |
| 7160 | 2.11E-05 | 3.66E-04 | cell-matrix adhesion |
| 90092 | 2.17E-05 | 3.75E-04 | regulation of transmembrane receptor protein serine/threonine kinase signaling pathway |
| 51493 | 2.23E-05 | 3.84E-04 | regulation of cytoskeleton organization |
| 40013 | 2.25E-05 | 3.86E-04 | negative regulation of locomotion |
| 32147 | 2.35E-05 | 4.00E-04 | activation of protein kinase activity |
| 43405 | 2.35E-05 | 4.00E-04 | regulation of MAP kinase activity |
| 5975 | 2.38E-05 | 4.04E-04 | carbohydrate metabolic process |
| 9214 | 2.46E-05 | 4.17E-04 | cyclic nucleotide catabolic process |
| 9309 | 2.54E-05 | 4.26E-04 | amine biosynthetic process |
| 6633 | 2.54E-05 | 4.26E-04 | fatty acid biosynthetic process |
| 1822 | 2.58E-05 | 4.31E-04 | kidney development |
| 6916 | 2.58E-05 | 4.31E-04 | anti-apoptosis |
| 2687 | 2.68E-05 | 4.46E-04 | positive regulation of leukocyte migration |
| 51480 | 2.72E-05 | 4.50E-04 | cytosolic calcium ion homeostasis |
| 10810 | 2.82E-05 | 4.65E-04 | regulation of cell-substrate adhesion |
| 6631 | 2.85E-05 | 4.69E-04 | fatty acid metabolic process |
| 44270 | 3.07E-05 | 5.02E-04 | cellular nitrogen compound catabolic process |
| 31346 | 3.07E-05 | 5.02E-04 | positive regulation of cell projection organization |
| 51046 | 3.16E-05 | 5.12E-04 | regulation of secretion |
| 60324 | 3.16E-05 | 5.12E-04 | face development |
| 46627 | 3.16E-05 | 5.12E-04 | negative regulation of insulin receptor signaling pathway |
| 90100 | 3.27E-05 | 5.27E-04 | positive regulation of transmembrane receptor protein serine/threonine kinase signaling pathway |
| 46700 | 3.35E-05 | 5.39E-04 | heterocycle catabolic process |
| 31667 | 3.48E-05 | 5.55E-04 | response to nutrient levels |
| 23014 | 3.48E-05 | 5.55E-04 | signal transmission via phosphorylation event |
| 7243 | 3.48E-05 | 5.55E-04 | intracellular protein kinase cascade |
| 44092 | 3.51E-05 | 5.57E-04 | negative regulation of molecular function |
| 7612 | 3.57E-05 | 5.65E-04 | learning |
| 50818 | 3.59E-05 | 5.65E-04 | regulation of coagulation |
| 6749 | 4.08E-05 | 6.38E-04 | glutathione metabolic process |
| 7159 | 4.08E-05 | 6.38E-04 | leukocyte cell-cell adhesion |
| 50900 | 4.17E-05 | 6.50E-04 | leukocyte migration |
| 72001 | 4.18E-05 | 6.50E-04 | renal system development |
| 8016 | 4.24E-05 | 6.57E-04 | regulation of heart contraction |
| 9607 | 4.27E-05 | 6.60E-04 | response to biotic stimulus |
| 19216 | 4.45E-05 | 6.86E-04 | regulation of lipid metabolic process |
| 46890 | 4.50E-05 | 6.89E-04 | regulation of lipid biosynthetic process |
| 7179 | 4.50E-05 | 6.89E-04 | transforming growth factor beta receptor signaling pathway |
| 15837 | 4.68E-05 | 7.15E-04 | amine transport |
| 6955 | 4.75E-05 | 7.23E-04 | immune response |
| 51249 | 4.97E-05 | 7.55E-04 | regulation of lymphocyte activation |
| 6520 | 4.99E-05 | 7.55E-04 | cellular amino acid metabolic process |
| 48754 | 5.09E-05 | 7.68E-04 | branching morphogenesis of a tube |
| 6917 | 5.14E-05 | 7.72E-04 | induction of apoptosis |
| 48008 | 5.27E-05 | 7.90E-04 | platelet-derived growth factor receptor signaling pathway |
| 51345 | 5.54E-05 | 8.28E-04 | positive regulation of hydrolase activity |
| 12502 | 5.57E-05 | 8.30E-04 | induction of programmed cell death |
| 51251 | 5.69E-05 | 8.41E-04 | positive regulation of lymphocyte activation |
| 51044 | 5.72E-05 | 8.41E-04 | positive regulation of membrane protein ectodomain proteolysis |
| 10953 | 5.72E-05 | 8.41E-04 | regulation of protein maturation by peptide bond cleavage |
| 70613 | 5.72E-05 | 8.41E-04 | regulation of protein processing |
| 48729 | 5.78E-05 | 8.48E-04 | tissue morphogenesis |
| 18108 | 5.89E-05 | 8.54E-04 | peptidyl-tyrosine phosphorylation |
| 22414 | 5.89E-05 | 8.54E-04 | reproductive process |
| 1934 | 5.90E-05 | 8.54E-04 | positive regulation of protein amino acid phosphorylation |
| 44262 | 5.90E-05 | 8.54E-04 | cellular carbohydrate metabolic process |
| 50730 | 5.91E-05 | 8.54E-04 | regulation of peptidyl-tyrosine phosphorylation |
| 19932 | 6.05E-05 | 8.71E-04 | second-messenger-mediated signaling |
| 3 | 6.81E-05 | 9.79E-04 | reproduction |
| 51346 | 7.01E-05 | 1.00E-03 | negative regulation of hydrolase activity |
| 6979 | 7.06E-05 | 1.01E-03 | response to oxidative stress |
| 48871 | 7.16E-05 | 1.02E-03 | multicellular organismal homeostasis |
| 7178 | 7.67E-05 | 1.09E-03 | transmembrane receptor protein serine/threonine kinase signaling pathway |
| 7565 | 7.77E-05 | 1.10E-03 | female pregnancy |
| 6198 | 7.89E-05 | 1.11E-03 | cAMP catabolic process |
| 45664 | 7.99E-05 | 1.13E-03 | regulation of neuron differentiation |
| 902 | 8.16E-05 | 1.15E-03 | cell morphogenesis |
| 22602 | 8.23E-05 | 1.15E-03 | ovulation cycle process |
| 2026 | 8.43E-05 | 1.17E-03 | regulation of the force of heart contraction |
| 8219 | 8.44E-05 | 1.17E-03 | cell death |
| 42698 | 8.45E-05 | 1.17E-03 | ovulation cycle |
| 22612 | 8.45E-05 | 1.17E-03 | gland morphogenesis |
| 30195 | 8.89E-05 | 1.23E-03 | negative regulation of blood coagulation |
| 70665 | 9.45E-05 | 1.30E-03 | positive regulation of leukocyte proliferation |
| 7156 | 9.64E-05 | 1.32E-03 | homophilic cell adhesion |
| 45765 | 9.99E-05 | 1.37E-03 | regulation of angiogenesis |
| 50921 | 1.01E-04 | 1.38E-03 | positive regulation of chemotaxis |
| 23056 | 1.05E-04 | 1.42E-03 | positive regulation of signaling process |
| 33003 | 1.08E-04 | 1.47E-03 | regulation of mast cell activation |
| 16265 | 1.09E-04 | 1.47E-03 | death |
| 16042 | 1.10E-04 | 1.48E-03 | lipid catabolic process |
| 70663 | 1.14E-04 | 1.53E-03 | regulation of leukocyte proliferation |
| 6576 | 1.14E-04 | 1.53E-03 | cellular biogenic amine metabolic process |
| 10518 | 1.14E-04 | 1.53E-03 | positive regulation of phospholipase activity |
| 10740 | 1.15E-04 | 1.54E-03 | positive regulation of intracellular protein kinase cascade |
| 10543 | 1.17E-04 | 1.55E-03 | regulation of platelet activation |
| 42326 | 1.18E-04 | 1.55E-03 | negative regulation of phosphorylation |
| 34655 | 1.18E-04 | 1.55E-03 | nucleobase, nucleoside, nucleotide and nucleic acid catabolic process |
| 34656 | 1.18E-04 | 1.55E-03 | nucleobase, nucleoside and nucleotide catabolic process |
| 42445 | 1.18E-04 | 1.55E-03 | hormone metabolic process |
| 2009 | 1.24E-04 | 1.63E-03 | morphogenesis of an epithelium |
| 52547 | 1.26E-04 | 1.65E-03 | regulation of peptidase activity |
| 904 | 1.26E-04 | 1.65E-03 | cell morphogenesis involved in differentiation |
| 19229 | 1.28E-04 | 1.67E-03 | regulation of vasoconstriction |
| 8299 | 1.30E-04 | 1.68E-03 | isoprenoid biosynthetic process |
| 14910 | 1.30E-04 | 1.68E-03 | regulation of smooth muscle cell migration |
| 60284 | 1.31E-04 | 1.69E-03 | regulation of cell development |
| 10594 | 1.31E-04 | 1.69E-03 | regulation of endothelial cell migration |
| 9967 | 1.42E-04 | 1.83E-03 | positive regulation of signal transduction |
| 6518 | 1.46E-04 | 1.87E-03 | peptide metabolic process |
| 50767 | 1.51E-04 | 1.93E-03 | regulation of neurogenesis |
| 45087 | 1.56E-04 | 1.98E-03 | innate immune response |
| 71495 | 1.56E-04 | 1.98E-03 | cellular response to endogenous stimulus |
| 32870 | 1.57E-04 | 1.99E-03 | cellular response to hormone stimulus |
| 9968 | 1.64E-04 | 2.07E-03 | negative regulation of signal transduction |
| 51960 | 1.66E-04 | 2.10E-03 | regulation of nervous system development |
| 45471 | 1.68E-04 | 2.11E-03 | response to ethanol |
| 60021 | 1.68E-04 | 2.11E-03 | palate development |
| 3012 | 1.73E-04 | 2.17E-03 | muscle system process |
| 7517 | 1.74E-04 | 2.18E-03 | muscle organ development |
| 52548 | 1.78E-04 | 2.21E-03 | regulation of endopeptidase activity |
| 48732 | 1.78E-04 | 2.21E-03 | gland development |
| 45907 | 1.79E-04 | 2.21E-03 | positive regulation of vasoconstriction |
| 50819 | 1.82E-04 | 2.23E-03 | negative regulation of coagulation |
| 2821 | 1.82E-04 | 2.23E-03 | positive regulation of adaptive immune response |
| 6195 | 1.82E-04 | 2.23E-03 | purine nucleotide catabolic process |
| 7435 | 1.82E-04 | 2.23E-03 | salivary gland morphogenesis |
| 32989 | 1.87E-04 | 2.29E-03 | cellular component morphogenesis |
| 2683 | 1.87E-04 | 2.29E-03 | negative regulation of immune system process |
| 1817 | 1.89E-04 | 2.30E-03 | regulation of cytokine production |
| 19935 | 1.92E-04 | 2.33E-03 | cyclic-nucleotide-mediated signaling |
| 60401 | 1.95E-04 | 2.35E-03 | cytosolic calcium ion transport |
| 46626 | 1.95E-04 | 2.35E-03 | regulation of insulin receptor signaling pathway |
| 10720 | 1.95E-04 | 2.35E-03 | positive regulation of cell development |
| 51051 | 1.95E-04 | 2.35E-03 | negative regulation of transport |
| 9063 | 1.98E-04 | 2.38E-03 | cellular amino acid catabolic process |
| 1558 | 2.00E-04 | 2.39E-03 | regulation of cell growth |
| 48666 | 2.03E-04 | 2.42E-03 | neuron development |
| 23057 | 2.11E-04 | 2.52E-03 | negative regulation of signaling process |
| 9074 | 2.17E-04 | 2.54E-03 | aromatic amino acid family catabolic process |
| 42423 | 2.17E-04 | 2.54E-03 | catecholamine biosynthetic process |
| 51953 | 2.17E-04 | 2.54E-03 | negative regulation of amine transport |
| 2920 | 2.17E-04 | 2.54E-03 | regulation of humoral immune response |
| 45822 | 2.17E-04 | 2.54E-03 | negative regulation of heart contraction |
| 7616 | 2.17E-04 | 2.54E-03 | long-term memory |
| 48608 | 2.17E-04 | 2.55E-03 | reproductive structure development |
| 16049 | 2.20E-04 | 2.57E-03 | cell growth |
| 50678 | 2.28E-04 | 2.65E-03 | regulation of epithelial cell proliferation |
| 51604 | 2.32E-04 | 2.69E-03 | protein maturation |
| 50671 | 2.32E-04 | 2.69E-03 | positive regulation of lymphocyte proliferation |
| 9743 | 2.34E-04 | 2.71E-03 | response to carbohydrate stimulus |
| 23036 | 2.36E-04 | 2.71E-03 | initiation of signal transduction |
| 23038 | 2.36E-04 | 2.71E-03 | signal initiation by diffusible mediator |
| 23049 | 2.36E-04 | 2.71E-03 | signal initiation by protein/peptide mediator |
| 32964 | 2.40E-04 | 2.73E-03 | collagen biosynthetic process |
| 51895 | 2.40E-04 | 2.73E-03 | negative regulation of focal adhesion assembly |
| 30449 | 2.40E-04 | 2.73E-03 | regulation of complement activation |
| 71675 | 2.40E-04 | 2.73E-03 | regulation of mononuclear cell migration |
| 31399 | 2.44E-04 | 2.77E-03 | regulation of protein modification process |
| 50920 | 2.47E-04 | 2.79E-03 | regulation of chemotaxis |
| 50670 | 2.52E-04 | 2.85E-03 | regulation of lymphocyte proliferation |
| 2673 | 2.53E-04 | 2.85E-03 | regulation of acute inflammatory response |
| 9617 | 2.58E-04 | 2.91E-03 | response to bacterium |
| 1503 | 2.63E-04 | 2.95E-03 | ossification |
| 7187 | 2.63E-04 | 2.95E-03 | G-protein signaling, coupled to cyclic nucleotide second messenger |
| 10563 | 2.68E-04 | 2.98E-03 | negative regulation of phosphorus metabolic process |
| 45936 | 2.68E-04 | 2.98E-03 | negative regulation of phosphate metabolic process |
| 9166 | 2.69E-04 | 2.99E-03 | nucleotide catabolic process |
| 10324 | 2.73E-04 | 3.02E-03 | membrane invagination |
| 6897 | 2.73E-04 | 3.02E-03 | endocytosis |
| 46777 | 2.75E-04 | 3.04E-03 | protein amino acid autophosphorylation |
| 32946 | 2.78E-04 | 3.07E-03 | positive regulation of mononuclear cell proliferation |
| 19915 | 2.81E-04 | 3.09E-03 | lipid storage |
| 60491 | 2.83E-04 | 3.11E-03 | regulation of cell projection assembly |
| 32944 | 2.92E-04 | 3.19E-03 | regulation of mononuclear cell proliferation |
| 9628 | 2.98E-04 | 3.26E-03 | response to abiotic stimulus |
| 30097 | 3.06E-04 | 3.34E-03 | hemopoiesis |
| 43687 | 3.31E-04 | 3.60E-03 | post-translational protein modification |
| 46903 | 3.37E-04 | 3.65E-03 | secretion |
| 2688 | 3.44E-04 | 3.72E-03 | regulation of leukocyte chemotaxis |
| 7431 | 3.44E-04 | 3.72E-03 | salivary gland development |
| 45833 | 3.60E-04 | 3.87E-03 | negative regulation of lipid metabolic process |
| 60341 | 3.62E-04 | 3.89E-03 | regulation of cellular localization |
| 10827 | 3.66E-04 | 3.93E-03 | regulation of glucose transport |
| 10632 | 3.74E-04 | 3.98E-03 | regulation of epithelial cell migration |
| 30865 | 3.74E-04 | 3.98E-03 | cortical cytoskeleton organization |
| 30866 | 3.74E-04 | 3.98E-03 | cortical actin cytoskeleton organization |
| 2253 | 3.86E-04 | 4.11E-03 | activation of immune response |
| 60193 | 3.90E-04 | 4.13E-03 | positive regulation of lipase activity |
| 2690 | 4.02E-04 | 4.25E-03 | positive regulation of leukocyte chemotaxis |
| 60322 | 4.02E-04 | 4.25E-03 | head development |
| 60390 | 4.10E-04 | 4.31E-03 | regulation of SMAD protein nuclear translocation |
| 3254 | 4.10E-04 | 4.31E-03 | regulation of membrane depolarization |
| 2526 | 4.14E-04 | 4.34E-03 | acute inflammatory response |
| 60326 | 4.17E-04 | 4.35E-03 | cell chemotaxis |
| 48520 | 4.17E-04 | 4.35E-03 | positive regulation of behavior |
| 6464 | 4.17E-04 | 4.35E-03 | protein modification process |
| 7204 | 4.23E-04 | 4.40E-03 | elevation of cytosolic calcium ion concentration |
| 51055 | 4.27E-04 | 4.43E-03 | negative regulation of lipid biosynthetic process |
| 8217 | 4.43E-04 | 4.59E-03 | regulation of blood pressure |
| 34329 | 4.52E-04 | 4.65E-03 | cell junction assembly |
| 48565 | 4.52E-04 | 4.65E-03 | digestive tract development |
| 48771 | 4.52E-04 | 4.65E-03 | tissue remodeling |
| 1508 | 4.57E-04 | 4.70E-03 | regulation of action potential |
| 32768 | 4.62E-04 | 4.73E-03 | regulation of monooxygenase activity |
| 7044 | 4.62E-04 | 4.73E-03 | cell-substrate junction assembly |
| 16485 | 4.64E-04 | 4.74E-03 | protein processing |
| 33993 | 4.74E-04 | 4.81E-03 | response to lipid |
| 42401 | 4.74E-04 | 4.81E-03 | cellular biogenic amine biosynthetic process |
| 48168 | 4.74E-04 | 4.81E-03 | regulation of neuronal synaptic plasticity |
| 7507 | 4.82E-04 | 4.88E-03 | heart development |
| 50863 | 4.87E-04 | 4.92E-03 | regulation of T cell activation |
| 44242 | 5.08E-04 | 5.12E-03 | cellular lipid catabolic process |
| 6936 | 5.22E-04 | 5.26E-03 | muscle contraction |
| 50866 | 5.57E-04 | 5.60E-03 | negative regulation of cell activation |
| 3006 | 5.59E-04 | 5.60E-03 | reproductive developmental process |
| 8285 | 5.65E-04 | 5.65E-03 | negative regulation of cell proliferation |
| 43407 | 5.69E-04 | 5.67E-03 | negative regulation of MAP kinase activity |
| 55067 | 5.69E-04 | 5.67E-03 | monovalent inorganic cation homeostasis |
| 90257 | 5.88E-04 | 5.84E-03 | regulation of muscle system process |
| 48534 | 5.99E-04 | 5.90E-03 | hemopoietic or lymphoid organ development |
| 10039 | 6.07E-04 | 5.90E-03 | response to iron ion |
| 33004 | 6.08E-04 | 5.90E-03 | negative regulation of mast cell activation |
| 1766 | 6.08E-04 | 5.90E-03 | membrane raft polarization |
| 35405 | 6.08E-04 | 5.90E-03 | histone-threonine phosphorylation |
| 60317 | 6.08E-04 | 5.90E-03 | cardiac epithelial to mesenchymal transition |
| 19405 | 6.08E-04 | 5.90E-03 | alditol catabolic process |
| 45760 | 6.08E-04 | 5.90E-03 | positive regulation of action potential |
| 46069 | 6.08E-04 | 5.90E-03 | cGMP catabolic process |
| 31580 | 6.08E-04 | 5.90E-03 | membrane raft distribution |
| 48170 | 6.08E-04 | 5.90E-03 | positive regulation of long-term neuronal synaptic plasticity |
| 32656 | 6.08E-04 | 5.90E-03 | regulation of interleukin-13 production |
| 60348 | 6.12E-04 | 5.93E-03 | bone development |
| 32228 | 6.27E-04 | 6.06E-03 | regulation of synaptic transmission, GABAergic |
| 32846 | 6.28E-04 | 6.06E-03 | positive regulation of homeostatic process |
| 33043 | 6.36E-04 | 6.13E-03 | regulation of organelle organization |
| 51048 | 6.48E-04 | 6.22E-03 | negative regulation of secretion |
| 19221 | 6.48E-04 | 6.22E-03 | cytokine-mediated signaling pathway |
| 6469 | 6.58E-04 | 6.29E-03 | negative regulation of protein kinase activity |
| 1894 | 6.62E-04 | 6.29E-03 | tissue homeostasis |
| 10863 | 6.62E-04 | 6.29E-03 | positive regulation of phospholipase C activity |
| 7202 | 6.62E-04 | 6.29E-03 | activation of phospholipase C activity |
| 10544 | 6.71E-04 | 6.33E-03 | negative regulation of platelet activation |
| 10955 | 6.71E-04 | 6.33E-03 | negative regulation of protein maturation by peptide bond cleavage |
| 71322 | 6.71E-04 | 6.33E-03 | cellular response to carbohydrate stimulus |
| 32695 | 6.71E-04 | 6.33E-03 | negative regulation of interleukin-12 production |
| 6694 | 6.78E-04 | 6.38E-03 | steroid biosynthetic process |
| 48468 | 6.81E-04 | 6.41E-03 | cell development |
| 42391 | 6.83E-04 | 6.41E-03 | regulation of membrane potential |
| 32270 | 7.44E-04 | 6.97E-03 | positive regulation of cellular protein metabolic process |
| 2824 | 7.61E-04 | 7.08E-03 | positive regulation of adaptive immune response based on somatic recombination of immune receptors built from immunoglobulin superfamily domains |
| 44271 | 7.61E-04 | 7.08E-03 | cellular nitrogen compound biosynthetic process |
| 1960 | 7.62E-04 | 7.08E-03 | negative regulation of cytokine-mediated signaling pathway |
| 10634 | 7.62E-04 | 7.08E-03 | positive regulation of epithelial cell migration |
| 46483 | 7.99E-04 | 7.41E-03 | heterocycle metabolic process |
| 33273 | 8.47E-04 | 7.84E-03 | response to vitamin |
| 30182 | 8.63E-04 | 7.97E-03 | neuron differentiation |
| 19218 | 8.71E-04 | 8.04E-03 | regulation of steroid metabolic process |
| 34330 | 8.84E-04 | 8.14E-03 | cell junction organization |
| 10876 | 8.89E-04 | 8.15E-03 | lipid localization |
| 48511 | 8.89E-04 | 8.15E-03 | rhythmic process |
| 33158 | 8.94E-04 | 8.17E-03 | regulation of protein import into nucleus, translocation |
| 7157 | 8.94E-04 | 8.17E-03 | heterophilic cell-cell adhesion |
| 2819 | 9.23E-04 | 8.41E-03 | regulation of adaptive immune response |
| 3018 | 9.23E-04 | 8.41E-03 | vascular process in circulatory system |
| 51592 | 9.36E-04 | 8.48E-03 | response to calcium ion |
| 33160 | 9.39E-04 | 8.48E-03 | positive regulation of protein import into nucleus, translocation |
| 42402 | 9.39E-04 | 8.48E-03 | cellular biogenic amine catabolic process |
| 15804 | 9.39E-04 | 8.48E-03 | neutral amino acid transport |
| 51047 | 9.41E-04 | 8.48E-03 | positive regulation of secretion |
| 30031 | 9.64E-04 | 8.68E-03 | cell projection assembly |
| 51952 | 9.66E-04 | 8.68E-03 | regulation of amine transport |
| 9056 | 9.93E-04 | 8.91E-03 | catabolic process |
| 2520 | 9.95E-04 | 8.91E-03 | immune system development |
| 50806 | 1.02E-03 | 9.09E-03 | positive regulation of synaptic transmission |
| 7566 | 1.02E-03 | 9.09E-03 | embryo implantation |
| 33555 | 1.03E-03 | 9.14E-03 | multicellular organismal response to stress |
| 43434 | 1.07E-03 | 9.47E-03 | response to peptide hormone stimulus |
| 10627 | 1.07E-03 | 9.48E-03 | regulation of intracellular protein kinase cascade |
| 51605 | 1.11E-03 | 9.81E-03 | protein maturation by peptide bond cleavage |
| 90276 | 1.11E-03 | 9.81E-03 | regulation of peptide hormone secretion |
| 51246 | 1.12E-03 | 9.86E-03 | regulation of protein metabolic process |
| 45137 | 1.12E-03 | 9.86E-03 | development of primary sexual characteristics |
| 51247 | 1.18E-03 | 1.04E-02 | positive regulation of protein metabolic process |
| 6732 | 1.19E-03 | 1.04E-02 | coenzyme metabolic process |
| 6937 | 1.19E-03 | 1.04E-02 | regulation of muscle contraction |
| 44236 | 1.20E-03 | 1.05E-02 | multicellular organismal metabolic process |
| 33673 | 1.22E-03 | 1.06E-02 | negative regulation of kinase activity |
| 42219 | 1.24E-03 | 1.08E-02 | cellular amino acid derivative catabolic process |
| 2053 | 1.24E-03 | 1.08E-02 | positive regulation of mesenchymal cell proliferation |
| 60393 | 1.24E-03 | 1.08E-02 | regulation of pathway-restricted SMAD protein phosphorylation |
| 46660 | 1.27E-03 | 1.10E-02 | female sex differentiation |
| 33628 | 1.30E-03 | 1.11E-02 | regulation of cell adhesion mediated by integrin |
| 9070 | 1.30E-03 | 1.11E-02 | serine family amino acid biosynthetic process |
| 1953 | 1.30E-03 | 1.11E-02 | negative regulation of cell-matrix adhesion |
| 2040 | 1.30E-03 | 1.11E-02 | sprouting angiogenesis |
| 51893 | 1.30E-03 | 1.11E-02 | regulation of focal adhesion assembly |
| 60325 | 1.30E-03 | 1.11E-02 | face morphogenesis |
| 90109 | 1.30E-03 | 1.11E-02 | regulation of cell-substrate junction assembly |
| 44259 | 1.31E-03 | 1.11E-02 | multicellular organismal macromolecule metabolic process |
| 45768 | 1.31E-03 | 1.11E-02 | positive regulation of anti-apoptosis |
| 2252 | 1.32E-03 | 1.12E-02 | immune effector process |
| 60537 | 1.32E-03 | 1.12E-02 | muscle tissue development |
| 50433 | 1.34E-03 | 1.14E-02 | regulation of catecholamine secretion |
| 45778 | 1.34E-03 | 1.14E-02 | positive regulation of ossification |
| 60627 | 1.34E-03 | 1.14E-02 | regulation of vesicle-mediated transport |
| 32970 | 1.36E-03 | 1.14E-02 | regulation of actin filament-based process |
| 9266 | 1.36E-03 | 1.14E-02 | response to temperature stimulus |
| 32268 | 1.36E-03 | 1.14E-02 | regulation of cellular protein metabolic process |
| 34199 | 1.40E-03 | 1.17E-02 | activation of protein kinase A activity |
| 46058 | 1.40E-03 | 1.17E-02 | cAMP metabolic process |
| 46676 | 1.40E-03 | 1.17E-02 | negative regulation of insulin secretion |
| 16572 | 1.46E-03 | 1.20E-02 | histone phosphorylation |
| 33137 | 1.46E-03 | 1.20E-02 | negative regulation of peptidyl-serine phosphorylation |
| 33604 | 1.46E-03 | 1.20E-02 | negative regulation of catecholamine secretion |
| 10829 | 1.46E-03 | 1.20E-02 | negative regulation of glucose transport |
| 60192 | 1.46E-03 | 1.20E-02 | negative regulation of lipase activity |
| 6072 | 1.46E-03 | 1.20E-02 | glycerol-3-phosphate metabolic process |
| 31579 | 1.46E-03 | 1.20E-02 | membrane raft organization |
| 48013 | 1.46E-03 | 1.20E-02 | ephrin receptor signaling pathway |
| 7185 | 1.46E-03 | 1.20E-02 | transmembrane receptor protein tyrosine phosphatase signaling pathway |
| 45767 | 1.48E-03 | 1.22E-02 | regulation of anti-apoptosis |
| 2791 | 1.54E-03 | 1.26E-02 | regulation of peptide secretion |
| 90087 | 1.54E-03 | 1.26E-02 | regulation of peptide transport |
| 7548 | 1.54E-03 | 1.26E-02 | sex differentiation |
| 50796 | 1.57E-03 | 1.29E-02 | regulation of insulin secretion |
| 50680 | 1.64E-03 | 1.34E-02 | negative regulation of epithelial cell proliferation |
| 43588 | 1.64E-03 | 1.34E-02 | skin development |
| 16192 | 1.65E-03 | 1.34E-02 | vesicle-mediated transport |
| 34367 | 1.69E-03 | 1.36E-02 | macromolecular complex remodeling |
| 34368 | 1.69E-03 | 1.36E-02 | protein-lipid complex remodeling |
| 34369 | 1.69E-03 | 1.36E-02 | plasma lipoprotein particle remodeling |
| 51385 | 1.69E-03 | 1.36E-02 | response to mineralocorticoid stimulus |
| 43535 | 1.69E-03 | 1.36E-02 | regulation of blood vessel endothelial cell migration |
| 30004 | 1.69E-03 | 1.36E-02 | cellular monovalent inorganic cation homeostasis |
| 50795 | 1.70E-03 | 1.36E-02 | regulation of behavior |
| 43281 | 1.70E-03 | 1.37E-02 | regulation of caspase activity |
| 61138 | 1.72E-03 | 1.38E-02 | morphogenesis of a branching epithelium |
| 50777 | 1.74E-03 | 1.38E-02 | negative regulation of immune response |
| 10466 | 1.74E-03 | 1.38E-02 | negative regulation of peptidase activity |
| 60688 | 1.74E-03 | 1.38E-02 | regulation of morphogenesis of a branching structure |
| 22407 | 1.74E-03 | 1.38E-02 | regulation of cell-cell adhesion |
| 8283 | 1.75E-03 | 1.39E-02 | cell proliferation |
| 50870 | 1.81E-03 | 1.43E-02 | positive regulation of T cell activation |
| 10243 | 1.81E-03 | 1.43E-02 | response to organic nitrogen |
| 46545 | 1.81E-03 | 1.43E-02 | development of primary female sexual characteristics |
| 50769 | 1.96E-03 | 1.54E-02 | positive regulation of neurogenesis |
| 12501 | 1.96E-03 | 1.54E-02 | programmed cell death |
| 55086 | 1.99E-03 | 1.56E-02 | nucleobase, nucleoside and nucleotide metabolic process |
| 90278 | 2.00E-03 | 1.56E-02 | negative regulation of peptide hormone secretion |
| 33198 | 2.00E-03 | 1.56E-02 | response to ATP |
| 10862 | 2.00E-03 | 1.56E-02 | positive regulation of pathway-restricted SMAD protein phosphorylation |
| 2675 | 2.00E-03 | 1.56E-02 | positive regulation of acute inflammatory response |
| 32233 | 2.00E-03 | 1.56E-02 | positive regulation of actin filament bundle assembly |
| 45638 | 2.05E-03 | 1.60E-02 | negative regulation of myeloid cell differentiation |
| 9893 | 2.06E-03 | 1.60E-02 | positive regulation of metabolic process |
| 6725 | 2.07E-03 | 1.60E-02 | cellular aromatic compound metabolic process |
| 31103 | 2.07E-03 | 1.60E-02 | axon regeneration |
| 6929 | 2.07E-03 | 1.60E-02 | substrate-bound cell migration |
| 51186 | 2.07E-03 | 1.60E-02 | cofactor metabolic process |
| 50770 | 2.09E-03 | 1.61E-02 | regulation of axonogenesis |
| 55123 | 2.09E-03 | 1.61E-02 | digestive system development |
| 32956 | 2.17E-03 | 1.67E-02 | regulation of actin cytoskeleton organization |
| 45619 | 2.19E-03 | 1.68E-02 | regulation of lymphocyte differentiation |
| 30595 | 2.20E-03 | 1.69E-02 | leukocyte chemotaxis |
| 51971 | 2.23E-03 | 1.70E-02 | positive regulation of transmission of nerve impulse |
| 8585 | 2.25E-03 | 1.71E-02 | female gonad development |
| 50673 | 2.26E-03 | 1.71E-02 | epithelial cell proliferation |
| 51665 | 2.28E-03 | 1.71E-02 | membrane raft localization |
| 10759 | 2.28E-03 | 1.71E-02 | positive regulation of macrophage chemotaxis |
| 2701 | 2.28E-03 | 1.71E-02 | negative regulation of production of molecular mediator of immune response |
| 2719 | 2.28E-03 | 1.71E-02 | negative regulation of cytokine production involved in immune response |
| 44268 | 2.28E-03 | 1.71E-02 | multicellular organismal protein metabolic process |
| 46541 | 2.28E-03 | 1.71E-02 | saliva secretion |
| 14012 | 2.28E-03 | 1.71E-02 | axon regeneration in the peripheral nervous system |
| 14805 | 2.28E-03 | 1.71E-02 | smooth muscle adaptation |
| 6883 | 2.28E-03 | 1.71E-02 | cellular sodium ion homeostasis |
| 32455 | 2.28E-03 | 1.71E-02 | nerve growth factor processing |
| 43412 | 2.30E-03 | 1.72E-02 | macromolecule modification |
| 51348 | 2.38E-03 | 1.78E-02 | negative regulation of transferase activity |
| 60485 | 2.42E-03 | 1.81E-02 | mesenchyme development |
| 35272 | 2.53E-03 | 1.89E-02 | exocrine system development |
| 30100 | 2.58E-03 | 1.93E-02 | regulation of endocytosis |
| 5996 | 2.60E-03 | 1.93E-02 | monosaccharide metabolic process |
| 32940 | 2.60E-03 | 1.93E-02 | secretion by cell |
| 32886 | 2.65E-03 | 1.97E-02 | regulation of microtubule-based process |
| 2822 | 2.65E-03 | 1.97E-02 | regulation of adaptive immune response based on somatic recombination of immune receptors built from immunoglobulin superfamily domains |
| 90316 | 2.66E-03 | 1.97E-02 | positive regulation of intracellular protein transport |
| 33627 | 2.72E-03 | 1.99E-02 | cell adhesion mediated by integrin |
| 51489 | 2.72E-03 | 1.99E-02 | regulation of filopodium assembly |
| 51491 | 2.72E-03 | 1.99E-02 | positive regulation of filopodium assembly |
| 10872 | 2.72E-03 | 1.99E-02 | regulation of cholesterol esterification |
| 51900 | 2.72E-03 | 1.99E-02 | regulation of mitochondrial depolarization |
| 60391 | 2.72E-03 | 1.99E-02 | positive regulation of SMAD protein nuclear translocation |
| 45779 | 2.72E-03 | 1.99E-02 | negative regulation of bone resorption |
| 46851 | 2.72E-03 | 1.99E-02 | negative regulation of bone remodeling |
| 51259 | 2.78E-03 | 2.03E-02 | protein oligomerization |
| 51412 | 2.79E-03 | 2.03E-02 | response to corticosterone stimulus |
| 2792 | 2.79E-03 | 2.03E-02 | negative regulation of peptide secretion |
| 30593 | 2.79E-03 | 2.03E-02 | neutrophil chemotaxis |
| 72006 | 2.79E-03 | 2.03E-02 | nephron development |
| 19637 | 2.80E-03 | 2.03E-02 | organophosphate metabolic process |
| 9636 | 2.80E-03 | 2.03E-02 | response to toxin |
| 18105 | 2.81E-03 | 2.03E-02 | peptidyl-serine phosphorylation |
| 10464 | 2.96E-03 | 2.13E-02 | regulation of mesenchymal cell proliferation |
| 32231 | 2.96E-03 | 2.13E-02 | regulation of actin filament bundle assembly |
| 8360 | 2.99E-03 | 2.15E-02 | regulation of cell shape |
| 6720 | 2.99E-03 | 2.15E-02 | isoprenoid metabolic process |
| 8652 | 3.13E-03 | 2.24E-02 | cellular amino acid biosynthetic process |
| 9755 | 3.13E-03 | 2.24E-02 | hormone-mediated signaling pathway |
| 1818 | 3.18E-03 | 2.27E-02 | negative regulation of cytokine production |
| 51341 | 3.18E-03 | 2.27E-02 | regulation of oxidoreductase activity |
| 19751 | 3.18E-03 | 2.27E-02 | polyol metabolic process |
| 34754 | 3.22E-03 | 2.29E-02 | cellular hormone metabolic process |
| 2697 | 3.34E-03 | 2.38E-02 | regulation of immune effector process |
| 7186 | 3.42E-03 | 2.43E-02 | G-protein coupled receptor protein signaling pathway |
| 42246 | 3.51E-03 | 2.48E-02 | tissue regeneration |
| 50885 | 3.51E-03 | 2.48E-02 | neuromuscular process controlling balance |
| 46324 | 3.51E-03 | 2.48E-02 | regulation of glucose import |
| 46888 | 3.51E-03 | 2.48E-02 | negative regulation of hormone secretion |
| 31646 | 3.51E-03 | 2.48E-02 | positive regulation of neurological system process |
| 31401 | 3.65E-03 | 2.57E-02 | positive regulation of protein modification process |
| 43406 | 3.71E-03 | 2.61E-02 | positive regulation of MAP kinase activity |
| 45786 | 3.73E-03 | 2.62E-02 | negative regulation of cell cycle |
| 45807 | 3.76E-03 | 2.63E-02 | positive regulation of endocytosis |
| 9072 | 3.79E-03 | 2.64E-02 | aromatic amino acid family metabolic process |
| 1569 | 3.79E-03 | 2.64E-02 | patterning of blood vessels |
| 43043 | 3.79E-03 | 2.64E-02 | peptide biosynthetic process |
| 2698 | 3.79E-03 | 2.64E-02 | negative regulation of immune effector process |
| 60402 | 3.79E-03 | 2.64E-02 | calcium ion transport into cytosol |
| 10595 | 3.81E-03 | 2.64E-02 | positive regulation of endothelial cell migration |
| 16339 | 3.81E-03 | 2.64E-02 | calcium-dependent cell-cell adhesion |
| 43523 | 3.86E-03 | 2.67E-02 | regulation of neuron apoptosis |
| 2703 | 4.01E-03 | 2.77E-02 | regulation of leukocyte mediated immunity |
| 31325 | 4.22E-03 | 2.92E-02 | positive regulation of cellular metabolic process |
| 43524 | 4.28E-03 | 2.96E-02 | negative regulation of neuron apoptosis |
| 6820 | 4.31E-03 | 2.97E-02 | anion transport |
| 9306 | 4.34E-03 | 2.98E-02 | protein secretion |
| 10811 | 4.34E-03 | 2.98E-02 | positive regulation of cell-substrate adhesion |
| 16358 | 4.34E-03 | 2.98E-02 | dendrite development |
| 51495 | 4.48E-03 | 3.06E-02 | positive regulation of cytoskeleton organization |
| 6672 | 4.48E-03 | 3.06E-02 | ceramide metabolic process |
| 32388 | 4.48E-03 | 3.06E-02 | positive regulation of intracellular transport |
| 9117 | 4.52E-03 | 3.07E-02 | nucleotide metabolic process |
| 6753 | 4.52E-03 | 3.07E-02 | nucleoside phosphate metabolic process |
| 31345 | 4.53E-03 | 3.07E-02 | negative regulation of cell projection organization |
| 51602 | 4.54E-03 | 3.07E-02 | response to electrical stimulus |
| 10717 | 4.54E-03 | 3.07E-02 | regulation of epithelial to mesenchymal transition |
| 30511 | 4.54E-03 | 3.07E-02 | positive regulation of transforming growth factor beta receptor signaling pathway |
| 22409 | 4.54E-03 | 3.07E-02 | positive regulation of cell-cell adhesion |
| 31102 | 4.54E-03 | 3.07E-02 | neuron projection regeneration |
| 8354 | 4.58E-03 | 3.07E-02 | germ cell migration |
| 34104 | 4.58E-03 | 3.07E-02 | negative regulation of tissue remodeling |
| 42447 | 4.58E-03 | 3.07E-02 | hormone catabolic process |
| 34374 | 4.58E-03 | 3.07E-02 | low-density lipoprotein particle remodeling |
| 10288 | 4.58E-03 | 3.07E-02 | response to lead ion |
| 43277 | 4.58E-03 | 3.07E-02 | apoptotic cell clearance |
| 6915 | 4.59E-03 | 3.07E-02 | apoptosis |
| 8643 | 4.61E-03 | 3.08E-02 | carbohydrate transport |
| 48705 | 4.80E-03 | 3.20E-02 | skeletal system morphogenesis |
| 18149 | 4.84E-03 | 3.21E-02 | peptide cross-linking |
| 43487 | 4.84E-03 | 3.21E-02 | regulation of RNA stability |
| 19400 | 4.84E-03 | 3.21E-02 | alditol metabolic process |
| 6040 | 4.84E-03 | 3.21E-02 | amino sugar metabolic process |
| 31110 | 4.84E-03 | 3.21E-02 | regulation of microtubule polymerization or depolymerization |
| 51222 | 4.88E-03 | 3.23E-02 | positive regulation of protein transport |
| 7188 | 4.88E-03 | 3.23E-02 | G-protein signaling, coupled to cAMP nucleotide second messenger |
| 10817 | 4.95E-03 | 3.27E-02 | regulation of hormone levels |
| 50905 | 4.98E-03 | 3.28E-02 | neuromuscular process |
| 2521 | 5.00E-03 | 3.29E-02 | leukocyte differentiation |
| 48812 | 5.01E-03 | 3.29E-02 | neuron projection morphogenesis |
| 18107 | 5.03E-03 | 3.30E-02 | peptidyl-threonine phosphorylation |
| 51492 | 5.03E-03 | 3.30E-02 | regulation of stress fiber assembly |
| 48169 | 5.03E-03 | 3.30E-02 | regulation of long-term neuronal synaptic plasticity |
| 6163 | 5.04E-03 | 3.30E-02 | purine nucleotide metabolic process |
| 9749 | 5.29E-03 | 3.39E-02 | response to glucose stimulus |
| 51494 | 5.29E-03 | 3.39E-02 | negative regulation of cytoskeleton organization |
| 1952 | 5.32E-03 | 3.39E-02 | regulation of cell-matrix adhesion |
| 30500 | 5.32E-03 | 3.39E-02 | regulation of bone mineralization |
| 30856 | 5.32E-03 | 3.39E-02 | regulation of epithelial cell differentiation |
| 15914 | 5.32E-03 | 3.39E-02 | phospholipid transport |
| 32811 | 5.34E-03 | 3.39E-02 | negative regulation of epinephrine secretion |
| 90189 | 5.34E-03 | 3.39E-02 | regulation of branching involved in ureteric bud morphogenesis |
| 90190 | 5.34E-03 | 3.39E-02 | positive regulation of branching involved in ureteric bud morphogenesis |
| 34382 | 5.34E-03 | 3.39E-02 | chylomicron remnant clearance |
| 51005 | 5.34E-03 | 3.39E-02 | negative regulation of lipoprotein lipase activity |
| 10700 | 5.34E-03 | 3.39E-02 | negative regulation of norepinephrine secretion |
| 10758 | 5.34E-03 | 3.39E-02 | regulation of macrophage chemotaxis |
| 60664 | 5.34E-03 | 3.39E-02 | epithelial cell proliferation involved in salivary gland morphogenesis |
| 45059 | 5.34E-03 | 3.39E-02 | positive thymic T cell selection |
| 70120 | 5.34E-03 | 3.39E-02 | ciliary neurotrophic factor-mediated signaling pathway |
| 45920 | 5.34E-03 | 3.39E-02 | negative regulation of exocytosis |
| 45989 | 5.34E-03 | 3.39E-02 | positive regulation of striated muscle contraction |
| 46174 | 5.34E-03 | 3.39E-02 | polyol catabolic process |
| 71398 | 5.34E-03 | 3.39E-02 | cellular response to fatty acid |
| 22614 | 5.34E-03 | 3.39E-02 | membrane to membrane docking |
| 6537 | 5.34E-03 | 3.39E-02 | glutamate biosynthetic process |
| 18209 | 5.41E-03 | 3.41E-02 | peptidyl-serine modification |
| 1933 | 5.41E-03 | 3.41E-02 | negative regulation of protein amino acid phosphorylation |
| 51781 | 5.41E-03 | 3.41E-02 | positive regulation of cell division |
| 6956 | 5.41E-03 | 3.41E-02 | complement activation |
| 7422 | 5.41E-03 | 3.41E-02 | peripheral nervous system development |
| 302 | 5.46E-03 | 3.44E-02 | response to reactive oxygen species |
| 30278 | 5.46E-03 | 3.44E-02 | regulation of ossification |
| 1819 | 5.75E-03 | 3.61E-02 | positive regulation of cytokine production |
| 45793 | 5.76E-03 | 3.61E-02 | positive regulation of cell size |
| 30098 | 5.95E-03 | 3.72E-02 | lymphocyte differentiation |
| 48872 | 5.95E-03 | 3.72E-02 | homeostasis of number of cells |
| 48678 | 6.06E-03 | 3.79E-02 | response to axon injury |
| 3007 | 6.10E-03 | 3.80E-02 | heart morphogenesis |
| 30879 | 6.10E-03 | 3.80E-02 | mammary gland development |
| 19226 | 6.10E-03 | 3.80E-02 | transmission of nerve impulse |
| 22607 | 6.14E-03 | 3.82E-02 | cellular component assembly |
| 45580 | 6.16E-03 | 3.82E-02 | regulation of T cell differentiation |
| 2460 | 6.17E-03 | 3.83E-02 | adaptive immune response based on somatic recombination of immune receptors built from immunoglobulin superfamily domains |
| 32770 | 6.34E-03 | 3.91E-02 | positive regulation of monooxygenase activity |
| 10574 | 6.34E-03 | 3.91E-02 | regulation of vascular endothelial growth factor production |
| 60323 | 6.34E-03 | 3.91E-02 | head morphogenesis |
| 48041 | 6.34E-03 | 3.91E-02 | focal adhesion assembly |
| 51260 | 6.34E-03 | 3.91E-02 | protein homooligomerization |
| 2541 | 6.42E-03 | 3.95E-02 | activation of plasma proteins involved in acute inflammatory response |
| 9712 | 6.45E-03 | 3.95E-02 | catechol metabolic process |
| 34311 | 6.45E-03 | 3.95E-02 | diol metabolic process |
| 19233 | 6.45E-03 | 3.95E-02 | sensory perception of pain |
| 6584 | 6.45E-03 | 3.95E-02 | catecholamine metabolic process |
| 50879 | 6.55E-03 | 3.97E-02 | multicellular organismal movement |
| 50881 | 6.55E-03 | 3.97E-02 | musculoskeletal movement |
| 1837 | 6.55E-03 | 3.97E-02 | epithelial to mesenchymal transition |
| 1959 | 6.55E-03 | 3.97E-02 | regulation of cytokine-mediated signaling pathway |
| 43154 | 6.55E-03 | 3.97E-02 | negative regulation of caspase activity |
| 19439 | 6.55E-03 | 3.97E-02 | aromatic compound catabolic process |
| 30501 | 6.55E-03 | 3.97E-02 | positive regulation of bone mineralization |
| 31532 | 6.55E-03 | 3.97E-02 | actin cytoskeleton reorganization |
| 32655 | 6.55E-03 | 3.97E-02 | regulation of interleukin-12 production |
| 32102 | 6.63E-03 | 4.01E-02 | negative regulation of response to external stimulus |
| 48762 | 6.63E-03 | 4.01E-02 | mesenchymal cell differentiation |
| 6811 | 6.82E-03 | 4.07E-02 | ion transport |
| 42476 | 6.88E-03 | 4.07E-02 | odontogenesis |
| 2449 | 6.88E-03 | 4.07E-02 | lymphocyte mediated immunity |
| 2250 | 6.96E-03 | 4.07E-02 | adaptive immune response |
| 8210 | 7.12E-03 | 4.07E-02 | estrogen metabolic process |
| 32835 | 7.12E-03 | 4.07E-02 | glomerulus development |
| 60343 | 7.12E-03 | 4.07E-02 | trabecula formation |
| 60693 | 7.12E-03 | 4.07E-02 | regulation of branching involved in salivary gland morphogenesis |
| 31113 | 7.12E-03 | 4.07E-02 | regulation of microtubule polymerization |
| 32148 | 7.12E-03 | 4.07E-02 | activation of protein kinase B activity |
| 48745 | 7.12E-03 | 4.07E-02 | smooth muscle tissue development |
| 9100 | 7.15E-03 | 4.07E-02 | glycoprotein metabolic process |
| 34101 | 7.16E-03 | 4.07E-02 | erythrocyte homeostasis |
| 51302 | 7.16E-03 | 4.07E-02 | regulation of cell division |
| 8215 | 7.18E-03 | 4.07E-02 | spermine metabolic process |
| 33239 | 7.18E-03 | 4.07E-02 | negative regulation of cellular amine metabolic process |
| 50653 | 7.18E-03 | 4.07E-02 | chondroitin sulfate proteoglycan biosynthetic process, polysaccharide chain biosynthetic process |
| 50779 | 7.18E-03 | 4.07E-02 | RNA destabilization |
| 50882 | 7.18E-03 | 4.07E-02 | voluntary musculoskeletal movement |
| 10040 | 7.18E-03 | 4.07E-02 | response to iron(II) ion |
| 1969 | 7.18E-03 | 4.07E-02 | regulation of activation of membrane attack complex |
| 1970 | 7.18E-03 | 4.07E-02 | positive regulation of activation of membrane attack complex |
| 1999 | 7.18E-03 | 4.07E-02 | renal response to blood flow involved in circulatory renin-angiotensin regulation of systemic arterial blood pressure |
| 2019 | 7.18E-03 | 4.07E-02 | regulation of renal output by angiotensin |
| 2086 | 7.18E-03 | 4.07E-02 | diaphragm contraction |
| 2291 | 7.18E-03 | 4.07E-02 | T cell activation via T cell receptor contact with antigen bound to MHC molecule on antigen presenting cell |
| 10511 | 7.18E-03 | 4.07E-02 | regulation of phosphatidylinositol biosynthetic process |
| 10512 | 7.18E-03 | 4.07E-02 | negative regulation of phosphatidylinositol biosynthetic process |
| 2432 | 7.18E-03 | 4.07E-02 | granuloma formation |
| 35408 | 7.18E-03 | 4.07E-02 | histone H3-T6 phosphorylation |
| 2691 | 7.18E-03 | 4.07E-02 | regulation of cellular extravasation |
| 60083 | 7.18E-03 | 4.07E-02 | smooth muscle contraction involved in micturition |
| 10996 | 7.18E-03 | 4.07E-02 | response to auditory stimulus |
| 60278 | 7.18E-03 | 4.07E-02 | regulation of ovulation |
| 60279 | 7.18E-03 | 4.07E-02 | positive regulation of ovulation |
| 19344 | 7.18E-03 | 4.07E-02 | cysteine biosynthetic process |
| 60346 | 7.18E-03 | 4.07E-02 | bone trabecula formation |
| 3011 | 7.18E-03 | 4.07E-02 | involuntary skeletal muscle contraction |
| 19459 | 7.18E-03 | 4.07E-02 | glutamate deamidation |
| 60452 | 7.18E-03 | 4.07E-02 | positive regulation of cardiac muscle contraction |
| 19563 | 7.18E-03 | 4.07E-02 | glycerol catabolic process |
| 45917 | 7.18E-03 | 4.07E-02 | positive regulation of complement activation |
| 46168 | 7.18E-03 | 4.07E-02 | glycerol-3-phosphate catabolic process |
| 70813 | 7.18E-03 | 4.07E-02 | hydrogen sulfide metabolic process |
| 70814 | 7.18E-03 | 4.07E-02 | hydrogen sulfide biosynthetic process |
| 14028 | 7.18E-03 | 4.07E-02 | notochord formation |
| 71391 | 7.18E-03 | 4.07E-02 | cellular response to estrogen stimulus |
| 71505 | 7.18E-03 | 4.07E-02 | response to mycophenolic acid |
| 71504 | 7.18E-03 | 4.07E-02 | cellular response to heparin |
| 71506 | 7.18E-03 | 4.07E-02 | cellular response to mycophenolic acid |
| 6145 | 7.18E-03 | 4.07E-02 | purine base catabolic process |
| 6597 | 7.18E-03 | 4.07E-02 | spermine biosynthetic process |
| 32459 | 7.18E-03 | 4.07E-02 | regulation of protein oligomerization |
| 32764 | 7.18E-03 | 4.07E-02 | negative regulation of mast cell cytokine production |
| 70838 | 7.22E-03 | 4.08E-02 | divalent metal ion transport |
| 60429 | 7.37E-03 | 4.17E-02 | epithelium development |
| 186 | 7.50E-03 | 4.21E-02 | activation of MAPKK activity |
| 9409 | 7.50E-03 | 4.21E-02 | response to cold |
| 46677 | 7.50E-03 | 4.21E-02 | response to antibiotic |
| 6939 | 7.50E-03 | 4.21E-02 | smooth muscle contraction |
| 15749 | 7.50E-03 | 4.21E-02 | monosaccharide transport |
| 48858 | 7.54E-03 | 4.23E-02 | cell projection morphogenesis |
| 42102 | 7.57E-03 | 4.24E-02 | positive regulation of T cell proliferation |
| 42552 | 7.57E-03 | 4.24E-02 | myelination |
| 31348 | 7.57E-03 | 4.24E-02 | negative regulation of defense response |
| 6892 | 7.60E-03 | 4.24E-02 | post-Golgi vesicle-mediated transport |
| 18958 | 7.75E-03 | 4.32E-02 | phenol metabolic process |
| 7259 | 7.75E-03 | 4.32E-02 | JAK-STAT cascade |
| 44248 | 8.09E-03 | 4.50E-02 | cellular catabolic process |
| 30307 | 8.29E-03 | 4.61E-02 | positive regulation of cell growth |
| 1974 | 8.37E-03 | 4.64E-02 | blood vessel remodeling |
| 10921 | 8.37E-03 | 4.64E-02 | regulation of phosphatase activity |
| 6816 | 8.37E-03 | 4.64E-02 | calcium ion transport |
| 43278 | 8.59E-03 | 4.72E-02 | response to morphine |
| 43462 | 8.59E-03 | 4.72E-02 | regulation of ATPase activity |
| 45124 | 8.59E-03 | 4.72E-02 | regulation of bone resorption |
| 14072 | 8.59E-03 | 4.72E-02 | response to isoquinoline alkaloid |
| 46850 | 8.59E-03 | 4.72E-02 | regulation of bone remodeling |
| 7009 | 8.59E-03 | 4.72E-02 | plasma membrane organization |
| 48844 | 8.59E-03 | 4.72E-02 | artery morphogenesis |
| 19228 | 8.68E-03 | 4.77E-02 | regulation of action potential in neuron |
| 42176 | 8.79E-03 | 4.82E-02 | regulation of protein catabolic process |
| 42129 | 8.82E-03 | 4.82E-02 | regulation of T cell proliferation |
| 34284 | 8.82E-03 | 4.82E-02 | response to monosaccharide stimulus |
| 9746 | 8.82E-03 | 4.82E-02 | response to hexose stimulus |
| 45454 | 8.82E-03 | 4.82E-02 | cell redox homeostasis |
| 45766 | 8.88E-03 | 4.84E-02 | positive regulation of angiogenesis |
| 30218 | 8.88E-03 | 4.84E-02 | erythrocyte differentiation |
| 9615 | 9.16E-03 | 4.98E-02 | response to virus |
| 48661 | 9.18E-03 | 4.98E-02 | positive regulation of smooth muscle cell proliferation |
| 48806 | 9.18E-03 | 4.98E-02 | genitalia development |
| 31100 | 9.24E-03 | 5.01E-02 | organ regeneration |
| 7050 | 9.24E-03 | 5.01E-02 | cell cycle arrest |
| 10638 | 9.25E-03 | 5.01E-02 | positive regulation of organelle organization |
| 9058 | 9.33E-03 | 5.05E-02 | biosynthetic process |
| 10604 | 9.44E-03 | 5.10E-02 | positive regulation of macromolecule metabolic process |
| 17015 | 9.55E-03 | 5.15E-02 | regulation of transforming growth factor beta receptor signaling pathway |
| 43410 | 9.58E-03 | 5.16E-02 | positive regulation of MAPKKK cascade |
| 6836 | 9.58E-03 | 5.16E-02 | neurotransmitter transport |
| 8277 | 9.87E-03 | 5.26E-02 | regulation of G-protein coupled receptor protein signaling pathway |
| 9123 | 9.87E-03 | 5.26E-02 | nucleoside monophosphate metabolic process |
| 19217 | 9.87E-03 | 5.26E-02 | regulation of fatty acid metabolic process |
| 8347 | 1.00E-02 | 5.26E-02 | glial cell migration |
| 42436 | 1.00E-02 | 5.26E-02 | indole derivative catabolic process |
| 51414 | 1.00E-02 | 5.26E-02 | response to cortisol stimulus |
| 51593 | 1.00E-02 | 5.26E-02 | response to folic acid |
| 2922 | 1.00E-02 | 5.26E-02 | positive regulation of humoral immune response |
| 60710 | 1.00E-02 | 5.26E-02 | chorio-allantoic fusion |
| 46218 | 1.00E-02 | 5.26E-02 | indolalkylamine catabolic process |
| 46325 | 1.00E-02 | 5.26E-02 | negative regulation of glucose import |
| 46479 | 1.00E-02 | 5.26E-02 | glycosphingolipid catabolic process |
| 14060 | 1.00E-02 | 5.26E-02 | regulation of epinephrine secretion |
| 55078 | 1.00E-02 | 5.26E-02 | sodium ion homeostasis |
| 71622 | 1.00E-02 | 5.26E-02 | regulation of granulocyte chemotaxis |
| 30853 | 1.00E-02 | 5.26E-02 | negative regulation of granulocyte differentiation |
| 6534 | 1.00E-02 | 5.26E-02 | cysteine metabolic process |
| 6558 | 1.00E-02 | 5.26E-02 | L-phenylalanine metabolic process |
| 6559 | 1.00E-02 | 5.26E-02 | L-phenylalanine catabolic process |
| 6569 | 1.00E-02 | 5.26E-02 | tryptophan catabolic process |
| 32230 | 1.00E-02 | 5.26E-02 | positive regulation of synaptic transmission, GABAergic |
| 51099 | 1.01E-02 | 5.31E-02 | positive regulation of binding |
| 48667 | 1.01E-02 | 5.32E-02 | cell morphogenesis involved in neuron differentiation |
| 50679 | 1.03E-02 | 5.41E-02 | positive regulation of epithelial cell proliferation |
| 6112 | 1.03E-02 | 5.41E-02 | energy reserve metabolic process |
| 48660 | 1.03E-02 | 5.41E-02 | regulation of smooth muscle cell proliferation |
| 32769 | 1.04E-02 | 5.43E-02 | negative regulation of monooxygenase activity |
| 10718 | 1.04E-02 | 5.43E-02 | positive regulation of epithelial to mesenchymal transition |
| 43500 | 1.04E-02 | 5.43E-02 | muscle adaptation |
| 10770 | 1.04E-02 | 5.43E-02 | positive regulation of cell morphogenesis involved in differentiation |
| 45622 | 1.04E-02 | 5.43E-02 | regulation of T-helper cell differentiation |
| 6750 | 1.04E-02 | 5.43E-02 | glutathione biosynthetic process |
| 18210 | 1.05E-02 | 5.44E-02 | peptidyl-threonine modification |
| 51607 | 1.05E-02 | 5.44E-02 | defense response to virus |
| 70169 | 1.05E-02 | 5.44E-02 | positive regulation of biomineral formation |
| 45933 | 1.05E-02 | 5.44E-02 | positive regulation of muscle contraction |
| 46637 | 1.05E-02 | 5.44E-02 | regulation of alpha-beta T cell differentiation |
| 48701 | 1.05E-02 | 5.44E-02 | embryonic cranial skeleton morphogenesis |
| 8406 | 1.06E-02 | 5.44E-02 | gonad development |
| 2443 | 1.06E-02 | 5.47E-02 | leukocyte mediated immunity |
| 46883 | 1.06E-02 | 5.47E-02 | regulation of hormone secretion |
| 10551 | 1.06E-02 | 5.48E-02 | regulation of gene-specific transcription from RNA polymerase II promoter |
| 70167 | 1.09E-02 | 5.61E-02 | regulation of biomineral formation |
| 45862 | 1.09E-02 | 5.61E-02 | positive regulation of proteolysis |
| 30850 | 1.09E-02 | 5.61E-02 | prostate gland development |
| 46519 | 1.09E-02 | 5.61E-02 | sphingoid metabolic process |
| 32963 | 1.11E-02 | 5.68E-02 | collagen metabolic process |
| 9187 | 1.11E-02 | 5.68E-02 | cyclic nucleotide metabolic process |
| 45995 | 1.11E-02 | 5.68E-02 | regulation of embryonic development |
| 19933 | 1.13E-02 | 5.74E-02 | cAMP-mediated signaling |
| 33280 | 1.13E-02 | 5.76E-02 | response to vitamin D |
| 10149 | 1.13E-02 | 5.76E-02 | senescence |
| 60445 | 1.13E-02 | 5.76E-02 | branching involved in salivary gland morphogenesis |
| 48814 | 1.13E-02 | 5.76E-02 | regulation of dendrite morphogenesis |
| 50878 | 1.15E-02 | 5.84E-02 | regulation of body fluid levels |
| 30308 | 1.17E-02 | 5.94E-02 | negative regulation of cell growth |
| 48015 | 1.18E-02 | 5.96E-02 | phosphoinositide-mediated signaling |
| 8366 | 1.20E-02 | 6.05E-02 | axon ensheathment |
| 1570 | 1.20E-02 | 6.05E-02 | vasculogenesis |
| 7272 | 1.20E-02 | 6.05E-02 | ensheathment of neurons |
| 43254 | 1.24E-02 | 6.24E-02 | regulation of protein complex assembly |
| 44272 | 1.25E-02 | 6.29E-02 | sulfur compound biosynthetic process |
| 48609 | 1.29E-02 | 6.46E-02 | reproductive process in a multicellular organism |
| 32504 | 1.29E-02 | 6.46E-02 | multicellular organism reproduction |
| 50805 | 1.31E-02 | 6.55E-02 | negative regulation of synaptic transmission |
| 6071 | 1.31E-02 | 6.55E-02 | glycerol metabolic process |
| 6887 | 1.31E-02 | 6.57E-02 | exocytosis |
| 7265 | 1.31E-02 | 6.57E-02 | Ras protein signal transduction |
| 51093 | 1.32E-02 | 6.59E-02 | negative regulation of developmental process |
| 14706 | 1.33E-02 | 6.63E-02 | striated muscle tissue development |
| 16525 | 1.33E-02 | 6.63E-02 | negative regulation of angiogenesis |
| 50772 | 1.33E-02 | 6.63E-02 | positive regulation of axonogenesis |
| 45428 | 1.33E-02 | 6.63E-02 | regulation of nitric oxide biosynthetic process |
| 45621 | 1.33E-02 | 6.63E-02 | positive regulation of lymphocyte differentiation |
| 6081 | 1.33E-02 | 6.63E-02 | cellular aldehyde metabolic process |
| 6869 | 1.34E-02 | 6.65E-02 | lipid transport |
| 3008 | 1.36E-02 | 6.73E-02 | system process |
| 1541 | 1.38E-02 | 6.84E-02 | ovarian follicle development |
| 45787 | 1.40E-02 | 6.91E-02 | positive regulation of cell cycle |
| 44264 | 1.42E-02 | 7.02E-02 | cellular polysaccharide metabolic process |
| 31331 | 1.42E-02 | 7.02E-02 | positive regulation of cellular catabolic process |
| 7264 | 1.45E-02 | 7.16E-02 | small GTPase mediated signal transduction |
| 34381 | 1.46E-02 | 7.16E-02 | lipoprotein particle clearance |
| 60840 | 1.46E-02 | 7.16E-02 | artery development |
| 30728 | 1.46E-02 | 7.16E-02 | ovulation |
| 90181 | 1.46E-02 | 7.16E-02 | regulation of cholesterol metabolic process |
| 17145 | 1.46E-02 | 7.16E-02 | stem cell division |
| 1958 | 1.46E-02 | 7.16E-02 | endochondral ossification |
| 2886 | 1.46E-02 | 7.16E-02 | regulation of myeloid leukocyte mediated immunity |
| 14061 | 1.46E-02 | 7.16E-02 | regulation of norepinephrine secretion |
| 8045 | 1.46E-02 | 7.16E-02 | motor axon guidance |
| 48568 | 1.48E-02 | 7.25E-02 | embryonic organ development |
| 33157 | 1.50E-02 | 7.30E-02 | regulation of intracellular protein transport |
| 7193 | 1.50E-02 | 7.30E-02 | inhibition of adenylate cyclase activity by G-protein signaling pathway |
| 32368 | 1.50E-02 | 7.30E-02 | regulation of lipid transport |
| 32990 | 1.50E-02 | 7.32E-02 | cell part morphogenesis |
| 51641 | 1.54E-02 | 7.47E-02 | cellular localization |
| 6644 | 1.57E-02 | 7.64E-02 | phospholipid metabolic process |
| 1936 | 1.58E-02 | 7.68E-02 | regulation of endothelial cell proliferation |
| 35303 | 1.59E-02 | 7.68E-02 | regulation of dephosphorylation |
| 38 | 1.60E-02 | 7.72E-02 | very long-chain fatty acid metabolic process |
| 43255 | 1.60E-02 | 7.72E-02 | regulation of carbohydrate biosynthetic process |
| 43488 | 1.60E-02 | 7.72E-02 | regulation of mRNA stability |
| 43279 | 1.61E-02 | 7.74E-02 | response to alkaloid |
| 2757 | 1.61E-02 | 7.74E-02 | immune response-activating signal transduction |
| 7190 | 1.61E-02 | 7.74E-02 | activation of adenylate cyclase activity |
| 32386 | 1.63E-02 | 7.74E-02 | regulation of intracellular transport |
| 42416 | 1.64E-02 | 7.74E-02 | dopamine biosynthetic process |
| 34372 | 1.64E-02 | 7.74E-02 | very-low-density lipoprotein particle remodeling |
| 51145 | 1.64E-02 | 7.74E-02 | smooth muscle cell differentiation |
| 43206 | 1.64E-02 | 7.74E-02 | fibril organization |
| 43368 | 1.64E-02 | 7.74E-02 | positive T cell selection |
| 35313 | 1.64E-02 | 7.74E-02 | wound healing, spreading of epidermal cells |
| 2643 | 1.64E-02 | 7.74E-02 | regulation of tolerance induction |
| 2645 | 1.64E-02 | 7.74E-02 | positive regulation of tolerance induction |
| 10873 | 1.64E-02 | 7.74E-02 | positive regulation of cholesterol esterification |
| 44319 | 1.64E-02 | 7.74E-02 | wound healing, spreading of epithelial cells |
| 45652 | 1.64E-02 | 7.74E-02 | regulation of megakaryocyte differentiation |
| 45717 | 1.64E-02 | 7.74E-02 | negative regulation of fatty acid biosynthetic process |
| 45824 | 1.64E-02 | 7.74E-02 | negative regulation of innate immune response |
| 46146 | 1.64E-02 | 7.74E-02 | tetrahydrobiopterin metabolic process |
| 71383 | 1.64E-02 | 7.74E-02 | cellular response to steroid hormone stimulus |
| 71396 | 1.64E-02 | 7.74E-02 | cellular response to lipid |
| 6002 | 1.64E-02 | 7.74E-02 | fructose 6-phosphate metabolic process |
| 31116 | 1.64E-02 | 7.74E-02 | positive regulation of microtubule polymerization |
| 6568 | 1.64E-02 | 7.74E-02 | tryptophan metabolic process |
| 14912 | 1.64E-02 | 7.74E-02 | negative regulation of smooth muscle cell migration |
| 32229 | 1.64E-02 | 7.74E-02 | negative regulation of synaptic transmission, GABAergic |
| 10578 | 1.74E-02 | 8.16E-02 | regulation of adenylate cyclase activity involved in G-protein signaling pathway |
| 10579 | 1.74E-02 | 8.16E-02 | positive regulation of adenylate cyclase activity by G-protein signaling pathway |
| 31333 | 1.74E-02 | 8.16E-02 | negative regulation of protein complex assembly |
| 6767 | 1.74E-02 | 8.16E-02 | water-soluble vitamin metabolic process |
| 7189 | 1.74E-02 | 8.16E-02 | activation of adenylate cyclase activity by G-protein signaling pathway |
| 21700 | 1.77E-02 | 8.32E-02 | developmental maturation |
| 9408 | 1.78E-02 | 8.35E-02 | response to heat |
| 60249 | 1.80E-02 | 8.44E-02 | anatomical structure homeostasis |
| 2695 | 1.81E-02 | 8.47E-02 | negative regulation of leukocyte activation |
| 45762 | 1.81E-02 | 8.47E-02 | positive regulation of adenylate cyclase activity |
| 42692 | 1.82E-02 | 8.47E-02 | muscle cell differentiation |
| 45926 | 1.82E-02 | 8.47E-02 | negative regulation of growth |
| 6766 | 1.82E-02 | 8.49E-02 | vitamin metabolic process |
| 34103 | 1.85E-02 | 8.54E-02 | regulation of tissue remodeling |
| 42304 | 1.85E-02 | 8.54E-02 | regulation of fatty acid biosynthetic process |
| 1759 | 1.85E-02 | 8.54E-02 | induction of an organ |
| 50999 | 1.85E-02 | 8.54E-02 | regulation of nitric-oxide synthase activity |
| 10171 | 1.85E-02 | 8.54E-02 | body morphogenesis |
| 19835 | 1.85E-02 | 8.54E-02 | cytolysis |
| 30539 | 1.85E-02 | 8.54E-02 | male genitalia development |
| 31111 | 1.85E-02 | 8.54E-02 | negative regulation of microtubule polymerization or depolymerization |
| 9069 | 1.93E-02 | 8.70E-02 | serine family amino acid metabolic process |
| 60349 | 1.93E-02 | 8.70E-02 | bone morphogenesis |
| 30516 | 1.93E-02 | 8.70E-02 | regulation of axon extension |
| 10639 | 1.93E-02 | 8.70E-02 | negative regulation of organelle organization |
| 40008 | 1.95E-02 | 8.70E-02 | regulation of growth |
| 51209 | 1.97E-02 | 8.70E-02 | release of sequestered calcium ion into cytosol |
| 51282 | 1.97E-02 | 8.70E-02 | regulation of sequestering of calcium ion |
| 51283 | 1.97E-02 | 8.70E-02 | negative regulation of sequestering of calcium ion |
| 43114 | 1.97E-02 | 8.70E-02 | regulation of vascular permeability |
| 10575 | 1.97E-02 | 8.70E-02 | positive regulation vascular endothelial growth factor production |
| 43370 | 1.97E-02 | 8.70E-02 | regulation of CD4-positive, alpha beta T cell differentiation |
| 10744 | 1.97E-02 | 8.70E-02 | positive regulation of macrophage derived foam cell differentiation |
| 43536 | 1.97E-02 | 8.70E-02 | positive regulation of blood vessel endothelial cell migration |
| 10884 | 1.97E-02 | 8.70E-02 | positive regulation of lipid storage |
| 10977 | 1.97E-02 | 8.70E-02 | negative regulation of neuron projection development |
| 45086 | 1.97E-02 | 8.70E-02 | positive regulation of interleukin-2 biosynthetic process |
| 45922 | 1.97E-02 | 8.70E-02 | negative regulation of fatty acid metabolic process |
| 14059 | 1.97E-02 | 8.70E-02 | regulation of dopamine secretion |
| 42306 | 1.99E-02 | 8.70E-02 | regulation of protein import into nucleus |
| 42542 | 1.99E-02 | 8.70E-02 | response to hydrogen peroxide |
| 7519 | 1.99E-02 | 8.70E-02 | skeletal muscle tissue development |
| 46822 | 2.00E-02 | 8.70E-02 | regulation of nucleocytoplasmic transport |
| 9062 | 2.00E-02 | 8.70E-02 | fatty acid catabolic process |
| 50880 | 2.00E-02 | 8.70E-02 | regulation of blood vessel size |
| 35150 | 2.00E-02 | 8.70E-02 | regulation of tube size |
| 2429 | 2.00E-02 | 8.70E-02 | immune response-activating cell surface receptor signaling pathway |
| 7266 | 2.00E-02 | 8.70E-02 | Rho protein signal transduction |
| 7268 | 2.02E-02 | 8.70E-02 | synaptic transmission |
| 103 | 2.03E-02 | 8.70E-02 | sulfate assimilation |
| 32958 | 2.03E-02 | 8.70E-02 | inositol phosphate biosynthetic process |
| 33083 | 2.03E-02 | 8.70E-02 | regulation of immature T cell proliferation |
| 33630 | 2.03E-02 | 8.70E-02 | positive regulation of cell adhesion mediated by integrin |
| 9448 | 2.03E-02 | 8.70E-02 | gamma-aminobutyric acid metabolic process |
| 50667 | 2.03E-02 | 8.70E-02 | homocysteine metabolic process |
| 9756 | 2.03E-02 | 8.70E-02 | carbohydrate mediated signaling |
| 34333 | 2.03E-02 | 8.70E-02 | adherens junction assembly |
| 50798 | 2.03E-02 | 8.70E-02 | activated T cell proliferation |
| 34447 | 2.03E-02 | 8.70E-02 | very-low-density lipoprotein particle clearance |
| 18126 | 2.03E-02 | 8.70E-02 | protein amino acid hydroxylation |
| 1845 | 2.03E-02 | 8.70E-02 | phagolysosome assembly |
| 10193 | 2.03E-02 | 8.70E-02 | response to ozone |
| 51208 | 2.03E-02 | 8.70E-02 | sequestering of calcium ion |
| 43320 | 2.03E-02 | 8.70E-02 | natural killer cell degranulation |
| 35234 | 2.03E-02 | 8.70E-02 | germ cell programmed cell death |
| 35283 | 2.03E-02 | 8.70E-02 | central nervous system segmentation |
| 35284 | 2.03E-02 | 8.70E-02 | brain segmentation |
| 10757 | 2.03E-02 | 8.70E-02 | negative regulation of plasminogen activation |
| 51825 | 2.03E-02 | 8.70E-02 | adhesion to other organism involved in symbiotic interaction |
| 2676 | 2.03E-02 | 8.70E-02 | regulation of chronic inflammatory response |
| 2678 | 2.03E-02 | 8.70E-02 | positive regulation of chronic inflammatory response |
| 51856 | 2.03E-02 | 8.70E-02 | adhesion to symbiont |
| 10919 | 2.03E-02 | 8.70E-02 | regulation of inositol phosphate biosynthetic process |
| 10951 | 2.03E-02 | 8.70E-02 | negative regulation of endopeptidase activity |
| 2863 | 2.03E-02 | 8.70E-02 | positive regulation of inflammatory response to antigenic stimulus |
| 2866 | 2.03E-02 | 8.70E-02 | positive regulation of acute inflammatory response to antigenic stimulus |
| 3197 | 2.03E-02 | 8.70E-02 | endocardial cushion development |
| 60605 | 2.03E-02 | 8.70E-02 | tube lumen cavitation |
| 44254 | 2.03E-02 | 8.70E-02 | multicellular organismal protein catabolic process |
| 44256 | 2.03E-02 | 8.70E-02 | protein digestion |
| 44266 | 2.03E-02 | 8.70E-02 | multicellular organismal macromolecule catabolic process |
| 60662 | 2.03E-02 | 8.70E-02 | salivary gland cavitation |
| 60684 | 2.03E-02 | 8.70E-02 | epithelial-mesenchymal cell signaling |
| 61036 | 2.03E-02 | 8.70E-02 | positive regulation of cartilage development |
| 45653 | 2.03E-02 | 8.70E-02 | negative regulation of megakaryocyte differentiation |
| 45759 | 2.03E-02 | 8.70E-02 | negative regulation of action potential |
| 45916 | 2.03E-02 | 8.70E-02 | negative regulation of complement activation |
| 71071 | 2.03E-02 | 8.70E-02 | regulation of phospholipid biosynthetic process |
| 71072 | 2.03E-02 | 8.70E-02 | negative regulation of phospholipid biosynthetic process |
| 46881 | 2.03E-02 | 8.70E-02 | positive regulation of follicle-stimulating hormone secretion |
| 71503 | 2.03E-02 | 8.70E-02 | response to heparin |
| 30730 | 2.03E-02 | 8.70E-02 | sequestering of triglyceride |
| 30953 | 2.03E-02 | 8.70E-02 | spindle astral microtubule organization |
| 6564 | 2.03E-02 | 8.70E-02 | L-serine biosynthetic process |
| 14832 | 2.03E-02 | 8.70E-02 | urinary bladder smooth muscle contraction |
| 14848 | 2.03E-02 | 8.70E-02 | urinary tract smooth muscle contraction |
| 6689 | 2.03E-02 | 8.70E-02 | ganglioside catabolic process |
| 6828 | 2.03E-02 | 8.70E-02 | manganese ion transport |
| 6931 | 2.03E-02 | 8.70E-02 | substrate-bound cell migration, cell attachment to substrate |
| 31584 | 2.03E-02 | 8.70E-02 | activation of phospholipase D activity |
| 7207 | 2.03E-02 | 8.70E-02 | activation of phospholipase C activity by muscarinic acetylcholine receptor signaling pathway |
| 32224 | 2.03E-02 | 8.70E-02 | positive regulation of synaptic transmission, cholinergic |
| 32278 | 2.03E-02 | 8.70E-02 | positive regulation of gonadotropin secretion |
| 32674 | 2.03E-02 | 8.70E-02 | regulation of interleukin-5 production |
| 32763 | 2.03E-02 | 8.70E-02 | regulation of mast cell cytokine production |
| 31281 | 2.04E-02 | 8.72E-02 | positive regulation of cyclase activity |
| 2274 | 2.05E-02 | 8.76E-02 | myeloid leukocyte activation |
| 51591 | 2.05E-02 | 8.76E-02 | response to cAMP |
| 43122 | 2.11E-02 | 8.98E-02 | regulation of I-kappaB kinase/NF-kappaB cascade |
| 48592 | 2.11E-02 | 9.00E-02 | eye morphogenesis |
| 43009 | 2.12E-02 | 9.01E-02 | chordate embryonic development |
| 45792 | 2.12E-02 | 9.01E-02 | negative regulation of cell size |
| 32583 | 2.15E-02 | 9.15E-02 | regulation of gene-specific transcription |
| 6885 | 2.19E-02 | 9.28E-02 | regulation of pH |
| 30001 | 2.21E-02 | 9.36E-02 | metal ion transport |
| 30162 | 2.21E-02 | 9.36E-02 | regulation of proteolysis |
| 50731 | 2.28E-02 | 9.65E-02 | positive regulation of peptidyl-tyrosine phosphorylation |
| 42417 | 2.30E-02 | 9.65E-02 | dopamine metabolic process |
| 50764 | 2.30E-02 | 9.65E-02 | regulation of phagocytosis |
| 50773 | 2.30E-02 | 9.65E-02 | regulation of dendrite development |
| 2285 | 2.30E-02 | 9.65E-02 | lymphocyte activation involved in immune response |
| 44246 | 2.30E-02 | 9.65E-02 | regulation of multicellular organismal metabolic process |
| 45646 | 2.30E-02 | 9.65E-02 | regulation of erythrocyte differentiation |
| 45884 | 2.30E-02 | 9.65E-02 | regulation of survival gene product expression |
| 6041 | 2.30E-02 | 9.65E-02 | glucosamine metabolic process |
| 6044 | 2.30E-02 | 9.65E-02 | N-acetylglucosamine metabolic process |
| 70507 | 2.30E-02 | 9.65E-02 | regulation of microtubule cytoskeleton organization |
| 8645 | 2.31E-02 | 9.67E-02 | hexose transport |
| 1101 | 2.31E-02 | 9.67E-02 | response to acid |
| 3014 | 2.31E-02 | 9.67E-02 | renal system process |
| 15758 | 2.31E-02 | 9.67E-02 | glucose transport |
| 8344 | 2.32E-02 | 9.68E-02 | adult locomotory behavior |
| 2706 | 2.32E-02 | 9.68E-02 | regulation of lymphocyte mediated immunity |
| 15698 | 2.32E-02 | 9.68E-02 | inorganic anion transport |
| 31329 | 2.37E-02 | 9.88E-02 | regulation of cellular catabolic process |
| 19318 | 2.38E-02 | 9.92E-02 | hexose metabolic process |
| 34637 | 2.45E-02 | 1.01E-01 | cellular carbohydrate biosynthetic process |
| 60538 | 2.45E-02 | 1.01E-01 | skeletal muscle organ development |
| 43388 | 2.45E-02 | 1.01E-01 | positive regulation of DNA binding |
| 7409 | 2.46E-02 | 1.01E-01 | axonogenesis |
| 90183 | 2.46E-02 | 1.01E-01 | regulation of kidney development |
| 33002 | 2.46E-02 | 1.01E-01 | muscle cell proliferation |
| 1991 | 2.46E-02 | 1.01E-01 | regulation of systemic arterial blood pressure by circulatory renin-angiotensin |
| 35024 | 2.46E-02 | 1.01E-01 | negative regulation of Rho protein signal transduction |
| 2714 | 2.46E-02 | 1.01E-01 | positive regulation of B cell mediated immunity |
| 2891 | 2.46E-02 | 1.01E-01 | positive regulation of immunoglobulin mediated immune response |
| 19377 | 2.46E-02 | 1.01E-01 | glycolipid catabolic process |
| 3078 | 2.46E-02 | 1.01E-01 | regulation of natriuresis |
| 60638 | 2.46E-02 | 1.01E-01 | mesenchymal-epithelial cell signaling |
| 30852 | 2.46E-02 | 1.01E-01 | regulation of granulocyte differentiation |
| 6563 | 2.46E-02 | 1.01E-01 | L-serine metabolic process |
| 6596 | 2.46E-02 | 1.01E-01 | polyamine biosynthetic process |
| 7217 | 2.46E-02 | 1.01E-01 | tachykinin receptor signaling pathway |
| 31958 | 2.46E-02 | 1.01E-01 | corticosteroid receptor signaling pathway |
| 9792 | 2.49E-02 | 1.02E-01 | embryonic development ending in birth or egg hatching |
| 51726 | 2.51E-02 | 1.03E-01 | regulation of cell cycle |
| 165 | 2.52E-02 | 1.03E-01 | MAPKKK cascade |
| 6487 | 2.54E-02 | 1.04E-01 | protein amino acid N-linked glycosylation |
| 51349 | 2.55E-02 | 1.04E-01 | positive regulation of lyase activity |
| 45637 | 2.56E-02 | 1.04E-01 | regulation of myeloid cell differentiation |
| 34375 | 2.58E-02 | 1.05E-01 | high-density lipoprotein particle remodeling |
| 34394 | 2.58E-02 | 1.05E-01 | protein localization at cell surface |
| 51496 | 2.58E-02 | 1.05E-01 | positive regulation of stress fiber assembly |
| 60713 | 2.58E-02 | 1.05E-01 | labyrinthine layer morphogenesis |
| 3382 | 2.58E-02 | 1.05E-01 | epithelial cell morphogenesis |
| 70328 | 2.58E-02 | 1.05E-01 | triglyceride homeostasis |
| 45773 | 2.58E-02 | 1.05E-01 | positive regulation of axon extension |
| 6835 | 2.58E-02 | 1.05E-01 | dicarboxylic acid transport |
| 6884 | 2.58E-02 | 1.05E-01 | cell volume homeostasis |
| 48265 | 2.58E-02 | 1.05E-01 | response to pain |
| 45596 | 2.59E-02 | 1.05E-01 | negative regulation of cell differentiation |
| 2699 | 2.62E-02 | 1.06E-01 | positive regulation of immune effector process |
| 45927 | 2.63E-02 | 1.06E-01 | positive regulation of growth |
| 7292 | 2.70E-02 | 1.09E-01 | female gamete generation |
| 9110 | 2.74E-02 | 1.10E-01 | vitamin biosynthetic process |
| 50810 | 2.74E-02 | 1.10E-01 | regulation of steroid biosynthetic process |
| 51353 | 2.74E-02 | 1.10E-01 | positive regulation of oxidoreductase activity |
| 2705 | 2.74E-02 | 1.10E-01 | positive regulation of leukocyte mediated immunity |
| 2708 | 2.74E-02 | 1.10E-01 | positive regulation of lymphocyte mediated immunity |
| 51970 | 2.74E-02 | 1.10E-01 | negative regulation of transmission of nerve impulse |
| 45582 | 2.74E-02 | 1.10E-01 | positive regulation of T cell differentiation |
| 70555 | 2.74E-02 | 1.10E-01 | response to interleukin-1 |
| 21675 | 2.74E-02 | 1.10E-01 | nerve development |
| 31341 | 2.74E-02 | 1.10E-01 | regulation of cell killing |
| 7015 | 2.81E-02 | 1.12E-01 | actin filament organization |
| 32845 | 2.81E-02 | 1.12E-01 | negative regulation of homeostatic process |
| 33344 | 2.81E-02 | 1.12E-01 | cholesterol efflux |
| 1836 | 2.81E-02 | 1.12E-01 | release of cytochrome c from mitochondria |
| 2062 | 2.81E-02 | 1.12E-01 | chondrocyte differentiation |
| 10469 | 2.81E-02 | 1.12E-01 | regulation of receptor activity |
| 21545 | 2.81E-02 | 1.12E-01 | cranial nerve development |
| 30514 | 2.81E-02 | 1.12E-01 | negative regulation of BMP signaling pathway |
| 48259 | 2.81E-02 | 1.12E-01 | regulation of receptor-mediated endocytosis |
| 50808 | 2.83E-02 | 1.12E-01 | synapse organization |
| 1657 | 2.83E-02 | 1.12E-01 | ureteric bud development |
| 2764 | 2.83E-02 | 1.12E-01 | immune response-regulating signaling pathway |
| 9101 | 2.85E-02 | 1.13E-01 | glycoprotein biosynthetic process |
| 51235 | 2.87E-02 | 1.13E-01 | maintenance of location |
| 30099 | 2.89E-02 | 1.14E-01 | myeloid cell differentiation |
| 5977 | 2.93E-02 | 1.16E-01 | glycogen metabolic process |
| 8629 | 2.93E-02 | 1.16E-01 | induction of apoptosis by intracellular signals |
| 7588 | 2.93E-02 | 1.16E-01 | excretion |
| 45732 | 2.97E-02 | 1.17E-01 | positive regulation of protein catabolic process |
| 6029 | 2.97E-02 | 1.17E-01 | proteoglycan metabolic process |
| 6109 | 2.97E-02 | 1.17E-01 | regulation of carbohydrate metabolic process |
| 48193 | 2.98E-02 | 1.17E-01 | Golgi vesicle transport |
| 32868 | 3.04E-02 | 1.19E-01 | response to insulin stimulus |
| 30799 | 3.04E-02 | 1.19E-01 | regulation of cyclic nucleotide metabolic process |
| 30814 | 3.09E-02 | 1.21E-01 | regulation of cAMP metabolic process |
| 30323 | 3.12E-02 | 1.22E-01 | respiratory tube development |
| 45761 | 3.13E-02 | 1.23E-01 | regulation of adenylate cyclase activity |
| 6958 | 3.21E-02 | 1.26E-01 | complement activation, classical pathway |
| 33559 | 3.27E-02 | 1.27E-01 | unsaturated fatty acid metabolic process |
| 51350 | 3.27E-02 | 1.27E-01 | negative regulation of lyase activity |
| 14031 | 3.27E-02 | 1.27E-01 | mesenchymal cell development |
| 31280 | 3.27E-02 | 1.27E-01 | negative regulation of cyclase activity |
| 7194 | 3.27E-02 | 1.27E-01 | negative regulation of adenylate cyclase activity |
| 33574 | 3.29E-02 | 1.27E-01 | response to testosterone stimulus |
| 50995 | 3.29E-02 | 1.27E-01 | negative regulation of lipid catabolic process |
| 51354 | 3.29E-02 | 1.27E-01 | negative regulation of oxidoreductase activity |
| 44253 | 3.29E-02 | 1.27E-01 | positive regulation of multicellular organismal metabolic process |
| 21955 | 3.29E-02 | 1.27E-01 | central nervous system neuron axonogenesis |
| 14048 | 3.29E-02 | 1.27E-01 | regulation of glutamate secretion |
| 14068 | 3.29E-02 | 1.27E-01 | positive regulation of phosphoinositide 3-kinase cascade |
| 6144 | 3.29E-02 | 1.27E-01 | purine base metabolic process |
| 31290 | 3.29E-02 | 1.27E-01 | retinal ganglion cell axon guidance |
| 43123 | 3.32E-02 | 1.28E-01 | positive regulation of I-kappaB kinase/NF-kappaB cascade |
| 6665 | 3.35E-02 | 1.29E-01 | sphingolipid metabolic process |
| 44042 | 3.36E-02 | 1.29E-01 | glucan metabolic process |
| 6073 | 3.36E-02 | 1.29E-01 | cellular glucan metabolic process |
| 2768 | 3.36E-02 | 1.29E-01 | immune response-regulating cell surface receptor signaling pathway |
| 46165 | 3.36E-02 | 1.29E-01 | alcohol biosynthetic process |
| 43269 | 3.36E-02 | 1.29E-01 | regulation of ion transport |
| 42098 | 3.39E-02 | 1.30E-01 | T cell proliferation |
| 9395 | 3.39E-02 | 1.30E-01 | phospholipid catabolic process |
| 30048 | 3.39E-02 | 1.30E-01 | actin filament-based movement |
| 32880 | 3.39E-02 | 1.30E-01 | regulation of protein localization |
| 70201 | 3.42E-02 | 1.30E-01 | regulation of establishment of protein localization |
| 42135 | 3.46E-02 | 1.30E-01 | neurotransmitter catabolic process |
| 34370 | 3.46E-02 | 1.30E-01 | triglyceride-rich lipoprotein particle remodeling |
| 42572 | 3.46E-02 | 1.30E-01 | retinol metabolic process |
| 43090 | 3.46E-02 | 1.30E-01 | amino acid import |
| 43129 | 3.46E-02 | 1.30E-01 | surfactant homeostasis |
| 10922 | 3.46E-02 | 1.30E-01 | positive regulation of phosphatase activity |
| 44062 | 3.46E-02 | 1.30E-01 | regulation of excretion |
| 19934 | 3.46E-02 | 1.30E-01 | cGMP-mediated signaling |
| 45540 | 3.46E-02 | 1.30E-01 | regulation of cholesterol biosynthetic process |
| 45749 | 3.46E-02 | 1.30E-01 | negative regulation of S phase of mitotic cell cycle |
| 46685 | 3.46E-02 | 1.30E-01 | response to arsenic |
| 6465 | 3.46E-02 | 1.30E-01 | signal peptide processing |
| 31112 | 3.46E-02 | 1.30E-01 | positive regulation of microtubule polymerization or depolymerization |
| 6570 | 3.46E-02 | 1.30E-01 | tyrosine metabolic process |
| 6599 | 3.46E-02 | 1.30E-01 | phosphagen metabolic process |
| 6600 | 3.46E-02 | 1.30E-01 | creatine metabolic process |
| 6911 | 3.46E-02 | 1.30E-01 | phagocytosis, engulfment |
| 31620 | 3.46E-02 | 1.30E-01 | regulation of fever |
| 31622 | 3.46E-02 | 1.30E-01 | positive regulation of fever |
| 48103 | 3.46E-02 | 1.30E-01 | somatic stem cell division |
| 40020 | 3.46E-02 | 1.30E-01 | regulation of meiosis |
| 15988 | 3.46E-02 | 1.30E-01 | energy coupled proton transport, against electrochemical gradient |
| 15991 | 3.46E-02 | 1.30E-01 | ATP hydrolysis coupled proton transport |
| 48875 | 3.46E-02 | 1.30E-01 | chemical homeostasis within a tissue |
| 32673 | 3.46E-02 | 1.30E-01 | regulation of interleukin-4 production |
| 30217 | 3.47E-02 | 1.30E-01 | T cell differentiation |
| 55088 | 3.47E-02 | 1.30E-01 | lipid homeostasis |
| 9894 | 3.47E-02 | 1.30E-01 | regulation of catabolic process |
| 31279 | 3.65E-02 | 1.36E-01 | regulation of cyclase activity |
| 10959 | 3.67E-02 | 1.36E-01 | regulation of metal ion transport |
| 60443 | 3.73E-02 | 1.36E-01 | mammary gland morphogenesis |
| 60675 | 3.73E-02 | 1.36E-01 | ureteric bud morphogenesis |
| 30168 | 3.73E-02 | 1.36E-01 | platelet activation |
| 30510 | 3.73E-02 | 1.36E-01 | regulation of BMP signaling pathway |
| 7200 | 3.77E-02 | 1.36E-01 | activation of phospholipase C activity by G-protein coupled receptor protein signaling pathway coupled to IP3 second messenger |
| 90101 | 3.77E-02 | 1.36E-01 | negative regulation of transmembrane receptor protein serine/threonine kinase signaling pathway |
| 2064 | 3.83E-02 | 1.36E-01 | epithelial cell development |
| 6940 | 3.83E-02 | 1.36E-01 | regulation of smooth muscle contraction |
| 32803 | 3.84E-02 | 1.36E-01 | regulation of low-density lipoprotein receptor catabolic process |
| 32908 | 3.84E-02 | 1.36E-01 | regulation of transforming growth factor-beta1 production |
| 90303 | 3.84E-02 | 1.36E-01 | positive regulation of wound healing |
| 288 | 3.84E-02 | 1.36E-01 | nuclear-transcribed mRNA catabolic process, deadenylation-dependent decay |
| 33145 | 3.84E-02 | 1.36E-01 | positive regulation of steroid hormone receptor signaling pathway |
| 33148 | 3.84E-02 | 1.36E-01 | positive regulation of estrogen receptor signaling pathway |
| 33240 | 3.84E-02 | 1.36E-01 | positive regulation of cellular amine metabolic process |
| 17121 | 3.84E-02 | 1.36E-01 | phospholipid scrambling |
| 33631 | 3.84E-02 | 1.36E-01 | cell-cell adhesion mediated by integrin |
| 33632 | 3.84E-02 | 1.36E-01 | regulation of cell-cell adhesion mediated by integrin |
| 42167 | 3.84E-02 | 1.36E-01 | heme catabolic process |
| 34204 | 3.84E-02 | 1.36E-01 | lipid translocation |
| 42420 | 3.84E-02 | 1.36E-01 | dopamine catabolic process |
| 50651 | 3.84E-02 | 1.36E-01 | dermatan sulfate proteoglycan biosynthetic process |
| 1554 | 3.84E-02 | 1.36E-01 | luteolysis |
| 51014 | 3.84E-02 | 1.36E-01 | actin filament severing |
| 51045 | 3.84E-02 | 1.36E-01 | negative regulation of membrane protein ectodomain proteolysis |
| 1955 | 3.84E-02 | 1.36E-01 | blood vessel maturation |
| 43001 | 3.84E-02 | 1.36E-01 | Golgi to plasma membrane protein transport |
| 2042 | 3.84E-02 | 1.36E-01 | cell migration involved in sprouting angiogenesis |
| 43116 | 3.84E-02 | 1.36E-01 | negative regulation of vascular permeability |
| 2323 | 3.84E-02 | 1.36E-01 | natural killer cell activation involved in immune response |
| 10572 | 3.84E-02 | 1.36E-01 | positive regulation of platelet activation |
| 10613 | 3.84E-02 | 1.36E-01 | positive regulation of cardiac muscle hypertrophy |
| 51597 | 3.84E-02 | 1.36E-01 | response to methylmercury |
| 51709 | 3.84E-02 | 1.36E-01 | regulation of killing of cells of another organism |
| 10755 | 3.84E-02 | 1.36E-01 | regulation of plasminogen activation |
| 10799 | 3.84E-02 | 1.36E-01 | regulation of peptidyl-threonine phosphorylation |
| 19067 | 3.84E-02 | 1.36E-01 | viral assembly, maturation, egress, and release |
| 51901 | 3.84E-02 | 1.36E-01 | positive regulation of mitochondrial depolarization |
| 10954 | 3.84E-02 | 1.36E-01 | positive regulation of protein maturation by peptide bond cleavage |
| 2921 | 3.84E-02 | 1.36E-01 | negative regulation of humoral immune response |
| 19374 | 3.84E-02 | 1.36E-01 | galactolipid metabolic process |
| 60397 | 3.84E-02 | 1.36E-01 | JAK-STAT cascade involved in growth hormone signaling pathway |
| 3079 | 3.84E-02 | 1.36E-01 | positive regulation of natriuresis |
| 60528 | 3.84E-02 | 1.36E-01 | secretory columnal luminar epithelial cell differentiation involved in prostate glandular acinus development |
| 19695 | 3.84E-02 | 1.36E-01 | choline metabolic process |
| 19852 | 3.84E-02 | 1.36E-01 | L-ascorbic acid metabolic process |
| 45332 | 3.84E-02 | 1.36E-01 | phospholipid translocation |
| 45650 | 3.84E-02 | 1.36E-01 | negative regulation of macrophage differentiation |
| 46113 | 3.84E-02 | 1.36E-01 | nucleobase catabolic process |
| 21571 | 3.84E-02 | 1.36E-01 | rhombomere 5 development |
| 46149 | 3.84E-02 | 1.36E-01 | pigment catabolic process |
| 21631 | 3.84E-02 | 1.36E-01 | optic nerve morphogenesis |
| 46322 | 3.84E-02 | 1.36E-01 | negative regulation of fatty acid oxidation |
| 46475 | 3.84E-02 | 1.36E-01 | glycerophospholipid catabolic process |
| 71277 | 3.84E-02 | 1.36E-01 | cellular response to calcium ion |
| 71326 | 3.84E-02 | 1.36E-01 | cellular response to monosaccharide stimulus |
| 71331 | 3.84E-02 | 1.36E-01 | cellular response to hexose stimulus |
| 71333 | 3.84E-02 | 1.36E-01 | cellular response to glucose stimulus |
| 46880 | 3.84E-02 | 1.36E-01 | regulation of follicle-stimulating hormone secretion |
| 46882 | 3.84E-02 | 1.36E-01 | negative regulation of follicle-stimulating hormone secretion |
| 6021 | 3.84E-02 | 1.36E-01 | inositol biosynthetic process |
| 22601 | 3.84E-02 | 1.36E-01 | menstrual cycle phase |
| 6549 | 3.84E-02 | 1.36E-01 | isoleucine metabolic process |
| 14742 | 3.84E-02 | 1.36E-01 | positive regulation of muscle hypertrophy |
| 6681 | 3.84E-02 | 1.36E-01 | galactosylceramide metabolic process |
| 31340 | 3.84E-02 | 1.36E-01 | positive regulation of vesicle fusion |
| 31649 | 3.84E-02 | 1.36E-01 | heat generation |
| 48318 | 3.84E-02 | 1.36E-01 | axial mesoderm development |
| 7638 | 3.84E-02 | 1.36E-01 | mechanosensory behavior |
| 32222 | 3.84E-02 | 1.36E-01 | regulation of synaptic transmission, cholinergic |
| 32276 | 3.84E-02 | 1.36E-01 | regulation of gonadotropin secretion |
| 32277 | 3.84E-02 | 1.36E-01 | negative regulation of gonadotropin secretion |
| 48861 | 3.84E-02 | 1.36E-01 | leukemia inhibitory factor signaling pathway |
| 90025 | 3.84E-02 | 1.36E-01 | regulation of monocyte chemotaxis |
| 90026 | 3.84E-02 | 1.36E-01 | positive regulation of monocyte chemotaxis |
| 19538 | 3.91E-02 | 1.38E-01 | protein metabolic process |
| 10565 | 3.97E-02 | 1.40E-01 | regulation of cellular ketone metabolic process |
| 6812 | 3.99E-02 | 1.41E-01 | cation transport |
| 42364 | 4.04E-02 | 1.42E-01 | water-soluble vitamin biosynthetic process |
| 3156 | 4.04E-02 | 1.42E-01 | regulation of organ formation |
| 6687 | 4.04E-02 | 1.42E-01 | glycosphingolipid metabolic process |
| 32371 | 4.04E-02 | 1.42E-01 | regulation of sterol transport |
| 32374 | 4.04E-02 | 1.42E-01 | regulation of cholesterol transport |
| 6814 | 4.07E-02 | 1.43E-01 | sodium ion transport |
| 16486 | 4.10E-02 | 1.43E-01 | peptide hormone processing |
| 32885 | 4.10E-02 | 1.43E-01 | regulation of polysaccharide biosynthetic process |
| 32965 | 4.10E-02 | 1.43E-01 | regulation of collagen biosynthetic process |
| 34341 | 4.10E-02 | 1.43E-01 | response to interferon-gamma |
| 50766 | 4.10E-02 | 1.43E-01 | positive regulation of phagocytosis |
| 51058 | 4.10E-02 | 1.43E-01 | negative regulation of small GTPase mediated signal transduction |
| 2244 | 4.10E-02 | 1.43E-01 | hemopoietic progenitor cell differentiation |
| 10962 | 4.10E-02 | 1.43E-01 | regulation of glucan biosynthetic process |
| 45940 | 4.10E-02 | 1.43E-01 | positive regulation of steroid metabolic process |
| 70542 | 4.10E-02 | 1.43E-01 | response to fatty acid |
| 30035 | 4.10E-02 | 1.43E-01 | microspike assembly |
| 46686 | 4.10E-02 | 1.43E-01 | response to cadmium ion |
| 14066 | 4.10E-02 | 1.43E-01 | regulation of phosphoinositide 3-kinase cascade |
| 5979 | 4.10E-02 | 1.43E-01 | regulation of glycogen biosynthetic process |
| 30947 | 4.10E-02 | 1.43E-01 | regulation of vascular endothelial growth factor receptor signaling pathway |
| 6536 | 4.10E-02 | 1.43E-01 | glutamate metabolic process |
| 48566 | 4.10E-02 | 1.43E-01 | embryonic digestive tract development |
| 7599 | 4.16E-02 | 1.45E-01 | hemostasis |
| 44249 | 4.22E-02 | 1.46E-01 | cellular biosynthetic process |
| 9895 | 4.22E-02 | 1.46E-01 | negative regulation of catabolic process |
| 15674 | 4.22E-02 | 1.46E-01 | di-, tri-valent inorganic cation transport |
| 51339 | 4.24E-02 | 1.47E-01 | regulation of lyase activity |
| 51091 | 4.30E-02 | 1.49E-01 | positive regulation of transcription factor activity |
| 90047 | 4.30E-02 | 1.49E-01 | positive regulation of transcription regulator activity |
| 2455 | 4.31E-02 | 1.49E-01 | humoral immune response mediated by circulating immunoglobulin |
| 46634 | 4.31E-02 | 1.49E-01 | regulation of alpha-beta T cell activation |
| 31645 | 4.31E-02 | 1.49E-01 | negative regulation of neurological system process |
| 32943 | 4.33E-02 | 1.49E-01 | mononuclear cell proliferation |
| 3205 | 4.33E-02 | 1.49E-01 | cardiac chamber development |
| 30301 | 4.33E-02 | 1.49E-01 | cholesterol transport |
| 15918 | 4.33E-02 | 1.49E-01 | sterol transport |
| 1701 | 4.36E-02 | 1.50E-01 | in utero embryonic development |
| 7423 | 4.36E-02 | 1.50E-01 | sensory organ development |
| 7417 | 4.46E-02 | 1.53E-01 | central nervous system development |
| 7411 | 4.62E-02 | 1.57E-01 | axon guidance |
| 33006 | 4.64E-02 | 1.57E-01 | regulation of mast cell activation involved in immune response |
| 33700 | 4.64E-02 | 1.57E-01 | phospholipid efflux |
| 34446 | 4.64E-02 | 1.57E-01 | substrate adhesion-dependent cell spreading |
| 51000 | 4.64E-02 | 1.57E-01 | positive regulation of nitric-oxide synthase activity |
| 1935 | 4.64E-02 | 1.57E-01 | endothelial cell proliferation |
| 10226 | 4.64E-02 | 1.57E-01 | response to lithium ion |
| 43304 | 4.64E-02 | 1.57E-01 | regulation of mast cell degranulation |
| 51918 | 4.64E-02 | 1.57E-01 | negative regulation of fibrinolysis |
| 2828 | 4.64E-02 | 1.57E-01 | regulation of T-helper 2 type immune response |
| 44060 | 4.64E-02 | 1.57E-01 | regulation of endocrine process |
| 45061 | 4.64E-02 | 1.57E-01 | thymic T cell selection |
| 45725 | 4.64E-02 | 1.57E-01 | positive regulation of glycogen biosynthetic process |
| 71241 | 4.64E-02 | 1.57E-01 | cellular response to inorganic substance |
| 71248 | 4.64E-02 | 1.57E-01 | cellular response to metal ion |
| 14065 | 4.64E-02 | 1.57E-01 | phosphoinositide 3-kinase cascade |
| 55117 | 4.64E-02 | 1.57E-01 | regulation of cardiac muscle contraction |
| 72073 | 4.64E-02 | 1.57E-01 | kidney epithelium development |
| 6595 | 4.64E-02 | 1.57E-01 | polyamine metabolic process |
| 31650 | 4.64E-02 | 1.57E-01 | regulation of heat generation |
| 31652 | 4.64E-02 | 1.57E-01 | positive regulation of heat generation |
| 32026 | 4.64E-02 | 1.57E-01 | response to magnesium ion |
| 30802 | 4.64E-02 | 1.57E-01 | regulation of cyclic nucleotide biosynthetic process |
| 30808 | 4.64E-02 | 1.57E-01 | regulation of nucleotide biosynthetic process |
| 46661 | 4.66E-02 | 1.57E-01 | male sex differentiation |
| 9891 | 4.67E-02 | 1.57E-01 | positive regulation of biosynthetic process |
| 55002 | 4.71E-02 | 1.59E-01 | striated muscle cell development |
| 96 | 4.76E-02 | 1.60E-01 | sulfur amino acid metabolic process |
| 42058 | 4.76E-02 | 1.60E-01 | regulation of epidermal growth factor receptor signaling pathway |
| 1910 | 4.76E-02 | 1.60E-01 | regulation of leukocyte mediated cytotoxicity |
| 6942 | 4.76E-02 | 1.60E-01 | regulation of striated muscle contraction |
| 48730 | 4.76E-02 | 1.60E-01 | epidermis morphogenesis |
| 6140 | 4.88E-02 | 1.63E-01 | regulation of nucleotide metabolic process |
| 2263 | 4.88E-02 | 1.63E-01 | cell activation involved in immune response |
| 2366 | 4.88E-02 | 1.63E-01 | leukocyte activation involved in immune response |
| 19058 | 4.88E-02 | 1.63E-01 | viral infectious cycle |
| 70661 | 4.88E-02 | 1.63E-01 | leukocyte proliferation |
| 9311 | 4.94E-02 | 1.65E-01 | oligosaccharide metabolic process |
| 50728 | 4.94E-02 | 1.65E-01 | negative regulation of inflammatory response |
| 1938 | 4.94E-02 | 1.65E-01 | positive regulation of endothelial cell proliferation |
| 31334 | 4.94E-02 | 1.65E-01 | positive regulation of protein complex assembly |
| 32881 | 5.02E-02 | 1.66E-01 | regulation of polysaccharide metabolic process |
| 9081 | 5.02E-02 | 1.66E-01 | branched chain family amino acid metabolic process |
| 42311 | 5.02E-02 | 1.66E-01 | vasodilation |
| 10596 | 5.02E-02 | 1.66E-01 | negative regulation of endothelial cell migration |
| 10712 | 5.02E-02 | 1.66E-01 | regulation of collagen metabolic process |
| 2709 | 5.02E-02 | 1.66E-01 | regulation of T cell mediated immunity |
| 43691 | 5.02E-02 | 1.66E-01 | reverse cholesterol transport |
| 60135 | 5.02E-02 | 1.66E-01 | maternal process involved in female pregnancy |
| 60350 | 5.02E-02 | 1.66E-01 | endochondral bone morphogenesis |
| 70873 | 5.02E-02 | 1.66E-01 | regulation of glycogen metabolic process |
| 46513 | 5.02E-02 | 1.66E-01 | ceramide biosynthetic process |
| 6000 | 5.02E-02 | 1.66E-01 | fructose metabolic process |
| 71695 | 5.02E-02 | 1.66E-01 | anatomical structure maturation |
| 32271 | 5.03E-02 | 1.66E-01 | regulation of protein polymerization |
| 7498 | 5.06E-02 | 1.67E-01 | mesoderm development |
| 30817 | 5.09E-02 | 1.68E-01 | regulation of cAMP biosynthetic process |
| 9150 | 5.09E-02 | 1.68E-01 | purine ribonucleotide metabolic process |
| 50817 | 5.23E-02 | 1.72E-01 | coagulation |
| 7596 | 5.23E-02 | 1.72E-01 | blood coagulation |
| 51649 | 5.24E-02 | 1.72E-01 | establishment of localization in cell |
| 32582 | 5.43E-02 | 1.78E-01 | negative regulation of gene-specific transcription |
| 60541 | 5.43E-02 | 1.78E-01 | respiratory system development |
| 16051 | 5.43E-02 | 1.78E-01 | carbohydrate biosynthetic process |
| 70372 | 5.48E-02 | 1.80E-01 | regulation of ERK1 and ERK2 cascade |
| 30534 | 5.48E-02 | 1.80E-01 | adult behavior |
| 9259 | 5.52E-02 | 1.81E-01 | ribonucleotide metabolic process |
| 50873 | 5.55E-02 | 1.81E-01 | brown fat cell differentiation |
| 45168 | 5.55E-02 | 1.81E-01 | cell-cell signaling involved in cell fate commitment |
| 45429 | 5.55E-02 | 1.81E-01 | positive regulation of nitric oxide biosynthetic process |
| 45777 | 5.55E-02 | 1.81E-01 | positive regulation of blood pressure |
| 31128 | 5.55E-02 | 1.81E-01 | developmental induction |
| 7618 | 5.55E-02 | 1.81E-01 | mating |
| 8306 | 5.62E-02 | 1.83E-01 | associative learning |
| 8630 | 5.62E-02 | 1.83E-01 | DNA damage response, signal transduction resulting in induction of apoptosis |
| 45740 | 5.62E-02 | 1.83E-01 | positive regulation of DNA replication |
| 70374 | 5.62E-02 | 1.83E-01 | positive regulation of ERK1 and ERK2 cascade |
| 51248 | 5.75E-02 | 1.85E-01 | negative regulation of protein metabolic process |
| 7398 | 5.76E-02 | 1.85E-01 | ectoderm development |
| 35148 | 5.78E-02 | 1.85E-01 | tube formation |
| 6690 | 5.78E-02 | 1.85E-01 | icosanoid metabolic process |
| 48593 | 5.78E-02 | 1.85E-01 | camera-type eye morphogenesis |
| 48638 | 5.78E-02 | 1.85E-01 | regulation of developmental growth |
| 16064 | 5.78E-02 | 1.85E-01 | immunoglobulin mediated immune response |
| 5976 | 5.79E-02 | 1.85E-01 | polysaccharide metabolic process |
| 6996 | 5.83E-02 | 1.85E-01 | organelle organization |
| 6643 | 5.85E-02 | 1.85E-01 | membrane lipid metabolic process |
| 42035 | 5.93E-02 | 1.85E-01 | regulation of cytokine biosynthetic process |
| 21537 | 5.93E-02 | 1.85E-01 | telencephalon development |
| 43280 | 5.96E-02 | 1.85E-01 | positive regulation of caspase activity |
| 10952 | 5.96E-02 | 1.85E-01 | positive regulation of peptidase activity |
| 34405 | 5.99E-02 | 1.85E-01 | response to fluid shear stress |
| 2065 | 5.99E-02 | 1.85E-01 | columnar/cuboidal epithelial cell differentiation |
| 51445 | 5.99E-02 | 1.85E-01 | regulation of meiotic cell cycle |
| 43300 | 5.99E-02 | 1.85E-01 | regulation of leukocyte degranulation |
| 2711 | 5.99E-02 | 1.85E-01 | positive regulation of T cell mediated immunity |
| 19369 | 5.99E-02 | 1.85E-01 | arachidonic acid metabolic process |
| 45123 | 5.99E-02 | 1.85E-01 | cellular extravasation |
| 45577 | 5.99E-02 | 1.85E-01 | regulation of B cell differentiation |
| 21602 | 5.99E-02 | 1.85E-01 | cranial nerve morphogenesis |
| 70875 | 5.99E-02 | 1.85E-01 | positive regulation of glycogen metabolic process |
| 71363 | 5.99E-02 | 1.85E-01 | cellular response to growth factor stimulus |
| 71634 | 5.99E-02 | 1.85E-01 | regulation of transforming growth factor-beta production |
| 31294 | 5.99E-02 | 1.85E-01 | lymphocyte costimulation |
| 31295 | 5.99E-02 | 1.85E-01 | T cell costimulation |
| 48070 | 5.99E-02 | 1.85E-01 | regulation of developmental pigmentation |
| 32006 | 5.99E-02 | 1.85E-01 | regulation of TOR signaling cascade |
| 2221 | 6.03E-02 | 1.85E-01 | pattern recognition receptor signaling pathway |
| 45445 | 6.03E-02 | 1.85E-01 | myoblast differentiation |
| 70997 | 6.03E-02 | 1.85E-01 | neuron death |
| 46638 | 6.03E-02 | 1.85E-01 | positive regulation of alpha-beta T cell differentiation |
| 32836 | 6.04E-02 | 1.85E-01 | glomerular basement membrane development |
| 32924 | 6.04E-02 | 1.85E-01 | activin receptor signaling pathway |
| 185 | 6.04E-02 | 1.85E-01 | activation of MAPKKK activity |
| 34067 | 6.04E-02 | 1.85E-01 | protein localization in Golgi apparatus |
| 42424 | 6.04E-02 | 1.85E-01 | catecholamine catabolic process |
| 50655 | 6.04E-02 | 1.85E-01 | dermatan sulfate proteoglycan metabolic process |
| 1514 | 6.04E-02 | 1.85E-01 | selenocysteine incorporation |
| 34313 | 6.04E-02 | 1.85E-01 | diol catabolic process |
| 1553 | 6.04E-02 | 1.85E-01 | luteinization |
| 34339 | 6.04E-02 | 1.85E-01 | regulation of transcription from RNA polymerase II promoter by nuclear hormone receptor |
| 34616 | 6.04E-02 | 1.85E-01 | response to laminar fluid shear stress |
| 1916 | 6.04E-02 | 1.85E-01 | positive regulation of T cell mediated cytotoxicity |
| 34694 | 6.04E-02 | 1.85E-01 | response to prostaglandin stimulus |
| 34695 | 6.04E-02 | 1.85E-01 | response to prostaglandin E stimulus |
| 1957 | 6.04E-02 | 1.85E-01 | intramembranous ossification |
| 42984 | 6.04E-02 | 1.85E-01 | regulation of amyloid precursor protein biosynthetic process |
| 51387 | 6.04E-02 | 1.85E-01 | negative regulation of nerve growth factor receptor signaling pathway |
| 43268 | 6.04E-02 | 1.85E-01 | positive regulation of potassium ion transport |
| 51481 | 6.04E-02 | 1.85E-01 | reduction of cytosolic calcium ion concentration |
| 2456 | 6.04E-02 | 1.85E-01 | T cell mediated immunity |
| 43587 | 6.04E-02 | 1.85E-01 | tongue morphogenesis |
| 43589 | 6.04E-02 | 1.85E-01 | skin morphogenesis |
| 2634 | 6.04E-02 | 1.85E-01 | regulation of germinal center formation |
| 2664 | 6.04E-02 | 1.85E-01 | regulation of T cell tolerance induction |
| 2666 | 6.04E-02 | 1.85E-01 | positive regulation of T cell tolerance induction |
| 10890 | 6.04E-02 | 1.85E-01 | positive regulation of sequestering of triglyceride |
| 60056 | 6.04E-02 | 1.85E-01 | mammary gland involution |
| 60073 | 6.04E-02 | 1.85E-01 | micturition |
| 2864 | 6.04E-02 | 1.85E-01 | regulation of acute inflammatory response to antigenic stimulus |
| 60291 | 6.04E-02 | 1.85E-01 | long-term synaptic potentiation |
| 3032 | 6.04E-02 | 1.85E-01 | detection of oxygen |
| 19441 | 6.04E-02 | 1.85E-01 | tryptophan catabolic process to kynurenine |
| 3157 | 6.04E-02 | 1.85E-01 | endocardium development |
| 3160 | 6.04E-02 | 1.85E-01 | endocardium morphogenesis |
| 19585 | 6.04E-02 | 1.85E-01 | glucuronate metabolic process |
| 19614 | 6.04E-02 | 1.85E-01 | catechol catabolic process |
| 60592 | 6.04E-02 | 1.85E-01 | mammary gland formation |
| 3337 | 6.04E-02 | 1.85E-01 | mesenchymal to epithelial transition involved in metanephros morphogenesis |
| 45019 | 6.04E-02 | 1.85E-01 | negative regulation of nitric oxide biosynthetic process |
| 45743 | 6.04E-02 | 1.85E-01 | positive regulation of fibroblast growth factor receptor signaling pathway |
| 70498 | 6.04E-02 | 1.85E-01 | interleukin-1-mediated signaling pathway |
| 21554 | 6.04E-02 | 1.85E-01 | optic nerve development |
| 30033 | 6.04E-02 | 1.85E-01 | microvillus assembly |
| 46500 | 6.04E-02 | 1.85E-01 | S-adenosylmethionine metabolic process |
| 6047 | 6.04E-02 | 1.85E-01 | UDP-N-acetylglucosamine metabolic process |
| 6063 | 6.04E-02 | 1.85E-01 | uronic acid metabolic process |
| 6451 | 6.04E-02 | 1.85E-01 | translational readthrough |
| 72028 | 6.04E-02 | 1.85E-01 | nephron morphogenesis |
| 72077 | 6.04E-02 | 1.85E-01 | renal vesicle morphogenesis |
| 72087 | 6.04E-02 | 1.85E-01 | renal vesicle development |
| 72088 | 6.04E-02 | 1.85E-01 | nephron epithelium morphogenesis |
| 6572 | 6.04E-02 | 1.85E-01 | tyrosine catabolic process |
| 72210 | 6.04E-02 | 1.85E-01 | metanephric nephron development |
| 72273 | 6.04E-02 | 1.85E-01 | metanephric nephron morphogenesis |
| 72283 | 6.04E-02 | 1.85E-01 | metanephric renal vesicle morphogenesis |
| 31338 | 6.04E-02 | 1.85E-01 | regulation of vesicle fusion |
| 6910 | 6.04E-02 | 1.85E-01 | phagocytosis, recognition |
| 31657 | 6.04E-02 | 1.85E-01 | regulation of cyclin-dependent protein kinase activity involved by G1/S |
| 31659 | 6.04E-02 | 1.85E-01 | positive regulation of cyclin-dependent protein kinase activity involved in G1/S |
| 7386 | 6.04E-02 | 1.85E-01 | compartment pattern formation |
| 15780 | 6.04E-02 | 1.85E-01 | nucleotide-sugar transport |
| 15781 | 6.04E-02 | 1.85E-01 | pyrimidine nucleotide-sugar transport |
| 48570 | 6.04E-02 | 1.85E-01 | notochord morphogenesis |
| 32288 | 6.04E-02 | 1.85E-01 | myelin assembly |
| 32528 | 6.04E-02 | 1.85E-01 | microvillus organization |
| 43242 | 6.11E-02 | 1.86E-01 | negative regulation of protein complex disassembly |
| 43648 | 6.11E-02 | 1.86E-01 | dicarboxylic acid metabolic process |
| 31668 | 6.14E-02 | 1.87E-01 | cellular response to extracellular stimulus |
| 51716 | 6.27E-02 | 1.91E-01 | cellular response to stimulus |
| 1890 | 6.29E-02 | 1.91E-01 | placenta development |
| 46364 | 6.36E-02 | 1.93E-01 | monosaccharide biosynthetic process |
| 32680 | 6.36E-02 | 1.93E-01 | regulation of tumor necrosis factor production |
| 9064 | 6.37E-02 | 1.93E-01 | glutamine family amino acid metabolic process |
| 42108 | 6.37E-02 | 1.93E-01 | positive regulation of cytokine biosynthetic process |
| 19724 | 6.37E-02 | 1.93E-01 | B cell mediated immunity |
| 45834 | 6.37E-02 | 1.93E-01 | positive regulation of lipid metabolic process |
| 50886 | 6.41E-02 | 1.94E-01 | endocrine process |
| 10332 | 6.41E-02 | 1.94E-01 | response to gamma radiation |
| 43271 | 6.41E-02 | 1.94E-01 | negative regulation of ion transport |
| 10743 | 6.41E-02 | 1.94E-01 | regulation of macrophage derived foam cell differentiation |
| 60512 | 6.41E-02 | 1.94E-01 | prostate gland morphogenesis |
| 45669 | 6.41E-02 | 1.94E-01 | positive regulation of osteoblast differentiation |
| 48546 | 6.41E-02 | 1.94E-01 | digestive tract morphogenesis |
| 45088 | 6.47E-02 | 1.95E-01 | regulation of innate immune response |
| 15672 | 6.49E-02 | 1.96E-01 | monovalent inorganic cation transport |
| 51223 | 6.51E-02 | 1.96E-01 | regulation of protein transport |
| 71496 | 6.56E-02 | 1.98E-01 | cellular response to external stimulus |
| 60562 | 6.68E-02 | 1.98E-01 | epithelial tube morphogenesis |
| 10675 | 6.79E-02 | 1.98E-01 | regulation of cellular carbohydrate metabolic process |
| 6006 | 6.86E-02 | 1.98E-01 | glucose metabolic process |
| 43244 | 6.99E-02 | 1.98E-01 | regulation of protein complex disassembly |
| 9896 | 7.00E-02 | 1.98E-01 | positive regulation of catabolic process |
| 19748 | 7.05E-02 | 1.98E-01 | secondary metabolic process |
| 33619 | 7.15E-02 | 1.98E-01 | membrane protein proteolysis |
| 9065 | 7.15E-02 | 1.98E-01 | glutamine family amino acid catabolic process |
| 43450 | 7.15E-02 | 1.98E-01 | alkene biosynthetic process |
| 19370 | 7.15E-02 | 1.98E-01 | leukotriene biosynthetic process |
| 45076 | 7.15E-02 | 1.98E-01 | regulation of interleukin-2 biosynthetic process |
| 48639 | 7.15E-02 | 1.98E-01 | positive regulation of developmental growth |
| 32370 | 7.15E-02 | 1.98E-01 | positive regulation of lipid transport |
| 50851 | 7.15E-02 | 1.98E-01 | antigen receptor-mediated signaling pathway |
| 7416 | 7.15E-02 | 1.98E-01 | synapse assembly |
| 32272 | 7.15E-02 | 1.98E-01 | negative regulation of protein polymerization |
| 48747 | 7.15E-02 | 1.98E-01 | muscle fiber development |
| 6754 | 7.22E-02 | 1.98E-01 | ATP biosynthetic process |
| 1654 | 7.23E-02 | 1.98E-01 | eye development |
| 44085 | 7.32E-02 | 1.98E-01 | cellular component biogenesis |
| 50830 | 7.34E-02 | 1.98E-01 | defense response to Gram-positive bacterium |
| 10883 | 7.34E-02 | 1.98E-01 | regulation of lipid storage |
| 3044 | 7.34E-02 | 1.98E-01 | regulation of systemic arterial blood pressure mediated by a chemical signal |
| 70304 | 7.34E-02 | 1.98E-01 | positive regulation of stress-activated protein kinase signaling cascade |
| 6090 | 7.34E-02 | 1.98E-01 | pyruvate metabolic process |
| 31330 | 7.34E-02 | 1.98E-01 | negative regulation of cellular catabolic process |
| 6921 | 7.34E-02 | 1.98E-01 | cellular component disassembly involved in apoptosis |
| 48741 | 7.34E-02 | 1.98E-01 | skeletal muscle fiber development |
| 8207 | 7.50E-02 | 1.98E-01 | C21-steroid hormone metabolic process |
| 32874 | 7.50E-02 | 1.98E-01 | positive regulation of stress-activated MAPK cascade |
| 32967 | 7.50E-02 | 1.98E-01 | positive regulation of collagen biosynthetic process |
| 9083 | 7.50E-02 | 1.98E-01 | branched chain family amino acid catabolic process |
| 42310 | 7.50E-02 | 1.98E-01 | vasoconstriction |
| 34377 | 7.50E-02 | 1.98E-01 | plasma lipoprotein particle assembly |
| 50930 | 7.50E-02 | 1.98E-01 | induction of positive chemotaxis |
| 2021 | 7.50E-02 | 1.98E-01 | response to dietary excess |
| 35019 | 7.50E-02 | 1.98E-01 | somatic stem cell maintenance |
| 51647 | 7.50E-02 | 1.98E-01 | nucleus localization |
| 10714 | 7.50E-02 | 1.98E-01 | positive regulation of collagen metabolic process |
| 43586 | 7.50E-02 | 1.98E-01 | tongue development |
| 10885 | 7.50E-02 | 1.98E-01 | regulation of cholesterol storage |
| 3081 | 7.50E-02 | 1.98E-01 | regulation of systemic arterial blood pressure by renin-angiotensin |
| 45648 | 7.50E-02 | 1.98E-01 | positive regulation of erythrocyte differentiation |
| 45737 | 7.50E-02 | 1.98E-01 | positive regulation of cyclin-dependent protein kinase activity |
| 45742 | 7.50E-02 | 1.98E-01 | positive regulation of epidermal growth factor receptor signaling pathway |
| 45885 | 7.50E-02 | 1.98E-01 | positive regulation of survival gene product expression |
| 45939 | 7.50E-02 | 1.98E-01 | negative regulation of steroid metabolic process |
| 46068 | 7.50E-02 | 1.98E-01 | cGMP metabolic process |
| 46503 | 7.50E-02 | 1.98E-01 | glycerolipid catabolic process |
| 21952 | 7.50E-02 | 1.98E-01 | central nervous system projection neuron axonogenesis |
| 30949 | 7.50E-02 | 1.98E-01 | positive regulation of vascular endothelial growth factor receptor signaling pathway |
| 14821 | 7.50E-02 | 1.98E-01 | phasic smooth muscle contraction |
| 65005 | 7.50E-02 | 1.98E-01 | protein-lipid complex assembly |
| 45216 | 7.51E-02 | 1.98E-01 | cell-cell junction organization |
| 30855 | 7.64E-02 | 1.98E-01 | epithelial cell differentiation |
| 31328 | 7.79E-02 | 1.98E-01 | positive regulation of cellular biosynthetic process |
| 43413 | 7.80E-02 | 1.98E-01 | macromolecule glycosylation |
| 70085 | 7.80E-02 | 1.98E-01 | glycosylation |
| 6486 | 7.80E-02 | 1.98E-01 | protein amino acid glycosylation |
| 9790 | 7.92E-02 | 1.98E-01 | embryonic development |
| 1649 | 7.99E-02 | 1.98E-01 | osteoblast differentiation |
| 6636 | 7.99E-02 | 1.98E-01 | unsaturated fatty acid biosynthetic process |
| 55085 | 8.01E-02 | 1.98E-01 | transmembrane transport |
| 17157 | 8.28E-02 | 1.98E-01 | regulation of exocytosis |
| 1658 | 8.34E-02 | 1.98E-01 | branching involved in ureteric bud morphogenesis |
| 19319 | 8.34E-02 | 1.98E-01 | hexose biosynthetic process |
| 30330 | 8.34E-02 | 1.98E-01 | DNA damage response, signal transduction by p53 class mediator |
| 48286 | 8.34E-02 | 1.98E-01 | lung alveolus development |
| 33135 | 8.36E-02 | 1.98E-01 | regulation of peptidyl-serine phosphorylation |
| 1659 | 8.36E-02 | 1.98E-01 | temperature homeostasis |
| 51004 | 8.36E-02 | 1.98E-01 | regulation of lipoprotein lipase activity |
| 43200 | 8.36E-02 | 1.98E-01 | response to amino acid stimulus |
| 35094 | 8.36E-02 | 1.98E-01 | response to nicotine |
| 2758 | 8.36E-02 | 1.98E-01 | innate immune response-activating signal transduction |
| 45744 | 8.36E-02 | 1.98E-01 | negative regulation of G-protein coupled receptor protein signaling pathway |
| 46849 | 8.36E-02 | 1.98E-01 | bone remodeling |
| 7628 | 8.36E-02 | 1.98E-01 | adult walking behavior |
| 48562 | 8.39E-02 | 1.98E-01 | embryonic organ morphogenesis |
| 43408 | 8.41E-02 | 1.98E-01 | regulation of MAPKKK cascade |
| 10552 | 8.46E-02 | 1.98E-01 | positive regulation of gene-specific transcription from RNA polymerase II promoter |
| 23 | 8.48E-02 | 1.98E-01 | maltose metabolic process |
| 32804 | 8.48E-02 | 1.98E-01 | negative regulation of low-density lipoprotein receptor catabolic process |
| 32805 | 8.48E-02 | 1.98E-01 | positive regulation of low-density lipoprotein receptor catabolic process |
| 90161 | 8.48E-02 | 1.98E-01 | Golgi ribbon formation |
| 32817 | 8.48E-02 | 1.98E-01 | regulation of natural killer cell proliferation |
| 32819 | 8.48E-02 | 1.98E-01 | positive regulation of natural killer cell proliferation |
| 90185 | 8.48E-02 | 1.98E-01 | negative regulation of kidney development |
| 90191 | 8.48E-02 | 1.98E-01 | negative regulation of branching involved in ureteric bud morphogenesis |
| 90194 | 8.48E-02 | 1.98E-01 | negative regulation of glomerulus development |
| 32878 | 8.48E-02 | 1.98E-01 | regulation of establishment or maintenance of cell polarity |
| 32911 | 8.48E-02 | 1.98E-01 | negative regulation of transforming growth factor-beta1 production |
| 90259 | 8.48E-02 | 1.98E-01 | regulation of retinal ganglion cell axon guidance |
| 90260 | 8.48E-02 | 1.98E-01 | negative regulation of retinal ganglion cell axon guidance |
| 90273 | 8.48E-02 | 1.98E-01 | regulation of somatostatin secretion |
| 90275 | 8.48E-02 | 1.98E-01 | negative regulation of somatostatin secretion |
| 90287 | 8.48E-02 | 1.98E-01 | regulation of cellular response to growth factor stimulus |
| 16560 | 8.48E-02 | 1.98E-01 | protein import into peroxisome matrix, docking |
| 90288 | 8.48E-02 | 1.98E-01 | negative regulation of cellular response to growth factor stimulus |
| 32972 | 8.48E-02 | 1.98E-01 | regulation of muscle filament sliding speed |
| 33007 | 8.48E-02 | 1.98E-01 | negative regulation of mast cell activation involved in immune response |
| 33091 | 8.48E-02 | 1.98E-01 | positive regulation of immature T cell proliferation |
| 33345 | 8.48E-02 | 1.98E-01 | asparagine catabolic process via L-aspartate |
| 33634 | 8.48E-02 | 1.98E-01 | positive regulation of cell-cell adhesion mediated by integrin |
| 9073 | 8.48E-02 | 1.98E-01 | aromatic amino acid family biosynthetic process |
| 9078 | 8.48E-02 | 1.98E-01 | pyruvate family amino acid metabolic process |
| 9095 | 8.48E-02 | 1.98E-01 | aromatic amino acid family biosynthetic process, prephenate pathway |
| 9098 | 8.48E-02 | 1.98E-01 | leucine biosynthetic process |
| 9115 | 8.48E-02 | 1.98E-01 | xanthine catabolic process |
| 33693 | 8.48E-02 | 1.98E-01 | neurofilament bundle assembly |
| 9236 | 8.48E-02 | 1.98E-01 | cobalamin biosynthetic process |
| 9372 | 8.48E-02 | 1.98E-01 | quorum sensing |
| 33986 | 8.48E-02 | 1.98E-01 | response to methanol |
| 34014 | 8.48E-02 | 1.98E-01 | response to triglyceride |
| 9438 | 8.48E-02 | 1.98E-01 | methylglyoxal metabolic process |
| 9449 | 8.48E-02 | 1.98E-01 | gamma-aminobutyric acid biosynthetic process |
| 42249 | 8.48E-02 | 1.98E-01 | establishment of planar polarity of embryonic epithelium |
| 42262 | 8.48E-02 | 1.98E-01 | DNA protection |
| 42264 | 8.48E-02 | 1.98E-01 | peptidyl-aspartic acid hydroxylation |
| 34118 | 8.48E-02 | 1.98E-01 | regulation of erythrocyte aggregation |
| 34120 | 8.48E-02 | 1.98E-01 | positive regulation of erythrocyte aggregation |
| 42413 | 8.48E-02 | 1.98E-01 | carnitine catabolic process |
| 50652 | 8.48E-02 | 1.98E-01 | dermatan sulfate proteoglycan biosynthetic process, polysaccharide chain biosynthetic process |
| 34275 | 8.48E-02 | 1.98E-01 | kynurenic acid metabolic process |
| 34276 | 8.48E-02 | 1.98E-01 | kynurenic acid biosynthetic process |
| 50668 | 8.48E-02 | 1.98E-01 | positive regulation of homocysteine metabolic process |
| 50674 | 8.48E-02 | 1.98E-01 | urothelial cell proliferation |
| 50675 | 8.48E-02 | 1.98E-01 | regulation of urothelial cell proliferation |
| 50677 | 8.48E-02 | 1.98E-01 | positive regulation of urothelial cell proliferation |
| 9720 | 8.48E-02 | 1.98E-01 | detection of hormone stimulus |
| 9726 | 8.48E-02 | 1.98E-01 | detection of endogenous stimulus |
| 50747 | 8.48E-02 | 1.98E-01 | positive regulation of lipoprotein metabolic process |
| 34373 | 8.48E-02 | 1.98E-01 | intermediate-density lipoprotein particle remodeling |
| 50783 | 8.48E-02 | 1.98E-01 | cocaine metabolic process |
| 50812 | 8.48E-02 | 1.98E-01 | regulation of acyl-CoA biosynthetic process |
| 34436 | 8.48E-02 | 1.98E-01 | glycoprotein transport |
| 34454 | 8.48E-02 | 1.98E-01 | microtubule anchoring at centrosome |
| 34465 | 8.48E-02 | 1.98E-01 | response to carbon monoxide |
| 34499 | 8.48E-02 | 1.98E-01 | late endosome to Golgi transport |
| 50893 | 8.48E-02 | 1.98E-01 | sensory processing |
| 42701 | 8.48E-02 | 1.98E-01 | progesterone secretion |
| 42706 | 8.48E-02 | 1.98E-01 | eye photoreceptor cell fate commitment |
| 34516 | 8.48E-02 | 1.98E-01 | response to vitamin B6 |
| 18146 | 8.48E-02 | 1.98E-01 | keratan sulfate biosynthetic process |
| 1771 | 8.48E-02 | 1.98E-01 | formation of immunological synapse |
| 50924 | 8.48E-02 | 1.98E-01 | positive regulation of negative chemotaxis |
| 50929 | 8.48E-02 | 1.98E-01 | induction of negative chemotaxis |
| 1796 | 8.48E-02 | 1.98E-01 | regulation of type IIa hypersensitivity |
| 1798 | 8.48E-02 | 1.98E-01 | positive regulation of type IIa hypersensitivity |
| 18197 | 8.48E-02 | 1.98E-01 | peptidyl-aspartic acid modification |
| 10037 | 8.48E-02 | 1.98E-01 | response to carbon dioxide |
| 34638 | 8.48E-02 | 1.98E-01 | phosphatidylcholine catabolic process |
| 42839 | 8.48E-02 | 1.98E-01 | D-glucuronate metabolic process |
| 1879 | 8.48E-02 | 1.98E-01 | detection of yeast |
| 42840 | 8.48E-02 | 1.98E-01 | D-glucuronate catabolic process |
| 18272 | 8.48E-02 | 1.98E-01 | protein-pyridoxal-5-phosphate linkage via peptidyl-N6-pyridoxal phosphate-L-lysine |
| 42851 | 8.48E-02 | 1.98E-01 | L-alanine metabolic process |
| 34699 | 8.48E-02 | 1.98E-01 | response to luteinizing hormone stimulus |
| 51089 | 8.48E-02 | 1.98E-01 | constitutive protein ectodomain proteolysis |
| 51126 | 8.48E-02 | 1.98E-01 | negative regulation of actin nucleation |
| 1998 | 8.48E-02 | 1.98E-01 | angiotensin mediated vasoconstriction involved in regulation of systemic arterial blood pressure |
| 2001 | 8.48E-02 | 1.98E-01 | renin secretion into blood stream |
| 51160 | 8.48E-02 | 1.98E-01 | L-xylitol catabolic process |
| 51164 | 8.48E-02 | 1.98E-01 | L-xylitol metabolic process |
| 43000 | 8.48E-02 | 1.98E-01 | Golgi to plasma membrane CFTR protein transport |
| 2041 | 8.48E-02 | 1.98E-01 | intussusceptive angiogenesis |
| 10260 | 8.48E-02 | 1.98E-01 | organ senescence |
| 43096 | 8.48E-02 | 1.98E-01 | purine base salvage |
| 43132 | 8.48E-02 | 1.98E-01 | NAD transport |
| 43181 | 8.48E-02 | 1.98E-01 | vacuolar sequestering |
| 43301 | 8.48E-02 | 1.98E-01 | negative regulation of leukocyte degranulation |
| 43305 | 8.48E-02 | 1.98E-01 | negative regulation of mast cell degranulation |
| 51543 | 8.48E-02 | 1.98E-01 | regulation of elastin biosynthetic process |
| 51545 | 8.48E-02 | 1.98E-01 | negative regulation of elastin biosynthetic process |
| 2396 | 8.48E-02 | 1.98E-01 | MHC protein complex assembly |
| 2397 | 8.48E-02 | 1.98E-01 | MHC class I protein complex assembly |
| 10593 | 8.48E-02 | 1.98E-01 | negative regulation of lamellipodium assembly |
| 43380 | 8.48E-02 | 1.98E-01 | regulation of memory T cell differentiation |
| 51581 | 8.48E-02 | 1.98E-01 | negative regulation of neurotransmitter uptake |
| 2430 | 8.48E-02 | 1.98E-01 | complement receptor mediated signaling pathway |
| 51585 | 8.48E-02 | 1.98E-01 | negative regulation of dopamine uptake |
| 2457 | 8.48E-02 | 1.98E-01 | T cell antigen processing and presentation |
| 43418 | 8.48E-02 | 1.98E-01 | homocysteine catabolic process |
| 51611 | 8.48E-02 | 1.98E-01 | regulation of serotonin uptake |
| 43420 | 8.48E-02 | 1.98E-01 | anthranilate metabolic process |
| 51612 | 8.48E-02 | 1.98E-01 | negative regulation of serotonin uptake |
| 51621 | 8.48E-02 | 1.98E-01 | regulation of norepinephrine uptake |
| 51622 | 8.48E-02 | 1.98E-01 | negative regulation of norepinephrine uptake |
| 2501 | 8.48E-02 | 1.98E-01 | peptide antigen assembly with MHC protein complex |
| 10693 | 8.48E-02 | 1.98E-01 | negative regulation of alkaline phosphatase activity |
| 2502 | 8.48E-02 | 1.98E-01 | peptide antigen assembly with MHC class I protein complex |
| 2534 | 8.48E-02 | 1.98E-01 | cytokine production involved in inflammatory response |
| 43503 | 8.48E-02 | 1.98E-01 | skeletal muscle fiber adaptation |
| 10749 | 8.48E-02 | 1.98E-01 | regulation of nitric oxide mediated signal transduction |
| 10751 | 8.48E-02 | 1.98E-01 | negative regulation of nitric oxide mediated signal transduction |
| 10752 | 8.48E-02 | 1.98E-01 | regulation of cGMP-mediated signaling |
| 10754 | 8.48E-02 | 1.98E-01 | negative regulation of cGMP-mediated signaling |
| 2575 | 8.48E-02 | 1.98E-01 | basophil chemotaxis |
| 2578 | 8.48E-02 | 1.98E-01 | negative regulation of antigen processing and presentation |
| 2580 | 8.48E-02 | 1.98E-01 | regulation of antigen processing and presentation of peptide or polysaccharide antigen via MHC class II |
| 2581 | 8.48E-02 | 1.98E-01 | negative regulation of antigen processing and presentation of peptide or polysaccharide antigen via MHC class II |
| 35356 | 8.48E-02 | 1.98E-01 | cellular triglyceride homeostasis |
| 2605 | 8.48E-02 | 1.98E-01 | negative regulation of dendritic cell antigen processing and presentation |
| 10801 | 8.48E-02 | 1.98E-01 | negative regulation of peptidyl-threonine phosphorylation |
| 35406 | 8.48E-02 | 1.98E-01 | histone-tyrosine phosphorylation |
| 35407 | 8.48E-02 | 1.98E-01 | histone H3-T11 phosphorylation |
| 35409 | 8.48E-02 | 1.98E-01 | histone H3-Y41 phosphorylation |
| 51794 | 8.48E-02 | 1.98E-01 | regulation of catagen |
| 51795 | 8.48E-02 | 1.98E-01 | positive regulation of catagen |
| 2649 | 8.48E-02 | 1.98E-01 | regulation of tolerance induction to self antigen |
| 2651 | 8.48E-02 | 1.98E-01 | positive regulation of tolerance induction to self antigen |
| 60019 | 8.48E-02 | 1.98E-01 | radial glial cell differentiation |
| 19063 | 8.48E-02 | 1.98E-01 | virion penetration into host cell |
| 19064 | 8.48E-02 | 1.98E-01 | viral envelope fusion with host membrane |
| 10878 | 8.48E-02 | 1.98E-01 | cholesterol storage |
| 60034 | 8.48E-02 | 1.98E-01 | notochord cell differentiation |
| 60035 | 8.48E-02 | 1.98E-01 | notochord cell development |
| 43652 | 8.48E-02 | 1.98E-01 | engulfment of apoptotic cell |
| 2693 | 8.48E-02 | 1.98E-01 | positive regulation of cellular extravasation |
| 10899 | 8.48E-02 | 1.98E-01 | regulation of phosphatidylcholine catabolic process |
| 10900 | 8.48E-02 | 1.98E-01 | negative regulation of phosphatidylcholine catabolic process |
| 35490 | 8.48E-02 | 1.98E-01 | regulation of leukotriene production involved in inflammatory response |
| 35491 | 8.48E-02 | 1.98E-01 | positive regulation of leukotriene production involved in inflammatory response |
| 10920 | 8.48E-02 | 1.98E-01 | negative regulation of inositol phosphate biosynthetic process |
| 60074 | 8.48E-02 | 1.98E-01 | synapse maturation |
| 10924 | 8.48E-02 | 1.98E-01 | regulation of inositol-polyphosphate 5-phosphatase activity |
| 10925 | 8.48E-02 | 1.98E-01 | positive regulation of inositol-polyphosphate 5-phosphatase activity |
| 60082 | 8.48E-02 | 1.98E-01 | eye blink reflex |
| 10932 | 8.48E-02 | 1.98E-01 | regulation of macrophage tolerance induction |
| 10933 | 8.48E-02 | 1.98E-01 | positive regulation of macrophage tolerance induction |
| 10936 | 8.48E-02 | 1.98E-01 | negative regulation of macrophage cytokine production |
| 51913 | 8.48E-02 | 1.98E-01 | regulation of synaptic plasticity by chemical substance |
| 51914 | 8.48E-02 | 1.98E-01 | positive regulation of synaptic plasticity by chemical substance |
| 51915 | 8.48E-02 | 1.98E-01 | induction of synaptic plasticity by chemical substance |
| 35555 | 8.48E-02 | 1.98E-01 | initiation of Roundabout signal transduction |
| 51939 | 8.48E-02 | 1.98E-01 | gamma-aminobutyric acid import |
| 51945 | 8.48E-02 | 1.98E-01 | negative regulation of catecholamine uptake involved in synaptic transmission |
| 2830 | 8.48E-02 | 1.98E-01 | positive regulation of T-helper 2 type immune response |
| 60209 | 8.48E-02 | 1.98E-01 | estrus |
| 60211 | 8.48E-02 | 1.98E-01 | regulation of nuclear-transcribed mRNA poly(A) tail shortening |
| 60213 | 8.48E-02 | 1.98E-01 | positive regulation of nuclear-transcribed mRNA poly(A) tail shortening |
| 60215 | 8.48E-02 | 1.98E-01 | primitive hemopoiesis |
| 60220 | 8.48E-02 | 1.98E-01 | camera-type eye photoreceptor cell fate commitment |
| 19265 | 8.48E-02 | 1.98E-01 | glycine biosynthetic process, by transamination of glyoxylate |
| 2888 | 8.48E-02 | 1.98E-01 | positive regulation of myeloid leukocyte mediated immunity |
| 2892 | 8.48E-02 | 1.98E-01 | regulation of type II hypersensitivity |
| 2894 | 8.48E-02 | 1.98E-01 | positive regulation of type II hypersensitivity |
| 60242 | 8.48E-02 | 1.98E-01 | contact inhibition |
| 52097 | 8.48E-02 | 1.98E-01 | interspecies quorum sensing |
| 52106 | 8.48E-02 | 1.98E-01 | quorum sensing involved in interaction with host |
| 19343 | 8.48E-02 | 1.98E-01 | cysteine biosynthetic process via cystathionine |
| 60305 | 8.48E-02 | 1.98E-01 | regulation of cell diameter |
| 60318 | 8.48E-02 | 1.98E-01 | definitive erythrocyte differentiation |
| 60319 | 8.48E-02 | 1.98E-01 | primitive erythrocyte differentiation |
| 19375 | 8.48E-02 | 1.98E-01 | galactolipid biosynthetic process |
| 19376 | 8.48E-02 | 1.98E-01 | galactolipid catabolic process |
| 19388 | 8.48E-02 | 1.98E-01 | galactose catabolic process |
| 60353 | 8.48E-02 | 1.98E-01 | regulation of cell adhesion molecule production |
| 60355 | 8.48E-02 | 1.98E-01 | positive regulation of cell adhesion molecule production |
| 60356 | 8.48E-02 | 1.98E-01 | leucine import |
| 19407 | 8.48E-02 | 1.98E-01 | hexitol catabolic process |
| 60373 | 8.48E-02 | 1.98E-01 | regulation of ventricular cardiomyocyte membrane depolarization |
| 60436 | 8.48E-02 | 1.98E-01 | bronchiole morphogenesis |
| 60440 | 8.48E-02 | 1.98E-01 | trachea formation |
| 19519 | 8.48E-02 | 1.98E-01 | pentitol metabolic process |
| 19527 | 8.48E-02 | 1.98E-01 | pentitol catabolic process |
| 60502 | 8.48E-02 | 1.98E-01 | epithelial cell proliferation involved in lung morphogenesis |
| 60503 | 8.48E-02 | 1.98E-01 | bud dilation involved in lung branching |
| 3176 | 8.48E-02 | 1.98E-01 | aortic valve development |
| 3180 | 8.48E-02 | 1.98E-01 | aortic valve morphogenesis |
| 60594 | 8.48E-02 | 1.98E-01 | mammary gland specification |
| 60595 | 8.48E-02 | 1.98E-01 | fibroblast growth factor receptor signaling pathway involved in mammary gland specification |
| 60615 | 8.48E-02 | 1.98E-01 | mammary gland bud formation |
| 60648 | 8.48E-02 | 1.98E-01 | mammary gland bud morphogenesis |
| 60667 | 8.48E-02 | 1.98E-01 | branch elongation involved in salivary gland morphogenesis |
| 60676 | 8.48E-02 | 1.98E-01 | ureteric bud formation |
| 60681 | 8.48E-02 | 1.98E-01 | branch elongation involved in ureteric bud branching |
| 60683 | 8.48E-02 | 1.98E-01 | regulation of branching involved in salivary gland morphogenesis by epithelial-mesenchymal signaling |
| 60730 | 8.48E-02 | 1.98E-01 | regulation of intestinal epithelial structure maintenance |
| 60731 | 8.48E-02 | 1.98E-01 | positive regulation of intestinal epithelial structure maintenance |
| 60739 | 8.48E-02 | 1.98E-01 | mesenchymal-epithelial cell signaling involved in prostate gland development |
| 3406 | 8.48E-02 | 1.98E-01 | retinal pigment epithelium development |
| 60904 | 8.48E-02 | 1.98E-01 | regulation of protein folding in endoplasmic reticulum |
| 60915 | 8.48E-02 | 1.98E-01 | mesenchymal cell differentiation involved in lung development |
| 61013 | 8.48E-02 | 1.98E-01 | regulation of mRNA catabolic process |
| 61014 | 8.48E-02 | 1.98E-01 | positive regulation of mRNA catabolic process |
| 61029 | 8.48E-02 | 1.98E-01 | eyelid development in camera-type eye |
| 61044 | 8.48E-02 | 1.98E-01 | negative regulation of vascular wound healing |
| 61045 | 8.48E-02 | 1.98E-01 | negative regulation of wound healing |
| 61155 | 8.48E-02 | 1.98E-01 | pulmonary artery endothelial tube morphogenesis |
| 61156 | 8.48E-02 | 1.98E-01 | pulmonary artery morphogenesis |
| 45065 | 8.48E-02 | 1.98E-01 | cytotoxic T cell differentiation |
| 45112 | 8.48E-02 | 1.98E-01 | integrin biosynthetic process |
| 45204 | 8.48E-02 | 1.98E-01 | MAPK export from nucleus |
| 45208 | 8.48E-02 | 1.98E-01 | MAPK phosphatase export from nucleus |
| 45209 | 8.48E-02 | 1.98E-01 | MAPK phosphatase export from nucleus, leptomycin B sensitive |
| 45210 | 8.48E-02 | 1.98E-01 | FasL biosynthetic process |
| 45338 | 8.48E-02 | 1.98E-01 | farnesyl diphosphate metabolic process |
| 45366 | 8.48E-02 | 1.98E-01 | regulation of interleukin-13 biosynthetic process |
| 45368 | 8.48E-02 | 1.98E-01 | positive regulation of interleukin-13 biosynthetic process |
| 70098 | 8.48E-02 | 1.98E-01 | chemokine-mediated signaling pathway |
| 70103 | 8.48E-02 | 1.98E-01 | regulation of interleukin-6-mediated signaling pathway |
| 70104 | 8.48E-02 | 1.98E-01 | negative regulation of interleukin-6-mediated signaling pathway |
| 70106 | 8.48E-02 | 1.98E-01 | interleukin-27-mediated signaling pathway |
| 70164 | 8.48E-02 | 1.98E-01 | negative regulation of adiponectin secretion |
| 45659 | 8.48E-02 | 1.98E-01 | negative regulation of neutrophil differentiation |
| 70257 | 8.48E-02 | 1.98E-01 | positive regulation of mucus secretion |
| 70278 | 8.48E-02 | 1.98E-01 | extracellular matrix constituent secretion |
| 45724 | 8.48E-02 | 1.98E-01 | positive regulation of flagellum assembly |
| 70316 | 8.48E-02 | 1.98E-01 | regulation of G0 to G1 transition |
| 70318 | 8.48E-02 | 1.98E-01 | positive regulation of G0 to G1 transition |
| 45764 | 8.48E-02 | 1.98E-01 | positive regulation of cellular amino acid metabolic process |
| 45794 | 8.48E-02 | 1.98E-01 | negative regulation of cell volume |
| 45795 | 8.48E-02 | 1.98E-01 | positive regulation of cell volume |
| 70407 | 8.48E-02 | 1.98E-01 | oxidation-dependent protein catabolic process |
| 45837 | 8.48E-02 | 1.98E-01 | negative regulation of membrane potential |
| 45870 | 8.48E-02 | 1.98E-01 | positive regulation of retroviral genome replication |
| 45914 | 8.48E-02 | 1.98E-01 | negative regulation of catecholamine metabolic process |
| 70495 | 8.48E-02 | 1.98E-01 | negative regulation of thrombin receptor signaling pathway |
| 70494 | 8.48E-02 | 1.98E-01 | regulation of thrombin receptor signaling pathway |
| 45963 | 8.48E-02 | 1.98E-01 | negative regulation of dopamine metabolic process |
| 70560 | 8.48E-02 | 1.98E-01 | protein secretion by platelet |
| 46032 | 8.48E-02 | 1.98E-01 | ADP catabolic process |
| 70627 | 8.48E-02 | 1.98E-01 | ferrous iron import |
| 46083 | 8.48E-02 | 1.98E-01 | adenine metabolic process |
| 46084 | 8.48E-02 | 1.98E-01 | adenine biosynthetic process |
| 46086 | 8.48E-02 | 1.98E-01 | adenosine biosynthetic process |
| 70671 | 8.48E-02 | 1.98E-01 | response to interleukin-12 |
| 70673 | 8.48E-02 | 1.98E-01 | response to interleukin-18 |
| 46104 | 8.48E-02 | 1.98E-01 | thymidine metabolic process |
| 46127 | 8.48E-02 | 1.98E-01 | pyrimidine deoxyribonucleoside catabolic process |
| 70715 | 8.48E-02 | 1.98E-01 | sodium-dependent organic cation transport |
| 21572 | 8.48E-02 | 1.98E-01 | rhombomere 6 development |
| 21594 | 8.48E-02 | 1.98E-01 | rhombomere formation |
| 21660 | 8.48E-02 | 1.98E-01 | rhombomere 3 formation |
| 21664 | 8.48E-02 | 1.98E-01 | rhombomere 5 morphogenesis |
| 21666 | 8.48E-02 | 1.98E-01 | rhombomere 5 formation |
| 46314 | 8.48E-02 | 1.98E-01 | phosphocreatine biosynthetic process |
| 21768 | 8.48E-02 | 1.98E-01 | nucleus accumbens development |
| 21784 | 8.48E-02 | 1.98E-01 | postganglionic parasympathetic nervous system development |
| 46370 | 8.48E-02 | 1.98E-01 | fructose biosynthetic process |
| 30007 | 8.48E-02 | 1.98E-01 | cellular potassium ion homeostasis |
| 30026 | 8.48E-02 | 1.98E-01 | cellular manganese ion homeostasis |
| 46417 | 8.48E-02 | 1.98E-01 | chorismate metabolic process |
| 46448 | 8.48E-02 | 1.98E-01 | tropane alkaloid metabolic process |
| 30092 | 8.48E-02 | 1.98E-01 | regulation of flagellum assembly |
| 21957 | 8.48E-02 | 1.98E-01 | corticospinal tract morphogenesis |
| 21966 | 8.48E-02 | 1.98E-01 | corticospinal neuron axon guidance |
| 21972 | 8.48E-02 | 1.98E-01 | corticospinal neuron axon guidance through the spinal cord |
| 46552 | 8.48E-02 | 1.98E-01 | photoreceptor cell fate commitment |
| 46654 | 8.48E-02 | 1.98E-01 | tetrahydrofolate biosynthetic process |
| 46687 | 8.48E-02 | 1.98E-01 | response to chromate |
| 46707 | 8.48E-02 | 1.98E-01 | IDP metabolic process |
| 46709 | 8.48E-02 | 1.98E-01 | IDP catabolic process |
| 46724 | 8.48E-02 | 1.98E-01 | oxalic acid secretion |
| 71336 | 8.48E-02 | 1.98E-01 | regulation of hair follicle cell proliferation |
| 71338 | 8.48E-02 | 1.98E-01 | positive regulation of hair follicle cell proliferation |
| 71361 | 8.48E-02 | 1.98E-01 | cellular response to ethanol |
| 46814 | 8.48E-02 | 1.98E-01 | virion attachment, binding of host cell surface coreceptor |
| 55071 | 8.48E-02 | 1.98E-01 | manganese ion homeostasis |
| 30505 | 8.48E-02 | 1.98E-01 | inorganic diphosphate transport |
| 5985 | 8.48E-02 | 1.98E-01 | sucrose metabolic process |
| 5997 | 8.48E-02 | 1.98E-01 | xylulose metabolic process |
| 6060 | 8.48E-02 | 1.98E-01 | sorbitol metabolic process |
| 6062 | 8.48E-02 | 1.98E-01 | sorbitol catabolic process |
| 6064 | 8.48E-02 | 1.98E-01 | glucuronate catabolic process |
| 71671 | 8.48E-02 | 1.98E-01 | regulation of smooth muscle cell chemotaxis |
| 71672 | 8.48E-02 | 1.98E-01 | negative regulation of smooth muscle cell chemotaxis |
| 71676 | 8.48E-02 | 1.98E-01 | negative regulation of mononuclear cell migration |
| 6168 | 8.48E-02 | 1.98E-01 | adenine salvage |
| 6214 | 8.48E-02 | 1.98E-01 | thymidine catabolic process |
| 30845 | 8.48E-02 | 1.98E-01 | inhibition of phospholipase C activity involved in G-protein coupled receptor signaling pathway |
| 30887 | 8.48E-02 | 1.98E-01 | positive regulation of myeloid dendritic cell activation |
| 6463 | 8.48E-02 | 1.98E-01 | steroid hormone receptor complex assembly |
| 6522 | 8.48E-02 | 1.98E-01 | alanine metabolic process |
| 6530 | 8.48E-02 | 1.98E-01 | asparagine catabolic process |
| 6535 | 8.48E-02 | 1.98E-01 | cysteine biosynthetic process from serine |
| 6550 | 8.48E-02 | 1.98E-01 | isoleucine catabolic process |
| 6571 | 8.48E-02 | 1.98E-01 | tyrosine biosynthetic process |
| 6579 | 8.48E-02 | 1.98E-01 | betaine catabolic process |
| 6603 | 8.48E-02 | 1.98E-01 | phosphocreatine metabolic process |
| 14807 | 8.48E-02 | 1.98E-01 | regulation of somitogenesis |
| 31223 | 8.48E-02 | 1.98E-01 | auditory behavior |
| 31247 | 8.48E-02 | 1.98E-01 | actin rod assembly |
| 14873 | 8.48E-02 | 1.98E-01 | response to muscle activity involved in regulation of muscle adaptation |
| 6682 | 8.48E-02 | 1.98E-01 | galactosylceramide biosynthetic process |
| 6683 | 8.48E-02 | 1.98E-01 | galactosylceramide catabolic process |
| 31268 | 8.48E-02 | 1.98E-01 | pseudopodium organization |
| 14895 | 8.48E-02 | 1.98E-01 | smooth muscle hypertrophy |
| 6711 | 8.48E-02 | 1.98E-01 | estrogen catabolic process |
| 6713 | 8.48E-02 | 1.98E-01 | glucocorticoid catabolic process |
| 6741 | 8.48E-02 | 1.98E-01 | NADP biosynthetic process |
| 31337 | 8.48E-02 | 1.98E-01 | positive regulation of sulfur amino acid metabolic process |
| 6797 | 8.48E-02 | 1.98E-01 | polyphosphate metabolic process |
| 6848 | 8.48E-02 | 1.98E-01 | pyruvate transport |
| 6867 | 8.48E-02 | 1.98E-01 | asparagine transport |
| 6868 | 8.48E-02 | 1.98E-01 | glutamine transport |
| 48074 | 8.48E-02 | 1.98E-01 | negative regulation of eye pigmentation |
| 48086 | 8.48E-02 | 1.98E-01 | negative regulation of developmental pigmentation |
| 7192 | 8.48E-02 | 1.98E-01 | activation of adenylate cyclase activity by serotonin receptor signaling pathway |
| 7208 | 8.48E-02 | 1.98E-01 | activation of phospholipase C activity by serotonin receptor signaling pathway |
| 48176 | 8.48E-02 | 1.98E-01 | regulation of hepatocyte growth factor biosynthetic process |
| 48178 | 8.48E-02 | 1.98E-01 | negative regulation of hepatocyte growth factor biosynthetic process |
| 48320 | 8.48E-02 | 1.98E-01 | axial mesoderm formation |
| 31944 | 8.48E-02 | 1.98E-01 | negative regulation of glucocorticoid metabolic process |
| 31946 | 8.48E-02 | 1.98E-01 | regulation of glucocorticoid biosynthetic process |
| 31947 | 8.48E-02 | 1.98E-01 | negative regulation of glucocorticoid biosynthetic process |
| 31959 | 8.48E-02 | 1.98E-01 | mineralocorticoid receptor signaling pathway |
| 48390 | 8.48E-02 | 1.98E-01 | intermediate mesoderm morphogenesis |
| 48391 | 8.48E-02 | 1.98E-01 | intermediate mesoderm formation |
| 48392 | 8.48E-02 | 1.98E-01 | intermediate mesodermal cell differentiation |
| 15675 | 8.48E-02 | 1.98E-01 | nickel ion transport |
| 15676 | 8.48E-02 | 1.98E-01 | vanadium ion transport |
| 15684 | 8.48E-02 | 1.98E-01 | ferrous iron transport |
| 7495 | 8.48E-02 | 1.98E-01 | visceral mesoderm-endoderm interaction involved in midgut development |
| 15692 | 8.48E-02 | 1.98E-01 | lead ion transport |
| 15728 | 8.48E-02 | 1.98E-01 | mevalonate transport |
| 15739 | 8.48E-02 | 1.98E-01 | sialic acid transport |
| 15785 | 8.48E-02 | 1.98E-01 | UDP-galactose transport |
| 15803 | 8.48E-02 | 1.98E-01 | branched-chain aliphatic amino acid transport |
| 15817 | 8.48E-02 | 1.98E-01 | histidine transport |
| 15820 | 8.48E-02 | 1.98E-01 | leucine transport |
| 15827 | 8.48E-02 | 1.98E-01 | tryptophan transport |
| 48683 | 8.48E-02 | 1.98E-01 | regulation of collateral sprouting of intact axon in response to injury |
| 48685 | 8.48E-02 | 1.98E-01 | negative regulation of collateral sprouting of intact axon in response to injury |
| 48818 | 8.48E-02 | 1.98E-01 | positive regulation of hair follicle maturation |
| 48819 | 8.48E-02 | 1.98E-01 | regulation of hair follicle maturation |
| 32460 | 8.48E-02 | 1.98E-01 | negative regulation of protein oligomerization |
| 32461 | 8.48E-02 | 1.98E-01 | positive regulation of protein oligomerization |
| 32462 | 8.48E-02 | 1.98E-01 | regulation of protein homooligomerization |
| 32463 | 8.48E-02 | 1.98E-01 | negative regulation of protein homooligomerization |
| 32468 | 8.48E-02 | 1.98E-01 | Golgi calcium ion homeostasis |
| 32472 | 8.48E-02 | 1.98E-01 | Golgi calcium ion transport |
| 48874 | 8.48E-02 | 1.98E-01 | homeostasis of number of cells in a free-living population |
| 32530 | 8.48E-02 | 1.98E-01 | regulation of microvillus organization |
| 32534 | 8.48E-02 | 1.98E-01 | regulation of microvillus assembly |
| 8049 | 8.48E-02 | 1.98E-01 | male courtship behavior |
| 32646 | 8.48E-02 | 1.98E-01 | regulation of hepatocyte growth factor production |
| 90024 | 8.48E-02 | 1.98E-01 | negative regulation of neutrophil chemotaxis |
| 32696 | 8.48E-02 | 1.98E-01 | negative regulation of interleukin-13 production |
| 32714 | 8.48E-02 | 1.98E-01 | negative regulation of interleukin-5 production |
| 32736 | 8.48E-02 | 1.98E-01 | positive regulation of interleukin-13 production |
| 32754 | 8.48E-02 | 1.98E-01 | positive regulation of interleukin-5 production |
| 48839 | 8.52E-02 | 1.98E-01 | inner ear development |
| 33015 | 8.56E-02 | 1.98E-01 | tetrapyrrole catabolic process |
| 8634 | 8.56E-02 | 1.98E-01 | negative regulation of survival gene product expression |
| 9068 | 8.56E-02 | 1.98E-01 | aspartate family amino acid catabolic process |
| 9437 | 8.56E-02 | 1.98E-01 | carnitine metabolic process |
| 9649 | 8.56E-02 | 1.98E-01 | entrainment of circadian clock |
| 42448 | 8.56E-02 | 1.98E-01 | progesterone metabolic process |
| 34332 | 8.56E-02 | 1.98E-01 | adherens junction organization |
| 34379 | 8.56E-02 | 1.98E-01 | very-low-density lipoprotein particle assembly |
| 50901 | 8.56E-02 | 1.98E-01 | leukocyte tethering or rolling |
| 50918 | 8.56E-02 | 1.98E-01 | positive chemotaxis |
| 50919 | 8.56E-02 | 1.98E-01 | negative chemotaxis |
| 1867 | 8.56E-02 | 1.98E-01 | complement activation, lectin pathway |
| 2067 | 8.56E-02 | 1.98E-01 | glandular epithelial cell differentiation |
| 51386 | 8.56E-02 | 1.98E-01 | regulation of nerve growth factor receptor signaling pathway |
| 51580 | 8.56E-02 | 1.98E-01 | regulation of neurotransmitter uptake |
| 51584 | 8.56E-02 | 1.98E-01 | regulation of dopamine uptake |
| 2467 | 8.56E-02 | 1.98E-01 | germinal center formation |
| 19062 | 8.56E-02 | 1.98E-01 | virion attachment to host cell surface receptor |
| 10886 | 8.56E-02 | 1.98E-01 | positive regulation of cholesterol storage |
| 60038 | 8.56E-02 | 1.98E-01 | cardiac muscle cell proliferation |
| 51940 | 8.56E-02 | 1.98E-01 | regulation of catecholamine uptake involved in synaptic transmission |
| 60351 | 8.56E-02 | 1.98E-01 | cartilage development involved in endochondral bone morphogenesis |
| 60665 | 8.56E-02 | 1.98E-01 | regulation of branching involved in salivary gland morphogenesis by mesenchymal-epithelial signaling |
| 3338 | 8.56E-02 | 1.98E-01 | metanephros morphogenesis |
| 60685 | 8.56E-02 | 1.98E-01 | regulation of prostatic bud formation |
| 45060 | 8.56E-02 | 1.98E-01 | negative thymic T cell selection |
| 45579 | 8.56E-02 | 1.98E-01 | positive regulation of B cell differentiation |
| 45628 | 8.56E-02 | 1.98E-01 | regulation of T-helper 2 cell differentiation |
| 45647 | 8.56E-02 | 1.98E-01 | negative regulation of erythrocyte differentiation |
| 45741 | 8.56E-02 | 1.98E-01 | positive regulation of epidermal growth factor receptor activity |
| 70371 | 8.56E-02 | 1.98E-01 | ERK1 and ERK2 cascade |
| 46173 | 8.56E-02 | 1.98E-01 | polyol biosynthetic process |
| 46487 | 8.56E-02 | 1.98E-01 | glyoxylate metabolic process |
| 46498 | 8.56E-02 | 1.98E-01 | S-adenosylhomocysteine metabolic process |
| 22027 | 8.56E-02 | 1.98E-01 | interkinetic nuclear migration |
| 30432 | 8.56E-02 | 1.98E-01 | peristalsis |
| 55017 | 8.56E-02 | 1.98E-01 | cardiac muscle tissue growth |
| 71453 | 8.56E-02 | 1.98E-01 | cellular response to oxygen levels |
| 71456 | 8.56E-02 | 1.98E-01 | cellular response to hypoxia |
| 6538 | 8.56E-02 | 1.98E-01 | glutamate catabolic process |
| 31122 | 8.56E-02 | 1.98E-01 | cytoplasmic microtubule organization |
| 6577 | 8.56E-02 | 1.98E-01 | betaine metabolic process |
| 14855 | 8.56E-02 | 1.98E-01 | striated muscle cell proliferation |
| 6729 | 8.56E-02 | 1.98E-01 | tetrahydrobiopterin biosynthetic process |
| 6787 | 8.56E-02 | 1.98E-01 | porphyrin catabolic process |
| 7210 | 8.56E-02 | 1.98E-01 | serotonin receptor signaling pathway |
| 7494 | 8.56E-02 | 1.98E-01 | midgut development |
| 48557 | 8.56E-02 | 1.98E-01 | embryonic digestive tract morphogenesis |
| 90022 | 8.56E-02 | 1.98E-01 | regulation of neutrophil chemotaxis |
| 50871 | 8.89E-02 | 2.05E-01 | positive regulation of B cell activation |
| 60070 | 8.89E-02 | 2.05E-01 | canonical Wnt receptor signaling pathway |
| 46651 | 8.89E-02 | 2.05E-01 | lymphocyte proliferation |
| 6721 | 8.89E-02 | 2.05E-01 | terpenoid metabolic process |
| 30324 | 8.98E-02 | 2.07E-01 | lung development |
| 55001 | 9.09E-02 | 2.09E-01 | muscle cell development |
| 30705 | 9.09E-02 | 2.09E-01 | cytoskeleton-dependent intracellular transport |
| 71375 | 9.11E-02 | 2.09E-01 | cellular response to peptide hormone stimulus |
| 48589 | 9.12E-02 | 2.09E-01 | developmental growth |
| 32925 | 9.16E-02 | 2.09E-01 | regulation of activin receptor signaling pathway |
| 9154 | 9.16E-02 | 2.09E-01 | purine ribonucleotide catabolic process |
| 42430 | 9.16E-02 | 2.09E-01 | indole and derivative metabolic process |
| 42434 | 9.16E-02 | 2.09E-01 | indole derivative metabolic process |
| 1542 | 9.16E-02 | 2.09E-01 | ovulation from ovarian follicle |
| 43266 | 9.16E-02 | 2.09E-01 | regulation of potassium ion transport |
| 43299 | 9.16E-02 | 2.09E-01 | leukocyte degranulation |
| 10559 | 9.16E-02 | 2.09E-01 | regulation of glycoprotein biosynthetic process |
| 51917 | 9.16E-02 | 2.09E-01 | regulation of fibrinolysis |
| 3009 | 9.16E-02 | 2.09E-01 | skeletal muscle contraction |
| 70228 | 9.16E-02 | 2.09E-01 | regulation of lymphocyte apoptosis |
| 45661 | 9.16E-02 | 2.09E-01 | regulation of myoblast differentiation |
| 45687 | 9.16E-02 | 2.09E-01 | positive regulation of glial cell differentiation |
| 45730 | 9.16E-02 | 2.09E-01 | respiratory burst |
| 30318 | 9.16E-02 | 2.09E-01 | melanocyte differentiation |
| 6586 | 9.16E-02 | 2.09E-01 | indolalkylamine metabolic process |
| 40036 | 9.16E-02 | 2.09E-01 | regulation of fibroblast growth factor receptor signaling pathway |
| 32091 | 9.16E-02 | 2.09E-01 | negative regulation of protein binding |
| 48662 | 9.16E-02 | 2.09E-01 | negative regulation of smooth muscle cell proliferation |
| 32653 | 9.16E-02 | 2.09E-01 | regulation of interleukin-10 production |
| 8637 | 9.41E-02 | 2.14E-01 | apoptotic mitochondrial changes |
| 1523 | 9.41E-02 | 2.14E-01 | retinoid metabolic process |
| 30512 | 9.41E-02 | 2.14E-01 | negative regulation of transforming growth factor beta receptor signaling pathway |
| 6635 | 9.41E-02 | 2.14E-01 | fatty acid beta-oxidation |
| 7595 | 9.41E-02 | 2.14E-01 | lactation |
| 16101 | 9.41E-02 | 2.14E-01 | diterpenoid metabolic process |
| 9108 | 9.66E-02 | 2.18E-01 | coenzyme biosynthetic process |
| 188 | 9.66E-02 | 2.18E-01 | inactivation of MAPK activity |
| 42133 | 9.66E-02 | 2.18E-01 | neurotransmitter metabolic process |
| 50848 | 9.66E-02 | 2.18E-01 | regulation of calcium-mediated signaling |
| 2218 | 9.66E-02 | 2.18E-01 | activation of innate immune response |
| 10676 | 9.66E-02 | 2.18E-01 | positive regulation of cellular carbohydrate metabolic process |
| 2712 | 9.66E-02 | 2.18E-01 | regulation of B cell mediated immunity |
| 2889 | 9.66E-02 | 2.18E-01 | regulation of immunoglobulin mediated immune response |
| 45103 | 9.66E-02 | 2.18E-01 | intermediate filament-based process |
| 45913 | 9.66E-02 | 2.18E-01 | positive regulation of carbohydrate metabolic process |
| 46520 | 9.66E-02 | 2.18E-01 | sphingoid biosynthetic process |
| 46635 | 9.66E-02 | 2.18E-01 | positive regulation of alpha-beta T cell activation |
| 6890 | 9.66E-02 | 2.18E-01 | retrograde vesicle-mediated transport, Golgi to ER |
| 48483 | 9.66E-02 | 2.18E-01 | autonomic nervous system development |
| 44267 | 9.77E-02 | 2.20E-01 | cellular protein metabolic process |
| 51648 | 9.84E-02 | 2.22E-01 | vesicle localization |
| 46496 | 9.84E-02 | 2.22E-01 | nicotinamide nucleotide metabolic process |
| 6953 | 9.84E-02 | 2.22E-01 | acute-phase response |
| 1892 | 9.86E-02 | 2.22E-01 | embryonic placenta development |
| 32526 | 9.86E-02 | 2.22E-01 | response to retinoic acid |
| 60173 | 1.01E-01 | 2.27E-01 | limb development |
| 48736 | 1.01E-01 | 2.27E-01 | appendage development |
| 6813 | 1.02E-01 | 2.30E-01 | potassium ion transport |
| 42509 | 1.05E-01 | 2.36E-01 | regulation of tyrosine phosphorylation of STAT protein |
| 6084 | 1.05E-01 | 2.36E-01 | acetyl-CoA metabolic process |
| 6664 | 1.05E-01 | 2.36E-01 | glycolipid metabolic process |
| 7589 | 1.05E-01 | 2.36E-01 | body fluid secretion |
| 32663 | 1.05E-01 | 2.36E-01 | regulation of interleukin-2 production |
| 51291 | 1.07E-01 | 2.39E-01 | protein heterooligomerization |
| 43933 | 1.08E-01 | 2.41E-01 | macromolecular complex subunit organization |
| 46546 | 1.08E-01 | 2.41E-01 | development of primary male sexual characteristics |
| 8544 | 1.08E-01 | 2.42E-01 | epidermis development |
| 45667 | 1.08E-01 | 2.42E-01 | regulation of osteoblast differentiation |
| 34440 | 1.08E-01 | 2.42E-01 | lipid oxidation |
| 19395 | 1.08E-01 | 2.42E-01 | fatty acid oxidation |
| 33238 | 1.10E-01 | 2.43E-01 | regulation of cellular amine metabolic process |
| 9225 | 1.10E-01 | 2.43E-01 | nucleotide-sugar metabolic process |
| 50869 | 1.10E-01 | 2.43E-01 | negative regulation of B cell activation |
| 50931 | 1.10E-01 | 2.43E-01 | pigment cell differentiation |
| 1829 | 1.10E-01 | 2.43E-01 | trophectodermal cell differentiation |
| 2028 | 1.10E-01 | 2.43E-01 | regulation of sodium ion transport |
| 2286 | 1.10E-01 | 2.43E-01 | T cell activation involved in immune response |
| 51668 | 1.10E-01 | 2.43E-01 | localization within membrane |
| 35329 | 1.10E-01 | 2.43E-01 | hippo signaling cascade |
| 51923 | 1.10E-01 | 2.43E-01 | sulfation |
| 30194 | 1.10E-01 | 2.43E-01 | positive regulation of blood coagulation |
| 14015 | 1.10E-01 | 2.43E-01 | positive regulation of gliogenesis |
| 6509 | 1.10E-01 | 2.43E-01 | membrane protein ectodomain proteolysis |
| 6625 | 1.10E-01 | 2.43E-01 | protein targeting to peroxisome |
| 7263 | 1.10E-01 | 2.43E-01 | nitric oxide mediated signal transduction |
| 32731 | 1.10E-01 | 2.43E-01 | positive regulation of interleukin-1 beta production |
| 46486 | 1.10E-01 | 2.44E-01 | glycerolipid metabolic process |
| 42220 | 1.11E-01 | 2.44E-01 | response to cocaine |
| 1990 | 1.11E-01 | 2.44E-01 | regulation of systemic arterial blood pressure by hormone |
| 10524 | 1.11E-01 | 2.44E-01 | positive regulation of calcium ion transport into cytosol |
| 2762 | 1.11E-01 | 2.44E-01 | negative regulation of myeloid leukocyte differentiation |
| 3229 | 1.11E-01 | 2.44E-01 | ventricular cardiac muscle tissue development |
| 45930 | 1.11E-01 | 2.44E-01 | negative regulation of mitotic cell cycle |
| 70848 | 1.11E-01 | 2.44E-01 | response to growth factor stimulus |
| 55010 | 1.11E-01 | 2.44E-01 | ventricular cardiac muscle tissue morphogenesis |
| 14073 | 1.11E-01 | 2.44E-01 | response to tropane |
| 6094 | 1.11E-01 | 2.44E-01 | gluconeogenesis |
| 31343 | 1.11E-01 | 2.44E-01 | positive regulation of cell killing |
| 6903 | 1.11E-01 | 2.44E-01 | vesicle targeting |
| 7031 | 1.11E-01 | 2.44E-01 | peroxisome organization |
| 32755 | 1.11E-01 | 2.44E-01 | positive regulation of interleukin-6 production |
| 51640 | 1.12E-01 | 2.45E-01 | organelle localization |
| 51090 | 1.13E-01 | 2.45E-01 | regulation of transcription factor activity |
| 90046 | 1.13E-01 | 2.45E-01 | regulation of transcription regulator activity |
| 70271 | 1.13E-01 | 2.45E-01 | protein complex biogenesis |
| 6461 | 1.13E-01 | 2.45E-01 | protein complex assembly |
| 9260 | 1.13E-01 | 2.45E-01 | ribonucleotide biosynthetic process |
| 33146 | 1.13E-01 | 2.45E-01 | regulation of estrogen receptor signaling pathway |
| 33194 | 1.13E-01 | 2.45E-01 | response to hydroperoxide |
| 42059 | 1.13E-01 | 2.45E-01 | negative regulation of epidermal growth factor receptor signaling pathway |
| 1573 | 1.13E-01 | 2.45E-01 | ganglioside metabolic process |
| 34383 | 1.13E-01 | 2.45E-01 | low-density lipoprotein particle clearance |
| 42730 | 1.13E-01 | 2.45E-01 | fibrinolysis |
| 51001 | 1.13E-01 | 2.45E-01 | negative regulation of nitric-oxide synthase activity |
| 1914 | 1.13E-01 | 2.45E-01 | regulation of T cell mediated cytotoxicity |
| 42921 | 1.13E-01 | 2.45E-01 | glucocorticoid receptor signaling pathway |
| 10269 | 1.13E-01 | 2.45E-01 | response to selenium ion |
| 43113 | 1.13E-01 | 2.45E-01 | receptor clustering |
| 43383 | 1.13E-01 | 2.45E-01 | negative T cell selection |
| 2548 | 1.13E-01 | 2.45E-01 | monocyte chemotaxis |
| 2686 | 1.13E-01 | 2.45E-01 | negative regulation of leukocyte migration |
| 43647 | 1.13E-01 | 2.45E-01 | inositol phosphate metabolic process |
| 10894 | 1.13E-01 | 2.45E-01 | negative regulation of steroid biosynthetic process |
| 2902 | 1.13E-01 | 2.45E-01 | regulation of B cell apoptosis |
| 3016 | 1.13E-01 | 2.45E-01 | respiratory system process |
| 60419 | 1.13E-01 | 2.45E-01 | heart growth |
| 60558 | 1.13E-01 | 2.45E-01 | regulation of calcidiol 1-monooxygenase activity |
| 60993 | 1.13E-01 | 2.45E-01 | kidney morphogenesis |
| 61098 | 1.13E-01 | 2.45E-01 | positive regulation of protein tyrosine kinase activity |
| 45576 | 1.13E-01 | 2.45E-01 | mast cell activation |
| 45624 | 1.13E-01 | 2.45E-01 | positive regulation of T-helper cell differentiation |
| 21546 | 1.13E-01 | 2.45E-01 | rhombomere development |
| 70723 | 1.13E-01 | 2.45E-01 | response to cholesterol |
| 46641 | 1.13E-01 | 2.45E-01 | positive regulation of alpha-beta T cell proliferation |
| 14049 | 1.13E-01 | 2.45E-01 | positive regulation of glutamate secretion |
| 6012 | 1.13E-01 | 2.45E-01 | galactose metabolic process |
| 6101 | 1.13E-01 | 2.45E-01 | citrate metabolic process |
| 14829 | 1.13E-01 | 2.45E-01 | vascular smooth muscle contraction |
| 7016 | 1.13E-01 | 2.45E-01 | cytoskeletal anchoring at plasma membrane |
| 48484 | 1.13E-01 | 2.45E-01 | enteric nervous system development |
| 48485 | 1.13E-01 | 2.45E-01 | sympathetic nervous system development |
| 32372 | 1.13E-01 | 2.45E-01 | negative regulation of sterol transport |
| 32375 | 1.13E-01 | 2.45E-01 | negative regulation of cholesterol transport |
| 32689 | 1.13E-01 | 2.45E-01 | negative regulation of interferon-gamma production |
| 51216 | 1.15E-01 | 2.48E-01 | cartilage development |
| 51924 | 1.15E-01 | 2.48E-01 | regulation of calcium ion transport |
| 6959 | 1.15E-01 | 2.48E-01 | humoral immune response |
| 48738 | 1.15E-01 | 2.48E-01 | cardiac muscle tissue development |
| 51101 | 1.16E-01 | 2.51E-01 | regulation of DNA binding |
| 42278 | 1.17E-01 | 2.53E-01 | purine nucleoside metabolic process |
| 46128 | 1.17E-01 | 2.53E-01 | purine ribonucleoside metabolic process |
| 22406 | 1.17E-01 | 2.53E-01 | membrane docking |
| 32649 | 1.17E-01 | 2.53E-01 | regulation of interferon-gamma production |
| 9119 | 1.18E-01 | 2.53E-01 | ribonucleoside metabolic process |
| 43270 | 1.18E-01 | 2.53E-01 | positive regulation of ion transport |
| 6638 | 1.18E-01 | 2.53E-01 | neutral lipid metabolic process |
| 43193 | 1.18E-01 | 2.54E-01 | positive regulation of gene-specific transcription |
| 6164 | 1.18E-01 | 2.54E-01 | purine nucleotide biosynthetic process |
| 48598 | 1.18E-01 | 2.54E-01 | embryonic morphogenesis |
| 9152 | 1.19E-01 | 2.56E-01 | purine ribonucleotide biosynthetic process |
| 33189 | 1.19E-01 | 2.56E-01 | response to vitamin A |
| 1505 | 1.19E-01 | 2.56E-01 | regulation of neurotransmitter levels |
| 187 | 1.22E-01 | 2.62E-01 | activation of MAPK activity |
| 6919 | 1.24E-01 | 2.65E-01 | activation of caspase activity |
| 10564 | 1.24E-01 | 2.65E-01 | regulation of cell cycle process |
| 31497 | 1.24E-01 | 2.66E-01 | chromatin assembly |
| 65003 | 1.24E-01 | 2.66E-01 | macromolecular complex assembly |
| 51098 | 1.25E-01 | 2.66E-01 | regulation of binding |
| 9066 | 1.25E-01 | 2.67E-01 | aspartate family amino acid metabolic process |
| 9112 | 1.25E-01 | 2.67E-01 | nucleobase metabolic process |
| 42177 | 1.25E-01 | 2.67E-01 | negative regulation of protein catabolic process |
| 42531 | 1.25E-01 | 2.67E-01 | positive regulation of tyrosine phosphorylation of STAT protein |
| 51897 | 1.25E-01 | 2.67E-01 | positive regulation of protein kinase B signaling cascade |
| 45685 | 1.25E-01 | 2.67E-01 | regulation of glial cell differentiation |
| 46326 | 1.25E-01 | 2.67E-01 | positive regulation of glucose import |
| 6691 | 1.25E-01 | 2.67E-01 | leukotriene metabolic process |
| 6706 | 1.25E-01 | 2.67E-01 | steroid catabolic process |
| 6821 | 1.25E-01 | 2.67E-01 | chloride transport |
| 48066 | 1.25E-01 | 2.67E-01 | developmental pigmentation |
| 32642 | 1.25E-01 | 2.67E-01 | regulation of chemokine production |
| 32651 | 1.25E-01 | 2.67E-01 | regulation of interleukin-1 beta production |
| 42063 | 1.28E-01 | 2.71E-01 | gliogenesis |
| 16331 | 1.28E-01 | 2.71E-01 | morphogenesis of embryonic epithelium |
| 8593 | 1.29E-01 | 2.71E-01 | regulation of Notch signaling pathway |
| 9261 | 1.29E-01 | 2.71E-01 | ribonucleotide catabolic process |
| 42771 | 1.29E-01 | 2.71E-01 | DNA damage response, signal transduction by p53 class mediator resulting in induction of apoptosis |
| 50982 | 1.29E-01 | 2.71E-01 | detection of mechanical stimulus |
| 1937 | 1.29E-01 | 2.71E-01 | negative regulation of endothelial cell proliferation |
| 1954 | 1.29E-01 | 2.71E-01 | positive regulation of cell-matrix adhesion |
| 2224 | 1.29E-01 | 2.71E-01 | toll-like receptor signaling pathway |
| 43574 | 1.29E-01 | 2.71E-01 | peroxisomal transport |
| 10976 | 1.29E-01 | 2.71E-01 | positive regulation of neuron projection development |
| 46580 | 1.29E-01 | 2.71E-01 | negative regulation of Ras protein signal transduction |
| 46847 | 1.29E-01 | 2.71E-01 | filopodium assembly |
| 6957 | 1.29E-01 | 2.71E-01 | complement activation, alternative pathway |
| 7274 | 1.29E-01 | 2.71E-01 | neuromuscular synaptic transmission |
| 7620 | 1.29E-01 | 2.71E-01 | copulation |
| 32369 | 1.29E-01 | 2.71E-01 | negative regulation of lipid transport |
| 48873 | 1.29E-01 | 2.71E-01 | homeostasis of number of cells within a tissue |
| 32720 | 1.29E-01 | 2.71E-01 | negative regulation of tumor necrosis factor production |
| 10948 | 1.30E-01 | 2.73E-01 | negative regulation of cell cycle process |
| 19362 | 1.30E-01 | 2.73E-01 | pyridine nucleotide metabolic process |
| 42446 | 1.30E-01 | 2.73E-01 | hormone biosynthetic process |
| 50994 | 1.30E-01 | 2.73E-01 | regulation of lipid catabolic process |
| 51896 | 1.30E-01 | 2.73E-01 | regulation of protein kinase B signaling cascade |
| 60711 | 1.30E-01 | 2.73E-01 | labyrinthine layer development |
| 30837 | 1.30E-01 | 2.73E-01 | negative regulation of actin filament polymerization |
| 30888 | 1.30E-01 | 2.73E-01 | regulation of B cell proliferation |
| 48146 | 1.30E-01 | 2.73E-01 | positive regulation of fibroblast proliferation |
| 48489 | 1.30E-01 | 2.73E-01 | synaptic vesicle transport |
| 48610 | 1.30E-01 | 2.74E-01 | reproductive cellular process |
| 43010 | 1.31E-01 | 2.76E-01 | camera-type eye development |
| 9206 | 1.32E-01 | 2.77E-01 | purine ribonucleoside triphosphate biosynthetic process |
| 51188 | 1.32E-01 | 2.77E-01 | cofactor biosynthetic process |
| 46034 | 1.32E-01 | 2.77E-01 | ATP metabolic process |
| 79 | 1.33E-01 | 2.78E-01 | regulation of cyclin-dependent protein kinase activity |
| 50864 | 1.38E-01 | 2.88E-01 | regulation of B cell activation |
| 51250 | 1.38E-01 | 2.88E-01 | negative regulation of lymphocyte activation |
| 6662 | 1.38E-01 | 2.88E-01 | glycerol ether metabolic process |
| 9145 | 1.39E-01 | 2.91E-01 | purine nucleoside triphosphate biosynthetic process |
| 9201 | 1.39E-01 | 2.91E-01 | ribonucleoside triphosphate biosynthetic process |
| 35107 | 1.39E-01 | 2.91E-01 | appendage morphogenesis |
| 35108 | 1.39E-01 | 2.91E-01 | limb morphogenesis |
| 8542 | 1.41E-01 | 2.91E-01 | visual learning |
| 42558 | 1.41E-01 | 2.91E-01 | pteridine and derivative metabolic process |
| 50852 | 1.41E-01 | 2.91E-01 | T cell receptor signaling pathway |
| 43449 | 1.41E-01 | 2.91E-01 | cellular alkene metabolic process |
| 10828 | 1.41E-01 | 2.91E-01 | positive regulation of glucose transport |
| 60740 | 1.41E-01 | 2.91E-01 | prostate gland epithelium morphogenesis |
| 7043 | 1.41E-01 | 2.91E-01 | cell-cell junction assembly |
| 7215 | 1.41E-01 | 2.91E-01 | glutamate signaling pathway |
| 6641 | 1.41E-01 | 2.91E-01 | triglyceride metabolic process |
| 32675 | 1.41E-01 | 2.91E-01 | regulation of interleukin-6 production |
| 22415 | 1.42E-01 | 2.91E-01 | viral reproductive process |
| 8064 | 1.42E-01 | 2.91E-01 | regulation of actin polymerization or depolymerization |
| 8211 | 1.43E-01 | 2.91E-01 | glucocorticoid metabolic process |
| 90136 | 1.43E-01 | 2.91E-01 | epithelial cell-cell adhesion |
| 9396 | 1.43E-01 | 2.91E-01 | folic acid and derivative biosynthetic process |
| 34109 | 1.43E-01 | 2.91E-01 | homotypic cell-cell adhesion |
| 50665 | 1.43E-01 | 2.91E-01 | hydrogen peroxide biosynthetic process |
| 50732 | 1.43E-01 | 2.91E-01 | negative regulation of peptidyl-tyrosine phosphorylation |
| 50775 | 1.43E-01 | 2.91E-01 | positive regulation of dendrite morphogenesis |
| 50884 | 1.43E-01 | 2.91E-01 | neuromuscular process controlling posture |
| 1823 | 1.43E-01 | 2.91E-01 | mesonephros development |
| 2016 | 1.43E-01 | 2.91E-01 | regulation of blood volume by renin-angiotensin |
| 51238 | 1.43E-01 | 2.91E-01 | sequestering of metal ion |
| 43092 | 1.43E-01 | 2.91E-01 | L-amino acid import |
| 10470 | 1.43E-01 | 2.91E-01 | regulation of gastrulation |
| 10623 | 1.43E-01 | 2.91E-01 | developmental programmed cell death |
| 35235 | 1.43E-01 | 2.91E-01 | ionotropic glutamate receptor signaling pathway |
| 60004 | 1.43E-01 | 2.91E-01 | reflex |
| 10875 | 1.43E-01 | 2.91E-01 | positive regulation of cholesterol efflux |
| 60037 | 1.43E-01 | 2.91E-01 | pharyngeal system development |
| 60052 | 1.43E-01 | 2.91E-01 | neurofilament cytoskeleton organization |
| 2861 | 1.43E-01 | 2.91E-01 | regulation of inflammatory response to antigenic stimulus |
| 60231 | 1.43E-01 | 2.91E-01 | mesenchymal to epithelial transition |
| 60347 | 1.43E-01 | 2.91E-01 | heart trabecula formation |
| 19433 | 1.43E-01 | 2.91E-01 | triglyceride catabolic process |
| 60396 | 1.43E-01 | 2.91E-01 | growth hormone receptor signaling pathway |
| 3071 | 1.43E-01 | 2.91E-01 | renal system process involved in regulation of systemic arterial blood pressure |
| 60669 | 1.43E-01 | 2.91E-01 | embryonic placenta morphogenesis |
| 60768 | 1.43E-01 | 2.91E-01 | regulation of epithelial cell proliferation involved in prostate gland development |
| 45080 | 1.43E-01 | 2.91E-01 | positive regulation of chemokine biosynthetic process |
| 45662 | 1.43E-01 | 2.91E-01 | negative regulation of myoblast differentiation |
| 45747 | 1.43E-01 | 2.91E-01 | positive regulation of Notch signaling pathway |
| 46543 | 1.43E-01 | 2.91E-01 | development of secondary female sexual characteristics |
| 71378 | 1.43E-01 | 2.91E-01 | cellular response to growth hormone stimulus |
| 30903 | 1.43E-01 | 2.91E-01 | notochord development |
| 72009 | 1.43E-01 | 2.91E-01 | nephron epithelium development |
| 6677 | 1.43E-01 | 2.91E-01 | glycosylceramide metabolic process |
| 6817 | 1.43E-01 | 2.91E-01 | phosphate transport |
| 6893 | 1.43E-01 | 2.91E-01 | Golgi to plasma membrane transport |
| 6895 | 1.43E-01 | 2.91E-01 | Golgi to endosome transport |
| 31503 | 1.43E-01 | 2.91E-01 | protein complex localization |
| 7164 | 1.43E-01 | 2.91E-01 | establishment of tissue polarity |
| 48532 | 1.43E-01 | 2.91E-01 | anatomical structure arrangement |
| 32303 | 1.43E-01 | 2.91E-01 | regulation of icosanoid secretion |
| 32305 | 1.43E-01 | 2.91E-01 | positive regulation of icosanoid secretion |
| 32306 | 1.43E-01 | 2.91E-01 | regulation of prostaglandin secretion |
| 32308 | 1.43E-01 | 2.91E-01 | positive regulation of prostaglandin secretion |
| 51650 | 1.43E-01 | 2.91E-01 | establishment of vesicle localization |
| 43467 | 1.43E-01 | 2.91E-01 | regulation of generation of precursor metabolites and energy |
| 51705 | 1.43E-01 | 2.91E-01 | behavioral interaction between organisms |
| 46320 | 1.43E-01 | 2.91E-01 | regulation of fatty acid oxidation |
| 46456 | 1.43E-01 | 2.91E-01 | icosanoid biosynthetic process |
| 34404 | 1.44E-01 | 2.91E-01 | nucleobase, nucleoside and nucleotide biosynthetic process |
| 34654 | 1.44E-01 | 2.91E-01 | nucleobase, nucleoside, nucleotide and nucleic acid biosynthetic process |
| 9205 | 1.46E-01 | 2.91E-01 | purine ribonucleoside triphosphate metabolic process |
| 50954 | 1.46E-01 | 2.91E-01 | sensory perception of mechanical stimulus |
| 43583 | 1.46E-01 | 2.91E-01 | ear development |
| 97 | 1.49E-01 | 2.91E-01 | sulfur amino acid biosynthetic process |
| 32872 | 1.49E-01 | 2.91E-01 | regulation of stress-activated MAPK cascade |
| 1502 | 1.49E-01 | 2.91E-01 | cartilage condensation |
| 51402 | 1.49E-01 | 2.91E-01 | neuron apoptosis |
| 43666 | 1.49E-01 | 2.91E-01 | regulation of phosphoprotein phosphatase activity |
| 60395 | 1.49E-01 | 2.91E-01 | SMAD protein signal transduction |
| 3279 | 1.49E-01 | 2.91E-01 | cardiac septum development |
| 45058 | 1.49E-01 | 2.91E-01 | T cell selection |
| 45599 | 1.49E-01 | 2.91E-01 | negative regulation of fat cell differentiation |
| 45823 | 1.49E-01 | 2.91E-01 | positive regulation of heart contraction |
| 45921 | 1.49E-01 | 2.91E-01 | positive regulation of exocytosis |
| 6103 | 1.49E-01 | 2.91E-01 | 2-oxoglutarate metabolic process |
| 80010 | 1.49E-01 | 2.91E-01 | regulation of oxygen and reactive oxygen species metabolic process |
| 31114 | 1.49E-01 | 2.91E-01 | regulation of microtubule depolymerization |
| 6825 | 1.49E-01 | 2.91E-01 | copper ion transport |
| 7026 | 1.49E-01 | 2.91E-01 | negative regulation of microtubule depolymerization |
| 7176 | 1.49E-01 | 2.91E-01 | regulation of epidermal growth factor receptor activity |
| 15807 | 1.49E-01 | 2.91E-01 | L-amino acid transport |
| 15844 | 1.49E-01 | 2.91E-01 | monoamine transport |
| 9199 | 1.53E-01 | 2.91E-01 | ribonucleoside triphosphate metabolic process |
| 7420 | 1.53E-01 | 2.91E-01 | brain development |
| 9142 | 1.54E-01 | 2.91E-01 | nucleoside triphosphate biosynthetic process |
| 21915 | 1.54E-01 | 2.91E-01 | neural tube development |
| 48706 | 1.55E-01 | 2.91E-01 | embryonic skeletal system development |
| 34613 | 1.55E-01 | 2.91E-01 | cellular protein localization |
| 9593 | 1.57E-01 | 2.91E-01 | detection of chemical stimulus |
| 42439 | 1.57E-01 | 2.91E-01 | ethanolamine and derivative metabolic process |
| 51899 | 1.57E-01 | 2.91E-01 | membrane depolarization |
| 3206 | 1.57E-01 | 2.91E-01 | cardiac chamber morphogenesis |
| 21987 | 1.57E-01 | 2.91E-01 | cerebral cortex development |
| 33077 | 1.57E-01 | 2.91E-01 | T cell differentiation in the thymus |
| 2260 | 1.57E-01 | 2.91E-01 | lymphocyte homeostasis |
| 43542 | 1.57E-01 | 2.91E-01 | endothelial cell migration |
| 3208 | 1.57E-01 | 2.91E-01 | cardiac ventricle morphogenesis |
| 30890 | 1.57E-01 | 2.91E-01 | positive regulation of B cell proliferation |
| 7040 | 1.57E-01 | 2.91E-01 | lysosome organization |
| 34220 | 1.59E-01 | 2.91E-01 | ion transmembrane transport |
| 42472 | 1.59E-01 | 2.91E-01 | inner ear morphogenesis |
| 18904 | 1.59E-01 | 2.91E-01 | organic ether metabolic process |
| 61025 | 1.59E-01 | 2.91E-01 | membrane fusion |
| 7389 | 1.59E-01 | 2.91E-01 | pattern specification process |
| 30832 | 1.62E-01 | 2.91E-01 | regulation of actin filament length |
| 32796 | 1.62E-01 | 2.91E-01 | uropod organization |
| 32808 | 1.62E-01 | 2.91E-01 | lacrimal gland development |
| 42 | 1.62E-01 | 2.91E-01 | protein targeting to Golgi |
| 90192 | 1.62E-01 | 2.91E-01 | regulation of glomerulus development |
| 98 | 1.62E-01 | 2.91E-01 | sulfur amino acid catabolic process |
| 32898 | 1.62E-01 | 2.91E-01 | neurotrophin production |
| 32902 | 1.62E-01 | 2.91E-01 | nerve growth factor production |
| 16539 | 1.62E-01 | 2.91E-01 | intein-mediated protein splicing |
| 32928 | 1.62E-01 | 2.91E-01 | regulation of superoxide anion generation |
| 32929 | 1.62E-01 | 2.91E-01 | negative regulation of superoxide anion generation |
| 32959 | 1.62E-01 | 2.91E-01 | inositol trisphosphate biosynthetic process |
| 90322 | 1.62E-01 | 2.91E-01 | regulation of superoxide metabolic process |
| 33026 | 1.62E-01 | 2.91E-01 | negative regulation of mast cell apoptosis |
| 33084 | 1.62E-01 | 2.91E-01 | regulation of immature T cell proliferation in the thymus |
| 33087 | 1.62E-01 | 2.91E-01 | negative regulation of immature T cell proliferation |
| 33088 | 1.62E-01 | 2.91E-01 | negative regulation of immature T cell proliferation in the thymus |
| 8611 | 1.62E-01 | 2.91E-01 | ether lipid biosynthetic process |
| 33484 | 1.62E-01 | 2.91E-01 | nitric oxide homeostasis |
| 733 | 1.62E-01 | 2.91E-01 | DNA strand renaturation |
| 33552 | 1.62E-01 | 2.91E-01 | response to vitamin B3 |
| 33602 | 1.62E-01 | 2.91E-01 | negative regulation of dopamine secretion |
| 33623 | 1.62E-01 | 2.91E-01 | regulation of integrin activation |
| 33625 | 1.62E-01 | 2.91E-01 | positive regulation of integrin activation |
| 9082 | 1.62E-01 | 2.91E-01 | branched chain family amino acid biosynthetic process |
| 9093 | 1.62E-01 | 2.91E-01 | cysteine catabolic process |
| 9128 | 1.62E-01 | 2.91E-01 | purine nucleoside monophosphate catabolic process |
| 9137 | 1.62E-01 | 2.91E-01 | purine nucleoside diphosphate catabolic process |
| 9158 | 1.62E-01 | 2.91E-01 | ribonucleoside monophosphate catabolic process |
| 9169 | 1.62E-01 | 2.91E-01 | purine ribonucleoside monophosphate catabolic process |
| 9181 | 1.62E-01 | 2.91E-01 | purine ribonucleoside diphosphate catabolic process |
| 9235 | 1.62E-01 | 2.91E-01 | cobalamin metabolic process |
| 33864 | 1.62E-01 | 2.91E-01 | positive regulation of NAD(P)H oxidase activity |
| 33875 | 1.62E-01 | 2.91E-01 | ribonucleoside bisphosphate metabolic process |
| 9439 | 1.62E-01 | 2.91E-01 | cyanate metabolic process |
| 9440 | 1.62E-01 | 2.91E-01 | cyanate catabolic process |
| 9450 | 1.62E-01 | 2.91E-01 | gamma-aminobutyric acid catabolic process |
| 34032 | 1.62E-01 | 2.91E-01 | purine nucleoside bisphosphate metabolic process |
| 34035 | 1.62E-01 | 2.91E-01 | purine ribonucleoside bisphosphate metabolic process |
| 50427 | 1.62E-01 | 2.91E-01 | 3'-phosphoadenosine 5'-phosphosulfate metabolic process |
| 34059 | 1.62E-01 | 2.91E-01 | response to anoxia |
| 1300 | 1.62E-01 | 2.91E-01 | chronological cell aging |
| 34112 | 1.62E-01 | 2.91E-01 | positive regulation of homotypic cell-cell adhesion |
| 34116 | 1.62E-01 | 2.91E-01 | positive regulation of heterotypic cell-cell adhesion |
| 9553 | 1.62E-01 | 2.91E-01 | embryo sac development |
| 34134 | 1.62E-01 | 2.91E-01 | toll-like receptor 2 signaling pathway |
| 9561 | 1.62E-01 | 2.91E-01 | megagametogenesis |
| 42335 | 1.62E-01 | 2.91E-01 | cuticle development |
| 42339 | 1.62E-01 | 2.91E-01 | keratan sulfate metabolic process |
| 42404 | 1.62E-01 | 2.91E-01 | thyroid hormone catabolic process |
| 50666 | 1.62E-01 | 2.91E-01 | regulation of homocysteine metabolic process |
| 1550 | 1.62E-01 | 2.91E-01 | ovarian cumulus expansion |
| 42524 | 1.62E-01 | 2.91E-01 | negative regulation of tyrosine phosphorylation of Stat5 protein |
| 34389 | 1.62E-01 | 2.91E-01 | lipid particle organization |
| 50774 | 1.62E-01 | 2.91E-01 | negative regulation of dendrite morphogenesis |
| 1660 | 1.62E-01 | 2.91E-01 | fever |
| 42637 | 1.62E-01 | 2.91E-01 | catagen |
| 1692 | 1.62E-01 | 2.91E-01 | histamine metabolic process |
| 50849 | 1.62E-01 | 2.91E-01 | negative regulation of calcium-mediated signaling |
| 42693 | 1.62E-01 | 2.91E-01 | muscle cell fate commitment |
| 1765 | 1.62E-01 | 2.91E-01 | membrane raft assembly |
| 1767 | 1.62E-01 | 2.91E-01 | establishment of lymphocyte polarity |
| 1768 | 1.62E-01 | 2.91E-01 | establishment of T cell polarity |
| 18153 | 1.62E-01 | 2.91E-01 | isopeptide cross-linking via N6-(L-isoglutamyl)-L-lysine |
| 42760 | 1.62E-01 | 2.91E-01 | very long-chain fatty acid catabolic process |
| 10002 | 1.62E-01 | 2.91E-01 | cardioblast differentiation |
| 18199 | 1.62E-01 | 2.91E-01 | peptidyl-glutamine modification |
| 34619 | 1.62E-01 | 2.91E-01 | cellular chaperone-mediated protein complex assembly |
| 10044 | 1.62E-01 | 2.91E-01 | response to aluminum ion |
| 1868 | 1.62E-01 | 2.91E-01 | regulation of complement activation, lectin pathway |
| 1869 | 1.62E-01 | 2.91E-01 | negative regulation of complement activation, lectin pathway |
| 18262 | 1.62E-01 | 2.91E-01 | isopeptide cross-linking |
| 10092 | 1.62E-01 | 2.91E-01 | specification of organ identity |
| 1941 | 1.62E-01 | 2.91E-01 | postsynaptic membrane organization |
| 18352 | 1.62E-01 | 2.91E-01 | protein-pyridoxal-5-phosphate linkage |
| 51125 | 1.62E-01 | 2.91E-01 | regulation of actin nucleation |
| 34755 | 1.62E-01 | 2.91E-01 | iron ion transmembrane transport |
| 1997 | 1.62E-01 | 2.91E-01 | positive regulation of the force of heart contraction by epinephrine-norepinephrine |
| 2005 | 1.62E-01 | 2.91E-01 | angiotensin catabolic process in blood |
| 2017 | 1.62E-01 | 2.91E-01 | regulation of blood volume by renal aldosterone |
| 2018 | 1.62E-01 | 2.91E-01 | renin-angiotensin regulation of aldosterone production |
| 2025 | 1.62E-01 | 2.91E-01 | vasodilation by norepinephrine-epinephrine involved in regulation of systemic arterial blood pressure |
| 42986 | 1.62E-01 | 2.91E-01 | positive regulation of amyloid precursor protein biosynthetic process |
| 2032 | 1.62E-01 | 2.91E-01 | desensitization of G-protein coupled receptor protein signaling pathway by arrestin |
| 2043 | 1.62E-01 | 2.91E-01 | blood vessel endothelial cell proliferation involved in sprouting angiogenesis |
| 2138 | 1.62E-01 | 2.91E-01 | retinoic acid biosynthetic process |
| 43117 | 1.62E-01 | 2.91E-01 | positive regulation of vascular permeability |
| 43163 | 1.62E-01 | 2.91E-01 | cell envelope organization |
| 10519 | 1.62E-01 | 2.91E-01 | negative regulation of phospholipase activity |
| 35104 | 1.62E-01 | 2.91E-01 | positive regulation of transcription via sterol regulatory element binding |
| 35106 | 1.62E-01 | 2.91E-01 | operant conditioning |
| 43366 | 1.62E-01 | 2.91E-01 | beta selection |
| 51589 | 1.62E-01 | 2.91E-01 | negative regulation of neurotransmitter transport |
| 2439 | 1.62E-01 | 2.91E-01 | chronic inflammatory response to antigenic stimulus |
| 10642 | 1.62E-01 | 2.91E-01 | negative regulation of platelet-derived growth factor receptor signaling pathway |
| 10670 | 1.62E-01 | 2.91E-01 | positive regulation of oxygen and reactive oxygen species metabolic process |
| 18874 | 1.62E-01 | 2.91E-01 | benzoate metabolic process |
| 43482 | 1.62E-01 | 2.91E-01 | cellular pigment accumulation |
| 10719 | 1.62E-01 | 2.91E-01 | negative regulation of epithelial to mesenchymal transition |
| 10735 | 1.62E-01 | 2.91E-01 | positive regulation of transcription via serum response element binding |
| 35330 | 1.62E-01 | 2.91E-01 | regulation of hippo signaling cascade |
| 10762 | 1.62E-01 | 2.91E-01 | regulation of fibroblast migration |
| 10763 | 1.62E-01 | 2.91E-01 | positive regulation of fibroblast migration |
| 2577 | 1.62E-01 | 2.91E-01 | regulation of antigen processing and presentation |
| 2604 | 1.62E-01 | 2.91E-01 | regulation of dendritic cell antigen processing and presentation |
| 35385 | 1.62E-01 | 2.91E-01 | Roundabout signaling pathway |
| 51773 | 1.62E-01 | 2.91E-01 | positive regulation of nitric-oxide synthase 2 biosynthetic process |
| 10825 | 1.62E-01 | 2.91E-01 | positive regulation of centrosome duplication |
| 10838 | 1.62E-01 | 2.91E-01 | positive regulation of keratinocyte proliferation |
| 10842 | 1.62E-01 | 2.91E-01 | retina layer formation |
| 43615 | 1.62E-01 | 2.91E-01 | astrocyte cell migration |
| 2661 | 1.62E-01 | 2.91E-01 | regulation of B cell tolerance induction |
| 2663 | 1.62E-01 | 2.91E-01 | positive regulation of B cell tolerance induction |
| 60012 | 1.62E-01 | 2.91E-01 | synaptic transmission, glycinergic |
| 60027 | 1.62E-01 | 2.91E-01 | convergent extension involved in gastrulation |
| 2716 | 1.62E-01 | 2.91E-01 | negative regulation of natural killer cell mediated immunity |
| 51902 | 1.62E-01 | 2.91E-01 | negative regulation of mitochondrial depolarization |
| 2752 | 1.62E-01 | 2.91E-01 | cell surface pattern recognition receptor signaling pathway |
| 10957 | 1.62E-01 | 2.91E-01 | negative regulation of vitamin D biosynthetic process |
| 51932 | 1.62E-01 | 2.91E-01 | synaptic transmission, GABAergic |
| 51957 | 1.62E-01 | 2.91E-01 | positive regulation of amino acid transport |
| 60151 | 1.62E-01 | 2.91E-01 | peroxisome localization |
| 60152 | 1.62E-01 | 2.91E-01 | microtubule-based peroxisome localization |
| 60166 | 1.62E-01 | 2.91E-01 | olfactory pit development |
| 60179 | 1.62E-01 | 2.91E-01 | male mating behavior |
| 19254 | 1.62E-01 | 2.91E-01 | carnitine metabolic process, CoA-linked |
| 2885 | 1.62E-01 | 2.91E-01 | positive regulation of hypersensitivity |
| 60315 | 1.62E-01 | 2.91E-01 | negative regulation of ryanodine-sensitive calcium-release channel activity |
| 43932 | 1.62E-01 | 2.91E-01 | ossification involved in bone remodeling |
| 60363 | 1.62E-01 | 2.91E-01 | cranial suture morphogenesis |
| 60364 | 1.62E-01 | 2.91E-01 | frontal suture morphogenesis |
| 60370 | 1.62E-01 | 2.91E-01 | susceptibility to T cell mediated cytotoxicity |
| 60399 | 1.62E-01 | 2.91E-01 | positive regulation of growth hormone receptor signaling pathway |
| 19442 | 1.62E-01 | 2.91E-01 | tryptophan catabolic process to acetyl-CoA |
| 19448 | 1.62E-01 | 2.91E-01 | L-cysteine catabolic process |
| 60447 | 1.62E-01 | 2.91E-01 | bud outgrowth involved in lung branching |
| 60449 | 1.62E-01 | 2.91E-01 | bud elongation involved in lung branching |
| 3130 | 1.62E-01 | 2.91E-01 | BMP signaling pathway involved in heart induction |
| 3159 | 1.62E-01 | 2.91E-01 | morphogenesis of an endothelium |
| 3170 | 1.62E-01 | 2.91E-01 | heart valve development |
| 3179 | 1.62E-01 | 2.91E-01 | heart valve morphogenesis |
| 44154 | 1.62E-01 | 2.91E-01 | histone H3-K14 acetylation |
| 44273 | 1.62E-01 | 2.91E-01 | sulfur compound catabolic process |
| 60677 | 1.62E-01 | 2.91E-01 | ureteric bud elongation |
| 3348 | 1.62E-01 | 2.91E-01 | cardiac endothelial cell differentiation |
| 60696 | 1.62E-01 | 2.91E-01 | regulation of phospholipid catabolic process |
| 60732 | 1.62E-01 | 2.91E-01 | positive regulation of inositol phosphate biosynthetic process |
| 60738 | 1.62E-01 | 2.91E-01 | epithelial-mesenchymal signaling involved in prostate gland development |
| 60741 | 1.62E-01 | 2.91E-01 | prostate gland stromal morphogenesis |
| 19853 | 1.62E-01 | 2.91E-01 | L-ascorbic acid biosynthetic process |
| 19860 | 1.62E-01 | 2.91E-01 | uracil metabolic process |
| 19896 | 1.62E-01 | 2.91E-01 | axon transport of mitochondrion |
| 60913 | 1.62E-01 | 2.91E-01 | cardiac cell fate determination |
| 60956 | 1.62E-01 | 2.91E-01 | endocardial cell differentiation |
| 61033 | 1.62E-01 | 2.91E-01 | secretion by lung epithelial cell involved in lung growth |
| 61043 | 1.62E-01 | 2.91E-01 | regulation of vascular wound healing |
| 61154 | 1.62E-01 | 2.91E-01 | endothelial tube morphogenesis |
| 45082 | 1.62E-01 | 2.91E-01 | positive regulation of interleukin-10 biosynthetic process |
| 45229 | 1.62E-01 | 2.91E-01 | external encapsulating structure organization |
| 45402 | 1.62E-01 | 2.91E-01 | regulation of interleukin-4 biosynthetic process |
| 45404 | 1.62E-01 | 2.91E-01 | positive regulation of interleukin-4 biosynthetic process |
| 70075 | 1.62E-01 | 2.91E-01 | tear secretion |
| 70094 | 1.62E-01 | 2.91E-01 | positive regulation of glucagon secretion |
| 70099 | 1.62E-01 | 2.91E-01 | regulation of chemokine-mediated signaling pathway |
| 70100 | 1.62E-01 | 2.91E-01 | negative regulation of chemokine-mediated signaling pathway |
| 45542 | 1.62E-01 | 2.91E-01 | positive regulation of cholesterol biosynthetic process |
| 45602 | 1.62E-01 | 2.91E-01 | negative regulation of endothelial cell differentiation |
| 45656 | 1.62E-01 | 2.91E-01 | negative regulation of monocyte differentiation |
| 45658 | 1.62E-01 | 2.91E-01 | regulation of neutrophil differentiation |
| 45719 | 1.62E-01 | 2.91E-01 | negative regulation of glycogen biosynthetic process |
| 45726 | 1.62E-01 | 2.91E-01 | positive regulation of integrin biosynthetic process |
| 45829 | 1.62E-01 | 2.91E-01 | negative regulation of isotype switching |
| 70427 | 1.62E-01 | 2.91E-01 | nucleotide-binding oligomerization domain containing 1 signaling pathway |
| 45875 | 1.62E-01 | 2.91E-01 | negative regulation of sister chromatid cohesion |
| 70483 | 1.62E-01 | 2.91E-01 | detection of hypoxia |
| 45915 | 1.62E-01 | 2.91E-01 | positive regulation of catecholamine metabolic process |
| 70493 | 1.62E-01 | 2.91E-01 | thrombin receptor signaling pathway |
| 45919 | 1.62E-01 | 2.91E-01 | positive regulation of cytolysis |
| 45953 | 1.62E-01 | 2.91E-01 | negative regulation of natural killer cell mediated cytotoxicity |
| 45955 | 1.62E-01 | 2.91E-01 | negative regulation of calcium ion-dependent exocytosis |
| 45964 | 1.62E-01 | 2.91E-01 | positive regulation of dopamine metabolic process |
| 70562 | 1.62E-01 | 2.91E-01 | regulation of vitamin D receptor signaling pathway |
| 70666 | 1.62E-01 | 2.91E-01 | regulation of mast cell proliferation |
| 70669 | 1.62E-01 | 2.91E-01 | response to interleukin-2 |
| 70668 | 1.62E-01 | 2.91E-01 | positive regulation of mast cell proliferation |
| 70672 | 1.62E-01 | 2.91E-01 | response to interleukin-15 |
| 46108 | 1.62E-01 | 2.91E-01 | uridine metabolic process |
| 46110 | 1.62E-01 | 2.91E-01 | xanthine metabolic process |
| 46135 | 1.62E-01 | 2.91E-01 | pyrimidine nucleoside catabolic process |
| 46137 | 1.62E-01 | 2.91E-01 | negative regulation of vitamin metabolic process |
| 21577 | 1.62E-01 | 2.91E-01 | hindbrain structural organization |
| 21589 | 1.62E-01 | 2.91E-01 | cerebellum structural organization |
| 21593 | 1.62E-01 | 2.91E-01 | rhombomere morphogenesis |
| 46185 | 1.62E-01 | 2.91E-01 | aldehyde catabolic process |
| 21658 | 1.62E-01 | 2.91E-01 | rhombomere 3 morphogenesis |
| 70836 | 1.62E-01 | 2.91E-01 | caveola assembly |
| 21823 | 1.62E-01 | 2.91E-01 | cerebral cortex tangential migration using cell-cell interactions |
| 21825 | 1.62E-01 | 2.91E-01 | substrate-dependent cerebral cortex tangential migration |
| 21827 | 1.62E-01 | 2.91E-01 | postnatal olfactory bulb interneuron migration |
| 21836 | 1.62E-01 | 2.91E-01 | chemorepulsion involved in postnatal olfactory bulb interneuron migration |
| 46439 | 1.62E-01 | 2.91E-01 | L-cysteine metabolic process |
| 30070 | 1.62E-01 | 2.91E-01 | insulin processing |
| 46476 | 1.62E-01 | 2.91E-01 | glycosylceramide biosynthetic process |
| 46485 | 1.62E-01 | 2.91E-01 | ether lipid metabolic process |
| 46533 | 1.62E-01 | 2.91E-01 | negative regulation of photoreceptor cell differentiation |
| 46544 | 1.62E-01 | 2.91E-01 | development of secondary male sexual characteristics |
| 71285 | 1.62E-01 | 2.91E-01 | cellular response to lithium ion |
| 71344 | 1.62E-01 | 2.91E-01 | diphosphate metabolic process |
| 14054 | 1.62E-01 | 2.91E-01 | positive regulation of gamma-aminobutyric acid secretion |
| 14062 | 1.62E-01 | 2.91E-01 | regulation of serotonin secretion |
| 14063 | 1.62E-01 | 2.91E-01 | negative regulation of serotonin secretion |
| 46951 | 1.62E-01 | 2.91E-01 | ketone body biosynthetic process |
| 30575 | 1.62E-01 | 2.91E-01 | nuclear body organization |
| 30578 | 1.62E-01 | 2.91E-01 | PML body organization |
| 6042 | 1.62E-01 | 2.91E-01 | glucosamine biosynthetic process |
| 6045 | 1.62E-01 | 2.91E-01 | N-acetylglucosamine biosynthetic process |
| 6048 | 1.62E-01 | 2.91E-01 | UDP-N-acetylglucosamine biosynthetic process |
| 6059 | 1.62E-01 | 2.91E-01 | hexitol metabolic process |
| 6097 | 1.62E-01 | 2.91E-01 | glyoxylate cycle |
| 71635 | 1.62E-01 | 2.91E-01 | negative regulation of transforming growth factor-beta production |
| 6116 | 1.62E-01 | 2.91E-01 | NADH oxidation |
| 30704 | 1.62E-01 | 2.91E-01 | vitelline membrane formation |
| 6184 | 1.62E-01 | 2.91E-01 | GTP catabolic process |
| 6196 | 1.62E-01 | 2.91E-01 | AMP catabolic process |
| 6212 | 1.62E-01 | 2.91E-01 | uracil catabolic process |
| 30836 | 1.62E-01 | 2.91E-01 | positive regulation of actin filament depolymerization |
| 30885 | 1.62E-01 | 2.91E-01 | regulation of myeloid dendritic cell activation |
| 30908 | 1.62E-01 | 2.91E-01 | protein splicing |
| 30913 | 1.62E-01 | 2.91E-01 | paranodal junction assembly |
| 72010 | 1.62E-01 | 2.91E-01 | glomerular epithelium development |
| 6478 | 1.62E-01 | 2.91E-01 | peptidyl-tyrosine sulfation |
| 72112 | 1.62E-01 | 2.91E-01 | glomerular visceral epithelial cell differentiation |
| 14806 | 1.62E-01 | 2.91E-01 | smooth muscle hyperplasia |
| 23032 | 1.62E-01 | 2.91E-01 | behavioral signaling |
| 6649 | 1.62E-01 | 2.91E-01 | phospholipid transfer to membrane |
| 31296 | 1.62E-01 | 2.91E-01 | B cell costimulation |
| 31335 | 1.62E-01 | 2.91E-01 | regulation of sulfur amino acid metabolic process |
| 72311 | 1.62E-01 | 2.91E-01 | glomerular epithelial cell differentiation |
| 6788 | 1.62E-01 | 2.91E-01 | heme oxidation |
| 31508 | 1.62E-01 | 2.91E-01 | centromeric heterochromatin formation |
| 6987 | 1.62E-01 | 2.91E-01 | activation of signaling protein activity involved in unfolded protein response |
| 48073 | 1.62E-01 | 2.91E-01 | regulation of eye pigmentation |
| 48165 | 1.62E-01 | 2.91E-01 | fused antrum stage |
| 7221 | 1.62E-01 | 2.91E-01 | positive regulation of transcription of Notch receptor target |
| 40019 | 1.62E-01 | 2.91E-01 | positive regulation of embryonic development |
| 48227 | 1.62E-01 | 2.91E-01 | plasma membrane to endosome transport |
| 48229 | 1.62E-01 | 2.91E-01 | gametophyte development |
| 7308 | 1.62E-01 | 2.91E-01 | oocyte construction |
| 7309 | 1.62E-01 | 2.91E-01 | oocyte axis specification |
| 48294 | 1.62E-01 | 2.91E-01 | negative regulation of isotype switching to IgE isotypes |
| 31915 | 1.62E-01 | 2.91E-01 | positive regulation of synaptic plasticity |
| 48314 | 1.62E-01 | 2.91E-01 | embryo sac morphogenesis |
| 48319 | 1.62E-01 | 2.91E-01 | axial mesoderm morphogenesis |
| 7403 | 1.62E-01 | 2.91E-01 | glial cell fate determination |
| 48378 | 1.62E-01 | 2.91E-01 | regulation of lateral mesodermal cell fate specification |
| 31999 | 1.62E-01 | 2.91E-01 | negative regulation of fatty acid beta-oxidation |
| 48389 | 1.62E-01 | 2.91E-01 | intermediate mesoderm development |
| 7497 | 1.62E-01 | 2.91E-01 | posterior midgut development |
| 7499 | 1.62E-01 | 2.91E-01 | ectoderm and mesoderm interaction |
| 15740 | 1.62E-01 | 2.91E-01 | C4-dicarboxylate transport |
| 15744 | 1.62E-01 | 2.91E-01 | succinate transport |
| 15788 | 1.62E-01 | 2.91E-01 | UDP-N-acetylglucosamine transport |
| 15801 | 1.62E-01 | 2.91E-01 | aromatic amino acid transport |
| 15808 | 1.62E-01 | 2.91E-01 | L-alanine transport |
| 7619 | 1.62E-01 | 2.91E-01 | courtship behavior |
| 48627 | 1.62E-01 | 2.91E-01 | myoblast development |
| 48659 | 1.62E-01 | 2.91E-01 | smooth muscle cell proliferation |
| 48670 | 1.62E-01 | 2.91E-01 | regulation of collateral sprouting |
| 48671 | 1.62E-01 | 2.91E-01 | negative regulation of collateral sprouting |
| 15939 | 1.62E-01 | 2.91E-01 | pantothenate metabolic process |
| 15942 | 1.62E-01 | 2.91E-01 | formate metabolic process |
| 16046 | 1.62E-01 | 2.91E-01 | detection of fungus |
| 48846 | 1.62E-01 | 2.91E-01 | axon extension involved in axon guidance |
| 16109 | 1.62E-01 | 2.91E-01 | tetraterpenoid biosynthetic process |
| 16114 | 1.62E-01 | 2.91E-01 | terpenoid biosynthetic process |
| 16117 | 1.62E-01 | 2.91E-01 | carotenoid biosynthetic process |
| 8065 | 1.62E-01 | 2.91E-01 | establishment of blood-nerve barrier |
| 90032 | 1.62E-01 | 2.91E-01 | negative regulation of steroid hormone biosynthetic process |
| 32693 | 1.62E-01 | 2.91E-01 | negative regulation of interleukin-10 production |
| 65004 | 1.63E-01 | 2.92E-01 | protein-DNA complex assembly |
| 9165 | 1.64E-01 | 2.94E-01 | nucleotide biosynthetic process |
| 42632 | 1.66E-01 | 2.96E-01 | cholesterol homeostasis |
| 30183 | 1.66E-01 | 2.96E-01 | B cell differentiation |
| 55092 | 1.66E-01 | 2.96E-01 | sterol homeostasis |
| 70727 | 1.66E-01 | 2.97E-01 | cellular macromolecule localization |
| 9084 | 1.70E-01 | 3.02E-01 | glutamine family amino acid biosynthetic process |
| 42596 | 1.70E-01 | 3.02E-01 | fear response |
| 50820 | 1.70E-01 | 3.02E-01 | positive regulation of coagulation |
| 1893 | 1.70E-01 | 3.02E-01 | maternal placenta development |
| 60048 | 1.70E-01 | 3.02E-01 | cardiac muscle contraction |
| 19438 | 1.70E-01 | 3.02E-01 | aromatic compound biosynthetic process |
| 61035 | 1.70E-01 | 3.02E-01 | regulation of cartilage development |
| 61097 | 1.70E-01 | 3.02E-01 | regulation of protein tyrosine kinase activity |
| 45806 | 1.70E-01 | 3.02E-01 | negative regulation of endocytosis |
| 30149 | 1.70E-01 | 3.02E-01 | sphingolipid catabolic process |
| 30641 | 1.70E-01 | 3.02E-01 | regulation of cellular pH |
| 31076 | 1.70E-01 | 3.02E-01 | embryonic camera-type eye development |
| 6739 | 1.70E-01 | 3.02E-01 | NADP metabolic process |
| 48645 | 1.70E-01 | 3.02E-01 | organ formation |
| 32732 | 1.70E-01 | 3.02E-01 | positive regulation of interleukin-1 production |
| 43473 | 1.70E-01 | 3.03E-01 | pigmentation |
| 1656 | 1.71E-01 | 3.04E-01 | metanephros development |
| 7205 | 1.71E-01 | 3.04E-01 | activation of protein kinase C activity by G-protein coupled receptor protein signaling pathway |
| 10553 | 1.72E-01 | 3.04E-01 | negative regulation of gene-specific transcription from RNA polymerase II promoter |
| 6334 | 1.73E-01 | 3.04E-01 | nucleosome assembly |
| 9141 | 1.73E-01 | 3.04E-01 | nucleoside triphosphate metabolic process |
| 32781 | 1.74E-01 | 3.04E-01 | positive regulation of ATPase activity |
| 8340 | 1.74E-01 | 3.04E-01 | determination of adult lifespan |
| 9163 | 1.74E-01 | 3.04E-01 | nucleoside biosynthetic process |
| 42053 | 1.74E-01 | 3.04E-01 | regulation of dopamine metabolic process |
| 42267 | 1.74E-01 | 3.04E-01 | natural killer cell mediated cytotoxicity |
| 42451 | 1.74E-01 | 3.04E-01 | purine nucleoside biosynthetic process |
| 42455 | 1.74E-01 | 3.04E-01 | ribonucleoside biosynthetic process |
| 1504 | 1.74E-01 | 3.04E-01 | neurotransmitter uptake |
| 1919 | 1.74E-01 | 3.04E-01 | regulation of receptor recycling |
| 42987 | 1.74E-01 | 3.04E-01 | amyloid precursor protein catabolic process |
| 43011 | 1.74E-01 | 3.04E-01 | myeloid dendritic cell differentiation |
| 2228 | 1.74E-01 | 3.04E-01 | natural killer cell mediated immunity |
| 35162 | 1.74E-01 | 3.04E-01 | embryonic hemopoiesis |
| 43372 | 1.74E-01 | 3.04E-01 | positive regulation of CD4-positive, alpha beta T cell differentiation |
| 10611 | 1.74E-01 | 3.04E-01 | regulation of cardiac muscle hypertrophy |
| 10874 | 1.74E-01 | 3.04E-01 | regulation of cholesterol efflux |
| 10889 | 1.74E-01 | 3.04E-01 | regulation of sequestering of triglyceride |
| 19359 | 1.74E-01 | 3.04E-01 | nicotinamide nucleotide biosynthetic process |
| 3084 | 1.74E-01 | 3.04E-01 | positive regulation of systemic arterial blood pressure |
| 44269 | 1.74E-01 | 3.04E-01 | glycerol ether catabolic process |
| 45136 | 1.74E-01 | 3.04E-01 | development of secondary sexual characteristics |
| 45176 | 1.74E-01 | 3.04E-01 | apical protein localization |
| 70207 | 1.74E-01 | 3.04E-01 | protein homotrimerization |
| 70229 | 1.74E-01 | 3.04E-01 | negative regulation of lymphocyte apoptosis |
| 45684 | 1.74E-01 | 3.04E-01 | positive regulation of epidermis development |
| 46129 | 1.74E-01 | 3.04E-01 | purine ribonucleoside biosynthetic process |
| 21756 | 1.74E-01 | 3.04E-01 | striatum development |
| 46461 | 1.74E-01 | 3.04E-01 | neutral lipid catabolic process |
| 46464 | 1.74E-01 | 3.04E-01 | acylglycerol catabolic process |
| 46653 | 1.74E-01 | 3.04E-01 | tetrahydrofolate metabolic process |
| 30857 | 1.74E-01 | 3.04E-01 | negative regulation of epithelial cell differentiation |
| 6734 | 1.74E-01 | 3.04E-01 | NADH metabolic process |
| 31638 | 1.74E-01 | 3.04E-01 | zymogen activation |
| 48486 | 1.74E-01 | 3.04E-01 | parasympathetic nervous system development |
| 32373 | 1.74E-01 | 3.04E-01 | positive regulation of sterol transport |
| 32376 | 1.74E-01 | 3.04E-01 | positive regulation of cholesterol transport |
| 9161 | 1.74E-01 | 3.04E-01 | ribonucleoside monophosphate metabolic process |
| 42490 | 1.74E-01 | 3.04E-01 | mechanoreceptor differentiation |
| 30834 | 1.74E-01 | 3.04E-01 | regulation of actin filament depolymerization |
| 7173 | 1.74E-01 | 3.04E-01 | epidermal growth factor receptor signaling pathway |
| 32652 | 1.74E-01 | 3.04E-01 | regulation of interleukin-1 production |
| 90090 | 1.74E-01 | 3.04E-01 | negative regulation of canonical Wnt receptor signaling pathway |
| 45165 | 1.77E-01 | 3.09E-01 | cell fate commitment |
| 30509 | 1.78E-01 | 3.11E-01 | BMP signaling pathway |
| 7605 | 1.80E-01 | 3.14E-01 | sensory perception of sound |
| 51092 | 1.82E-01 | 3.17E-01 | positive regulation of NF-kappaB transcription factor activity |
| 6898 | 1.82E-01 | 3.17E-01 | receptor-mediated endocytosis |
| 32869 | 1.83E-01 | 3.19E-01 | cellular response to insulin stimulus |
| 30258 | 1.83E-01 | 3.19E-01 | lipid modification |
| 9144 | 1.83E-01 | 3.19E-01 | purine nucleoside triphosphate metabolic process |
| 31400 | 1.84E-01 | 3.21E-01 | negative regulation of protein modification process |
| 46425 | 1.86E-01 | 3.23E-01 | regulation of JAK-STAT cascade |
| 31214 | 1.86E-01 | 3.23E-01 | biomineral formation |
| 6970 | 1.86E-01 | 3.23E-01 | response to osmotic stress |
| 10557 | 1.86E-01 | 3.24E-01 | positive regulation of macromolecule biosynthetic process |
| 6605 | 1.87E-01 | 3.24E-01 | protein targeting |
| 51146 | 1.91E-01 | 3.30E-01 | striated muscle cell differentiation |
| 51606 | 1.91E-01 | 3.30E-01 | detection of stimulus |
| 14075 | 1.91E-01 | 3.30E-01 | response to amine stimulus |
| 46887 | 1.91E-01 | 3.30E-01 | positive regulation of hormone secretion |
| 60415 | 1.92E-01 | 3.30E-01 | muscle tissue morphogenesis |
| 44243 | 1.92E-01 | 3.30E-01 | multicellular organismal catabolic process |
| 46427 | 1.92E-01 | 3.30E-01 | positive regulation of JAK-STAT cascade |
| 30166 | 1.92E-01 | 3.30E-01 | proteoglycan biosynthetic process |
| 14013 | 1.92E-01 | 3.30E-01 | regulation of gliogenesis |
| 55008 | 1.92E-01 | 3.30E-01 | cardiac muscle tissue morphogenesis |
| 7632 | 1.92E-01 | 3.30E-01 | visual behavior |
| 42116 | 1.92E-01 | 3.30E-01 | macrophage activation |
| 42269 | 1.92E-01 | 3.30E-01 | regulation of natural killer cell mediated cytotoxicity |
| 1578 | 1.92E-01 | 3.30E-01 | microtubule bundle formation |
| 1912 | 1.92E-01 | 3.30E-01 | positive regulation of leukocyte mediated cytotoxicity |
| 42993 | 1.92E-01 | 3.30E-01 | positive regulation of transcription factor import into nucleus |
| 43029 | 1.92E-01 | 3.30E-01 | T cell homeostasis |
| 35265 | 1.92E-01 | 3.30E-01 | organ growth |
| 2715 | 1.92E-01 | 3.30E-01 | regulation of natural killer cell mediated immunity |
| 10907 | 1.92E-01 | 3.30E-01 | positive regulation of glucose metabolic process |
| 2718 | 1.92E-01 | 3.30E-01 | regulation of cytokine production involved in immune response |
| 45987 | 1.92E-01 | 3.30E-01 | positive regulation of smooth muscle contraction |
| 46466 | 1.92E-01 | 3.30E-01 | membrane lipid catabolic process |
| 6020 | 1.92E-01 | 3.30E-01 | inositol metabolic process |
| 6516 | 1.92E-01 | 3.30E-01 | glycoprotein catabolic process |
| 31623 | 1.92E-01 | 3.30E-01 | receptor internalization |
| 7090 | 1.92E-01 | 3.30E-01 | regulation of S phase of mitotic cell cycle |
| 7212 | 1.92E-01 | 3.30E-01 | dopamine receptor signaling pathway |
| 7528 | 1.92E-01 | 3.30E-01 | neuromuscular junction development |
| 32410 | 1.92E-01 | 3.30E-01 | negative regulation of transporter activity |
| 51173 | 1.93E-01 | 3.32E-01 | positive regulation of nitrogen compound metabolic process |
| 48469 | 1.93E-01 | 3.32E-01 | cell maturation |
| 42440 | 1.94E-01 | 3.33E-01 | pigment metabolic process |
| 45444 | 1.94E-01 | 3.33E-01 | fat cell differentiation |
| 42471 | 1.94E-01 | 3.33E-01 | ear morphogenesis |
| 51651 | 1.94E-01 | 3.33E-01 | maintenance of location in cell |
| 45944 | 1.96E-01 | 3.35E-01 | positive regulation of transcription from RNA polymerase II promoter |
| 6091 | 2.00E-01 | 3.44E-01 | generation of precursor metabolites and energy |
| 33013 | 2.01E-01 | 3.44E-01 | tetrapyrrole metabolic process |
| 43112 | 2.01E-01 | 3.44E-01 | receptor metabolic process |
| 10906 | 2.01E-01 | 3.44E-01 | regulation of glucose metabolic process |
| 45639 | 2.01E-01 | 3.44E-01 | positive regulation of myeloid cell differentiation |
| 22600 | 2.01E-01 | 3.44E-01 | digestive system process |
| 6778 | 2.01E-01 | 3.44E-01 | porphyrin metabolic process |
| 8624 | 2.03E-01 | 3.47E-01 | induction of apoptosis by extracellular signals |
| 30900 | 2.05E-01 | 3.47E-01 | forebrain development |
| 50714 | 2.05E-01 | 3.47E-01 | positive regulation of protein secretion |
| 72175 | 2.05E-01 | 3.47E-01 | epithelial tube formation |
| 15718 | 2.05E-01 | 3.47E-01 | monocarboxylic acid transport |
| 16558 | 2.06E-01 | 3.47E-01 | protein import into peroxisome matrix |
| 42069 | 2.06E-01 | 3.47E-01 | regulation of catecholamine metabolic process |
| 50853 | 2.06E-01 | 3.47E-01 | B cell receptor signaling pathway |
| 1773 | 2.06E-01 | 3.47E-01 | myeloid dendritic cell activation |
| 1774 | 2.06E-01 | 3.47E-01 | microglial cell activation |
| 18196 | 2.06E-01 | 3.47E-01 | peptidyl-asparagine modification |
| 18279 | 2.06E-01 | 3.47E-01 | protein amino acid N-linked glycosylation via asparagine |
| 43124 | 2.06E-01 | 3.47E-01 | negative regulation of I-kappaB kinase/NF-kappaB cascade |
| 43537 | 2.06E-01 | 3.47E-01 | negative regulation of blood vessel endothelial cell migration |
| 43584 | 2.06E-01 | 3.47E-01 | nose development |
| 51806 | 2.06E-01 | 3.47E-01 | entry into cell of other organism involved in symbiotic interaction |
| 51828 | 2.06E-01 | 3.47E-01 | entry into other organism involved in symbiotic interaction |
| 52126 | 2.06E-01 | 3.47E-01 | movement in host environment |
| 52192 | 2.06E-01 | 3.47E-01 | movement in environment of other organism involved in symbiotic interaction |
| 60416 | 2.06E-01 | 3.47E-01 | response to growth hormone stimulus |
| 60602 | 2.06E-01 | 3.47E-01 | branch elongation of an epithelium |
| 3401 | 2.06E-01 | 3.47E-01 | axis elongation |
| 3407 | 2.06E-01 | 3.47E-01 | neural retina development |
| 44409 | 2.06E-01 | 3.47E-01 | entry into host |
| 45649 | 2.06E-01 | 3.47E-01 | regulation of macrophage differentiation |
| 30002 | 2.06E-01 | 3.47E-01 | cellular anion homeostasis |
| 30260 | 2.06E-01 | 3.47E-01 | entry into host cell |
| 46718 | 2.06E-01 | 3.47E-01 | entry of virus into host cell |
| 22408 | 2.06E-01 | 3.47E-01 | negative regulation of cell-cell adhesion |
| 14743 | 2.06E-01 | 3.47E-01 | regulation of muscle hypertrophy |
| 14911 | 2.06E-01 | 3.47E-01 | positive regulation of smooth muscle cell migration |
| 7143 | 2.06E-01 | 3.47E-01 | female meiosis |
| 7213 | 2.06E-01 | 3.47E-01 | muscarinic acetylcholine receptor signaling pathway |
| 48200 | 2.06E-01 | 3.47E-01 | Golgi transport vesicle coating |
| 48205 | 2.06E-01 | 3.47E-01 | COPI coating of Golgi vesicle |
| 48246 | 2.06E-01 | 3.47E-01 | macrophage chemotaxis |
| 7440 | 2.06E-01 | 3.47E-01 | foregut morphogenesis |
| 32094 | 2.06E-01 | 3.47E-01 | response to food |
| 7567 | 2.06E-01 | 3.47E-01 | parturition |
| 32757 | 2.06E-01 | 3.47E-01 | positive regulation of interleukin-8 production |
| 71103 | 2.07E-01 | 3.49E-01 | DNA conformation change |
| 6323 | 2.08E-01 | 3.50E-01 | DNA packaging |
| 42168 | 2.10E-01 | 3.53E-01 | heme metabolic process |
| 45598 | 2.10E-01 | 3.53E-01 | regulation of fat cell differentiation |
| 6752 | 2.10E-01 | 3.53E-01 | group transfer coenzyme metabolic process |
| 46164 | 2.14E-01 | 3.60E-01 | alcohol catabolic process |
| 33143 | 2.15E-01 | 3.60E-01 | regulation of steroid hormone receptor signaling pathway |
| 9126 | 2.15E-01 | 3.60E-01 | purine nucleoside monophosphate metabolic process |
| 9167 | 2.15E-01 | 3.60E-01 | purine ribonucleoside monophosphate metabolic process |
| 1825 | 2.15E-01 | 3.60E-01 | blastocyst formation |
| 60560 | 2.15E-01 | 3.60E-01 | developmental growth involved in morphogenesis |
| 45055 | 2.15E-01 | 3.60E-01 | regulated secretory pathway |
| 45104 | 2.15E-01 | 3.60E-01 | intermediate filament cytoskeleton organization |
| 30101 | 2.15E-01 | 3.60E-01 | natural killer cell activation |
| 30177 | 2.15E-01 | 3.60E-01 | positive regulation of Wnt receptor signaling pathway |
| 46579 | 2.15E-01 | 3.60E-01 | positive regulation of Ras protein signal transduction |
| 46631 | 2.15E-01 | 3.60E-01 | alpha-beta T cell activation |
| 31069 | 2.15E-01 | 3.60E-01 | hair follicle morphogenesis |
| 6900 | 2.15E-01 | 3.60E-01 | membrane budding |
| 16032 | 2.17E-01 | 3.61E-01 | viral reproduction |
| 1824 | 2.17E-01 | 3.61E-01 | blastocyst development |
| 60041 | 2.17E-01 | 3.61E-01 | retina development in camera-type eye |
| 3073 | 2.17E-01 | 3.61E-01 | regulation of systemic arterial blood pressure |
| 6941 | 2.17E-01 | 3.61E-01 | striated muscle contraction |
| 30902 | 2.18E-01 | 3.61E-01 | hindbrain development |
| 6639 | 2.19E-01 | 3.61E-01 | acylglycerol metabolic process |
| 44419 | 2.21E-01 | 3.61E-01 | interspecies interaction between organisms |
| 90277 | 2.28E-01 | 3.61E-01 | positive regulation of peptide hormone secretion |
| 45981 | 2.28E-01 | 3.61E-01 | positive regulation of nucleotide metabolic process |
| 30801 | 2.28E-01 | 3.61E-01 | positive regulation of cyclic nucleotide metabolic process |
| 30804 | 2.28E-01 | 3.61E-01 | positive regulation of cyclic nucleotide biosynthetic process |
| 30810 | 2.28E-01 | 3.61E-01 | positive regulation of nucleotide biosynthetic process |
| 6776 | 2.28E-01 | 3.61E-01 | vitamin A metabolic process |
| 31670 | 2.28E-01 | 3.61E-01 | cellular response to nutrient |
| 15992 | 2.31E-01 | 3.61E-01 | proton transport |
| 50821 | 2.33E-01 | 3.61E-01 | protein stabilization |
| 51701 | 2.33E-01 | 3.61E-01 | interaction with host |
| 6775 | 2.33E-01 | 3.61E-01 | fat-soluble vitamin metabolic process |
| 48477 | 2.33E-01 | 3.61E-01 | oogenesis |
| 48663 | 2.33E-01 | 3.61E-01 | neuron fate commitment |
| 45089 | 2.33E-01 | 3.61E-01 | positive regulation of innate immune response |
| 32823 | 2.33E-01 | 3.61E-01 | regulation of natural killer cell differentiation |
| 32825 | 2.33E-01 | 3.61E-01 | positive regulation of natural killer cell differentiation |
| 90205 | 2.33E-01 | 3.61E-01 | positive regulation of cholesterol metabolic process |
| 90206 | 2.33E-01 | 3.61E-01 | negative regulation of cholesterol metabolic process |
| 8295 | 2.33E-01 | 3.61E-01 | spermidine biosynthetic process |
| 32914 | 2.33E-01 | 3.61E-01 | positive regulation of transforming growth factor-beta1 production |
| 90279 | 2.33E-01 | 3.61E-01 | regulation of calcium ion import |
| 16559 | 2.33E-01 | 3.61E-01 | peroxisome fission |
| 90292 | 2.33E-01 | 3.61E-01 | nuclear matrix anchoring at nuclear membrane |
| 32971 | 2.33E-01 | 3.61E-01 | regulation of muscle filament sliding |
| 33025 | 2.33E-01 | 3.61E-01 | regulation of mast cell apoptosis |
| 301 | 2.33E-01 | 3.61E-01 | retrograde transport, vesicle recycling within Golgi |
| 33089 | 2.33E-01 | 3.61E-01 | positive regulation of T cell differentiation in the thymus |
| 8614 | 2.33E-01 | 3.61E-01 | pyridoxine metabolic process |
| 8615 | 2.33E-01 | 3.61E-01 | pyridoxine biosynthetic process |
| 16999 | 2.33E-01 | 3.61E-01 | antibiotic metabolic process |
| 17000 | 2.33E-01 | 3.61E-01 | antibiotic biosynthetic process |
| 17004 | 2.33E-01 | 3.61E-01 | cytochrome complex assembly |
| 33563 | 2.33E-01 | 3.61E-01 | dorsal/ventral axon guidance |
| 33629 | 2.33E-01 | 3.61E-01 | negative regulation of cell adhesion mediated by integrin |
| 9173 | 2.33E-01 | 3.61E-01 | pyrimidine ribonucleoside monophosphate metabolic process |
| 9174 | 2.33E-01 | 3.61E-01 | pyrimidine ribonucleoside monophosphate biosynthetic process |
| 42271 | 2.33E-01 | 3.61E-01 | susceptibility to natural killer cell mediated cytotoxicity |
| 34114 | 2.33E-01 | 3.61E-01 | regulation of heterotypic cell-cell adhesion |
| 34122 | 2.33E-01 | 3.61E-01 | negative regulation of toll-like receptor signaling pathway |
| 42396 | 2.33E-01 | 3.61E-01 | phosphagen biosynthetic process |
| 50685 | 2.33E-01 | 3.61E-01 | positive regulation of mRNA processing |
| 1543 | 2.33E-01 | 3.61E-01 | ovarian follicle rupture |
| 42508 | 2.33E-01 | 3.61E-01 | tyrosine phosphorylation of Stat1 protein |
| 42510 | 2.33E-01 | 3.61E-01 | regulation of tyrosine phosphorylation of Stat1 protein |
| 42511 | 2.33E-01 | 3.61E-01 | positive regulation of tyrosine phosphorylation of Stat1 protein |
| 50705 | 2.33E-01 | 3.61E-01 | regulation of interleukin-1 alpha secretion |
| 50717 | 2.33E-01 | 3.61E-01 | positive regulation of interleukin-1 alpha secretion |
| 34371 | 2.33E-01 | 3.61E-01 | chylomicron remodeling |
| 50765 | 2.33E-01 | 3.61E-01 | negative regulation of phagocytosis |
| 34433 | 2.33E-01 | 3.61E-01 | steroid esterification |
| 34434 | 2.33E-01 | 3.61E-01 | sterol esterification |
| 34435 | 2.33E-01 | 3.61E-01 | cholesterol esterification |
| 50862 | 2.33E-01 | 3.61E-01 | positive regulation of T cell receptor signaling pathway |
| 34505 | 2.33E-01 | 3.61E-01 | tooth mineralization |
| 50923 | 2.33E-01 | 3.61E-01 | regulation of negative chemotaxis |
| 42732 | 2.33E-01 | 3.61E-01 | D-xylose metabolic process |
| 50942 | 2.33E-01 | 3.61E-01 | positive regulation of pigment cell differentiation |
| 50955 | 2.33E-01 | 3.61E-01 | thermoception |
| 42816 | 2.33E-01 | 3.61E-01 | vitamin B6 metabolic process |
| 42819 | 2.33E-01 | 3.61E-01 | vitamin B6 biosynthetic process |
| 42866 | 2.33E-01 | 3.61E-01 | pyruvate biosynthetic process |
| 10107 | 2.33E-01 | 3.61E-01 | potassium ion import |
| 51088 | 2.33E-01 | 3.61E-01 | PMA-inducible membrane protein ectodomain proteolysis |
| 10159 | 2.33E-01 | 3.61E-01 | specification of organ position |
| 1973 | 2.33E-01 | 3.61E-01 | adenosine receptor signaling pathway |
| 51136 | 2.33E-01 | 3.61E-01 | regulation of NK T cell differentiation |
| 51138 | 2.33E-01 | 3.61E-01 | positive regulation of NK T cell differentiation |
| 1996 | 2.33E-01 | 3.61E-01 | positive regulation of heart rate by epinephrine-norepinephrine |
| 42977 | 2.33E-01 | 3.61E-01 | activation of JAK2 kinase activity |
| 18401 | 2.33E-01 | 3.61E-01 | peptidyl-proline hydroxylation to 4-hydroxy-L-proline |
| 10216 | 2.33E-01 | 3.61E-01 | maintenance of DNA methylation |
| 51176 | 2.33E-01 | 3.61E-01 | positive regulation of sulfur metabolic process |
| 2024 | 2.33E-01 | 3.61E-01 | diet induced thermogenesis |
| 2034 | 2.33E-01 | 3.61E-01 | regulation of blood vessel size by renin-angiotensin |
| 2035 | 2.33E-01 | 3.61E-01 | brain renin-angiotensin system |
| 2066 | 2.33E-01 | 3.61E-01 | columnar/cuboidal epithelial cell development |
| 2072 | 2.33E-01 | 3.61E-01 | optic cup morphogenesis involved in camera-type eye development |
| 43045 | 2.33E-01 | 3.61E-01 | DNA methylation involved in embryonic development |
| 2238 | 2.33E-01 | 3.61E-01 | response to molecule of fungal origin |
| 10459 | 2.33E-01 | 3.61E-01 | negative regulation of heart rate |
| 35067 | 2.33E-01 | 3.61E-01 | negative regulation of histone acetylation |
| 2318 | 2.33E-01 | 3.61E-01 | myeloid progenitor cell differentiation |
| 51497 | 2.33E-01 | 3.61E-01 | negative regulation of stress fiber assembly |
| 10633 | 2.33E-01 | 3.61E-01 | negative regulation of epithelial cell migration |
| 10662 | 2.33E-01 | 3.61E-01 | regulation of striated muscle cell apoptosis |
| 10664 | 2.33E-01 | 3.61E-01 | negative regulation of striated muscle cell apoptosis |
| 10665 | 2.33E-01 | 3.61E-01 | regulation of cardiac muscle cell apoptosis |
| 10667 | 2.33E-01 | 3.61E-01 | negative regulation of cardiac muscle cell apoptosis |
| 10692 | 2.33E-01 | 3.61E-01 | regulation of alkaline phosphatase activity |
| 43476 | 2.33E-01 | 3.61E-01 | pigment accumulation |
| 10715 | 2.33E-01 | 3.61E-01 | regulation of extracellular matrix disassembly |
| 10716 | 2.33E-01 | 3.61E-01 | negative regulation of extracellular matrix disassembly |
| 43490 | 2.33E-01 | 3.61E-01 | malate-aspartate shuttle |
| 10742 | 2.33E-01 | 3.61E-01 | macrophage derived foam cell differentiation |
| 51712 | 2.33E-01 | 3.61E-01 | positive regulation of killing of cells of another organism |
| 2576 | 2.33E-01 | 3.61E-01 | platelet degranulation |
| 10771 | 2.33E-01 | 3.61E-01 | negative regulation of cell morphogenesis involved in differentiation |
| 10800 | 2.33E-01 | 3.61E-01 | positive regulation of peptidyl-threonine phosphorylation |
| 43578 | 2.33E-01 | 3.61E-01 | nuclear matrix organization |
| 51772 | 2.33E-01 | 3.61E-01 | regulation of nitric-oxide synthase 2 biosynthetic process |
| 2636 | 2.33E-01 | 3.61E-01 | positive regulation of germinal center formation |
| 60013 | 2.33E-01 | 3.61E-01 | righting reflex |
| 60014 | 2.33E-01 | 3.61E-01 | granulosa cell differentiation |
| 60026 | 2.33E-01 | 3.61E-01 | convergent extension |
| 2689 | 2.33E-01 | 3.61E-01 | negative regulation of leukocyte chemotaxis |
| 51877 | 2.33E-01 | 3.61E-01 | pigment granule aggregation in cell center |
| 60087 | 2.33E-01 | 3.61E-01 | relaxation of vascular smooth muscle |
| 10944 | 2.33E-01 | 3.61E-01 | negative regulation of transcription by competitive promoter binding |
| 10956 | 2.33E-01 | 3.61E-01 | negative regulation of calcidiol 1-monooxygenase activity |
| 60137 | 2.33E-01 | 3.61E-01 | maternal process involved in parturition |
| 2827 | 2.33E-01 | 3.61E-01 | positive regulation of T-helper 1 type immune response |
| 60197 | 2.33E-01 | 3.61E-01 | cloacal septation |
| 60218 | 2.33E-01 | 3.61E-01 | hemopoietic stem cell differentiation |
| 19276 | 2.33E-01 | 3.61E-01 | UDP-N-acetylgalactosamine metabolic process |
| 60261 | 2.33E-01 | 3.61E-01 | positive regulation of transcription initiation from RNA polymerase II promoter |
| 60272 | 2.33E-01 | 3.61E-01 | embryonic skeletal joint morphogenesis |
| 43902 | 2.33E-01 | 3.61E-01 | positive regulation of multi-organism process |
| 60295 | 2.33E-01 | 3.61E-01 | regulation of cilium movement involved in cell motility |
| 60296 | 2.33E-01 | 3.61E-01 | regulation of cilium beat frequency involved in ciliary motility |
| 19371 | 2.33E-01 | 3.61E-01 | cyclooxygenase pathway |
| 60394 | 2.33E-01 | 3.61E-01 | negative regulation of pathway-restricted SMAD protein phosphorylation |
| 60398 | 2.33E-01 | 3.61E-01 | regulation of growth hormone receptor signaling pathway |
| 3072 | 2.33E-01 | 3.61E-01 | renal control of peripheral vascular resistance involved in regulation of systemic arterial blood pressure |
| 19471 | 2.33E-01 | 3.61E-01 | 4-hydroxyproline metabolic process |
| 60435 | 2.33E-01 | 3.61E-01 | bronchiole development |
| 60437 | 2.33E-01 | 3.61E-01 | lung growth |
| 3099 | 2.33E-01 | 3.61E-01 | positive regulation of the force of heart contraction by chemical signal |
| 19511 | 2.33E-01 | 3.61E-01 | peptidyl-proline hydroxylation |
| 3129 | 2.33E-01 | 3.61E-01 | heart induction |
| 3133 | 2.33E-01 | 3.61E-01 | endodermal-mesodermal cell signaling |
| 3134 | 2.33E-01 | 3.61E-01 | endodermal-mesodermal cell signaling involved in heart induction |
| 60559 | 2.33E-01 | 3.61E-01 | positive regulation of calcidiol 1-monooxygenase activity |
| 60586 | 2.33E-01 | 3.61E-01 | multicellular organismal iron ion homeostasis |
| 60686 | 2.33E-01 | 3.61E-01 | negative regulation of prostatic bud formation |
| 3352 | 2.33E-01 | 3.61E-01 | regulation of cilium movement |
| 60708 | 2.33E-01 | 3.61E-01 | spongiotrophoblast differentiation |
| 19805 | 2.33E-01 | 3.61E-01 | quinolinate biosynthetic process |
| 19859 | 2.33E-01 | 3.61E-01 | thymine metabolic process |
| 60914 | 2.33E-01 | 3.61E-01 | heart formation |
| 60998 | 2.33E-01 | 3.61E-01 | regulation of dendritic spine development |
| 61001 | 2.33E-01 | 3.61E-01 | regulation of dendritic spine morphogenesis |
| 45074 | 2.33E-01 | 3.61E-01 | regulation of interleukin-10 biosynthetic process |
| 45077 | 2.33E-01 | 3.61E-01 | negative regulation of interferon-gamma biosynthetic process |
| 45113 | 2.33E-01 | 3.61E-01 | regulation of integrin biosynthetic process |
| 45329 | 2.33E-01 | 3.61E-01 | carnitine biosynthetic process |
| 45475 | 2.33E-01 | 3.61E-01 | locomotor rhythm |
| 70092 | 2.33E-01 | 3.61E-01 | regulation of glucagon secretion |
| 45541 | 2.33E-01 | 3.61E-01 | negative regulation of cholesterol biosynthetic process |
| 45578 | 2.33E-01 | 3.61E-01 | negative regulation of B cell differentiation |
| 70163 | 2.33E-01 | 3.61E-01 | regulation of adiponectin secretion |
| 45629 | 2.33E-01 | 3.61E-01 | negative regulation of T-helper 2 cell differentiation |
| 45630 | 2.33E-01 | 3.61E-01 | positive regulation of T-helper 2 cell differentiation |
| 45636 | 2.33E-01 | 3.61E-01 | positive regulation of melanocyte differentiation |
| 45654 | 2.33E-01 | 3.61E-01 | positive regulation of megakaryocyte differentiation |
| 70255 | 2.33E-01 | 3.61E-01 | regulation of mucus secretion |
| 45896 | 2.33E-01 | 3.61E-01 | regulation of transcription, mitotic |
| 45897 | 2.33E-01 | 3.61E-01 | positive regulation of transcription, mitotic |
| 70574 | 2.33E-01 | 3.61E-01 | cadmium ion transmembrane transport |
| 46007 | 2.33E-01 | 3.61E-01 | negative regulation of activated T cell proliferation |
| 70587 | 2.33E-01 | 3.61E-01 | regulation of cell-cell adhesion involved in gastrulation |
| 46021 | 2.33E-01 | 3.61E-01 | regulation of transcription from RNA polymerase II promoter, mitotic |
| 46022 | 2.33E-01 | 3.61E-01 | positive regulation of transcription from RNA polymerase II promoter, mitotic |
| 46049 | 2.33E-01 | 3.61E-01 | UMP metabolic process |
| 70634 | 2.33E-01 | 3.61E-01 | transepithelial ammonium transport |
| 46085 | 2.33E-01 | 3.61E-01 | adenosine metabolic process |
| 70670 | 2.33E-01 | 3.61E-01 | response to interleukin-4 |
| 46121 | 2.33E-01 | 3.61E-01 | deoxyribonucleoside catabolic process |
| 70777 | 2.33E-01 | 3.61E-01 | D-aspartate transport |
| 70779 | 2.33E-01 | 3.61E-01 | D-aspartate import |
| 70828 | 2.33E-01 | 3.61E-01 | heterochromatin organization |
| 46323 | 2.33E-01 | 3.61E-01 | glucose import |
| 46349 | 2.33E-01 | 3.61E-01 | amino sugar biosynthetic process |
| 46449 | 2.33E-01 | 3.61E-01 | creatinine metabolic process |
| 46548 | 2.33E-01 | 3.61E-01 | retinal rod cell development |
| 30210 | 2.33E-01 | 3.61E-01 | heparin biosynthetic process |
| 46628 | 2.33E-01 | 3.61E-01 | positive regulation of insulin receptor signaling pathway |
| 46633 | 2.33E-01 | 3.61E-01 | alpha-beta T cell proliferation |
| 30320 | 2.33E-01 | 3.61E-01 | cellular monovalent inorganic anion homeostasis |
| 71318 | 2.33E-01 | 3.61E-01 | cellular response to ATP |
| 46813 | 2.33E-01 | 3.61E-01 | virion attachment, binding of host cell surface receptor |
| 71407 | 2.33E-01 | 3.61E-01 | cellular response to organic cyclic substance |
| 55064 | 2.33E-01 | 3.61E-01 | chloride ion homeostasis |
| 46877 | 2.33E-01 | 3.61E-01 | regulation of saliva secretion |
| 55083 | 2.33E-01 | 3.61E-01 | monovalent inorganic anion homeostasis |
| 46931 | 2.33E-01 | 3.61E-01 | pore complex assembly |
| 30644 | 2.33E-01 | 3.61E-01 | cellular chloride ion homeostasis |
| 6085 | 2.33E-01 | 3.61E-01 | acetyl-CoA biosynthetic process |
| 6208 | 2.33E-01 | 3.61E-01 | pyrimidine base catabolic process |
| 6210 | 2.33E-01 | 3.61E-01 | thymine catabolic process |
| 6222 | 2.33E-01 | 3.61E-01 | UMP biosynthetic process |
| 30948 | 2.33E-01 | 3.61E-01 | negative regulation of vascular endothelial growth factor receptor signaling pathway |
| 31055 | 2.33E-01 | 3.61E-01 | chromatin remodeling at centromere |
| 31077 | 2.33E-01 | 3.61E-01 | post-embryonic camera-type eye development |
| 6521 | 2.33E-01 | 3.61E-01 | regulation of cellular amino acid metabolic process |
| 6528 | 2.33E-01 | 3.61E-01 | asparagine metabolic process |
| 31115 | 2.33E-01 | 3.61E-01 | negative regulation of microtubule polymerization |
| 31133 | 2.33E-01 | 3.61E-01 | regulation of axon diameter |
| 6565 | 2.33E-01 | 3.61E-01 | L-serine catabolic process |
| 14874 | 2.33E-01 | 3.61E-01 | response to stimulus involved in regulation of muscle adaptation |
| 31442 | 2.33E-01 | 3.61E-01 | positive regulation of mRNA 3'-end processing |
| 6922 | 2.33E-01 | 3.61E-01 | cleavage of lamin |
| 6923 | 2.33E-01 | 3.61E-01 | cleavage of cytoskeletal proteins involved in apoptosis |
| 6926 | 2.33E-01 | 3.61E-01 | virus-infected cell apoptosis |
| 6930 | 2.33E-01 | 3.61E-01 | substrate-bound cell migration, cell extension |
| 31507 | 2.33E-01 | 3.61E-01 | heterochromatin formation |
| 7262 | 2.33E-01 | 3.61E-01 | STAT protein nuclear translocation |
| 48305 | 2.33E-01 | 3.61E-01 | immunoglobulin secretion |
| 31943 | 2.33E-01 | 3.61E-01 | regulation of glucocorticoid metabolic process |
| 48388 | 2.33E-01 | 3.61E-01 | endosomal lumen acidification |
| 32060 | 2.33E-01 | 3.61E-01 | bleb assembly |
| 15691 | 2.33E-01 | 3.61E-01 | cadmium ion transport |
| 32075 | 2.33E-01 | 3.61E-01 | positive regulation of nuclease activity |
| 7509 | 2.33E-01 | 3.61E-01 | mesoderm migration |
| 48552 | 2.33E-01 | 3.61E-01 | regulation of metalloenzyme activity |
| 48554 | 2.33E-01 | 3.61E-01 | positive regulation of metalloenzyme activity |
| 7614 | 2.33E-01 | 3.61E-01 | short-term memory |
| 15865 | 2.33E-01 | 3.61E-01 | purine nucleotide transport |
| 15868 | 2.33E-01 | 3.61E-01 | purine ribonucleotide transport |
| 48681 | 2.33E-01 | 3.61E-01 | negative regulation of axon regeneration |
| 15917 | 2.33E-01 | 3.61E-01 | aminophospholipid transport |
| 48702 | 2.33E-01 | 3.61E-01 | embryonic neurocranium morphogenesis |
| 48711 | 2.33E-01 | 3.61E-01 | positive regulation of astrocyte differentiation |
| 32328 | 2.33E-01 | 3.61E-01 | alanine transport |
| 32351 | 2.33E-01 | 3.61E-01 | negative regulation of hormone metabolic process |
| 32353 | 2.33E-01 | 3.61E-01 | negative regulation of hormone biosynthetic process |
| 32354 | 2.33E-01 | 3.61E-01 | response to follicle-stimulating hormone stimulus |
| 16108 | 2.33E-01 | 3.61E-01 | tetraterpenoid metabolic process |
| 32497 | 2.33E-01 | 3.61E-01 | detection of lipopolysaccharide |
| 16116 | 2.33E-01 | 3.61E-01 | carotenoid metabolic process |
| 32509 | 2.33E-01 | 3.61E-01 | endosome transport via multivesicular body sorting pathway |
| 32536 | 2.33E-01 | 3.61E-01 | regulation of cell projection size |
| 8090 | 2.33E-01 | 3.61E-01 | retrograde axon cargo transport |
| 90023 | 2.33E-01 | 3.61E-01 | positive regulation of neutrophil chemotaxis |
| 90077 | 2.33E-01 | 3.61E-01 | foam cell differentiation |
| 90084 | 2.33E-01 | 3.61E-01 | negative regulation of inclusion body assembly |
| 32753 | 2.33E-01 | 3.61E-01 | positive regulation of interleukin-4 production |
| 8206 | 2.38E-01 | 3.63E-01 | bile acid metabolic process |
| 32890 | 2.38E-01 | 3.63E-01 | regulation of organic acid transport |
| 50654 | 2.38E-01 | 3.63E-01 | chondroitin sulfate proteoglycan metabolic process |
| 1754 | 2.38E-01 | 3.63E-01 | eye photoreceptor cell differentiation |
| 1755 | 2.38E-01 | 3.63E-01 | neural crest cell migration |
| 51057 | 2.38E-01 | 3.63E-01 | positive regulation of small GTPase mediated signal transduction |
| 48742 | 2.38E-01 | 3.63E-01 | regulation of skeletal muscle fiber development |
| 8154 | 2.38E-01 | 3.63E-01 | actin polymerization or depolymerization |
| 32892 | 2.38E-01 | 3.63E-01 | positive regulation of organic acid transport |
| 33081 | 2.38E-01 | 3.63E-01 | regulation of T cell differentiation in the thymus |
| 42517 | 2.38E-01 | 3.63E-01 | positive regulation of tyrosine phosphorylation of Stat3 protein |
| 50829 | 2.38E-01 | 3.63E-01 | defense response to Gram-negative bacterium |
| 50974 | 2.38E-01 | 3.63E-01 | detection of mechanical stimulus involved in sensory perception |
| 51150 | 2.38E-01 | 3.63E-01 | regulation of smooth muscle cell differentiation |
| 42982 | 2.38E-01 | 3.63E-01 | amyloid precursor protein metabolic process |
| 43030 | 2.38E-01 | 3.63E-01 | regulation of macrophage activation |
| 51281 | 2.38E-01 | 3.63E-01 | positive regulation of release of sequestered calcium ion into cytosol |
| 43330 | 2.38E-01 | 3.63E-01 | response to exogenous dsRNA |
| 43403 | 2.38E-01 | 3.63E-01 | skeletal muscle tissue regeneration |
| 10677 | 2.38E-01 | 3.63E-01 | negative regulation of cellular carbohydrate metabolic process |
| 60428 | 2.38E-01 | 3.63E-01 | lung epithelium development |
| 60525 | 2.38E-01 | 3.63E-01 | prostate glandular acinus development |
| 60706 | 2.38E-01 | 3.63E-01 | cell differentiation involved in embryonic placenta development |
| 60742 | 2.38E-01 | 3.63E-01 | epithelial cell differentiation involved in prostate gland development |
| 45026 | 2.38E-01 | 3.63E-01 | plasma membrane fusion |
| 45073 | 2.38E-01 | 3.63E-01 | regulation of chemokine biosynthetic process |
| 45723 | 2.38E-01 | 3.63E-01 | positive regulation of fatty acid biosynthetic process |
| 45912 | 2.38E-01 | 3.63E-01 | negative regulation of carbohydrate metabolic process |
| 45932 | 2.38E-01 | 3.63E-01 | negative regulation of muscle contraction |
| 21544 | 2.38E-01 | 3.63E-01 | subpallium development |
| 30041 | 2.38E-01 | 3.63E-01 | actin filament polymerization |
| 46640 | 2.38E-01 | 3.63E-01 | regulation of alpha-beta T cell proliferation |
| 6107 | 2.38E-01 | 3.63E-01 | oxaloacetate metabolic process |
| 30858 | 2.38E-01 | 3.63E-01 | positive regulation of epithelial cell differentiation |
| 6809 | 2.38E-01 | 3.63E-01 | nitric oxide biosynthetic process |
| 31581 | 2.38E-01 | 3.63E-01 | hemidesmosome assembly |
| 48194 | 2.38E-01 | 3.63E-01 | Golgi vesicle budding |
| 7252 | 2.38E-01 | 3.63E-01 | I-kappaB phosphorylation |
| 7320 | 2.38E-01 | 3.63E-01 | insemination |
| 31998 | 2.38E-01 | 3.63E-01 | regulation of fatty acid beta-oxidation |
| 15697 | 2.38E-01 | 3.63E-01 | quaternary ammonium group transport |
| 15893 | 2.38E-01 | 3.63E-01 | drug transport |
| 48820 | 2.38E-01 | 3.63E-01 | hair follicle maturation |
| 32494 | 2.38E-01 | 3.63E-01 | response to peptidoglycan |
| 8105 | 2.38E-01 | 3.63E-01 | asymmetric protein localization |
| 48704 | 2.44E-01 | 3.72E-01 | embryonic skeletal system morphogenesis |
| 32269 | 2.44E-01 | 3.72E-01 | negative regulation of cellular protein metabolic process |
| 10522 | 2.47E-01 | 3.76E-01 | regulation of calcium ion transport into cytosol |
| 2793 | 2.47E-01 | 3.76E-01 | positive regulation of peptide secretion |
| 21549 | 2.47E-01 | 3.76E-01 | cerebellum development |
| 21954 | 2.47E-01 | 3.76E-01 | central nervous system neuron development |
| 46824 | 2.47E-01 | 3.76E-01 | positive regulation of nucleocytoplasmic transport |
| 21543 | 2.47E-01 | 3.76E-01 | pallium development |
| 9892 | 2.48E-01 | 3.77E-01 | negative regulation of metabolic process |
| 15711 | 2.49E-01 | 3.78E-01 | organic anion transport |
| 51656 | 2.52E-01 | 3.83E-01 | establishment of organelle localization |
| 6818 | 2.57E-01 | 3.91E-01 | hydrogen transport |
| 86 | 2.61E-01 | 3.96E-01 | G2/M transition of mitotic cell cycle |
| 42036 | 2.61E-01 | 3.96E-01 | negative regulation of cytokine biosynthetic process |
| 50927 | 2.61E-01 | 3.96E-01 | positive regulation of positive chemotaxis |
| 51017 | 2.61E-01 | 3.96E-01 | actin filament bundle assembly |
| 19059 | 2.61E-01 | 3.96E-01 | initiation of viral infection |
| 60047 | 2.61E-01 | 3.96E-01 | heart contraction |
| 3015 | 2.61E-01 | 3.96E-01 | heart process |
| 45776 | 2.61E-01 | 3.96E-01 | negative regulation of blood pressure |
| 46330 | 2.61E-01 | 3.96E-01 | positive regulation of JNK cascade |
| 7030 | 2.61E-01 | 3.96E-01 | Golgi organization |
| 32273 | 2.61E-01 | 3.96E-01 | positive regulation of protein polymerization |
| 6733 | 2.62E-01 | 3.96E-01 | oxidoreduction coenzyme metabolic process |
| 32945 | 2.66E-01 | 4.02E-01 | negative regulation of mononuclear cell proliferation |
| 50672 | 2.66E-01 | 4.02E-01 | negative regulation of lymphocyte proliferation |
| 1709 | 2.66E-01 | 4.02E-01 | cell fate determination |
| 10927 | 2.66E-01 | 4.02E-01 | cellular component assembly involved in morphogenesis |
| 3231 | 2.66E-01 | 4.02E-01 | cardiac ventricle development |
| 70664 | 2.66E-01 | 4.02E-01 | negative regulation of leukocyte proliferation |
| 9582 | 2.71E-01 | 4.06E-01 | detection of abiotic stimulus |
| 18130 | 2.71E-01 | 4.06E-01 | heterocycle biosynthetic process |
| 1889 | 2.71E-01 | 4.06E-01 | liver development |
| 33032 | 2.71E-01 | 4.06E-01 | regulation of myeloid cell apoptosis |
| 33865 | 2.71E-01 | 4.06E-01 | nucleoside bisphosphate metabolic process |
| 50650 | 2.71E-01 | 4.06E-01 | chondroitin sulfate proteoglycan biosynthetic process |
| 42522 | 2.71E-01 | 4.06E-01 | regulation of tyrosine phosphorylation of Stat5 protein |
| 50718 | 2.71E-01 | 4.06E-01 | positive regulation of interleukin-1 beta secretion |
| 50856 | 2.71E-01 | 4.06E-01 | regulation of T cell receptor signaling pathway |
| 1909 | 2.71E-01 | 4.06E-01 | leukocyte mediated cytotoxicity |
| 43534 | 2.71E-01 | 4.06E-01 | blood vessel endothelial cell migration |
| 45616 | 2.71E-01 | 4.06E-01 | regulation of keratinocyte differentiation |
| 46112 | 2.71E-01 | 4.06E-01 | nucleobase biosynthetic process |
| 46209 | 2.71E-01 | 4.06E-01 | nitric oxide metabolic process |
| 6906 | 2.71E-01 | 4.06E-01 | vesicle fusion |
| 48199 | 2.71E-01 | 4.06E-01 | vesicle targeting, to, from or within Golgi |
| 7340 | 2.71E-01 | 4.06E-01 | acrosome reaction |
| 7617 | 2.71E-01 | 4.06E-01 | mating behavior |
| 32225 | 2.71E-01 | 4.06E-01 | regulation of synaptic transmission, dopaminergic |
| 48640 | 2.71E-01 | 4.06E-01 | negative regulation of developmental growth |
| 32413 | 2.71E-01 | 4.06E-01 | negative regulation of ion transmembrane transporter activity |
| 48813 | 2.71E-01 | 4.06E-01 | dendrite morphogenesis |
| 33036 | 2.73E-01 | 4.10E-01 | macromolecule localization |
| 34728 | 2.73E-01 | 4.10E-01 | nucleosome organization |
| 35023 | 2.76E-01 | 4.14E-01 | regulation of Rho protein signal transduction |
| 6944 | 2.77E-01 | 4.15E-01 | cellular membrane fusion |
| 7163 | 2.77E-01 | 4.15E-01 | establishment or maintenance of cell polarity |
| 50868 | 2.82E-01 | 4.21E-01 | negative regulation of T cell activation |
| 1942 | 2.82E-01 | 4.21E-01 | hair follicle development |
| 22404 | 2.82E-01 | 4.21E-01 | molting cycle process |
| 22405 | 2.82E-01 | 4.21E-01 | hair cycle process |
| 7276 | 2.84E-01 | 4.21E-01 | gamete generation |
| 9116 | 2.84E-01 | 4.21E-01 | nucleoside metabolic process |
| 61008 | 2.84E-01 | 4.21E-01 | hepaticobiliary system development |
| 17156 | 2.85E-01 | 4.21E-01 | calcium ion-dependent exocytosis |
| 50803 | 2.85E-01 | 4.21E-01 | regulation of synapse structure and activity |
| 50926 | 2.85E-01 | 4.21E-01 | regulation of positive chemotaxis |
| 51693 | 2.85E-01 | 4.21E-01 | actin filament capping |
| 3158 | 2.85E-01 | 4.21E-01 | endothelium development |
| 60603 | 2.85E-01 | 4.21E-01 | mammary gland duct morphogenesis |
| 19674 | 2.85E-01 | 4.21E-01 | NAD metabolic process |
| 45682 | 2.85E-01 | 4.21E-01 | regulation of epidermis development |
| 15695 | 2.85E-01 | 4.21E-01 | organic cation transport |
| 1776 | 2.86E-01 | 4.21E-01 | leukocyte homeostasis |
| 51789 | 2.88E-01 | 4.21E-01 | response to protein stimulus |
| 42113 | 2.89E-01 | 4.21E-01 | B cell activation |
| 6275 | 2.89E-01 | 4.21E-01 | regulation of DNA replication |
| 10741 | 2.92E-01 | 4.21E-01 | negative regulation of intracellular protein kinase cascade |
| 16052 | 2.97E-01 | 4.21E-01 | carbohydrate catabolic process |
| 51054 | 2.98E-01 | 4.21E-01 | positive regulation of DNA metabolic process |
| 32780 | 2.98E-01 | 4.21E-01 | negative regulation of ATPase activity |
| 90130 | 2.98E-01 | 4.21E-01 | tissue migration |
| 8216 | 2.98E-01 | 4.21E-01 | spermidine metabolic process |
| 8218 | 2.98E-01 | 4.21E-01 | bioluminescence |
| 90184 | 2.98E-01 | 4.21E-01 | positive regulation of kidney development |
| 32863 | 2.98E-01 | 4.21E-01 | activation of Rac GTPase activity |
| 32909 | 2.98E-01 | 4.21E-01 | regulation of transforming growth factor-beta2 production |
| 16557 | 2.98E-01 | 4.21E-01 | peroxisome membrane biogenesis |
| 32957 | 2.98E-01 | 4.21E-01 | inositol trisphosphate metabolic process |
| 32966 | 2.98E-01 | 4.21E-01 | negative regulation of collagen biosynthetic process |
| 255 | 2.98E-01 | 4.21E-01 | allantoin metabolic process |
| 33539 | 2.98E-01 | 4.21E-01 | fatty acid beta-oxidation using acyl-CoA dehydrogenase |
| 33591 | 2.98E-01 | 4.21E-01 | response to L-ascorbic acid |
| 33603 | 2.98E-01 | 4.21E-01 | positive regulation of dopamine secretion |
| 920 | 2.98E-01 | 4.21E-01 | cytokinetic cell separation |
| 9129 | 2.98E-01 | 4.21E-01 | pyrimidine nucleoside monophosphate metabolic process |
| 9130 | 2.98E-01 | 4.21E-01 | pyrimidine nucleoside monophosphate biosynthetic process |
| 9191 | 2.98E-01 | 4.21E-01 | ribonucleoside diphosphate catabolic process |
| 33860 | 2.98E-01 | 4.21E-01 | regulation of NAD(P)H oxidase activity |
| 9313 | 2.98E-01 | 4.21E-01 | oligosaccharide catabolic process |
| 42149 | 2.98E-01 | 4.21E-01 | cellular response to glucose starvation |
| 42268 | 2.98E-01 | 4.21E-01 | regulation of cytolysis |
| 34110 | 2.98E-01 | 4.21E-01 | regulation of homotypic cell-cell adhesion |
| 34142 | 2.98E-01 | 4.21E-01 | toll-like receptor 4 signaling pathway |
| 42473 | 2.98E-01 | 4.21E-01 | outer ear morphogenesis |
| 1561 | 2.98E-01 | 4.21E-01 | fatty acid alpha-oxidation |
| 42541 | 2.98E-01 | 4.21E-01 | hemoglobin biosynthetic process |
| 42661 | 2.98E-01 | 4.21E-01 | regulation of mesodermal cell fate specification |
| 1778 | 2.98E-01 | 4.21E-01 | plasma membrane repair |
| 42762 | 2.98E-01 | 4.21E-01 | regulation of sulfur metabolic process |
| 50966 | 2.98E-01 | 4.21E-01 | detection of mechanical stimulus involved in sensory perception of pain |
| 18206 | 2.98E-01 | 4.21E-01 | peptidyl-methionine modification |
| 18208 | 2.98E-01 | 4.21E-01 | peptidyl-proline modification |
| 34629 | 2.98E-01 | 4.21E-01 | cellular protein complex localization |
| 42832 | 2.98E-01 | 4.21E-01 | defense response to protozoan |
| 1911 | 2.98E-01 | 4.21E-01 | negative regulation of leukocyte mediated cytotoxicity |
| 42904 | 2.98E-01 | 4.21E-01 | 9-cis-retinoic acid biosynthetic process |
| 42905 | 2.98E-01 | 4.21E-01 | 9-cis-retinoic acid metabolic process |
| 1956 | 2.98E-01 | 4.21E-01 | positive regulation of neurotransmitter secretion |
| 42976 | 2.98E-01 | 4.21E-01 | activation of Janus kinase activity |
| 43032 | 2.98E-01 | 4.21E-01 | positive regulation of macrophage activation |
| 2118 | 2.98E-01 | 4.21E-01 | aggressive behavior |
| 10389 | 2.98E-01 | 4.21E-01 | regulation of G2/M transition of mitotic cell cycle |
| 43174 | 2.98E-01 | 4.21E-01 | nucleoside salvage |
| 2220 | 2.98E-01 | 4.21E-01 | innate immune response activating cell surface receptor signaling pathway |
| 51409 | 2.98E-01 | 4.21E-01 | response to nitrosative stress |
| 2283 | 2.98E-01 | 4.21E-01 | neutrophil activation involved in immune response |
| 43312 | 2.98E-01 | 4.21E-01 | neutrophil degranulation |
| 51547 | 2.98E-01 | 4.21E-01 | regulation of keratinocyte migration |
| 51549 | 2.98E-01 | 4.21E-01 | positive regulation of keratinocyte migration |
| 10640 | 2.98E-01 | 4.21E-01 | regulation of platelet-derived growth factor receptor signaling pathway |
| 35238 | 2.98E-01 | 4.21E-01 | vitamin A biosynthetic process |
| 10671 | 2.98E-01 | 4.21E-01 | negative regulation of oxygen and reactive oxygen species metabolic process |
| 35306 | 2.98E-01 | 4.21E-01 | positive regulation of dephosphorylation |
| 35307 | 2.98E-01 | 4.21E-01 | positive regulation of protein amino acid dephosphorylation |
| 43501 | 2.98E-01 | 4.21E-01 | skeletal muscle adaptation |
| 10748 | 2.98E-01 | 4.21E-01 | negative regulation of plasma membrane long-chain fatty acid transport |
| 43551 | 2.98E-01 | 4.21E-01 | regulation of phosphoinositide 3-kinase activity |
| 43552 | 2.98E-01 | 4.21E-01 | positive regulation of phosphoinositide 3-kinase activity |
| 10819 | 2.98E-01 | 4.21E-01 | regulation of T cell chemotaxis |
| 10820 | 2.98E-01 | 4.21E-01 | positive regulation of T cell chemotaxis |
| 2639 | 2.98E-01 | 4.21E-01 | positive regulation of immunoglobulin production |
| 10837 | 2.98E-01 | 4.21E-01 | regulation of keratinocyte proliferation |
| 51798 | 2.98E-01 | 4.21E-01 | positive regulation of hair follicle development |
| 43619 | 2.98E-01 | 4.21E-01 | regulation of transcription from RNA polymerase II promoter in response to oxidative stress |
| 60009 | 2.98E-01 | 4.21E-01 | Sertoli cell development |
| 10915 | 2.98E-01 | 4.21E-01 | regulation of very-low-density lipoprotein particle clearance |
| 60068 | 2.98E-01 | 4.21E-01 | vagina development |
| 10916 | 2.98E-01 | 4.21E-01 | negative regulation of very-low-density lipoprotein particle clearance |
| 10935 | 2.98E-01 | 4.21E-01 | regulation of macrophage cytokine production |
| 2829 | 2.98E-01 | 4.21E-01 | negative regulation of T-helper 2 type immune response |
| 60206 | 2.98E-01 | 4.21E-01 | estrous cycle phase |
| 60214 | 2.98E-01 | 4.21E-01 | endocardium formation |
| 60219 | 2.98E-01 | 4.21E-01 | camera-type eye photoreceptor cell differentiation |
| 2883 | 2.98E-01 | 4.21E-01 | regulation of hypersensitivity |
| 60235 | 2.98E-01 | 4.21E-01 | lens induction in camera-type eye |
| 2925 | 2.98E-01 | 4.21E-01 | positive regulation of humoral immune response mediated by circulating immunoglobulin |
| 60292 | 2.98E-01 | 4.21E-01 | long term synaptic depression |
| 19372 | 2.98E-01 | 4.21E-01 | lipoxygenase pathway |
| 3057 | 2.98E-01 | 4.21E-01 | regulation of the force of heart contraction by chemical signal |
| 60430 | 2.98E-01 | 4.21E-01 | lung saccule development |
| 60433 | 2.98E-01 | 4.21E-01 | bronchus development |
| 60456 | 2.98E-01 | 4.21E-01 | positive regulation of digestive system process |
| 19530 | 2.98E-01 | 4.21E-01 | taurine metabolic process |
| 60556 | 2.98E-01 | 4.21E-01 | regulation of vitamin D biosynthetic process |
| 3321 | 2.98E-01 | 4.21E-01 | positive regulation of blood pressure by epinephrine-norepinephrine |
| 60743 | 2.98E-01 | 4.21E-01 | epithelial cell maturation involved in prostate gland development |
| 60753 | 2.98E-01 | 4.21E-01 | regulation of mast cell chemotaxis |
| 60754 | 2.98E-01 | 4.21E-01 | positive regulation of mast cell chemotaxis |
| 60763 | 2.98E-01 | 4.21E-01 | mammary duct terminal end bud growth |
| 19856 | 2.98E-01 | 4.21E-01 | pyrimidine base biosynthetic process |
| 60911 | 2.98E-01 | 4.21E-01 | cardiac cell fate commitment |
| 19987 | 2.98E-01 | 4.21E-01 | negative regulation of anti-apoptosis |
| 60986 | 2.98E-01 | 4.21E-01 | endocrine hormone secretion |
| 45002 | 2.98E-01 | 4.21E-01 | double-strand break repair via single-strand annealing |
| 45091 | 2.98E-01 | 4.21E-01 | regulation of retroviral genome replication |
| 45110 | 2.98E-01 | 4.21E-01 | intermediate filament bundle assembly |
| 45356 | 2.98E-01 | 4.21E-01 | positive regulation of interferon-alpha biosynthetic process |
| 70096 | 2.98E-01 | 4.21E-01 | mitochondrial outer membrane translocase complex assembly |
| 45586 | 2.98E-01 | 4.21E-01 | regulation of gamma-delta T cell differentiation |
| 45588 | 2.98E-01 | 4.21E-01 | positive regulation of gamma-delta T cell differentiation |
| 45627 | 2.98E-01 | 4.21E-01 | positive regulation of T-helper 1 cell differentiation |
| 45780 | 2.98E-01 | 4.21E-01 | positive regulation of bone resorption |
| 70423 | 2.98E-01 | 4.21E-01 | nucleotide-binding oligomerization domain containing signaling pathway |
| 70431 | 2.98E-01 | 4.21E-01 | nucleotide-binding oligomerization domain containing 2 signaling pathway |
| 70527 | 2.98E-01 | 4.21E-01 | platelet aggregation |
| 70528 | 2.98E-01 | 4.21E-01 | protein kinase C signaling cascade |
| 70571 | 2.98E-01 | 4.21E-01 | negative regulation of neuron projection regeneration |
| 46125 | 2.98E-01 | 4.21E-01 | pyrimidine deoxyribonucleoside metabolic process |
| 21569 | 2.98E-01 | 4.21E-01 | rhombomere 3 development |
| 21610 | 2.98E-01 | 4.21E-01 | facial nerve morphogenesis |
| 21612 | 2.98E-01 | 4.21E-01 | facial nerve structural organization |
| 70874 | 2.98E-01 | 4.21E-01 | negative regulation of glycogen metabolic process |
| 46398 | 2.98E-01 | 4.21E-01 | UDP-glucuronate metabolic process |
| 30091 | 2.98E-01 | 4.21E-01 | protein repair |
| 46477 | 2.98E-01 | 4.21E-01 | glycosylceramide catabolic process |
| 46532 | 2.98E-01 | 4.21E-01 | regulation of photoreceptor cell differentiation |
| 21978 | 2.98E-01 | 4.21E-01 | telencephalon regionalization |
| 30202 | 2.98E-01 | 4.21E-01 | heparin metabolic process |
| 30219 | 2.98E-01 | 4.21E-01 | megakaryocyte differentiation |
| 22028 | 2.98E-01 | 4.21E-01 | tangential migration from the subventricular zone to the olfactory bulb |
| 46643 | 2.98E-01 | 4.21E-01 | regulation of gamma-delta T cell activation |
| 46645 | 2.98E-01 | 4.21E-01 | positive regulation of gamma-delta T cell activation |
| 30263 | 2.98E-01 | 4.21E-01 | apoptotic chromosome condensation |
| 14052 | 2.98E-01 | 4.21E-01 | regulation of gamma-aminobutyric acid secretion |
| 46838 | 2.98E-01 | 4.21E-01 | phosphorylated carbohydrate dephosphorylation |
| 46852 | 2.98E-01 | 4.21E-01 | positive regulation of bone remodeling |
| 46855 | 2.98E-01 | 4.21E-01 | inositol phosphate dephosphorylation |
| 46856 | 2.98E-01 | 4.21E-01 | phosphoinositide dephosphorylation |
| 55075 | 2.98E-01 | 4.21E-01 | potassium ion homeostasis |
| 30540 | 2.98E-01 | 4.21E-01 | female genitalia development |
| 46950 | 2.98E-01 | 4.21E-01 | cellular ketone body metabolic process |
| 71549 | 2.98E-01 | 4.21E-01 | cellular response to dexamethasone stimulus |
| 71548 | 2.98E-01 | 4.21E-01 | response to dexamethasone stimulus |
| 30656 | 2.98E-01 | 4.21E-01 | regulation of vitamin metabolic process |
| 6166 | 2.98E-01 | 4.21E-01 | purine ribonucleoside salvage |
| 6207 | 2.98E-01 | 4.21E-01 | 'de novo' pyrimidine base biosynthetic process |
| 22605 | 2.98E-01 | 4.21E-01 | oogenesis stage |
| 6269 | 2.98E-01 | 4.21E-01 | DNA replication, synthesis of RNA primer |
| 6346 | 2.98E-01 | 4.21E-01 | methylation-dependent chromatin silencing |
| 6531 | 2.98E-01 | 4.21E-01 | aspartate metabolic process |
| 6533 | 2.98E-01 | 4.21E-01 | aspartate catabolic process |
| 6545 | 2.98E-01 | 4.21E-01 | glycine biosynthetic process |
| 14826 | 2.98E-01 | 4.21E-01 | vein smooth muscle contraction |
| 6663 | 2.98E-01 | 4.21E-01 | platelet activating factor biosynthetic process |
| 6686 | 2.98E-01 | 4.21E-01 | sphingomyelin biosynthetic process |
| 6704 | 2.98E-01 | 4.21E-01 | glucocorticoid biosynthetic process |
| 31392 | 2.98E-01 | 4.21E-01 | regulation of prostaglandin biosynthetic process |
| 31394 | 2.98E-01 | 4.21E-01 | positive regulation of prostaglandin biosynthetic process |
| 15014 | 2.98E-01 | 4.21E-01 | heparan sulfate proteoglycan biosynthetic process, polysaccharide chain biosynthetic process |
| 6862 | 2.98E-01 | 4.21E-01 | nucleotide transport |
| 31440 | 2.98E-01 | 4.21E-01 | regulation of mRNA 3'-end processing |
| 7008 | 2.98E-01 | 4.21E-01 | outer mitochondrial membrane organization |
| 7035 | 2.98E-01 | 4.21E-01 | vacuolar acidification |
| 7063 | 2.98E-01 | 4.21E-01 | regulation of sister chromatid cohesion |
| 31639 | 2.98E-01 | 4.21E-01 | plasminogen activation |
| 48087 | 2.98E-01 | 4.21E-01 | positive regulation of developmental pigmentation |
| 48143 | 2.98E-01 | 4.21E-01 | astrocyte activation |
| 48293 | 2.98E-01 | 4.21E-01 | regulation of isotype switching to IgE isotypes |
| 7341 | 2.98E-01 | 4.21E-01 | penetration of zona pellucida |
| 32008 | 2.98E-01 | 4.21E-01 | positive regulation of TOR signaling cascade |
| 32069 | 2.98E-01 | 4.21E-01 | regulation of nuclease activity |
| 7500 | 2.98E-01 | 4.21E-01 | mesodermal cell fate determination |
| 32096 | 2.98E-01 | 4.21E-01 | negative regulation of response to food |
| 32099 | 2.98E-01 | 4.21E-01 | negative regulation of appetite |
| 7549 | 2.98E-01 | 4.21E-01 | dosage compensation |
| 15793 | 2.98E-01 | 4.21E-01 | glycerol transport |
| 15802 | 2.98E-01 | 4.21E-01 | basic amino acid transport |
| 15812 | 2.98E-01 | 4.21E-01 | gamma-aminobutyric acid transport |
| 15824 | 2.98E-01 | 4.21E-01 | proline transport |
| 32344 | 2.98E-01 | 4.21E-01 | regulation of aldosterone metabolic process |
| 32488 | 2.98E-01 | 4.21E-01 | Cdc42 protein signal transduction |
| 32516 | 2.98E-01 | 4.21E-01 | positive regulation of phosphoprotein phosphatase activity |
| 16137 | 2.98E-01 | 4.21E-01 | glycoside metabolic process |
| 32525 | 2.98E-01 | 4.21E-01 | somite rostral/caudal axis specification |
| 32650 | 2.98E-01 | 4.21E-01 | regulation of interleukin-1 alpha production |
| 90030 | 2.98E-01 | 4.21E-01 | regulation of steroid hormone biosynthetic process |
| 90037 | 2.98E-01 | 4.21E-01 | positive regulation of protein kinase C signaling cascade |
| 90036 | 2.98E-01 | 4.21E-01 | regulation of protein kinase C signaling cascade |
| 32713 | 2.98E-01 | 4.21E-01 | negative regulation of interleukin-4 production |
| 32730 | 2.98E-01 | 4.21E-01 | positive regulation of interleukin-1 alpha production |
| 46148 | 2.99E-01 | 4.21E-01 | pigment biosynthetic process |
| 48145 | 2.99E-01 | 4.21E-01 | regulation of fibroblast proliferation |
| 6886 | 3.01E-01 | 4.24E-01 | intracellular protein transport |
| 31324 | 3.01E-01 | 4.24E-01 | negative regulation of cellular metabolic process |
| 42346 | 3.03E-01 | 4.24E-01 | positive regulation of NF-kappaB import into nucleus |
| 50706 | 3.03E-01 | 4.24E-01 | regulation of interleukin-1 beta secretion |
| 50716 | 3.03E-01 | 4.24E-01 | positive regulation of interleukin-1 secretion |
| 42559 | 3.03E-01 | 4.24E-01 | pteridine and derivative biosynthetic process |
| 51452 | 3.03E-01 | 4.24E-01 | intracellular pH reduction |
| 51457 | 3.03E-01 | 4.24E-01 | maintenance of protein location in nucleus |
| 43502 | 3.03E-01 | 4.24E-01 | regulation of muscle adaptation |
| 44058 | 3.03E-01 | 4.24E-01 | regulation of digestive system process |
| 60571 | 3.03E-01 | 4.24E-01 | morphogenesis of an epithelial fold |
| 60749 | 3.03E-01 | 4.24E-01 | mammary gland alveolus development |
| 45072 | 3.03E-01 | 4.24E-01 | regulation of interferon-gamma biosynthetic process |
| 46006 | 3.03E-01 | 4.24E-01 | regulation of activated T cell proliferation |
| 30201 | 3.03E-01 | 4.24E-01 | heparan sulfate proteoglycan metabolic process |
| 46716 | 3.03E-01 | 4.24E-01 | muscle cell homeostasis |
| 55081 | 3.03E-01 | 4.24E-01 | anion homeostasis |
| 6555 | 3.03E-01 | 4.24E-01 | methionine metabolic process |
| 6833 | 3.03E-01 | 4.24E-01 | water transport |
| 48260 | 3.03E-01 | 4.24E-01 | positive regulation of receptor-mediated endocytosis |
| 48596 | 3.03E-01 | 4.24E-01 | embryonic camera-type eye morphogenesis |
| 32350 | 3.03E-01 | 4.24E-01 | regulation of hormone metabolic process |
| 48821 | 3.03E-01 | 4.24E-01 | erythrocyte development |
| 32760 | 3.03E-01 | 4.24E-01 | positive regulation of tumor necrosis factor production |
| 8543 | 3.05E-01 | 4.27E-01 | fibroblast growth factor receptor signaling pathway |
| 1667 | 3.05E-01 | 4.27E-01 | ameboidal cell migration |
| 50906 | 3.05E-01 | 4.27E-01 | detection of stimulus involved in sensory perception |
| 1843 | 3.05E-01 | 4.27E-01 | neural tube closure |
| 51187 | 3.05E-01 | 4.27E-01 | cofactor catabolic process |
| 60606 | 3.05E-01 | 4.27E-01 | tube closure |
| 42307 | 3.09E-01 | 4.31E-01 | positive regulation of protein import into nucleus |
| 35264 | 3.09E-01 | 4.31E-01 | multicellular organism growth |
| 45923 | 3.09E-01 | 4.31E-01 | positive regulation of fatty acid metabolic process |
| 21575 | 3.09E-01 | 4.31E-01 | hindbrain morphogenesis |
| 46530 | 3.09E-01 | 4.31E-01 | photoreceptor cell differentiation |
| 46889 | 3.09E-01 | 4.31E-01 | positive regulation of lipid biosynthetic process |
| 6024 | 3.09E-01 | 4.31E-01 | glycosaminoglycan biosynthetic process |
| 50708 | 3.12E-01 | 4.34E-01 | regulation of protein secretion |
| 9791 | 3.12E-01 | 4.34E-01 | post-embryonic development |
| 6800 | 3.12E-01 | 4.34E-01 | oxygen and reactive oxygen species metabolic process |
| 42303 | 3.16E-01 | 4.40E-01 | molting cycle |
| 42633 | 3.16E-01 | 4.40E-01 | hair cycle |
| 8654 | 3.18E-01 | 4.43E-01 | phospholipid biosynthetic process |
| 30833 | 3.22E-01 | 4.48E-01 | regulation of actin filament polymerization |
| 16050 | 3.22E-01 | 4.48E-01 | vesicle organization |
| 8037 | 3.22E-01 | 4.48E-01 | cell recognition |
| 46907 | 3.23E-01 | 4.49E-01 | intracellular transport |
| 30148 | 3.25E-01 | 4.52E-01 | sphingolipid biosynthetic process |
| 10212 | 3.26E-01 | 4.53E-01 | response to ionizing radiation |
| 6650 | 3.29E-01 | 4.56E-01 | glycerophospholipid metabolic process |
| 33059 | 3.33E-01 | 4.61E-01 | cellular pigmentation |
| 33261 | 3.33E-01 | 4.61E-01 | regulation of S phase |
| 17144 | 3.33E-01 | 4.61E-01 | drug metabolic process |
| 9156 | 3.33E-01 | 4.61E-01 | ribonucleoside monophosphate biosynthetic process |
| 19827 | 3.33E-01 | 4.61E-01 | stem cell maintenance |
| 30262 | 3.33E-01 | 4.61E-01 | apoptotic nuclear change |
| 30835 | 3.33E-01 | 4.61E-01 | negative regulation of actin filament depolymerization |
| 40018 | 3.33E-01 | 4.61E-01 | positive regulation of multicellular organism growth |
| 32024 | 3.33E-01 | 4.61E-01 | positive regulation of insulin secretion |
| 51258 | 3.33E-01 | 4.62E-01 | protein polymerization |
| 33121 | 3.35E-01 | 4.62E-01 | regulation of purine nucleotide catabolic process |
| 30811 | 3.35E-01 | 4.62E-01 | regulation of nucleotide catabolic process |
| 42044 | 3.36E-01 | 4.62E-01 | fluid transport |
| 50704 | 3.36E-01 | 4.62E-01 | regulation of interleukin-1 secretion |
| 42554 | 3.36E-01 | 4.62E-01 | superoxide anion generation |
| 50854 | 3.36E-01 | 4.62E-01 | regulation of antigen receptor-mediated signaling pathway |
| 1964 | 3.36E-01 | 4.62E-01 | startle response |
| 10259 | 3.36E-01 | 4.62E-01 | multicellular organismal aging |
| 51954 | 3.36E-01 | 4.62E-01 | positive regulation of amine transport |
| 60441 | 3.36E-01 | 4.62E-01 | epithelial tube branching involved in lung morphogenesis |
| 45408 | 3.36E-01 | 4.62E-01 | regulation of interleukin-6 biosynthetic process |
| 45453 | 3.36E-01 | 4.62E-01 | bone resorption |
| 45851 | 3.36E-01 | 4.62E-01 | pH reduction |
| 21772 | 3.36E-01 | 4.62E-01 | olfactory bulb development |
| 46504 | 3.36E-01 | 4.62E-01 | glycerol ether biosynthetic process |
| 46697 | 3.36E-01 | 4.62E-01 | decidualization |
| 6972 | 3.36E-01 | 4.62E-01 | hyperosmotic response |
| 48339 | 3.36E-01 | 4.62E-01 | paraxial mesoderm development |
| 32104 | 3.36E-01 | 4.62E-01 | regulation of response to extracellular stimulus |
| 32107 | 3.36E-01 | 4.62E-01 | regulation of response to nutrient levels |
| 15800 | 3.36E-01 | 4.62E-01 | acidic amino acid transport |
| 32330 | 3.36E-01 | 4.62E-01 | regulation of chondrocyte differentiation |
| 32400 | 3.36E-01 | 4.62E-01 | melanosome localization |
| 10721 | 3.38E-01 | 4.65E-01 | negative regulation of cell development |
| 51100 | 3.41E-01 | 4.69E-01 | negative regulation of binding |
| 22037 | 3.45E-01 | 4.73E-01 | metencephalon development |
| 48863 | 3.45E-01 | 4.73E-01 | stem cell differentiation |
| 15980 | 3.46E-01 | 4.73E-01 | energy derivation by oxidation of organic compounds |
| 1704 | 3.51E-01 | 4.73E-01 | formation of primary germ layer |
| 1838 | 3.51E-01 | 4.73E-01 | embryonic epithelial tube formation |
| 30203 | 3.53E-01 | 4.73E-01 | glycosaminoglycan metabolic process |
| 33014 | 3.57E-01 | 4.73E-01 | tetrapyrrole biosynthetic process |
| 51588 | 3.57E-01 | 4.73E-01 | regulation of neurotransmitter transport |
| 6023 | 3.57E-01 | 4.73E-01 | aminoglycan biosynthetic process |
| 30816 | 3.57E-01 | 4.73E-01 | positive regulation of cAMP metabolic process |
| 30819 | 3.57E-01 | 4.73E-01 | positive regulation of cAMP biosynthetic process |
| 6779 | 3.57E-01 | 4.73E-01 | porphyrin biosynthetic process |
| 7492 | 3.57E-01 | 4.73E-01 | endoderm development |
| 90218 | 3.58E-01 | 4.73E-01 | positive regulation of lipid kinase activity |
| 90286 | 3.58E-01 | 4.73E-01 | cytoskeletal anchoring at nuclear membrane |
| 33033 | 3.58E-01 | 4.73E-01 | negative regulation of myeloid cell apoptosis |
| 9134 | 3.58E-01 | 4.73E-01 | nucleoside diphosphate catabolic process |
| 42117 | 3.58E-01 | 4.73E-01 | monocyte activation |
| 42362 | 3.58E-01 | 4.73E-01 | fat-soluble vitamin biosynthetic process |
| 1547 | 3.58E-01 | 4.73E-01 | antral ovarian follicle growth |
| 1562 | 3.58E-01 | 4.73E-01 | response to protozoan |
| 42532 | 3.58E-01 | 4.73E-01 | negative regulation of tyrosine phosphorylation of STAT protein |
| 34380 | 3.58E-01 | 4.73E-01 | high-density lipoprotein particle assembly |
| 50857 | 3.58E-01 | 4.73E-01 | positive regulation of antigen receptor-mediated signaling pathway |
| 18101 | 3.58E-01 | 4.73E-01 | peptidyl-citrulline biosynthetic process from peptidyl-arginine |
| 34501 | 3.58E-01 | 4.73E-01 | protein localization to kinetochore |
| 42695 | 3.58E-01 | 4.73E-01 | thelarche |
| 1736 | 3.58E-01 | 4.73E-01 | establishment of planar polarity |
| 42713 | 3.58E-01 | 4.73E-01 | sperm ejaculation |
| 50932 | 3.58E-01 | 4.73E-01 | regulation of pigment cell differentiation |
| 50951 | 3.58E-01 | 4.73E-01 | sensory perception of temperature stimulus |
| 51016 | 3.58E-01 | 4.73E-01 | barbed-end actin filament capping |
| 1878 | 3.58E-01 | 4.73E-01 | response to yeast |
| 1921 | 3.58E-01 | 4.73E-01 | positive regulation of receptor recycling |
| 1961 | 3.58E-01 | 4.73E-01 | positive regulation of cytokine-mediated signaling pathway |
| 1977 | 3.58E-01 | 4.73E-01 | renal system process involved in regulation of blood volume |
| 42940 | 3.58E-01 | 4.73E-01 | D-amino acid transport |
| 1993 | 3.58E-01 | 4.73E-01 | regulation of systemic arterial blood pressure by norepinephrine-epinephrine |
| 2002 | 3.58E-01 | 4.73E-01 | regulation of angiotensin levels in blood |
| 43101 | 3.58E-01 | 4.73E-01 | purine salvage |
| 2246 | 3.58E-01 | 4.73E-01 | wound healing involved in inflammatory response |
| 35058 | 3.58E-01 | 4.73E-01 | sensory cilium assembly |
| 43267 | 3.58E-01 | 4.73E-01 | negative regulation of potassium ion transport |
| 10561 | 3.58E-01 | 4.73E-01 | negative regulation of glycoprotein biosynthetic process |
| 10591 | 3.58E-01 | 4.73E-01 | regulation of lamellipodium assembly |
| 43371 | 3.58E-01 | 4.73E-01 | negative regulation of CD4-positive, alpha beta T cell differentiation |
| 43374 | 3.58E-01 | 4.73E-01 | CD8-positive, alpha-beta T cell differentiation |
| 10713 | 3.58E-01 | 4.73E-01 | negative regulation of collagen metabolic process |
| 10739 | 3.58E-01 | 4.73E-01 | positive regulation of protein kinase A signaling cascade |
| 10765 | 3.58E-01 | 4.73E-01 | positive regulation of sodium ion transport |
| 43569 | 3.58E-01 | 4.73E-01 | negative regulation of insulin-like growth factor receptor signaling pathway |
| 10821 | 3.58E-01 | 4.73E-01 | regulation of mitochondrion organization |
| 51788 | 3.58E-01 | 4.73E-01 | response to misfolded protein |
| 35413 | 3.58E-01 | 4.73E-01 | positive regulation of catenin protein nuclear translocation |
| 51797 | 3.58E-01 | 4.73E-01 | regulation of hair follicle development |
| 43618 | 3.58E-01 | 4.73E-01 | regulation of transcription from RNA polymerase II promoter in response to stress |
| 60054 | 3.58E-01 | 4.73E-01 | positive regulation of epithelial cell proliferation involved in wound healing |
| 60055 | 3.58E-01 | 4.73E-01 | angiogenesis involved in wound healing |
| 10923 | 3.58E-01 | 4.73E-01 | negative regulation of phosphatase activity |
| 51890 | 3.58E-01 | 4.73E-01 | regulation of cardioblast differentiation |
| 51891 | 3.58E-01 | 4.73E-01 | positive regulation of cardioblast differentiation |
| 51894 | 3.58E-01 | 4.73E-01 | positive regulation of focal adhesion assembly |
| 2755 | 3.58E-01 | 4.73E-01 | MyD88-dependent toll-like receptor signaling pathway |
| 60216 | 3.58E-01 | 4.73E-01 | definitive hemopoiesis |
| 60236 | 3.58E-01 | 4.73E-01 | regulation of mitotic spindle organization |
| 2903 | 3.58E-01 | 4.73E-01 | negative regulation of B cell apoptosis |
| 60259 | 3.58E-01 | 4.73E-01 | regulation of feeding behavior |
| 2923 | 3.58E-01 | 4.73E-01 | regulation of humoral immune response mediated by circulating immunoglobulin |
| 3085 | 3.58E-01 | 4.73E-01 | negative regulation of systemic arterial blood pressure |
| 60439 | 3.58E-01 | 4.73E-01 | trachea morphogenesis |
| 44091 | 3.58E-01 | 4.73E-01 | membrane biogenesis |
| 60632 | 3.58E-01 | 4.73E-01 | regulation of microtubule-based movement |
| 44252 | 3.58E-01 | 4.73E-01 | negative regulation of multicellular organismal metabolic process |
| 60666 | 3.58E-01 | 4.73E-01 | dichotomous subdivision of terminal units involved in salivary gland branching |
| 60744 | 3.58E-01 | 4.73E-01 | mammary gland branching involved in thelarche |
| 60770 | 3.58E-01 | 4.73E-01 | negative regulation of epithelial cell proliferation involved in prostate gland development |
| 60900 | 3.58E-01 | 4.73E-01 | embryonic camera-type eye formation |
| 61005 | 3.58E-01 | 4.73E-01 | cell differentiation involved in kidney development |
| 45008 | 3.58E-01 | 4.73E-01 | depyrimidination |
| 45010 | 3.58E-01 | 4.73E-01 | actin nucleation |
| 45070 | 3.58E-01 | 4.73E-01 | positive regulation of viral genome replication |
| 45354 | 3.58E-01 | 4.73E-01 | regulation of interferon-alpha biosynthetic process |
| 45409 | 3.58E-01 | 4.73E-01 | negative regulation of interleukin-6 biosynthetic process |
| 45606 | 3.58E-01 | 4.73E-01 | positive regulation of epidermal cell differentiation |
| 45618 | 3.58E-01 | 4.73E-01 | positive regulation of keratinocyte differentiation |
| 45623 | 3.58E-01 | 4.73E-01 | negative regulation of T-helper cell differentiation |
| 45634 | 3.58E-01 | 4.73E-01 | regulation of melanocyte differentiation |
| 70233 | 3.58E-01 | 4.73E-01 | negative regulation of T cell apoptosis |
| 70391 | 3.58E-01 | 4.73E-01 | response to lipoteichoic acid |
| 45880 | 3.58E-01 | 4.73E-01 | positive regulation of smoothened signaling pathway |
| 70472 | 3.58E-01 | 4.73E-01 | regulation of uterine smooth muscle contraction |
| 70474 | 3.58E-01 | 4.73E-01 | positive regulation of uterine smooth muscle contraction |
| 45986 | 3.58E-01 | 4.73E-01 | negative regulation of smooth muscle contraction |
| 45988 | 3.58E-01 | 4.73E-01 | negative regulation of striated muscle contraction |
| 21561 | 3.58E-01 | 4.73E-01 | facial nerve development |
| 21604 | 3.58E-01 | 4.73E-01 | cranial nerve structural organization |
| 21800 | 3.58E-01 | 4.73E-01 | cerebral cortex tangential migration |
| 21891 | 3.58E-01 | 4.73E-01 | olfactory bulb interneuron development |
| 46469 | 3.58E-01 | 4.73E-01 | platelet activating factor metabolic process |
| 30224 | 3.58E-01 | 4.73E-01 | monocyte differentiation |
| 30240 | 3.58E-01 | 4.73E-01 | skeletal muscle thin filament assembly |
| 30321 | 3.58E-01 | 4.73E-01 | transepithelial chloride transport |
| 71385 | 3.58E-01 | 4.73E-01 | cellular response to glucocorticoid stimulus |
| 71384 | 3.58E-01 | 4.73E-01 | cellular response to corticosteroid stimulus |
| 14047 | 3.58E-01 | 4.73E-01 | glutamate secretion |
| 46827 | 3.58E-01 | 4.73E-01 | positive regulation of protein export from nucleus |
| 46874 | 3.58E-01 | 4.73E-01 | quinolinate metabolic process |
| 5984 | 3.58E-01 | 4.73E-01 | disaccharide metabolic process |
| 22417 | 3.58E-01 | 4.73E-01 | protein maturation by protein folding |
| 6102 | 3.58E-01 | 4.73E-01 | isocitrate metabolic process |
| 71681 | 3.58E-01 | 4.73E-01 | cellular response to indole-3-methanol |
| 71680 | 3.58E-01 | 4.73E-01 | response to indole-3-methanol |
| 30825 | 3.58E-01 | 4.73E-01 | positive regulation of cGMP metabolic process |
| 30828 | 3.58E-01 | 4.73E-01 | positive regulation of cGMP biosynthetic process |
| 6551 | 3.58E-01 | 4.73E-01 | leucine metabolic process |
| 6573 | 3.58E-01 | 4.73E-01 | valine metabolic process |
| 14850 | 3.58E-01 | 4.73E-01 | response to muscle activity |
| 14896 | 3.58E-01 | 4.73E-01 | muscle hypertrophy |
| 31342 | 3.58E-01 | 4.73E-01 | negative regulation of cell killing |
| 6824 | 3.58E-01 | 4.73E-01 | cobalt ion transport |
| 48011 | 3.58E-01 | 4.73E-01 | nerve growth factor receptor signaling pathway |
| 7175 | 3.58E-01 | 4.73E-01 | negative regulation of epidermal growth factor receptor activity |
| 48149 | 3.58E-01 | 4.73E-01 | behavioral response to ethanol |
| 7195 | 3.58E-01 | 4.73E-01 | inhibition of adenylate cyclase activity by dopamine receptor signaling pathway |
| 7199 | 3.58E-01 | 4.73E-01 | G-protein signaling, coupled to cGMP nucleotide second messenger |
| 7256 | 3.58E-01 | 4.73E-01 | activation of JNKK activity |
| 48266 | 3.58E-01 | 4.73E-01 | behavioral response to pain |
| 31953 | 3.58E-01 | 4.73E-01 | negative regulation of protein amino acid autophosphorylation |
| 7598 | 3.58E-01 | 4.73E-01 | blood coagulation, extrinsic pathway |
| 15840 | 3.58E-01 | 4.73E-01 | urea transport |
| 32232 | 3.58E-01 | 4.73E-01 | negative regulation of actin filament bundle assembly |
| 48715 | 3.58E-01 | 4.73E-01 | negative regulation of oligodendrocyte differentiation |
| 32366 | 3.58E-01 | 4.73E-01 | intracellular sterol transport |
| 32367 | 3.58E-01 | 4.73E-01 | intracellular cholesterol transport |
| 48843 | 3.58E-01 | 4.73E-01 | negative regulation of axon extension involved in axon guidance |
| 32506 | 3.58E-01 | 4.73E-01 | cytokinetic process |
| 90004 | 3.58E-01 | 4.73E-01 | positive regulation of establishment of protein localization in plasma membrane |
| 8089 | 3.58E-01 | 4.73E-01 | anterograde axon cargo transport |
| 32691 | 3.58E-01 | 4.73E-01 | negative regulation of interleukin-1 beta production |
| 90075 | 3.58E-01 | 4.73E-01 | relaxation of muscle |
| 90080 | 3.58E-01 | 4.73E-01 | positive regulation of MAPKKK cascade by fibroblast growth factor receptor signaling pathway |
| 90083 | 3.58E-01 | 4.73E-01 | regulation of inclusion body assembly |
| 19953 | 3.61E-01 | 4.77E-01 | sexual reproduction |
| 61180 | 3.65E-01 | 4.81E-01 | mammary gland epithelium development |
| 14020 | 3.65E-01 | 4.81E-01 | primary neural tube formation |
| 42157 | 3.67E-01 | 4.81E-01 | lipoprotein metabolic process |
| 8209 | 3.67E-01 | 4.81E-01 | androgen metabolic process |
| 8272 | 3.67E-01 | 4.81E-01 | sulfate transport |
| 16998 | 3.67E-01 | 4.81E-01 | cell wall macromolecule catabolic process |
| 17158 | 3.67E-01 | 4.81E-01 | regulation of calcium ion-dependent exocytosis |
| 9143 | 3.67E-01 | 4.81E-01 | nucleoside triphosphate catabolic process |
| 51148 | 3.67E-01 | 4.81E-01 | negative regulation of muscle cell differentiation |
| 51453 | 3.67E-01 | 4.81E-01 | regulation of intracellular pH |
| 35088 | 3.67E-01 | 4.81E-01 | establishment or maintenance of apical/basal cell polarity |
| 43526 | 3.67E-01 | 4.81E-01 | neuroprotection |
| 19079 | 3.67E-01 | 4.81E-01 | viral genome replication |
| 2717 | 3.67E-01 | 4.81E-01 | positive regulation of natural killer cell mediated immunity |
| 51875 | 3.67E-01 | 4.81E-01 | pigment granule localization |
| 51963 | 3.67E-01 | 4.81E-01 | regulation of synaptogenesis |
| 19363 | 3.67E-01 | 4.81E-01 | pyridine nucleotide biosynthetic process |
| 45069 | 3.67E-01 | 4.81E-01 | regulation of viral genome replication |
| 45604 | 3.67E-01 | 4.81E-01 | regulation of epidermal cell differentiation |
| 45620 | 3.67E-01 | 4.81E-01 | negative regulation of lymphocyte differentiation |
| 45954 | 3.67E-01 | 4.81E-01 | positive regulation of natural killer cell mediated cytotoxicity |
| 30032 | 3.67E-01 | 4.81E-01 | lamellipodium assembly |
| 21846 | 3.67E-01 | 4.81E-01 | cell proliferation in forebrain |
| 21904 | 3.67E-01 | 4.81E-01 | dorsal/ventral neural tube patterning |
| 21988 | 3.67E-01 | 4.81E-01 | olfactory lobe development |
| 71156 | 3.67E-01 | 4.81E-01 | regulation of cell cycle arrest |
| 46632 | 3.67E-01 | 4.81E-01 | alpha-beta T cell differentiation |
| 30282 | 3.67E-01 | 4.81E-01 | bone mineralization |
| 6611 | 3.67E-01 | 4.81E-01 | protein export from nucleus |
| 7172 | 3.67E-01 | 4.81E-01 | signal complex assembly |
| 32092 | 3.67E-01 | 4.81E-01 | positive regulation of protein binding |
| 16079 | 3.67E-01 | 4.81E-01 | synaptic vesicle exocytosis |
| 32722 | 3.67E-01 | 4.81E-01 | positive regulation of chemokine production |
| 271 | 3.68E-01 | 4.82E-01 | polysaccharide biosynthetic process |
| 2761 | 3.68E-01 | 4.82E-01 | regulation of myeloid leukocyte differentiation |
| 45185 | 3.68E-01 | 4.82E-01 | maintenance of protein location |
| 44275 | 3.80E-01 | 4.98E-01 | cellular carbohydrate catabolic process |
| 33692 | 3.81E-01 | 4.98E-01 | cellular polysaccharide biosynthetic process |
| 42130 | 3.81E-01 | 4.98E-01 | negative regulation of T cell proliferation |
| 50771 | 3.81E-01 | 4.98E-01 | negative regulation of axonogenesis |
| 42743 | 3.81E-01 | 4.98E-01 | hydrogen peroxide metabolic process |
| 48641 | 3.81E-01 | 4.98E-01 | regulation of skeletal muscle tissue development |
| 48864 | 3.81E-01 | 4.98E-01 | stem cell development |
| 22900 | 3.84E-01 | 5.02E-01 | electron transport chain |
| 10001 | 3.84E-01 | 5.02E-01 | glial cell differentiation |
| 40014 | 3.84E-01 | 5.02E-01 | regulation of multicellular organism growth |
| 32507 | 3.84E-01 | 5.02E-01 | maintenance of protein location in cell |
| 51928 | 3.85E-01 | 5.02E-01 | positive regulation of calcium ion transport |
| 46467 | 3.85E-01 | 5.02E-01 | membrane lipid biosynthetic process |
| 910 | 3.85E-01 | 5.02E-01 | cytokinesis |
| 45935 | 3.87E-01 | 5.05E-01 | positive regulation of nucleobase, nucleoside, nucleotide and nucleic acid metabolic process |
| 42770 | 3.90E-01 | 5.09E-01 | DNA damage response, signal transduction |
| 70302 | 3.94E-01 | 5.13E-01 | regulation of stress-activated protein kinase signaling cascade |
| 16197 | 3.97E-01 | 5.17E-01 | endosome transport |
| 42491 | 3.99E-01 | 5.17E-01 | auditory receptor cell differentiation |
| 34605 | 3.99E-01 | 5.17E-01 | cellular response to heat |
| 50996 | 3.99E-01 | 5.17E-01 | positive regulation of lipid catabolic process |
| 51279 | 3.99E-01 | 5.17E-01 | regulation of release of sequestered calcium ion into cytosol |
| 19098 | 3.99E-01 | 5.17E-01 | reproductive behavior |
| 44036 | 3.99E-01 | 5.17E-01 | cell wall macromolecule metabolic process |
| 70206 | 3.99E-01 | 5.17E-01 | protein trimerization |
| 30204 | 3.99E-01 | 5.17E-01 | chondroitin sulfate metabolic process |
| 71554 | 3.99E-01 | 5.17E-01 | cell wall organization or biogenesis |
| 6760 | 3.99E-01 | 5.17E-01 | folic acid and derivative metabolic process |
| 6333 | 4.02E-01 | 5.17E-01 | chromatin assembly or disassembly |
| 51153 | 4.04E-01 | 5.17E-01 | regulation of striated muscle cell differentiation |
| 43331 | 4.04E-01 | 5.17E-01 | response to dsRNA |
| 45666 | 4.04E-01 | 5.17E-01 | positive regulation of neuron differentiation |
| 30433 | 4.04E-01 | 5.17E-01 | ER-associated protein catabolic process |
| 7041 | 4.04E-01 | 5.17E-01 | lysosomal transport |
| 2573 | 4.04E-01 | 5.17E-01 | myeloid leukocyte differentiation |
| 8228 | 4.12E-01 | 5.17E-01 | opsonization |
| 90224 | 4.12E-01 | 5.17E-01 | regulation of spindle organization |
| 16584 | 4.12E-01 | 5.17E-01 | nucleosome positioning |
| 9120 | 4.12E-01 | 5.17E-01 | deoxyribonucleoside metabolic process |
| 42159 | 4.12E-01 | 5.17E-01 | lipoprotein catabolic process |
| 34105 | 4.12E-01 | 5.17E-01 | positive regulation of tissue remodeling |
| 34308 | 4.12E-01 | 5.17E-01 | monohydric alcohol metabolic process |
| 42535 | 4.12E-01 | 5.17E-01 | positive regulation of tumor necrosis factor biosynthetic process |
| 42537 | 4.12E-01 | 5.17E-01 | benzene and derivative metabolic process |
| 1580 | 4.12E-01 | 5.17E-01 | detection of chemical stimulus involved in sensory perception of bitter taste |
| 34384 | 4.12E-01 | 5.17E-01 | high-density lipoprotein particle clearance |
| 42634 | 4.12E-01 | 5.17E-01 | regulation of hair cycle |
| 1706 | 4.12E-01 | 5.17E-01 | endoderm formation |
| 10042 | 4.12E-01 | 5.17E-01 | response to manganese ion |
| 42886 | 4.12E-01 | 5.17E-01 | amide transport |
| 34698 | 4.12E-01 | 5.17E-01 | response to gonadotropin stimulus |
| 18345 | 4.12E-01 | 5.17E-01 | protein palmitoylation |
| 2063 | 4.12E-01 | 5.17E-01 | chondrocyte development |
| 43149 | 4.12E-01 | 5.17E-01 | stress fiber assembly |
| 43171 | 4.12E-01 | 5.17E-01 | peptide catabolic process |
| 10560 | 4.12E-01 | 5.17E-01 | positive regulation of glycoprotein biosynthetic process |
| 2437 | 4.12E-01 | 5.17E-01 | inflammatory response to antigenic stimulus |
| 51590 | 4.12E-01 | 5.17E-01 | positive regulation of neurotransmitter transport |
| 2448 | 4.12E-01 | 5.17E-01 | mast cell mediated immunity |
| 10656 | 4.12E-01 | 5.17E-01 | negative regulation of muscle cell apoptosis |
| 51654 | 4.12E-01 | 5.17E-01 | establishment of mitochondrion localization |
| 2532 | 4.12E-01 | 5.17E-01 | production of molecular mediator involved in inflammatory response |
| 10738 | 4.12E-01 | 5.17E-01 | regulation of protein kinase A signaling cascade |
| 10746 | 4.12E-01 | 5.17E-01 | regulation of plasma membrane long-chain fatty acid transport |
| 43620 | 4.12E-01 | 5.17E-01 | regulation of transcription in response to stress |
| 60008 | 4.12E-01 | 5.17E-01 | Sertoli cell differentiation |
| 60017 | 4.12E-01 | 5.17E-01 | parathyroid gland development |
| 10866 | 4.12E-01 | 5.17E-01 | regulation of triglyceride biosynthetic process |
| 10887 | 4.12E-01 | 5.17E-01 | negative regulation of cholesterol storage |
| 51938 | 4.12E-01 | 5.17E-01 | L-glutamate import |
| 10985 | 4.12E-01 | 5.17E-01 | negative regulation of lipoprotein particle clearance |
| 51962 | 4.12E-01 | 5.17E-01 | positive regulation of nervous system development |
| 51965 | 4.12E-01 | 5.17E-01 | positive regulation of synaptogenesis |
| 51967 | 4.12E-01 | 5.17E-01 | negative regulation of synaptic transmission, glutamatergic |
| 60177 | 4.12E-01 | 5.17E-01 | regulation of angiotensin metabolic process |
| 2855 | 4.12E-01 | 5.17E-01 | regulation of natural killer cell mediated immune response to tumor cell |
| 19240 | 4.12E-01 | 5.17E-01 | citrulline biosynthetic process |
| 2857 | 4.12E-01 | 5.17E-01 | positive regulation of natural killer cell mediated immune response to tumor cell |
| 2858 | 4.12E-01 | 5.17E-01 | regulation of natural killer cell mediated cytotoxicity directed against tumor cell target |
| 2860 | 4.12E-01 | 5.17E-01 | positive regulation of natural killer cell mediated cytotoxicity directed against tumor cell target |
| 52031 | 4.12E-01 | 5.17E-01 | modulation by symbiont of host defense response |
| 52033 | 4.12E-01 | 5.17E-01 | pathogen-associated molecular pattern dependent induction by symbiont of host innate immunity |
| 52166 | 4.12E-01 | 5.17E-01 | positive regulation by symbiont of host innate immunity |
| 52167 | 4.12E-01 | 5.17E-01 | modulation by symbiont of host innate immunity |
| 52169 | 4.12E-01 | 5.17E-01 | pathogen-associated molecular pattern dependent modulation by symbiont of host innate immunity |
| 43981 | 4.12E-01 | 5.17E-01 | histone H4-K5 acetylation |
| 43982 | 4.12E-01 | 5.17E-01 | histone H4-K8 acetylation |
| 60412 | 4.12E-01 | 5.17E-01 | ventricular septum morphogenesis |
| 52255 | 4.12E-01 | 5.17E-01 | modulation by organism of defense response of other organism involved in symbiotic interaction |
| 52257 | 4.12E-01 | 5.17E-01 | pathogen-associated molecular pattern dependent induction by organism of innate immunity of other organism involved in symbiotic interaction |
| 85029 | 4.12E-01 | 5.17E-01 | extracellular matrix assembly |
| 44089 | 4.12E-01 | 5.17E-01 | positive regulation of cellular component biogenesis |
| 3151 | 4.12E-01 | 5.17E-01 | outflow tract morphogenesis |
| 52305 | 4.12E-01 | 5.17E-01 | positive regulation by organism of innate immunity in other organism involved in symbiotic interaction |
| 52306 | 4.12E-01 | 5.17E-01 | modulation by organism of innate immunity in other organism involved in symbiotic interaction |
| 52308 | 4.12E-01 | 5.17E-01 | pathogen-associated molecular pattern dependent modulation by organism of innate immunity in other organism involved in symbiotic interaction |
| 60572 | 4.12E-01 | 5.17E-01 | morphogenesis of an epithelial bud |
| 60670 | 4.12E-01 | 5.17E-01 | branching involved in embryonic placenta morphogenesis |
| 60687 | 4.12E-01 | 5.17E-01 | regulation of branching involved in prostate gland morphogenesis |
| 52509 | 4.12E-01 | 5.17E-01 | positive regulation by symbiont of host defense response |
| 52510 | 4.12E-01 | 5.17E-01 | positive regulation by organism of defense response of other organism involved in symbiotic interaction |
| 60707 | 4.12E-01 | 5.17E-01 | trophoblast giant cell differentiation |
| 52552 | 4.12E-01 | 5.17E-01 | modulation by organism of immune response of other organism involved in symbiotic interaction |
| 52553 | 4.12E-01 | 5.17E-01 | modulation by symbiont of host immune response |
| 52555 | 4.12E-01 | 5.17E-01 | positive regulation by organism of immune response of other organism involved in symbiotic interaction |
| 52556 | 4.12E-01 | 5.17E-01 | positive regulation by symbiont of host immune response |
| 52564 | 4.12E-01 | 5.17E-01 | response to immune response of other organism involved in symbiotic interaction |
| 19800 | 4.12E-01 | 5.17E-01 | peptide cross-linking via chondroitin 4-sulfate glycosaminoglycan |
| 52572 | 4.12E-01 | 5.17E-01 | response to host immune response |
| 61099 | 4.12E-01 | 5.17E-01 | negative regulation of protein tyrosine kinase activity |
| 45410 | 4.12E-01 | 5.17E-01 | positive regulation of interleukin-6 biosynthetic process |
| 45601 | 4.12E-01 | 5.17E-01 | regulation of endothelial cell differentiation |
| 45625 | 4.12E-01 | 5.17E-01 | regulation of T-helper 1 cell differentiation |
| 45655 | 4.12E-01 | 5.17E-01 | regulation of monocyte differentiation |
| 45663 | 4.12E-01 | 5.17E-01 | positive regulation of myoblast differentiation |
| 46031 | 4.12E-01 | 5.17E-01 | ADP metabolic process |
| 46033 | 4.12E-01 | 5.17E-01 | AMP metabolic process |
| 46426 | 4.12E-01 | 5.17E-01 | negative regulation of JAK-STAT cascade |
| 46473 | 4.12E-01 | 5.17E-01 | phosphatidic acid metabolic process |
| 46514 | 4.12E-01 | 5.17E-01 | ceramide catabolic process |
| 71157 | 4.12E-01 | 5.17E-01 | negative regulation of cell cycle arrest |
| 46639 | 4.12E-01 | 5.17E-01 | negative regulation of alpha-beta T cell differentiation |
| 14050 | 4.12E-01 | 5.17E-01 | negative regulation of glutamate secretion |
| 46839 | 4.12E-01 | 5.17E-01 | phospholipid dephosphorylation |
| 30513 | 4.12E-01 | 5.17E-01 | positive regulation of BMP signaling pathway |
| 55091 | 4.12E-01 | 5.17E-01 | phospholipid homeostasis |
| 5980 | 4.12E-01 | 5.17E-01 | glycogen catabolic process |
| 71560 | 4.12E-01 | 5.17E-01 | cellular response to transforming growth factor beta stimulus |
| 30643 | 4.12E-01 | 5.17E-01 | cellular phosphate ion homeostasis |
| 6067 | 4.12E-01 | 5.17E-01 | ethanol metabolic process |
| 6069 | 4.12E-01 | 5.17E-01 | ethanol oxidation |
| 71636 | 4.12E-01 | 5.17E-01 | positive regulation of transforming growth factor-beta production |
| 6171 | 4.12E-01 | 5.17E-01 | cAMP biosynthetic process |
| 6467 | 4.12E-01 | 5.17E-01 | protein thiol-disulfide exchange |
| 6477 | 4.12E-01 | 5.17E-01 | protein amino acid sulfation |
| 6590 | 4.12E-01 | 5.17E-01 | thyroid hormone generation |
| 6642 | 4.12E-01 | 5.17E-01 | triglyceride mobilization |
| 14866 | 4.12E-01 | 5.17E-01 | skeletal myofibril assembly |
| 14888 | 4.12E-01 | 5.17E-01 | striated muscle adaptation |
| 6702 | 4.12E-01 | 5.17E-01 | androgen biosynthetic process |
| 48010 | 4.12E-01 | 5.17E-01 | vascular endothelial growth factor receptor signaling pathway |
| 31641 | 4.12E-01 | 5.17E-01 | regulation of myelination |
| 7184 | 4.12E-01 | 5.17E-01 | SMAD protein nuclear translocation |
| 7191 | 4.12E-01 | 5.17E-01 | activation of adenylate cyclase activity by dopamine receptor signaling pathway |
| 40023 | 4.12E-01 | 5.17E-01 | establishment of nucleus localization |
| 7260 | 4.12E-01 | 5.17E-01 | tyrosine phosphorylation of STAT protein |
| 40037 | 4.12E-01 | 5.17E-01 | negative regulation of fibroblast growth factor receptor signaling pathway |
| 48247 | 4.12E-01 | 5.17E-01 | lymphocyte chemotaxis |
| 48261 | 4.12E-01 | 5.17E-01 | negative regulation of receptor-mediated endocytosis |
| 31929 | 4.12E-01 | 5.17E-01 | TOR signaling cascade |
| 48387 | 4.12E-01 | 5.17E-01 | negative regulation of retinoic acid receptor signaling pathway |
| 15696 | 4.12E-01 | 5.17E-01 | ammonium transport |
| 48537 | 4.12E-01 | 5.17E-01 | mucosal-associated lymphoid tissue development |
| 48541 | 4.12E-01 | 5.17E-01 | Peyer's patch development |
| 15810 | 4.12E-01 | 5.17E-01 | aspartate transport |
| 32331 | 4.12E-01 | 5.17E-01 | negative regulation of chondrocyte differentiation |
| 48739 | 4.12E-01 | 5.17E-01 | cardiac muscle fiber development |
| 32429 | 4.12E-01 | 5.17E-01 | regulation of phospholipase A2 activity |
| 48841 | 4.12E-01 | 5.17E-01 | regulation of axon extension involved in axon guidance |
| 32469 | 4.12E-01 | 5.17E-01 | endoplasmic reticulum calcium ion homeostasis |
| 16090 | 4.12E-01 | 5.17E-01 | prenol metabolic process |
| 16093 | 4.12E-01 | 5.17E-01 | polyprenol metabolic process |
| 32692 | 4.12E-01 | 5.17E-01 | negative regulation of interleukin-1 production |
| 32743 | 4.12E-01 | 5.17E-01 | positive regulation of interleukin-2 production |
| 8584 | 4.15E-01 | 5.21E-01 | male gonad development |
| 30518 | 4.15E-01 | 5.21E-01 | steroid hormone receptor signaling pathway |
| 1707 | 4.24E-01 | 5.31E-01 | mesoderm formation |
| 51147 | 4.24E-01 | 5.31E-01 | regulation of muscle cell differentiation |
| 60828 | 4.24E-01 | 5.31E-01 | regulation of canonical Wnt receptor signaling pathway |
| 9127 | 4.29E-01 | 5.35E-01 | purine nucleoside monophosphate biosynthetic process |
| 9168 | 4.29E-01 | 5.35E-01 | purine ribonucleoside monophosphate biosynthetic process |
| 42516 | 4.29E-01 | 5.35E-01 | regulation of tyrosine phosphorylation of Stat3 protein |
| 42573 | 4.29E-01 | 5.35E-01 | retinoic acid metabolic process |
| 35116 | 4.29E-01 | 5.35E-01 | embryonic hindlimb morphogenesis |
| 35383 | 4.29E-01 | 5.35E-01 | thioester metabolic process |
| 51926 | 4.29E-01 | 5.35E-01 | negative regulation of calcium ion transport |
| 21795 | 4.29E-01 | 5.35E-01 | cerebral cortex cell migration |
| 30239 | 4.29E-01 | 5.35E-01 | myofibril assembly |
| 71222 | 4.29E-01 | 5.35E-01 | cellular response to lipopolysaccharide |
| 6004 | 4.29E-01 | 5.35E-01 | fucose metabolic process |
| 6541 | 4.29E-01 | 5.35E-01 | glutamine metabolic process |
| 6637 | 4.29E-01 | 5.35E-01 | acyl-CoA metabolic process |
| 6901 | 4.29E-01 | 5.35E-01 | vesicle coating |
| 48048 | 4.29E-01 | 5.35E-01 | embryonic eye morphogenesis |
| 7622 | 4.29E-01 | 5.35E-01 | rhythmic behavior |
| 9124 | 4.44E-01 | 5.53E-01 | nucleoside monophosphate biosynthetic process |
| 42990 | 4.44E-01 | 5.53E-01 | regulation of transcription factor import into nucleus |
| 2700 | 4.44E-01 | 5.53E-01 | regulation of production of molecular mediator of immune response |
| 51056 | 4.50E-01 | 5.61E-01 | regulation of small GTPase mediated signal transduction |
| 1947 | 4.51E-01 | 5.61E-01 | heart looping |
| 51289 | 4.51E-01 | 5.61E-01 | protein homotetramerization |
| 43547 | 4.51E-01 | 5.61E-01 | positive regulation of GTPase activity |
| 3143 | 4.51E-01 | 5.61E-01 | embryonic heart tube morphogenesis |
| 21983 | 4.51E-01 | 5.61E-01 | pituitary gland development |
| 7006 | 4.51E-01 | 5.61E-01 | mitochondrial membrane organization |
| 7270 | 4.51E-01 | 5.61E-01 | nerve-nerve synaptic transmission |
| 31327 | 4.51E-01 | 5.61E-01 | negative regulation of cellular biosynthetic process |
| 737 | 4.59E-01 | 5.62E-01 | DNA catabolic process, endonucleolytic |
| 42462 | 4.59E-01 | 5.62E-01 | eye photoreceptor cell development |
| 50850 | 4.59E-01 | 5.62E-01 | positive regulation of calcium-mediated signaling |
| 18205 | 4.59E-01 | 5.62E-01 | peptidyl-lysine modification |
| 51181 | 4.59E-01 | 5.62E-01 | cofactor transport |
| 43489 | 4.59E-01 | 5.62E-01 | RNA stabilization |
| 51702 | 4.59E-01 | 5.62E-01 | interaction with symbiont |
| 60042 | 4.59E-01 | 5.62E-01 | retina morphogenesis in camera-type eye |
| 60079 | 4.59E-01 | 5.62E-01 | regulation of excitatory postsynaptic membrane potential |
| 60674 | 4.59E-01 | 5.62E-01 | placenta blood vessel development |
| 46131 | 4.59E-01 | 5.62E-01 | pyrimidine ribonucleoside metabolic process |
| 46488 | 4.59E-01 | 5.62E-01 | phosphatidylinositol metabolic process |
| 46717 | 4.59E-01 | 5.62E-01 | acid secretion |
| 6309 | 4.59E-01 | 5.62E-01 | DNA fragmentation involved in apoptotic nuclear change |
| 14902 | 4.59E-01 | 5.62E-01 | myotube differentiation |
| 48255 | 4.59E-01 | 5.62E-01 | mRNA stabilization |
| 30111 | 4.60E-01 | 5.62E-01 | regulation of Wnt receptor signaling pathway |
| 52 | 4.62E-01 | 5.62E-01 | citrulline metabolic process |
| 32933 | 4.62E-01 | 5.62E-01 | SREBP-mediated signaling pathway |
| 9135 | 4.62E-01 | 5.62E-01 | purine nucleoside diphosphate metabolic process |
| 9179 | 4.62E-01 | 5.62E-01 | purine ribonucleoside diphosphate metabolic process |
| 9223 | 4.62E-01 | 5.62E-01 | pyrimidine deoxyribonucleotide catabolic process |
| 9251 | 4.62E-01 | 5.62E-01 | glucan catabolic process |
| 9404 | 4.62E-01 | 5.62E-01 | toxin metabolic process |
| 42359 | 4.62E-01 | 5.62E-01 | vitamin D metabolic process |
| 50746 | 4.62E-01 | 5.62E-01 | regulation of lipoprotein metabolic process |
| 9820 | 4.62E-01 | 5.62E-01 | alkaloid metabolic process |
| 50858 | 4.62E-01 | 5.62E-01 | negative regulation of antigen receptor-mediated signaling pathway |
| 50860 | 4.62E-01 | 5.62E-01 | negative regulation of T cell receptor signaling pathway |
| 34508 | 4.62E-01 | 5.62E-01 | centromere complex assembly |
| 50910 | 4.62E-01 | 5.62E-01 | detection of mechanical stimulus involved in sensory perception of sound |
| 50922 | 4.62E-01 | 5.62E-01 | negative regulation of chemotaxis |
| 1839 | 4.62E-01 | 5.62E-01 | neural plate morphogenesis |
| 1895 | 4.62E-01 | 5.62E-01 | retina homeostasis |
| 1967 | 4.62E-01 | 5.62E-01 | suckling behavior |
| 51131 | 4.62E-01 | 5.62E-01 | chaperone-mediated protein complex assembly |
| 2031 | 4.62E-01 | 5.62E-01 | G-protein coupled receptor internalization |
| 10310 | 4.62E-01 | 5.62E-01 | regulation of hydrogen peroxide metabolic process |
| 2282 | 4.62E-01 | 5.62E-01 | microglial cell activation involved in immune response |
| 2320 | 4.62E-01 | 5.62E-01 | lymphoid progenitor cell differentiation |
| 43297 | 4.62E-01 | 5.62E-01 | apical junction assembly |
| 10669 | 4.62E-01 | 5.62E-01 | epithelial structure maintenance |
| 35304 | 4.62E-01 | 5.62E-01 | regulation of protein amino acid dephosphorylation |
| 51775 | 4.62E-01 | 5.62E-01 | response to redox state |
| 10824 | 4.62E-01 | 5.62E-01 | regulation of centrosome duplication |
| 51955 | 4.62E-01 | 5.62E-01 | regulation of amino acid transport |
| 60263 | 4.62E-01 | 5.62E-01 | regulation of respiratory burst |
| 43983 | 4.62E-01 | 5.62E-01 | histone H4-K12 acetylation |
| 60405 | 4.62E-01 | 5.62E-01 | regulation of penile erection |
| 60600 | 4.62E-01 | 5.62E-01 | dichotomous subdivision of an epithelial terminal unit |
| 44247 | 4.62E-01 | 5.62E-01 | cellular polysaccharide catabolic process |
| 20027 | 4.62E-01 | 5.62E-01 | hemoglobin metabolic process |
| 45084 | 4.62E-01 | 5.62E-01 | positive regulation of interleukin-12 biosynthetic process |
| 45348 | 4.62E-01 | 5.62E-01 | positive regulation of MHC class II biosynthetic process |
| 45357 | 4.62E-01 | 5.62E-01 | regulation of interferon-beta biosynthetic process |
| 45359 | 4.62E-01 | 5.62E-01 | positive regulation of interferon-beta biosynthetic process |
| 45416 | 4.62E-01 | 5.62E-01 | positive regulation of interleukin-8 biosynthetic process |
| 70232 | 4.62E-01 | 5.62E-01 | regulation of T cell apoptosis |
| 45879 | 4.62E-01 | 5.62E-01 | negative regulation of smoothened signaling pathway |
| 70570 | 4.62E-01 | 5.62E-01 | regulation of neuron projection regeneration |
| 21783 | 4.62E-01 | 5.62E-01 | preganglionic parasympathetic nervous system development |
| 21889 | 4.62E-01 | 5.62E-01 | olfactory bulb interneuron differentiation |
| 46521 | 4.62E-01 | 5.62E-01 | sphingoid catabolic process |
| 46622 | 4.62E-01 | 5.62E-01 | positive regulation of organ growth |
| 30277 | 4.62E-01 | 5.62E-01 | maintenance of gastrointestinal epithelium |
| 30319 | 4.62E-01 | 5.62E-01 | cellular di-, tri-valent inorganic anion homeostasis |
| 55062 | 4.62E-01 | 5.62E-01 | phosphate ion homeostasis |
| 71559 | 4.62E-01 | 5.62E-01 | response to transforming growth factor beta stimulus |
| 30889 | 4.62E-01 | 5.62E-01 | negative regulation of B cell proliferation |
| 6622 | 4.62E-01 | 5.62E-01 | protein targeting to lysosome |
| 6623 | 4.62E-01 | 5.62E-01 | protein targeting to vacuole |
| 31274 | 4.62E-01 | 5.62E-01 | positive regulation of pseudopodium assembly |
| 6700 | 4.62E-01 | 5.62E-01 | C21-steroid hormone biosynthetic process |
| 6977 | 4.62E-01 | 5.62E-01 | DNA damage response, signal transduction by p53 class mediator resulting in cell cycle arrest |
| 7158 | 4.62E-01 | 5.62E-01 | neuron cell-cell adhesion |
| 7216 | 4.62E-01 | 5.62E-01 | metabotropic glutamate receptor signaling pathway |
| 40015 | 4.62E-01 | 5.62E-01 | negative regulation of multicellular organism growth |
| 32000 | 4.62E-01 | 5.62E-01 | positive regulation of fatty acid beta-oxidation |
| 32042 | 4.62E-01 | 5.62E-01 | mitochondrial DNA metabolic process |
| 32105 | 4.62E-01 | 5.62E-01 | negative regulation of response to extracellular stimulus |
| 32108 | 4.62E-01 | 5.62E-01 | negative regulation of response to nutrient levels |
| 15791 | 4.62E-01 | 5.62E-01 | polyol transport |
| 15838 | 4.62E-01 | 5.62E-01 | betaine transport |
| 15879 | 4.62E-01 | 5.62E-01 | carnitine transport |
| 48679 | 4.62E-01 | 5.62E-01 | regulation of axon regeneration |
| 32465 | 4.62E-01 | 5.62E-01 | regulation of cytokinesis |
| 16255 | 4.62E-01 | 5.62E-01 | attachment of GPI anchor to protein |
| 90003 | 4.62E-01 | 5.62E-01 | regulation of establishment of protein localization in plasma membrane |
| 43393 | 4.63E-01 | 5.63E-01 | regulation of protein binding |
| 48332 | 4.63E-01 | 5.63E-01 | mesoderm morphogenesis |
| 31669 | 4.68E-01 | 5.68E-01 | cellular response to nutrient levels |
| 9314 | 4.69E-01 | 5.69E-01 | response to radiation |
| 10628 | 4.70E-01 | 5.71E-01 | positive regulation of gene expression |
| 50768 | 4.71E-01 | 5.72E-01 | negative regulation of neurogenesis |
| 43392 | 4.77E-01 | 5.79E-01 | negative regulation of DNA binding |
| 15985 | 4.82E-01 | 5.85E-01 | energy coupled proton transport, down electrochemical gradient |
| 15986 | 4.82E-01 | 5.85E-01 | ATP synthesis coupled proton transport |
| 42147 | 4.87E-01 | 5.90E-01 | retrograde transport, endosome to Golgi |
| 50807 | 4.87E-01 | 5.90E-01 | regulation of synapse organization |
| 2444 | 4.87E-01 | 5.90E-01 | myeloid leukocyte mediated immunity |
| 51966 | 4.87E-01 | 5.90E-01 | regulation of synaptic transmission, glutamatergic |
| 21587 | 4.87E-01 | 5.90E-01 | cerebellum morphogenesis |
| 71300 | 4.87E-01 | 5.90E-01 | cellular response to retinoic acid |
| 6471 | 4.87E-01 | 5.90E-01 | protein amino acid ADP-ribosylation |
| 6891 | 4.87E-01 | 5.90E-01 | intra-Golgi vesicle-mediated transport |
| 32392 | 4.87E-01 | 5.90E-01 | DNA geometric change |
| 32508 | 4.87E-01 | 5.90E-01 | DNA duplex unwinding |
| 34599 | 4.88E-01 | 5.90E-01 | cellular response to oxidative stress |
| 30178 | 4.88E-01 | 5.90E-01 | negative regulation of Wnt receptor signaling pathway |
| 30384 | 4.96E-01 | 5.99E-01 | phosphoinositide metabolic process |
| 60 | 4.96E-01 | 5.99E-01 | protein import into nucleus, translocation |
| 14032 | 4.96E-01 | 5.99E-01 | neural crest cell development |
| 7034 | 4.96E-01 | 5.99E-01 | vacuolar transport |
| 1841 | 5.01E-01 | 6.00E-01 | neural tube formation |
| 90068 | 5.01E-01 | 6.00E-01 | positive regulation of cell cycle process |
| 9890 | 5.01E-01 | 6.00E-01 | negative regulation of biosynthetic process |
| 6022 | 5.07E-01 | 6.00E-01 | aminoglycan metabolic process |
| 32816 | 5.08E-01 | 6.00E-01 | positive regulation of natural killer cell activation |
| 32855 | 5.08E-01 | 6.00E-01 | positive regulation of Rac GTPase activity |
| 32891 | 5.08E-01 | 6.00E-01 | negative regulation of organic acid transport |
| 16540 | 5.08E-01 | 6.00E-01 | protein autoprocessing |
| 33005 | 5.08E-01 | 6.00E-01 | positive regulation of mast cell activation |
| 33205 | 5.08E-01 | 6.00E-01 | cell cycle cytokinesis |
| 8631 | 5.08E-01 | 6.00E-01 | induction of apoptosis by oxidative stress |
| 8653 | 5.08E-01 | 6.00E-01 | lipopolysaccharide metabolic process |
| 17085 | 5.08E-01 | 6.00E-01 | response to insecticide |
| 33483 | 5.08E-01 | 6.00E-01 | gas homeostasis |
| 9103 | 5.08E-01 | 6.00E-01 | lipopolysaccharide biosynthetic process |
| 9113 | 5.08E-01 | 6.00E-01 | purine base biosynthetic process |
| 9164 | 5.08E-01 | 6.00E-01 | nucleoside catabolic process |
| 42074 | 5.08E-01 | 6.00E-01 | cell migration involved in gastrulation |
| 42136 | 5.08E-01 | 6.00E-01 | neurotransmitter biosynthetic process |
| 9435 | 5.08E-01 | 6.00E-01 | NAD biosynthetic process |
| 75136 | 5.08E-01 | 6.00E-01 | response to host |
| 9629 | 5.08E-01 | 6.00E-01 | response to gravity |
| 34285 | 5.08E-01 | 6.00E-01 | response to disaccharide stimulus |
| 9744 | 5.08E-01 | 6.00E-01 | response to sucrose stimulus |
| 42574 | 5.08E-01 | 6.00E-01 | retinal metabolic process |
| 42640 | 5.08E-01 | 6.00E-01 | anagen |
| 1710 | 5.08E-01 | 6.00E-01 | mesodermal cell fate commitment |
| 50912 | 5.08E-01 | 6.00E-01 | detection of chemical stimulus involved in sensory perception of taste |
| 42756 | 5.08E-01 | 6.00E-01 | drinking behavior |
| 50957 | 5.08E-01 | 6.00E-01 | equilibrioception |
| 1840 | 5.08E-01 | 6.00E-01 | neural plate development |
| 1945 | 5.08E-01 | 6.00E-01 | lymph vessel development |
| 42989 | 5.08E-01 | 6.00E-01 | sequestering of actin monomers |
| 2087 | 5.08E-01 | 6.00E-01 | regulation of respiratory gaseous exchange by neurological system process |
| 43094 | 5.08E-01 | 6.00E-01 | cellular metabolic compound salvage |
| 43243 | 5.08E-01 | 6.00E-01 | positive regulation of protein complex disassembly |
| 35065 | 5.08E-01 | 6.00E-01 | regulation of histone acetylation |
| 43288 | 5.08E-01 | 6.00E-01 | apocarotenoid metabolic process |
| 35121 | 5.08E-01 | 6.00E-01 | tail morphogenesis |
| 51568 | 5.08E-01 | 6.00E-01 | histone H3-K4 methylation |
| 43550 | 5.08E-01 | 6.00E-01 | regulation of lipid kinase activity |
| 35412 | 5.08E-01 | 6.00E-01 | regulation of catenin protein nuclear translocation |
| 60045 | 5.08E-01 | 6.00E-01 | positive regulation of cardiac muscle cell proliferation |
| 2704 | 5.08E-01 | 6.00E-01 | negative regulation of leukocyte mediated immunity |
| 2707 | 5.08E-01 | 6.00E-01 | negative regulation of lymphocyte mediated immunity |
| 51898 | 5.08E-01 | 6.00E-01 | negative regulation of protein kinase B signaling cascade |
| 51927 | 5.08E-01 | 6.00E-01 | negative regulation of calcium ion transport via voltage-gated calcium channel activity |
| 60123 | 5.08E-01 | 6.00E-01 | regulation of growth hormone secretion |
| 60134 | 5.08E-01 | 6.00E-01 | prepulse inhibition |
| 10984 | 5.08E-01 | 6.00E-01 | regulation of lipoprotein particle clearance |
| 2825 | 5.08E-01 | 6.00E-01 | regulation of T-helper 1 type immune response |
| 2834 | 5.08E-01 | 6.00E-01 | regulation of response to tumor cell |
| 2836 | 5.08E-01 | 6.00E-01 | positive regulation of response to tumor cell |
| 2837 | 5.08E-01 | 6.00E-01 | regulation of immune response to tumor cell |
| 2839 | 5.08E-01 | 6.00E-01 | positive regulation of immune response to tumor cell |
| 60314 | 5.08E-01 | 6.00E-01 | regulation of ryanodine-sensitive calcium-release channel activity |
| 52173 | 5.08E-01 | 6.00E-01 | response to defenses of other organism involved in symbiotic interaction |
| 52200 | 5.08E-01 | 6.00E-01 | response to host defenses |
| 60438 | 5.08E-01 | 6.00E-01 | trachea development |
| 44065 | 5.08E-01 | 6.00E-01 | regulation of respiratory system process |
| 60644 | 5.08E-01 | 6.00E-01 | mammary gland epithelial cell differentiation |
| 45075 | 5.08E-01 | 6.00E-01 | regulation of interleukin-12 biosynthetic process |
| 45161 | 5.08E-01 | 6.00E-01 | neuronal ion channel clustering |
| 45214 | 5.08E-01 | 6.00E-01 | sarcomere organization |
| 45600 | 5.08E-01 | 6.00E-01 | positive regulation of fat cell differentiation |
| 45843 | 5.08E-01 | 6.00E-01 | negative regulation of striated muscle tissue development |
| 45910 | 5.08E-01 | 6.00E-01 | negative regulation of DNA recombination |
| 21680 | 5.08E-01 | 6.00E-01 | cerebellar Purkinje cell layer development |
| 21781 | 5.08E-01 | 6.00E-01 | glial cell fate commitment |
| 71158 | 5.08E-01 | 6.00E-01 | positive regulation of cell cycle arrest |
| 46605 | 5.08E-01 | 6.00E-01 | regulation of centrosome cycle |
| 14002 | 5.08E-01 | 6.00E-01 | astrocyte development |
| 55061 | 5.08E-01 | 6.00E-01 | di-, tri-valent inorganic anion homeostasis |
| 6108 | 5.08E-01 | 6.00E-01 | malate metabolic process |
| 6244 | 5.08E-01 | 6.00E-01 | pyrimidine nucleotide catabolic process |
| 30826 | 5.08E-01 | 6.00E-01 | regulation of cGMP biosynthetic process |
| 6285 | 5.08E-01 | 6.00E-01 | base-excision repair, AP site formation |
| 6491 | 5.08E-01 | 6.00E-01 | N-glycan processing |
| 6527 | 5.08E-01 | 6.00E-01 | arginine catabolic process |
| 31272 | 5.08E-01 | 6.00E-01 | regulation of pseudopodium assembly |
| 6882 | 5.08E-01 | 6.00E-01 | cellular zinc ion homeostasis |
| 48148 | 5.08E-01 | 6.00E-01 | behavioral response to cocaine |
| 48385 | 5.08E-01 | 6.00E-01 | regulation of retinoic acid receptor signaling pathway |
| 32098 | 5.08E-01 | 6.00E-01 | regulation of appetite |
| 48569 | 5.08E-01 | 6.00E-01 | post-embryonic organ development |
| 48703 | 5.08E-01 | 6.00E-01 | embryonic viscerocranium morphogenesis |
| 48710 | 5.08E-01 | 6.00E-01 | regulation of astrocyte differentiation |
| 32332 | 5.08E-01 | 6.00E-01 | positive regulation of chondrocyte differentiation |
| 32365 | 5.08E-01 | 6.00E-01 | intracellular lipid transport |
| 48857 | 5.08E-01 | 6.00E-01 | neural nucleus development |
| 32490 | 5.08E-01 | 6.00E-01 | detection of molecule of bacterial origin |
| 32733 | 5.08E-01 | 6.00E-01 | positive regulation of interleukin-10 production |
| 7586 | 5.12E-01 | 6.04E-01 | digestion |
| 7346 | 5.13E-01 | 6.05E-01 | regulation of mitotic cell cycle |
| 8589 | 5.15E-01 | 6.06E-01 | regulation of smoothened signaling pathway |
| 9595 | 5.15E-01 | 6.06E-01 | detection of biotic stimulus |
| 34612 | 5.15E-01 | 6.06E-01 | response to tumor necrosis factor |
| 10043 | 5.15E-01 | 6.06E-01 | response to zinc ion |
| 1975 | 5.15E-01 | 6.06E-01 | response to amphetamine |
| 2275 | 5.15E-01 | 6.06E-01 | myeloid cell activation involved in immune response |
| 43525 | 5.15E-01 | 6.06E-01 | positive regulation of neuron apoptosis |
| 45446 | 5.15E-01 | 6.06E-01 | endothelial cell differentiation |
| 22029 | 5.15E-01 | 6.06E-01 | telencephalon cell migration |
| 71219 | 5.15E-01 | 6.06E-01 | cellular response to molecule of bacterial origin |
| 30317 | 5.15E-01 | 6.06E-01 | sperm motility |
| 71299 | 5.15E-01 | 6.06E-01 | cellular response to vitamin A |
| 46928 | 5.15E-01 | 6.06E-01 | regulation of neurotransmitter secretion |
| 7214 | 5.15E-01 | 6.06E-01 | gamma-aminobutyric acid signaling pathway |
| 48284 | 5.15E-01 | 6.06E-01 | organelle fusion |
| 48599 | 5.15E-01 | 6.06E-01 | oocyte development |
| 32436 | 5.15E-01 | 6.06E-01 | positive regulation of proteasomal ubiquitin-dependent protein catabolic process |
| 42733 | 5.17E-01 | 6.08E-01 | embryonic digit morphogenesis |
| 14033 | 5.17E-01 | 6.08E-01 | neural crest cell differentiation |
| 43161 | 5.18E-01 | 6.09E-01 | proteasomal ubiquitin-dependent protein catabolic process |
| 10498 | 5.18E-01 | 6.09E-01 | proteasomal protein catabolic process |
| 1816 | 5.19E-01 | 6.10E-01 | cytokine production |
| 7033 | 5.19E-01 | 6.10E-01 | vacuole organization |
| 46578 | 5.19E-01 | 6.10E-01 | regulation of Ras protein signal transduction |
| 33365 | 5.19E-01 | 6.10E-01 | protein localization in organelle |
| 44403 | 5.21E-01 | 6.11E-01 | symbiosis, encompassing mutualism through parasitism |
| 7369 | 5.23E-01 | 6.14E-01 | gastrulation |
| 43433 | 5.37E-01 | 6.29E-01 | negative regulation of transcription factor activity |
| 90048 | 5.37E-01 | 6.29E-01 | negative regulation of transcription regulator activity |
| 34504 | 5.37E-01 | 6.29E-01 | protein localization in nucleus |
| 51262 | 5.37E-01 | 6.30E-01 | protein tetramerization |
| 1708 | 5.39E-01 | 6.31E-01 | cell fate specification |
| 1906 | 5.39E-01 | 6.31E-01 | cell killing |
| 43409 | 5.39E-01 | 6.31E-01 | negative regulation of MAPKKK cascade |
| 42461 | 5.42E-01 | 6.32E-01 | photoreceptor cell development |
| 50684 | 5.42E-01 | 6.32E-01 | regulation of mRNA processing |
| 9994 | 5.42E-01 | 6.32E-01 | oocyte differentiation |
| 45931 | 5.42E-01 | 6.32E-01 | positive regulation of mitotic cell cycle |
| 21532 | 5.42E-01 | 6.32E-01 | neural tube patterning |
| 21885 | 5.42E-01 | 6.32E-01 | forebrain cell migration |
| 46470 | 5.42E-01 | 6.32E-01 | phosphatidylcholine metabolic process |
| 30574 | 5.42E-01 | 6.32E-01 | collagen catabolic process |
| 6692 | 5.42E-01 | 6.32E-01 | prostanoid metabolic process |
| 6693 | 5.42E-01 | 6.32E-01 | prostaglandin metabolic process |
| 6783 | 5.42E-01 | 6.32E-01 | heme biosynthetic process |
| 32677 | 5.42E-01 | 6.32E-01 | regulation of interleukin-8 production |
| 16055 | 5.49E-01 | 6.32E-01 | Wnt receptor signaling pathway |
| 32814 | 5.50E-01 | 6.32E-01 | regulation of natural killer cell activation |
| 90263 | 5.50E-01 | 6.32E-01 | positive regulation of canonical Wnt receptor signaling pathway |
| 160 | 5.50E-01 | 6.32E-01 | two-component signal transduction system (phosphorelay) |
| 16601 | 5.50E-01 | 6.32E-01 | Rac protein signal transduction |
| 33144 | 5.50E-01 | 6.32E-01 | negative regulation of steroid hormone receptor signaling pathway |
| 381 | 5.50E-01 | 6.32E-01 | regulation of alternative nuclear mRNA splicing, via spliceosome |
| 33275 | 5.50E-01 | 6.32E-01 | actin-myosin filament sliding |
| 33605 | 5.50E-01 | 6.32E-01 | positive regulation of catecholamine secretion |
| 9185 | 5.50E-01 | 6.32E-01 | ribonucleoside diphosphate metabolic process |
| 9203 | 5.50E-01 | 6.32E-01 | ribonucleoside triphosphate catabolic process |
| 9207 | 5.50E-01 | 6.32E-01 | purine ribonucleoside triphosphate catabolic process |
| 42026 | 5.50E-01 | 6.32E-01 | protein refolding |
| 42119 | 5.50E-01 | 6.32E-01 | neutrophil activation |
| 34121 | 5.50E-01 | 6.32E-01 | regulation of toll-like receptor signaling pathway |
| 42355 | 5.50E-01 | 6.32E-01 | L-fucose catabolic process |
| 42403 | 5.50E-01 | 6.32E-01 | thyroid hormone metabolic process |
| 42438 | 5.50E-01 | 6.32E-01 | melanin biosynthetic process |
| 18065 | 5.50E-01 | 6.32E-01 | protein-cofactor linkage |
| 50907 | 5.50E-01 | 6.32E-01 | detection of chemical stimulus involved in sensory perception |
| 50913 | 5.50E-01 | 6.32E-01 | sensory perception of bitter taste |
| 1782 | 5.50E-01 | 6.32E-01 | B cell homeostasis |
| 18195 | 5.50E-01 | 6.32E-01 | peptidyl-arginine modification |
| 42772 | 5.50E-01 | 6.32E-01 | DNA damage response, signal transduction resulting in transcription |
| 1963 | 5.50E-01 | 6.32E-01 | synaptic transmission, dopaminergic |
| 1976 | 5.50E-01 | 6.32E-01 | neurological system process involved in regulation of systemic arterial blood pressure |
| 42953 | 5.50E-01 | 6.32E-01 | lipoprotein transport |
| 2833 | 5.50E-01 | 6.32E-01 | positive regulation of response to biotic stimulus |
| 60260 | 5.50E-01 | 6.32E-01 | regulation of transcription initiation from RNA polymerase II promoter |
| 19317 | 5.50E-01 | 6.32E-01 | fucose catabolic process |
| 44003 | 5.50E-01 | 6.32E-01 | modification by symbiont of host morphology or physiology |
| 60712 | 5.50E-01 | 6.32E-01 | spongiotrophoblast layer development |
| 45047 | 5.50E-01 | 6.32E-01 | protein targeting to ER |
| 45197 | 5.50E-01 | 6.32E-01 | establishment or maintenance of epithelial cell apical/basal polarity |
| 45414 | 5.50E-01 | 6.32E-01 | regulation of interleukin-8 biosynthetic process |
| 70252 | 5.50E-01 | 6.32E-01 | actin-mediated cell contraction |
| 70373 | 5.50E-01 | 6.32E-01 | negative regulation of ERK1 and ERK2 cascade |
| 30049 | 5.50E-01 | 6.32E-01 | muscle filament sliding |
| 21936 | 5.50E-01 | 6.32E-01 | regulation of granule cell precursor proliferation |
| 21940 | 5.50E-01 | 6.32E-01 | positive regulation of granule cell precursor proliferation |
| 30206 | 5.50E-01 | 6.32E-01 | chondroitin sulfate biosynthetic process |
| 30431 | 5.50E-01 | 6.32E-01 | sleep |
| 55069 | 5.50E-01 | 6.32E-01 | zinc ion homeostasis |
| 46885 | 5.50E-01 | 6.32E-01 | regulation of hormone biosynthetic process |
| 30517 | 5.50E-01 | 6.32E-01 | negative regulation of axon extension |
| 46902 | 5.50E-01 | 6.32E-01 | regulation of mitochondrial membrane permeability |
| 6206 | 5.50E-01 | 6.32E-01 | pyrimidine base metabolic process |
| 30815 | 5.50E-01 | 6.32E-01 | negative regulation of cAMP metabolic process |
| 30818 | 5.50E-01 | 6.32E-01 | negative regulation of cAMP biosynthetic process |
| 30823 | 5.50E-01 | 6.32E-01 | regulation of cGMP metabolic process |
| 6265 | 5.50E-01 | 6.32E-01 | DNA topological change |
| 6266 | 5.50E-01 | 6.32E-01 | DNA ligation |
| 47496 | 5.50E-01 | 6.32E-01 | vesicle transport along microtubule |
| 6878 | 5.50E-01 | 6.32E-01 | cellular copper ion homeostasis |
| 31529 | 5.50E-01 | 6.32E-01 | ruffle organization |
| 6978 | 5.50E-01 | 6.32E-01 | DNA damage response, signal transduction by p53 class mediator resulting in transcription of p21 class mediator |
| 48009 | 5.50E-01 | 6.32E-01 | insulin-like growth factor receptor signaling pathway |
| 31648 | 5.50E-01 | 6.32E-01 | protein destabilization |
| 7342 | 5.50E-01 | 6.32E-01 | fusion of sperm to egg plasma membrane |
| 48333 | 5.50E-01 | 6.32E-01 | mesodermal cell differentiation |
| 7413 | 5.50E-01 | 6.32E-01 | axonal fasciculation |
| 32095 | 5.50E-01 | 6.32E-01 | regulation of response to food |
| 15721 | 5.50E-01 | 6.32E-01 | bile acid and bile salt transport |
| 7625 | 5.50E-01 | 6.32E-01 | grooming behavior |
| 48617 | 5.50E-01 | 6.32E-01 | embryonic foregut morphogenesis |
| 48635 | 5.50E-01 | 6.32E-01 | negative regulation of muscle organ development |
| 32402 | 5.50E-01 | 6.32E-01 | melanosome transport |
| 32647 | 5.50E-01 | 6.32E-01 | regulation of interferon-alpha production |
| 1764 | 5.53E-01 | 6.34E-01 | neuron migration |
| 35282 | 5.53E-01 | 6.34E-01 | segmentation |
| 31647 | 5.53E-01 | 6.34E-01 | regulation of protein stability |
| 7005 | 5.59E-01 | 6.42E-01 | mitochondrion organization |
| 35051 | 5.59E-01 | 6.42E-01 | cardiac cell differentiation |
| 60271 | 5.59E-01 | 6.42E-01 | cilium morphogenesis |
| 42742 | 5.60E-01 | 6.42E-01 | defense response to bacterium |
| 31398 | 5.62E-01 | 6.44E-01 | positive regulation of protein ubiquitination |
| 45017 | 5.63E-01 | 6.46E-01 | glycerolipid biosynthetic process |
| 8633 | 5.68E-01 | 6.49E-01 | activation of pro-apoptotic gene products |
| 42312 | 5.68E-01 | 6.49E-01 | regulation of vasodilation |
| 2027 | 5.68E-01 | 6.49E-01 | regulation of heart rate |
| 60078 | 5.68E-01 | 6.49E-01 | regulation of postsynaptic membrane potential |
| 60113 | 5.68E-01 | 6.49E-01 | inner ear receptor cell differentiation |
| 60425 | 5.68E-01 | 6.49E-01 | lung morphogenesis |
| 45494 | 5.68E-01 | 6.49E-01 | photoreceptor cell maintenance |
| 6099 | 5.68E-01 | 6.49E-01 | tricarboxylic acid cycle |
| 16338 | 5.68E-01 | 6.49E-01 | calcium-independent cell-cell adhesion |
| 22411 | 5.68E-01 | 6.50E-01 | cellular component disassembly |
| 45941 | 5.68E-01 | 6.50E-01 | positive regulation of transcription |
| 6470 | 5.70E-01 | 6.51E-01 | protein amino acid dephosphorylation |
| 8104 | 5.71E-01 | 6.53E-01 | protein localization |
| 10608 | 5.74E-01 | 6.56E-01 | posttranscriptional regulation of gene expression |
| 16311 | 5.79E-01 | 6.61E-01 | dephosphorylation |
| 34765 | 5.79E-01 | 6.62E-01 | regulation of ion transmembrane transport |
| 21761 | 5.79E-01 | 6.62E-01 | limbic system development |
| 7368 | 5.79E-01 | 6.62E-01 | determination of left/right symmetry |
| 31396 | 5.81E-01 | 6.62E-01 | regulation of protein ubiquitination |
| 51254 | 5.82E-01 | 6.62E-01 | positive regulation of RNA metabolic process |
| 19882 | 5.83E-01 | 6.62E-01 | antigen processing and presentation |
| 2 | 5.88E-01 | 6.62E-01 | mitochondrial genome maintenance |
| 32862 | 5.88E-01 | 6.62E-01 | activation of Rho GTPase activity |
| 33057 | 5.88E-01 | 6.62E-01 | reproductive behavior in a multicellular organism |
| 9077 | 5.88E-01 | 6.62E-01 | histidine family amino acid catabolic process |
| 9086 | 5.88E-01 | 6.62E-01 | methionine biosynthetic process |
| 42088 | 5.88E-01 | 6.62E-01 | T-helper 1 type immune response |
| 42104 | 5.88E-01 | 6.62E-01 | positive regulation of activated T cell proliferation |
| 42523 | 5.88E-01 | 6.62E-01 | positive regulation of tyrosine phosphorylation of Stat5 protein |
| 42659 | 5.88E-01 | 6.62E-01 | regulation of cell fate specification |
| 1738 | 5.88E-01 | 6.62E-01 | morphogenesis of a polarized epithelium |
| 50908 | 5.88E-01 | 6.62E-01 | detection of light stimulus involved in visual perception |
| 50962 | 5.88E-01 | 6.62E-01 | detection of light stimulus involved in sensory perception |
| 2029 | 5.88E-01 | 6.62E-01 | desensitization of G-protein coupled receptor protein signaling pathway |
| 2070 | 5.88E-01 | 6.62E-01 | epithelial cell maturation |
| 10453 | 5.88E-01 | 6.62E-01 | regulation of cell fate commitment |
| 2281 | 5.88E-01 | 6.62E-01 | macrophage activation involved in immune response |
| 51482 | 5.88E-01 | 6.62E-01 | elevation of cytosolic calcium ion concentration involved in G-protein signaling coupled to IP3 second messenger |
| 10660 | 5.88E-01 | 6.62E-01 | regulation of muscle cell apoptosis |
| 43491 | 5.88E-01 | 6.62E-01 | protein kinase B signaling cascade |
| 51904 | 5.88E-01 | 6.62E-01 | pigment granule transport |
| 2820 | 5.88E-01 | 6.62E-01 | negative regulation of adaptive immune response |
| 2823 | 5.88E-01 | 6.62E-01 | negative regulation of adaptive immune response based on somatic recombination of immune receptors built from immunoglobulin superfamily domains |
| 51983 | 5.88E-01 | 6.62E-01 | regulation of chromosome segregation |
| 19432 | 5.88E-01 | 6.62E-01 | triglyceride biosynthetic process |
| 60411 | 5.88E-01 | 6.62E-01 | cardiac septum morphogenesis |
| 60736 | 5.88E-01 | 6.62E-01 | prostate gland growth |
| 60795 | 5.88E-01 | 6.62E-01 | cell fate commitment involved in the formation of primary germ layers |
| 45109 | 5.88E-01 | 6.62E-01 | intermediate filament organization |
| 45346 | 5.88E-01 | 6.62E-01 | regulation of MHC class II biosynthetic process |
| 70306 | 5.88E-01 | 6.62E-01 | lens fiber cell differentiation |
| 70633 | 5.88E-01 | 6.62E-01 | transepithelial transport |
| 46321 | 5.88E-01 | 6.62E-01 | positive regulation of fatty acid oxidation |
| 14074 | 5.88E-01 | 6.62E-01 | response to purine |
| 55070 | 5.88E-01 | 6.62E-01 | copper ion homeostasis |
| 55093 | 5.88E-01 | 6.62E-01 | response to hyperoxia |
| 22401 | 5.88E-01 | 6.62E-01 | negative adaptation of signaling pathway |
| 6105 | 5.88E-01 | 6.62E-01 | succinate metabolic process |
| 30800 | 5.88E-01 | 6.62E-01 | negative regulation of cyclic nucleotide metabolic process |
| 30803 | 5.88E-01 | 6.62E-01 | negative regulation of cyclic nucleotide biosynthetic process |
| 30809 | 5.88E-01 | 6.62E-01 | negative regulation of nucleotide biosynthetic process |
| 31057 | 5.88E-01 | 6.62E-01 | negative regulation of histone modification |
| 6548 | 5.88E-01 | 6.62E-01 | histidine catabolic process |
| 6582 | 5.88E-01 | 6.62E-01 | melanin metabolic process |
| 23058 | 5.88E-01 | 6.62E-01 | adaptation of signaling pathway |
| 6688 | 5.88E-01 | 6.62E-01 | glycosphingolipid biosynthetic process |
| 6707 | 5.88E-01 | 6.62E-01 | cholesterol catabolic process |
| 6740 | 5.88E-01 | 6.62E-01 | NADPH regeneration |
| 15012 | 5.88E-01 | 6.62E-01 | heparan sulfate proteoglycan biosynthetic process |
| 6983 | 5.88E-01 | 6.62E-01 | ER overload response |
| 31663 | 5.88E-01 | 6.62E-01 | lipopolysaccharide-mediated signaling pathway |
| 31952 | 5.88E-01 | 6.62E-01 | regulation of protein amino acid autophosphorylation |
| 48675 | 5.88E-01 | 6.62E-01 | axon extension |
| 15936 | 5.88E-01 | 6.62E-01 | coenzyme A metabolic process |
| 48713 | 5.88E-01 | 6.62E-01 | regulation of oligodendrocyte differentiation |
| 32401 | 5.88E-01 | 6.62E-01 | establishment of melanosome localization |
| 16127 | 5.88E-01 | 6.62E-01 | sterol catabolic process |
| 32715 | 5.88E-01 | 6.62E-01 | negative regulation of interleukin-6 production |
| 32735 | 5.88E-01 | 6.62E-01 | positive regulation of interleukin-12 production |
| 6357 | 5.92E-01 | 6.66E-01 | regulation of transcription from RNA polymerase II promoter |
| 46356 | 5.92E-01 | 6.66E-01 | acetyl-CoA catabolic process |
| 71295 | 5.92E-01 | 6.66E-01 | cellular response to vitamin |
| 31032 | 5.92E-01 | 6.66E-01 | actomyosin structure organization |
| 48538 | 5.92E-01 | 6.66E-01 | thymus development |
| 46328 | 5.93E-01 | 6.67E-01 | regulation of JNK cascade |
| 6968 | 5.98E-01 | 6.73E-01 | cellular defense response |
| 8286 | 5.99E-01 | 6.73E-01 | insulin receptor signaling pathway |
| 55072 | 5.99E-01 | 6.73E-01 | iron ion homeostasis |
| 6984 | 5.99E-01 | 6.73E-01 | ER-nucleus signaling pathway |
| 7585 | 5.99E-01 | 6.73E-01 | respiratory gaseous exchange |
| 6402 | 6.06E-01 | 6.80E-01 | mRNA catabolic process |
| 7286 | 6.06E-01 | 6.80E-01 | spermatid development |
| 9581 | 6.06E-01 | 6.80E-01 | detection of external stimulus |
| 7249 | 6.13E-01 | 6.88E-01 | I-kappaB kinase/NF-kappaB cascade |
| 7338 | 6.13E-01 | 6.88E-01 | single fertilization |
| 35113 | 6.14E-01 | 6.88E-01 | embryonic appendage morphogenesis |
| 30326 | 6.14E-01 | 6.88E-01 | embryonic limb morphogenesis |
| 45893 | 6.14E-01 | 6.88E-01 | positive regulation of transcription, DNA-dependent |
| 42345 | 6.16E-01 | 6.88E-01 | regulation of NF-kappaB import into nucleus |
| 42752 | 6.16E-01 | 6.88E-01 | regulation of circadian rhythm |
| 51785 | 6.16E-01 | 6.88E-01 | positive regulation of nuclear division |
| 2637 | 6.16E-01 | 6.88E-01 | regulation of immunoglobulin production |
| 10970 | 6.16E-01 | 6.88E-01 | microtubule-based transport |
| 45840 | 6.16E-01 | 6.88E-01 | positive regulation of mitosis |
| 45861 | 6.16E-01 | 6.88E-01 | negative regulation of proteolysis |
| 6213 | 6.16E-01 | 6.88E-01 | pyrimidine nucleoside metabolic process |
| 14823 | 6.16E-01 | 6.88E-01 | response to activity |
| 6904 | 6.16E-01 | 6.88E-01 | vesicle docking involved in exocytosis |
| 31575 | 6.16E-01 | 6.88E-01 | G1/S transition checkpoint |
| 9410 | 6.18E-01 | 6.90E-01 | response to xenobiotic stimulus |
| 42475 | 6.18E-01 | 6.90E-01 | odontogenesis of dentine-containing tooth |
| 9799 | 6.18E-01 | 6.90E-01 | specification of symmetry |
| 9855 | 6.18E-01 | 6.90E-01 | determination of bilateral symmetry |
| 43507 | 6.18E-01 | 6.90E-01 | positive regulation of JUN kinase activity |
| 6220 | 6.18E-01 | 6.90E-01 | pyrimidine nucleotide metabolic process |
| 7269 | 6.18E-01 | 6.90E-01 | neurotransmitter secretion |
| 16202 | 6.22E-01 | 6.90E-01 | regulation of striated muscle tissue development |
| 305 | 6.23E-01 | 6.90E-01 | response to oxygen radical |
| 9146 | 6.23E-01 | 6.90E-01 | purine nucleoside triphosphate catabolic process |
| 50482 | 6.23E-01 | 6.90E-01 | arachidonic acid secretion |
| 42354 | 6.23E-01 | 6.90E-01 | L-fucose metabolic process |
| 42534 | 6.23E-01 | 6.90E-01 | regulation of tumor necrosis factor biosynthetic process |
| 1885 | 6.23E-01 | 6.90E-01 | endothelial cell development |
| 43046 | 6.23E-01 | 6.90E-01 | DNA methylation involved in gamete generation |
| 34968 | 6.23E-01 | 6.90E-01 | histone lysine methylation |
| 2204 | 6.23E-01 | 6.90E-01 | somatic recombination of immunoglobulin genes involved in immune response |
| 2208 | 6.23E-01 | 6.90E-01 | somatic diversification of immunoglobulins involved in immune response |
| 35020 | 6.23E-01 | 6.90E-01 | regulation of Rac protein signal transduction |
| 10460 | 6.23E-01 | 6.90E-01 | positive regulation of heart rate |
| 43576 | 6.23E-01 | 6.90E-01 | regulation of respiratory gaseous exchange |
| 60081 | 6.23E-01 | 6.90E-01 | membrane hyperpolarization |
| 51905 | 6.23E-01 | 6.90E-01 | establishment of pigment granule localization |
| 60442 | 6.23E-01 | 6.90E-01 | branching involved in prostate gland morphogenesis |
| 3281 | 6.23E-01 | 6.90E-01 | ventricular septum development |
| 45078 | 6.23E-01 | 6.90E-01 | positive regulation of interferon-gamma biosynthetic process |
| 45190 | 6.23E-01 | 6.90E-01 | isotype switching |
| 45686 | 6.23E-01 | 6.90E-01 | negative regulation of glial cell differentiation |
| 46636 | 6.23E-01 | 6.90E-01 | negative regulation of alpha-beta T cell activation |
| 14037 | 6.23E-01 | 6.90E-01 | Schwann cell differentiation |
| 46825 | 6.23E-01 | 6.90E-01 | regulation of protein export from nucleus |
| 71715 | 6.23E-01 | 6.90E-01 | icosanoid transport |
| 23050 | 6.23E-01 | 6.90E-01 | consequence of signal transmission |
| 31293 | 6.23E-01 | 6.90E-01 | membrane protein intracellular domain proteolysis |
| 7029 | 6.23E-01 | 6.90E-01 | endoplasmic reticulum organization |
| 7080 | 6.23E-01 | 6.90E-01 | mitotic metaphase plate congression |
| 7379 | 6.23E-01 | 6.90E-01 | segment specification |
| 7512 | 6.23E-01 | 6.90E-01 | adult heart development |
| 7520 | 6.23E-01 | 6.90E-01 | myoblast fusion |
| 48521 | 6.23E-01 | 6.90E-01 | negative regulation of behavior |
| 32309 | 6.23E-01 | 6.90E-01 | icosanoid secretion |
| 48708 | 6.23E-01 | 6.90E-01 | astrocyte differentiation |
| 43623 | 6.36E-01 | 7.04E-01 | cellular protein complex assembly |
| 35050 | 6.36E-01 | 7.04E-01 | embryonic heart tube development |
| 21536 | 6.36E-01 | 7.04E-01 | diencephalon development |
| 122 | 6.37E-01 | 7.05E-01 | negative regulation of transcription from RNA polymerase II promoter |
| 33500 | 6.38E-01 | 7.05E-01 | carbohydrate homeostasis |
| 42593 | 6.38E-01 | 7.05E-01 | glucose homeostasis |
| 48634 | 6.38E-01 | 7.05E-01 | regulation of muscle organ development |
| 42755 | 6.38E-01 | 7.05E-01 | eating behavior |
| 35137 | 6.38E-01 | 7.05E-01 | hindlimb morphogenesis |
| 43967 | 6.38E-01 | 7.05E-01 | histone H4 acetylation |
| 21766 | 6.38E-01 | 7.05E-01 | hippocampus development |
| 35270 | 6.46E-01 | 7.13E-01 | endocrine system development |
| 30522 | 6.46E-01 | 7.13E-01 | intracellular receptor mediated signaling pathway |
| 7281 | 6.50E-01 | 7.17E-01 | germ cell development |
| 82 | 6.53E-01 | 7.17E-01 | G1/S transition of mitotic cell cycle |
| 48515 | 6.53E-01 | 7.17E-01 | spermatid differentiation |
| 9880 | 6.54E-01 | 7.17E-01 | embryonic pattern specification |
| 34762 | 6.54E-01 | 7.17E-01 | regulation of transmembrane transport |
| 6888 | 6.54E-01 | 7.17E-01 | ER to Golgi vesicle-mediated transport |
| 768 | 6.55E-01 | 7.17E-01 | syncytium formation by plasma membrane fusion |
| 9071 | 6.55E-01 | 7.17E-01 | serine family amino acid catabolic process |
| 9075 | 6.55E-01 | 7.17E-01 | histidine family amino acid metabolic process |
| 9219 | 6.55E-01 | 7.17E-01 | pyrimidine deoxyribonucleotide metabolic process |
| 1556 | 6.55E-01 | 7.17E-01 | oocyte maturation |
| 34453 | 6.55E-01 | 7.17E-01 | microtubule anchoring |
| 2089 | 6.55E-01 | 7.17E-01 | lens morphogenesis in camera-type eye |
| 2381 | 6.55E-01 | 7.17E-01 | immunoglobulin production involved in immunoglobulin mediated immune response |
| 2446 | 6.55E-01 | 7.17E-01 | neutrophil mediated immunity |
| 19321 | 6.55E-01 | 7.17E-01 | pentose metabolic process |
| 43968 | 6.55E-01 | 7.17E-01 | histone H2A acetylation |
| 45191 | 6.55E-01 | 7.17E-01 | regulation of isotype switching |
| 45581 | 6.55E-01 | 7.17E-01 | negative regulation of T cell differentiation |
| 45671 | 6.55E-01 | 7.17E-01 | negative regulation of osteoclast differentiation |
| 45736 | 6.55E-01 | 7.17E-01 | negative regulation of cyclin-dependent protein kinase activity |
| 45980 | 6.55E-01 | 7.17E-01 | negative regulation of nucleotide metabolic process |
| 46039 | 6.55E-01 | 7.17E-01 | GTP metabolic process |
| 46460 | 6.55E-01 | 7.17E-01 | neutral lipid biosynthetic process |
| 46463 | 6.55E-01 | 7.17E-01 | acylglycerol biosynthetic process |
| 30520 | 6.55E-01 | 7.17E-01 | estrogen receptor signaling pathway |
| 6110 | 6.55E-01 | 7.17E-01 | regulation of glycolysis |
| 6268 | 6.55E-01 | 7.17E-01 | DNA unwinding involved in replication |
| 6547 | 6.55E-01 | 7.17E-01 | histidine metabolic process |
| 6613 | 6.55E-01 | 7.17E-01 | cotranslational protein targeting to membrane |
| 6684 | 6.55E-01 | 7.17E-01 | sphingomyelin metabolic process |
| 7076 | 6.55E-01 | 7.17E-01 | mitotic chromosome condensation |
| 48384 | 6.55E-01 | 7.17E-01 | retinoic acid receptor signaling pathway |
| 48488 | 6.55E-01 | 7.17E-01 | synaptic vesicle endocytosis |
| 7631 | 6.55E-01 | 7.18E-01 | feeding behavior |
| 50877 | 6.57E-01 | 7.19E-01 | neurological system process |
| 31397 | 6.58E-01 | 7.20E-01 | negative regulation of protein ubiquitination |
| 2377 | 6.60E-01 | 7.21E-01 | immunoglobulin production |
| 43966 | 6.60E-01 | 7.21E-01 | histone H3 acetylation |
| 30261 | 6.60E-01 | 7.21E-01 | chromosome condensation |
| 6801 | 6.60E-01 | 7.21E-01 | superoxide metabolic process |
| 7257 | 6.60E-01 | 7.21E-01 | activation of JUN kinase activity |
| 48278 | 6.60E-01 | 7.21E-01 | vesicle docking |
| 32088 | 6.60E-01 | 7.21E-01 | negative regulation of NF-kappaB transcription factor activity |
| 6986 | 6.69E-01 | 7.31E-01 | response to unfolded protein |
| 51052 | 6.73E-01 | 7.35E-01 | regulation of DNA metabolic process |
| 80135 | 6.73E-01 | 7.35E-01 | regulation of cellular response to stress |
| 9109 | 6.80E-01 | 7.42E-01 | coenzyme catabolic process |
| 2440 | 6.80E-01 | 7.42E-01 | production of molecular mediator of immune response |
| 61136 | 6.80E-01 | 7.42E-01 | regulation of proteasomal protein catabolic process |
| 21782 | 6.80E-01 | 7.42E-01 | glial cell development |
| 6305 | 6.80E-01 | 7.42E-01 | DNA alkylation |
| 6306 | 6.80E-01 | 7.42E-01 | DNA methylation |
| 48709 | 6.80E-01 | 7.42E-01 | oligodendrocyte differentiation |
| 32434 | 6.80E-01 | 7.42E-01 | regulation of proteasomal ubiquitin-dependent protein catabolic process |
| 7623 | 6.83E-01 | 7.42E-01 | circadian rhythm |
| 90207 | 6.84E-01 | 7.42E-01 | regulation of triglyceride metabolic process |
| 9247 | 6.84E-01 | 7.42E-01 | glycolipid biosynthetic process |
| 42474 | 6.84E-01 | 7.42E-01 | middle ear morphogenesis |
| 1516 | 6.84E-01 | 7.42E-01 | prostaglandin biosynthetic process |
| 1662 | 6.84E-01 | 7.42E-01 | behavioral fear response |
| 51310 | 6.84E-01 | 7.42E-01 | metaphase plate congression |
| 51646 | 6.84E-01 | 7.42E-01 | mitochondrion localization |
| 10745 | 6.84E-01 | 7.42E-01 | negative regulation of macrophage derived foam cell differentiation |
| 43616 | 6.84E-01 | 7.42E-01 | keratinocyte proliferation |
| 60043 | 6.84E-01 | 7.42E-01 | regulation of cardiac muscle cell proliferation |
| 60716 | 6.84E-01 | 7.42E-01 | labyrinthine layer blood vessel development |
| 21871 | 6.84E-01 | 7.42E-01 | forebrain regionalization |
| 46457 | 6.84E-01 | 7.42E-01 | prostanoid biosynthetic process |
| 55021 | 6.84E-01 | 7.42E-01 | regulation of cardiac muscle tissue growth |
| 55024 | 6.84E-01 | 7.42E-01 | regulation of cardiac muscle tissue development |
| 6525 | 6.84E-01 | 7.42E-01 | arginine metabolic process |
| 6607 | 6.84E-01 | 7.42E-01 | NLS-bearing substrate import into nucleus |
| 31571 | 6.84E-01 | 7.42E-01 | G1/S DNA damage checkpoint |
| 48024 | 6.84E-01 | 7.42E-01 | regulation of nuclear mRNA splicing, via spliceosome |
| 15813 | 6.84E-01 | 7.42E-01 | L-glutamate transport |
| 8088 | 6.84E-01 | 7.42E-01 | axon cargo transport |
| 32729 | 6.84E-01 | 7.42E-01 | positive regulation of interferon-gamma production |
| 1756 | 6.87E-01 | 7.45E-01 | somitogenesis |
| 43506 | 6.87E-01 | 7.45E-01 | regulation of JUN kinase activity |
| 6304 | 6.87E-01 | 7.45E-01 | DNA modification |
| 31016 | 6.87E-01 | 7.45E-01 | pancreas development |
| 226 | 6.94E-01 | 7.52E-01 | microtubule cytoskeleton organization |
| 272 | 7.00E-01 | 7.58E-01 | polysaccharide catabolic process |
| 50715 | 7.00E-01 | 7.58E-01 | positive regulation of cytokine secretion |
| 50792 | 7.00E-01 | 7.58E-01 | regulation of viral reproduction |
| 32409 | 7.03E-01 | 7.61E-01 | regulation of transporter activity |
| 32856 | 7.11E-01 | 7.65E-01 | activation of Ras GTPase activity |
| 33138 | 7.11E-01 | 7.65E-01 | positive regulation of peptidyl-serine phosphorylation |
| 726 | 7.11E-01 | 7.65E-01 | non-recombinational repair |
| 9190 | 7.11E-01 | 7.65E-01 | cyclic nucleotide biosynthetic process |
| 9264 | 7.11E-01 | 7.65E-01 | deoxyribonucleotide catabolic process |
| 9268 | 7.11E-01 | 7.65E-01 | response to pH |
| 42089 | 7.11E-01 | 7.65E-01 | cytokine biosynthetic process |
| 42994 | 7.11E-01 | 7.65E-01 | cytoplasmic sequestering of transcription factor |
| 51193 | 7.11E-01 | 7.65E-01 | regulation of cofactor metabolic process |
| 51196 | 7.11E-01 | 7.65E-01 | regulation of coenzyme metabolic process |
| 2209 | 7.11E-01 | 7.65E-01 | behavioral defense response |
| 43567 | 7.11E-01 | 7.65E-01 | regulation of insulin-like growth factor receptor signaling pathway |
| 10888 | 7.11E-01 | 7.65E-01 | negative regulation of lipid storage |
| 2702 | 7.11E-01 | 7.65E-01 | positive regulation of production of molecular mediator of immune response |
| 51930 | 7.11E-01 | 7.65E-01 | regulation of sensory perception of pain |
| 51931 | 7.11E-01 | 7.65E-01 | regulation of sensory perception |
| 60420 | 7.11E-01 | 7.65E-01 | regulation of heart growth |
| 45909 | 7.11E-01 | 7.65E-01 | positive regulation of vasodilation |
| 14014 | 7.11E-01 | 7.65E-01 | negative regulation of gliogenesis |
| 6027 | 7.11E-01 | 7.65E-01 | glycosaminoglycan catabolic process |
| 6544 | 7.11E-01 | 7.65E-01 | glycine metabolic process |
| 6656 | 7.11E-01 | 7.65E-01 | phosphatidylcholine biosynthetic process |
| 31365 | 7.11E-01 | 7.65E-01 | N-terminal protein amino acid modification |
| 6949 | 7.11E-01 | 7.65E-01 | syncytium formation |
| 48588 | 7.11E-01 | 7.65E-01 | developmental cell growth |
| 45892 | 7.13E-01 | 7.67E-01 | negative regulation of transcription, DNA-dependent |
| 3002 | 7.17E-01 | 7.71E-01 | regionalization |
| 42384 | 7.18E-01 | 7.72E-01 | cilium assembly |
| 43954 | 7.18E-01 | 7.72E-01 | cellular component maintenance |
| 9267 | 7.18E-01 | 7.73E-01 | cellular response to starvation |
| 46474 | 7.19E-01 | 7.73E-01 | glycerophospholipid biosynthetic process |
| 6508 | 7.22E-01 | 7.76E-01 | proteolysis |
| 61053 | 7.33E-01 | 7.87E-01 | somite development |
| 46489 | 7.33E-01 | 7.87E-01 | phosphoinositide biosynthetic process |
| 45665 | 7.35E-01 | 7.87E-01 | negative regulation of neuron differentiation |
| 32319 | 7.35E-01 | 7.87E-01 | regulation of Rho GTPase activity |
| 9132 | 7.35E-01 | 7.87E-01 | nucleoside diphosphate metabolic process |
| 42107 | 7.35E-01 | 7.87E-01 | cytokine metabolic process |
| 9651 | 7.35E-01 | 7.87E-01 | response to salt stress |
| 10165 | 7.35E-01 | 7.87E-01 | response to X-ray |
| 51784 | 7.35E-01 | 7.87E-01 | negative regulation of nuclear division |
| 51925 | 7.35E-01 | 7.87E-01 | regulation of calcium ion transport via voltage-gated calcium channel activity |
| 60444 | 7.35E-01 | 7.87E-01 | branching involved in mammary gland duct morphogenesis |
| 45839 | 7.35E-01 | 7.87E-01 | negative regulation of mitosis |
| 21695 | 7.35E-01 | 7.87E-01 | cerebellar cortex development |
| 30325 | 7.35E-01 | 7.87E-01 | adrenal gland development |
| 14003 | 7.35E-01 | 7.87E-01 | oligodendrocyte development |
| 6376 | 7.35E-01 | 7.87E-01 | mRNA splice site selection |
| 48512 | 7.35E-01 | 7.87E-01 | circadian behavior |
| 48854 | 7.35E-01 | 7.87E-01 | brain morphogenesis |
| 34622 | 7.45E-01 | 7.97E-01 | cellular macromolecular complex assembly |
| 51253 | 7.48E-01 | 8.00E-01 | negative regulation of RNA metabolic process |
| 42158 | 7.48E-01 | 8.01E-01 | lipoprotein biosynthetic process |
| 50953 | 7.50E-01 | 8.02E-01 | sensory perception of light stimulus |
| 7601 | 7.50E-01 | 8.02E-01 | visual perception |
| 33124 | 7.51E-01 | 8.03E-01 | regulation of GTP catabolic process |
| 43087 | 7.51E-01 | 8.03E-01 | regulation of GTPase activity |
| 46620 | 7.52E-01 | 8.03E-01 | regulation of organ growth |
| 6997 | 7.52E-01 | 8.03E-01 | nucleus organization |
| 32412 | 7.52E-01 | 8.03E-01 | regulation of ion transmembrane transporter activity |
| 10605 | 7.58E-01 | 8.08E-01 | negative regulation of macromolecule metabolic process |
| 16447 | 7.58E-01 | 8.08E-01 | somatic recombination of immunoglobulin gene segments |
| 8333 | 7.58E-01 | 8.08E-01 | endosome to lysosome transport |
| 9067 | 7.58E-01 | 8.08E-01 | aspartate family amino acid biosynthetic process |
| 9220 | 7.58E-01 | 8.08E-01 | pyrimidine ribonucleotide biosynthetic process |
| 9584 | 7.58E-01 | 8.08E-01 | detection of visible light |
| 50892 | 7.58E-01 | 8.08E-01 | intestinal absorption |
| 51149 | 7.58E-01 | 8.08E-01 | positive regulation of muscle cell differentiation |
| 70972 | 7.58E-01 | 8.08E-01 | protein localization in endoplasmic reticulum |
| 6270 | 7.58E-01 | 8.08E-01 | DNA-dependent DNA replication initiation |
| 48665 | 7.58E-01 | 8.08E-01 | neuron fate specification |
| 16254 | 7.58E-01 | 8.08E-01 | preassembly of GPI anchor in ER membrane |
| 51053 | 7.60E-01 | 8.10E-01 | negative regulation of DNA metabolic process |
| 43900 | 7.60E-01 | 8.10E-01 | regulation of multi-organism process |
| 41 | 7.63E-01 | 8.12E-01 | transition metal ion transport |
| 51297 | 7.68E-01 | 8.17E-01 | centrosome organization |
| 45727 | 7.68E-01 | 8.17E-01 | positive regulation of translation |
| 6826 | 7.68E-01 | 8.17E-01 | iron ion transport |
| 15031 | 7.72E-01 | 8.21E-01 | protein transport |
| 51329 | 7.72E-01 | 8.21E-01 | interphase of mitotic cell cycle |
| 80 | 7.78E-01 | 8.25E-01 | G1 phase of mitotic cell cycle |
| 50000 | 7.78E-01 | 8.25E-01 | chromosome localization |
| 9218 | 7.78E-01 | 8.25E-01 | pyrimidine ribonucleotide metabolic process |
| 42744 | 7.78E-01 | 8.25E-01 | hydrogen peroxide catabolic process |
| 18298 | 7.78E-01 | 8.25E-01 | protein-chromophore linkage |
| 51303 | 7.78E-01 | 8.25E-01 | establishment of chromosome localization |
| 2474 | 7.78E-01 | 8.25E-01 | antigen processing and presentation of peptide antigen via MHC class I |
| 43470 | 7.78E-01 | 8.25E-01 | regulation of carbohydrate catabolic process |
| 43471 | 7.78E-01 | 8.25E-01 | regulation of cellular carbohydrate catabolic process |
| 45668 | 7.78E-01 | 8.25E-01 | negative regulation of osteoblast differentiation |
| 30104 | 7.78E-01 | 8.25E-01 | water homeostasis |
| 30279 | 7.78E-01 | 8.25E-01 | negative regulation of ossification |
| 46688 | 7.78E-01 | 8.25E-01 | response to copper ion |
| 7032 | 7.78E-01 | 8.25E-01 | endosome organization |
| 7271 | 7.78E-01 | 8.25E-01 | synaptic transmission, cholinergic |
| 48524 | 7.78E-01 | 8.25E-01 | positive regulation of viral reproduction |
| 32321 | 7.78E-01 | 8.25E-01 | positive regulation of Rho GTPase activity |
| 32648 | 7.78E-01 | 8.25E-01 | regulation of interferon-beta production |
| 71466 | 7.82E-01 | 8.28E-01 | cellular response to xenobiotic stimulus |
| 31023 | 7.82E-01 | 8.28E-01 | microtubule organizing center organization |
| 22898 | 7.82E-01 | 8.28E-01 | regulation of transmembrane transporter activity |
| 6805 | 7.82E-01 | 8.28E-01 | xenobiotic metabolic process |
| 6457 | 7.86E-01 | 8.32E-01 | protein folding |
| 19320 | 7.93E-01 | 8.39E-01 | hexose catabolic process |
| 9060 | 7.96E-01 | 8.41E-01 | aerobic respiration |
| 2088 | 7.96E-01 | 8.41E-01 | lens development in camera-type eye |
| 6506 | 7.96E-01 | 8.41E-01 | GPI anchor biosynthetic process |
| 8156 | 7.96E-01 | 8.41E-01 | negative regulation of DNA replication |
| 71216 | 7.97E-01 | 8.41E-01 | cellular response to biotic stimulus |
| 9988 | 7.97E-01 | 8.41E-01 | cell-cell recognition |
| 51220 | 7.97E-01 | 8.41E-01 | cytoplasmic sequestering of protein |
| 35176 | 7.97E-01 | 8.41E-01 | social behavior |
| 2504 | 7.97E-01 | 8.41E-01 | antigen processing and presentation of peptide or polysaccharide antigen via MHC class II |
| 45739 | 7.97E-01 | 8.41E-01 | positive regulation of DNA repair |
| 30901 | 7.97E-01 | 8.41E-01 | midbrain development |
| 7098 | 7.97E-01 | 8.41E-01 | centrosome cycle |
| 9566 | 8.02E-01 | 8.46E-01 | fertilization |
| 51325 | 8.05E-01 | 8.49E-01 | interphase |
| 45184 | 8.07E-01 | 8.51E-01 | establishment of protein localization |
| 9952 | 8.09E-01 | 8.53E-01 | anterior/posterior pattern formation |
| 19722 | 8.09E-01 | 8.53E-01 | calcium-mediated signaling |
| 6505 | 8.09E-01 | 8.53E-01 | GPI anchor metabolic process |
| 7569 | 8.09E-01 | 8.53E-01 | cell aging |
| 16481 | 8.11E-01 | 8.54E-01 | negative regulation of transcription |
| 16445 | 8.14E-01 | 8.57E-01 | somatic diversification of immunoglobulins |
| 42992 | 8.14E-01 | 8.57E-01 | negative regulation of transcription factor import into nucleus |
| 43603 | 8.14E-01 | 8.57E-01 | cellular amide metabolic process |
| 31018 | 8.14E-01 | 8.57E-01 | endocrine pancreas development |
| 31640 | 8.14E-01 | 8.57E-01 | killing of cells of another organism |
| 48536 | 8.14E-01 | 8.57E-01 | spleen development |
| 32479 | 8.14E-01 | 8.57E-01 | regulation of type I interferon production |
| 32313 | 8.19E-01 | 8.61E-01 | regulation of Rab GTPase activity |
| 32483 | 8.19E-01 | 8.61E-01 | regulation of Rab protein signal transduction |
| 70 | 8.22E-01 | 8.63E-01 | mitotic sister chromatid segregation |
| 50707 | 8.22E-01 | 8.63E-01 | regulation of cytokine secretion |
| 51352 | 8.22E-01 | 8.63E-01 | negative regulation of ligase activity |
| 51444 | 8.22E-01 | 8.63E-01 | negative regulation of ubiquitin-protein ligase activity |
| 46365 | 8.22E-01 | 8.63E-01 | monosaccharide catabolic process |
| 51438 | 8.27E-01 | 8.69E-01 | regulation of ubiquitin-protein ligase activity |
| 51172 | 8.29E-01 | 8.70E-01 | negative regulation of nitrogen compound metabolic process |
| 578 | 8.30E-01 | 8.71E-01 | embryonic axis specification |
| 42551 | 8.30E-01 | 8.71E-01 | neuron maturation |
| 42787 | 8.30E-01 | 8.71E-01 | protein ubiquitination involved in ubiquitin-dependent protein catabolic process |
| 51318 | 8.30E-01 | 8.71E-01 | G1 phase |
| 2763 | 8.30E-01 | 8.71E-01 | positive regulation of myeloid leukocyte differentiation |
| 31572 | 8.30E-01 | 8.71E-01 | G2/M transition DNA damage checkpoint |
| 17148 | 8.33E-01 | 8.73E-01 | negative regulation of translation |
| 819 | 8.33E-01 | 8.73E-01 | sister chromatid segregation |
| 9798 | 8.33E-01 | 8.73E-01 | axis specification |
| 3001 | 8.43E-01 | 8.83E-01 | generation of a signal involved in cell-cell signaling |
| 23061 | 8.43E-01 | 8.83E-01 | signal release |
| 9583 | 8.44E-01 | 8.83E-01 | detection of light stimulus |
| 9394 | 8.45E-01 | 8.83E-01 | 2'-deoxyribonucleotide metabolic process |
| 42308 | 8.45E-01 | 8.83E-01 | negative regulation of protein import into nucleus |
| 34620 | 8.45E-01 | 8.83E-01 | cellular response to unfolded protein |
| 19048 | 8.45E-01 | 8.83E-01 | virus-host interaction |
| 6026 | 8.45E-01 | 8.83E-01 | aminoglycan catabolic process |
| 30968 | 8.45E-01 | 8.83E-01 | endoplasmic reticulum unfolded protein response |
| 7224 | 8.45E-01 | 8.83E-01 | smoothened signaling pathway |
| 15850 | 8.45E-01 | 8.83E-01 | organic alcohol transport |
| 15909 | 8.45E-01 | 8.83E-01 | long-chain fatty acid transport |
| 10558 | 8.47E-01 | 8.85E-01 | negative regulation of macromolecule biosynthetic process |
| 6497 | 8.48E-01 | 8.86E-01 | protein amino acid lipidation |
| 51340 | 8.50E-01 | 8.88E-01 | regulation of ligase activity |
| 8213 | 8.55E-01 | 8.92E-01 | protein amino acid alkylation |
| 6479 | 8.55E-01 | 8.92E-01 | protein amino acid methylation |
| 51443 | 8.55E-01 | 8.93E-01 | positive regulation of ubiquitin-protein ligase activity |
| 7218 | 8.57E-01 | 8.94E-01 | neuropeptide signaling pathway |
| 16444 | 8.58E-01 | 8.94E-01 | somatic cell DNA recombination |
| 9948 | 8.58E-01 | 8.94E-01 | anterior/posterior axis specification |
| 2562 | 8.58E-01 | 8.94E-01 | somatic diversification of immune receptors via germline recombination within a single locus |
| 51817 | 8.58E-01 | 8.94E-01 | modification of morphology or physiology of other organism involved in symbiotic interaction |
| 6342 | 8.58E-01 | 8.94E-01 | chromatin silencing |
| 31056 | 8.58E-01 | 8.94E-01 | regulation of histone modification |
| 8038 | 8.58E-01 | 8.94E-01 | neuron recognition |
| 90305 | 8.64E-01 | 9.00E-01 | nucleic acid phosphodiester bond hydrolysis |
| 30521 | 8.64E-01 | 9.00E-01 | androgen receptor signaling pathway |
| 34621 | 8.65E-01 | 9.01E-01 | cellular macromolecular complex subunit organization |
| 1702 | 8.70E-01 | 9.05E-01 | gastrulation with mouth forming second |
| 51180 | 8.70E-01 | 9.05E-01 | vitamin transport |
| 6221 | 8.70E-01 | 9.05E-01 | pyrimidine nucleotide biosynthetic process |
| 43414 | 8.70E-01 | 9.05E-01 | macromolecule methylation |
| 6401 | 8.70E-01 | 9.05E-01 | RNA catabolic process |
| 32259 | 8.70E-01 | 9.05E-01 | methylation |
| 9416 | 8.71E-01 | 9.05E-01 | response to light stimulus |
| 48232 | 8.72E-01 | 9.07E-01 | male gamete generation |
| 7283 | 8.72E-01 | 9.07E-01 | spermatogenesis |
| 42594 | 8.72E-01 | 9.07E-01 | response to starvation |
| 6308 | 8.72E-01 | 9.07E-01 | DNA catabolic process |
| 6119 | 8.73E-01 | 9.07E-01 | oxidative phosphorylation |
| 956 | 8.73E-01 | 9.07E-01 | nuclear-transcribed mRNA catabolic process |
| 51351 | 8.77E-01 | 9.10E-01 | positive regulation of ligase activity |
| 17038 | 8.77E-01 | 9.10E-01 | protein import |
| 6338 | 8.80E-01 | 9.13E-01 | chromatin remodeling |
| 2200 | 8.81E-01 | 9.14E-01 | somatic diversification of immune receptors |
| 46823 | 8.81E-01 | 9.14E-01 | negative regulation of nucleocytoplasmic transport |
| 32320 | 8.81E-01 | 9.14E-01 | positive regulation of Ras GTPase activity |
| 33044 | 8.82E-01 | 9.14E-01 | regulation of chromosome organization |
| 21510 | 8.82E-01 | 9.14E-01 | spinal cord development |
| 6096 | 8.82E-01 | 9.14E-01 | glycolysis |
| 6606 | 8.83E-01 | 9.15E-01 | protein import into nucleus |
| 77 | 8.87E-01 | 9.18E-01 | DNA damage checkpoint |
| 9953 | 8.87E-01 | 9.18E-01 | dorsal/ventral pattern formation |
| 51783 | 8.87E-01 | 9.18E-01 | regulation of nuclear division |
| 7088 | 8.87E-01 | 9.18E-01 | regulation of mitosis |
| 7254 | 8.87E-01 | 9.18E-01 | JNK cascade |
| 21953 | 8.89E-01 | 9.20E-01 | central nervous system neuron differentiation |
| 6839 | 8.89E-01 | 9.20E-01 | mitochondrial transport |
| 16571 | 8.91E-01 | 9.21E-01 | histone methylation |
| 45670 | 8.91E-01 | 9.21E-01 | regulation of osteoclast differentiation |
| 70301 | 8.91E-01 | 9.21E-01 | cellular response to hydrogen peroxide |
| 45814 | 8.91E-01 | 9.21E-01 | negative regulation of gene expression, epigenetic |
| 30010 | 8.91E-01 | 9.21E-01 | establishment of cell polarity |
| 6282 | 8.91E-01 | 9.21E-01 | regulation of DNA repair |
| 31576 | 8.91E-01 | 9.21E-01 | G2/M transition checkpoint |
| 48002 | 8.91E-01 | 9.21E-01 | antigen processing and presentation of peptide antigen |
| 32318 | 8.97E-01 | 9.26E-01 | regulation of Ras GTPase activity |
| 50909 | 8.97E-01 | 9.27E-01 | sensory perception of taste |
| 6417 | 8.98E-01 | 9.27E-01 | regulation of translation |
| 7017 | 8.99E-01 | 9.28E-01 | microtubule-based process |
| 9620 | 9.00E-01 | 9.29E-01 | response to fungus |
| 6284 | 9.00E-01 | 9.29E-01 | base-excision repair |
| 10629 | 9.00E-01 | 9.29E-01 | negative regulation of gene expression |
| 51170 | 9.01E-01 | 9.29E-01 | nuclear import |
| 6807 | 9.04E-01 | 9.32E-01 | nitrogen compound metabolic process |
| 51436 | 9.06E-01 | 9.34E-01 | negative regulation of ubiquitin-protein ligase activity involved in mitotic cell cycle |
| 18 | 9.09E-01 | 9.36E-01 | regulation of DNA recombination |
| 9262 | 9.09E-01 | 9.36E-01 | deoxyribonucleotide metabolic process |
| 71445 | 9.09E-01 | 9.36E-01 | cellular response to protein stimulus |
| 6493 | 9.09E-01 | 9.36E-01 | protein amino acid O-linked glycosylation |
| 30216 | 9.12E-01 | 9.38E-01 | keratinocyte differentiation |
| 31098 | 9.12E-01 | 9.38E-01 | stress-activated protein kinase signaling cascade |
| 31145 | 9.12E-01 | 9.38E-01 | anaphase-promoting complex-dependent proteasomal ubiquitin-dependent protein catabolic process |
| 31570 | 9.12E-01 | 9.38E-01 | DNA integrity checkpoint |
| 45934 | 9.13E-01 | 9.40E-01 | negative regulation of nucleobase, nucleoside, nucleotide and nucleic acid metabolic process |
| 7018 | 9.13E-01 | 9.40E-01 | microtubule-based movement |
| 90317 | 9.17E-01 | 9.42E-01 | negative regulation of intracellular protein transport |
| 43484 | 9.17E-01 | 9.42E-01 | regulation of RNA splicing |
| 31123 | 9.17E-01 | 9.42E-01 | RNA 3'-end processing |
| 15908 | 9.17E-01 | 9.42E-01 | fatty acid transport |
| 51437 | 9.17E-01 | 9.42E-01 | positive regulation of ubiquitin-protein ligase activity involved in mitotic cell cycle |
| 45333 | 9.18E-01 | 9.43E-01 | cellular respiration |
| 43543 | 9.22E-01 | 9.47E-01 | protein amino acid acylation |
| 22904 | 9.22E-01 | 9.47E-01 | respiratory electron transport chain |
| 32012 | 9.23E-01 | 9.48E-01 | regulation of ARF protein signal transduction |
| 43170 | 9.24E-01 | 9.48E-01 | macromolecule metabolic process |
| 51603 | 9.26E-01 | 9.50E-01 | proteolysis involved in cellular protein catabolic process |
| 30163 | 9.28E-01 | 9.52E-01 | protein catabolic process |
| 7051 | 9.28E-01 | 9.52E-01 | spindle organization |
| 6879 | 9.30E-01 | 9.54E-01 | cellular iron ion homeostasis |
| 32387 | 9.30E-01 | 9.54E-01 | negative regulation of intracellular transport |
| 51301 | 9.33E-01 | 9.56E-01 | cell division |
| 44257 | 9.33E-01 | 9.56E-01 | cellular protein catabolic process |
| 16573 | 9.33E-01 | 9.56E-01 | histone acetylation |
| 7219 | 9.33E-01 | 9.56E-01 | Notch signaling pathway |
| 16569 | 9.35E-01 | 9.58E-01 | covalent chromatin modification |
| 9913 | 9.36E-01 | 9.58E-01 | epidermal cell differentiation |
| 51439 | 9.36E-01 | 9.58E-01 | regulation of ubiquitin-protein ligase activity involved in mitotic cell cycle |
| 9057 | 9.36E-01 | 9.58E-01 | macromolecule catabolic process |
| 70585 | 9.41E-01 | 9.63E-01 | protein localization in mitochondrion |
| 6626 | 9.41E-01 | 9.63E-01 | protein targeting to mitochondrion |
| 7602 | 9.41E-01 | 9.63E-01 | phototransduction |
| 32312 | 9.41E-01 | 9.63E-01 | regulation of ARF GTPase activity |
| 7093 | 9.42E-01 | 9.64E-01 | mitotic cell cycle checkpoint |
| 6007 | 9.46E-01 | 9.68E-01 | glucose catabolic process |
| 70925 | 9.50E-01 | 9.71E-01 | organelle assembly |
| 245 | 9.51E-01 | 9.72E-01 | spliceosome assembly |
| 34976 | 9.51E-01 | 9.72E-01 | response to endoplasmic reticulum stress |
| 6473 | 9.54E-01 | 9.74E-01 | protein amino acid acetylation |
| 2831 | 9.59E-01 | 9.79E-01 | regulation of response to biotic stimulus |
| 6612 | 9.59E-01 | 9.79E-01 | protein targeting to membrane |
| 42773 | 9.60E-01 | 9.80E-01 | ATP synthesis coupled electron transport |
| 42775 | 9.60E-01 | 9.80E-01 | mitochondrial ATP synthesis coupled electron transport |
| 44265 | 9.62E-01 | 9.81E-01 | cellular macromolecule catabolic process |
| 6730 | 9.62E-01 | 9.81E-01 | one-carbon metabolic process |
| 51168 | 9.63E-01 | 9.82E-01 | nuclear export |
| 6261 | 9.63E-01 | 9.82E-01 | DNA-dependent DNA replication |
| 16570 | 9.64E-01 | 9.83E-01 | histone modification |
| 34614 | 9.66E-01 | 9.84E-01 | cellular response to reactive oxygen species |
| 6913 | 9.68E-01 | 9.86E-01 | nucleocytoplasmic transport |
| 34641 | 9.69E-01 | 9.87E-01 | cellular nitrogen compound metabolic process |
| 51169 | 9.69E-01 | 9.87E-01 | nuclear transport |
| 6414 | 9.69E-01 | 9.87E-01 | translational elongation |
| 6302 | 9.70E-01 | 9.88E-01 | double-strand break repair |
| 46879 | 9.71E-01 | 9.89E-01 | hormone secretion |
| 31424 | 9.74E-01 | 9.91E-01 | keratinization |
| 6325 | 9.76E-01 | 9.93E-01 | chromatin organization |
| 7059 | 9.76E-01 | 9.93E-01 | chromosome segregation |
| 6120 | 9.78E-01 | 9.95E-01 | mitochondrial electron transport, NADH to ubiquinone |
| 6914 | 9.78E-01 | 9.95E-01 | autophagy |
| 22402 | 9.78E-01 | 9.95E-01 | cell cycle process |
| 9914 | 9.80E-01 | 9.96E-01 | hormone transport |
| 44260 | 9.81E-01 | 9.97E-01 | cellular macromolecule metabolic process |
| 6310 | 9.81E-01 | 9.97E-01 | DNA recombination |
| 6446 | 9.82E-01 | 9.97E-01 | regulation of translational initiation |
| 75 | 9.84E-01 | 9.99E-01 | cell cycle checkpoint |
| 43632 | 9.84E-01 | 9.99E-01 | modification-dependent macromolecule catabolic process |
| 19941 | 9.84E-01 | 9.99E-01 | modification-dependent protein catabolic process |
| 50890 | 9.85E-01 | 1.00E+00 | cognition |
| 16579 | 9.86E-01 | 1 | protein deubiquitination |
| 40029 | 9.86E-01 | 1 | regulation of gene expression, epigenetic |
| 51224 | 9.87E-01 | 1 | negative regulation of protein transport |
| 70646 | 9.89E-01 | 1 | protein modification by small protein removal |
| 6354 | 9.89E-01 | 1 | RNA elongation |
| 19222 | 9.89E-01 | 1 | regulation of metabolic process |
| 6511 | 9.90E-01 | 1 | ubiquitin-dependent protein catabolic process |
| 6366 | 9.90E-01 | 1 | transcription from RNA polymerase II promoter |
| 16568 | 9.91E-01 | 1 | chromatin modification |
| 33554 | 9.93E-01 | 1 | cellular response to stress |
| 48285 | 9.94E-01 | 1 | organelle fission |
| 31323 | 9.95E-01 | 1 | regulation of cellular metabolic process |
| 280 | 9.96E-01 | 1 | nuclear division |
| 7067 | 9.96E-01 | 1 | mitosis |
| 9411 | 9.96E-01 | 1 | response to UV |
| 7049 | 9.97E-01 | 1 | cell cycle |
| 278 | 9.97E-01 | 1 | mitotic cell cycle |
| 87 | 9.97E-01 | 1 | M phase of mitotic cell cycle |
| 16458 | 9.97E-01 | 1 | gene silencing |
| 32774 | 9.98E-01 | 1 | RNA biosynthetic process |
| 51276 | 9.98E-01 | 1 | chromosome organization |
| 6974 | 9.98E-01 | 1 | response to DNA damage stimulus |
| 51327 | 9.99E-01 | 1 | M phase of meiotic cell cycle |
| 7126 | 9.99E-01 | 1 | meiosis |
| 51321 | 9.99E-01 | 1 | meiotic cell cycle |
| 6260 | 9.99E-01 | 1 | DNA replication |
| 6351 | 9.99E-01 | 1 | transcription, DNA-dependent |
| 22618 | 9.99E-01 | 1 | ribonucleoprotein complex assembly |
| 22403 | 9.99E-01 | 1 | cell cycle phase |
| 16567 | 9.99E-01 | 1 | protein ubiquitination |
| 6350 | 9.99E-01 | 1 | transcription |
| 6352 | 9.99E-01 | 1 | transcription initiation |
| 377 | 1.00E+00 | 1 | RNA splicing, via transesterification reactions with bulged adenosine as nucleophile |
| 398 | 1.00E+00 | 1 | nuclear mRNA splicing, via spliceosome |
| 32446 | 1.00E+00 | 1 | protein modification by small protein conjugation |
| 279 | 1.00E+00 | 1 | M phase |
| 6259 | 1.00E+00 | 1 | DNA metabolic process |
| 80090 | 1.00E+00 | 1 | regulation of primary metabolic process |
| 6364 | 1.00E+00 | 1 | rRNA processing |
| 375 | 1.00E+00 | 1 | RNA splicing, via transesterification reactions |
| 16072 | 1.00E+00 | 1 | rRNA metabolic process |
| 16071 | 1.00E+00 | 1 | mRNA metabolic process |
| 9059 | 1.00E+00 | 1 | macromolecule biosynthetic process |
| 70647 | 1.00E+00 | 1 | protein modification by small protein conjugation or removal |
| 15931 | 1.00E+00 | 1 | nucleobase, nucleoside, nucleotide and nucleic acid transport |
| 9889 | 1.00E+00 | 1 | regulation of biosynthetic process |
| 60255 | 1.00E+00 | 1 | regulation of macromolecule metabolic process |
| 42254 | 1.00E+00 | 1 | ribosome biogenesis |
| 6281 | 1.00E+00 | 1 | DNA repair |
| 31326 | 1.00E+00 | 1 | regulation of cellular biosynthetic process |
| 34645 | 1 | 1 | cellular macromolecule biosynthetic process |
| 7600 | 1 | 1 | sensory perception |
| 51171 | 1 | 1 | regulation of nitrogen compound metabolic process |
| 6397 | 1 | 1 | mRNA processing |
| 6412 | 1 | 1 | translation |
| 22613 | 1 | 1 | ribonucleoprotein complex biogenesis |
| 6139 | 1 | 1 | nucleobase, nucleoside, nucleotide and nucleic acid metabolic process |
| 19219 | 1 | 1 | regulation of nucleobase, nucleoside, nucleotide and nucleic acid metabolic process |
| 8380 | 1 | 1 | RNA splicing |
| 10468 | 1 | 1 | regulation of gene expression |
| 10556 | 1 | 1 | regulation of macromolecule biosynthetic process |
| 51252 | 1 | 1 | regulation of RNA metabolic process |
| 34470 | 1 | 1 | ncRNA processing |
| 6355 | 1 | 1 | regulation of transcription, DNA-dependent |
| 45449 | 1 | 1 | regulation of transcription |
| 34660 | 1 | 1 | ncRNA metabolic process |
| 10467 | 1 | 1 | gene expression |
| 16070 | 1 | 1 | RNA metabolic process |
| 6396 | 1 | 1 | RNA processing |
| 90304 | 1 | 1 | nucleic acid metabolic process |
| 7606 | 1 | 1 | sensory perception of chemical stimulus |
| 7608 | 1 | 1 | sensory perception of smell |
| 8150 | 1 | 1 | biological_process |
